# Stereochemistry Drives the Macromolecular Conformation and Biological Activity of Glycopolymers

**DOI:** 10.1021/acscentsci.5c00768

**Published:** 2025-08-06

**Authors:** Muhammad Waqas Ishaq, Parisa Farzeen, Lindsay R. Vaughn, Daniel J. Stone, Sanket A. Deshmukh, Cassandra E. Callmann

**Affiliations:** † Department of Chemistry, 118710 University of Texas at Austin, Austin, Texas 78712, United States; ‡ Department of Chemical Engineering, 1757 Virginia Tech, Blacksburg, Virginia 24061, United States

## Abstract

Chirality plays a fundamental role in biology, where stereochemical information governs how molecules fold, interact, and function. While the effects of stereochemistry are well-established for small molecules and natural biomacromolecules, less is known about how it shapes the properties of synthetic, biomimetic polymers. In this study, we explore how backbone and glycan stereochemistry influences conformation, physical interactions, and biological behavior in water-soluble glycopolymers. Using ring opening metathesis polymerization (ROMP), we synthesized precision glycopolymers (PGPs) from two diastereomeric norbornenyl moieties (*endo* and *exo*) and monosaccharides (glucose, galactose, and mannose). Despite having nearly identical molecular and macromolecular compositions, the resulting PGPs displayed major differences in their physical and biological properties. Glycopolymers with β-linkages showed distinct circular dichroism (CD) signals, and *exo*-derived backbones displayed more hydrophobic local environments, as confirmed by all-atom molecular dynamics simulations and dye interaction studies. These structural differences had clear functional consequences. *exo*-PGPs bound plant lectins more rapidly and with higher avidity, whereas *endo*-PGPs showed greater selectivity toward human galectin-3, stronger inhibition of cholera toxin, and enhanced uptake into 4T1 triple-negative breast cancer cells. Together, these findings provide the first demonstration of biological activity in *endo*-derived glycopolymers and establish backbone stereochemistry as a key design element that encodes macromolecular behavior in biologically relevant contexts.

## Graphical abstract

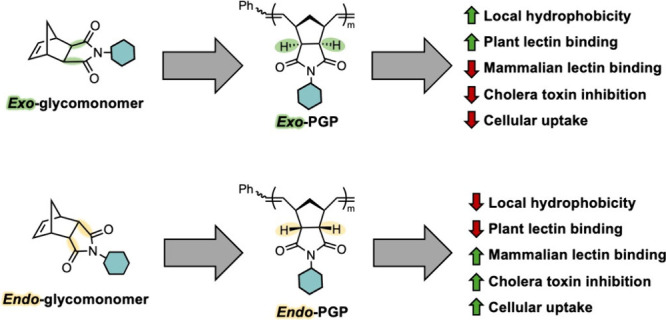

## Introduction

The stereochemistry of life’s building blocks (i.e., amino acids, sugars, and nucleic acids) is essential for the structure and function of biological macromolecules. This molecular-level information governs folding, recognition, and interactions that underlie key cellular processes. Enzymes and receptors rely on well-defined chiral architectures to recognize specific ligands,^1^ and even minor changes in stereochemistry can lead to major changes in biological activity. In turn, these relationships are central to processes like immune signaling, metabolic regulation, and pathogen recognition.

Given the importance of stereochemistry, there has been significant emphasis in drug development on producing stereochemically pure, structurally defined small molecules.^2–4^ However, much less is known about how stereochemical information impacts the behavior of synthetic and biomimetic polymers, particularly those intended to function in biological environments. As interest grows in designing materials that can mimic or engage biological systems,^5–7^ it becomes increasingly important to understand how stereochemistry influences polymer conformation and function.

Recent studies have shown that stereochemistry in synthetic macromolecules can dramatically influence their biological properties. For example, it has been shown that the absolute configuration of glycooligomers affects their lectin-binding selectivity.^8^ In other systems, tuning the ratio of cis versus trans units in the polymer backbone alters the overall chain conformation, leading to differences in binding strength despite identical monomer composition.^9^ Moreover, side-chain chirality impacts polymer uptake, cytotoxicity, and biodistribution in vivo.^10^ These findings highlight that stereochemical control is not only relevant to small molecules, but also plays a critical role in dictating how larger, more complex systems behave in biological contexts. Despite this growing interest, no studies to date have investigated the biological activity of glycopolymers derived from *endo*-substituted norbornene monomers, leaving an entire stereochemical space unexplored.

Motivated by this, we sought herein to investigate how polymer backbone stereochemistry shapes the physical and biological properties of synthetic glycopolymers. To this end, we first synthesized a panel of glycomonomers (Figure 1a) that differ in the stereochemical configuration of the norbornenyl core (*exo* vs *endo*), the identity of the pendant sugar (glucose, galactose, or mannose), and the glycosidic linkage (alpha vs beta). To reflect biologically relevant recognition patterns, we selected glycosidic linkages that mirror the native configurations for each sugar: β for glucose and galactose, and α for mannose. Monomers were prepared via a modular click reaction between azide-functionalized monosaccharides and alkyne-functionalized norbornenes. These monomers were then polymerized using protecting-group-free, single-solvent ring-opening metathesis polymerization (ROMP) to yield a series of glycopolymers (Figure 1b). Owing to their unambiguous local chemical composition and well-defined global structure, we refer to these materials as precision glycopolymers (PGPs). All PGPs exhibited high water solubility and solution dimensions comparable to those of small proteins, making them well suited for probing structure–function relationships in biologically relevant environments.^10^

**Figure 1:**
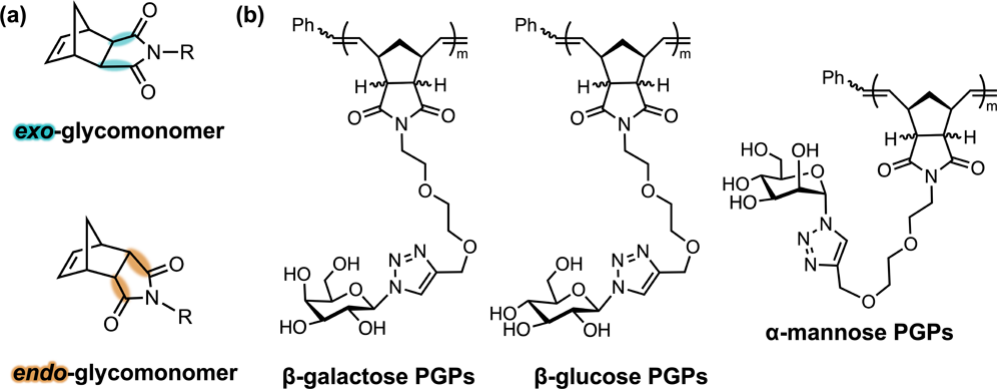
(a) Structures of glycomonomers synthesized using *exo*-norbornene (top) and *endo*-norbornene (bottom). R = sugar epitope + linker. (b) General structure of precision glycopolymers (PGPs) synthesized using *exo*- and *endo*-β-galactose (left), *exo*- and *endo*-β-glucose (middle), and *exo*- and *endo*-α-mannose (right).

We then used CD spectroscopy, 2D-NMR, and hydrophobic molecule inclusion assays to characterize the conformational properties of the PGPs, and found that stereochemistry directly impacts self-association, hydrophobicity, and protein binding. Notably, *exo*-derived backbones formed more hydrophobic structures and bound plant lectins with higher avidity, while *endo*-PGPs were more selective for human lectins and showed greater uptake into mammalian cells. Together, these findings underscore how subtle changes in stereochemistry translate to meaningful differences in function, offering a path toward designing glycomaterials that more precisely engage biological targets.

## Results and Discussion

### PGP Design and Synthesis

Starting from two commercially ROMP-amenable moieties (cis-5-Norbornene-*exo*-2,3-dicarboxylic anhydride and cis-5-Norbornene-*endo*-2,3-dicarboxylic anhydride), we performed a condensation reaction with amino-PEG_2_-alcohol “linkers” to afford two molecules with hydroxyl moieties amenable to glycosylation reactions, termed *exo*-OH, and *endo*-OH, respectively (Scheme S1). We then modified both norbornene linkers with alkyne groups and subjected them to copper-catalyzed azide–alkyne cycloaddition (CuAAC) with either acetyl-protected 1-azido-galactose, 1-azido-glucose, or 1-azido-mannose, to yield 6 “protected” glycomonomers: *exo*-β-Gal-OAc, *endo*-β-Gal-OAc, *exo*-β-Glc-OAc, *endo*-β-Glc-OAc, *exo*-α-Man-OAc, and *endo*-α-Man-OAc (Scheme S2–S4). Deprotection of these acetyl-protected glycomonomers resulted in 6 final, protecting-group-free glycomonomers: *exo*-β-Gal, *endo*-β-Gal, *exo*-β-Glc, *endo*-β-Glc, *exo*-α-Man, and *endo*-α-Man (Scheme S5–S7).

We then subjected all glycomonomers to ROMP using Grubbs third-generation catalyst in dimethylformamide (DMF) at room temperature.^11–13^ We used ^1^H NMR to evaluate their respective polymerization rates and extent of conversion to PGPs (Figure 2). As expected, all *exo*-norbornene monomers showed rapid conversion to polymers (Figure 2a), as indicated by the linearity of the plot, which followed pseudo first order kinetics (Figure 2b). Monomer identity had a significant impact on the rate of polymerization, where the hydroxylated side chain (*exo*-OH and *endo*-OH) polymerized the fastest, followed by acetylated sugars, and then finally the protecting group-free sugars. This is further supported by the apparent propagation rate constant (*k*_p,app_), where the slope of this semilogarithmic plot is utilized to determine the *k*_p,app_ (Table 1). The calculated *k*_p,app_ for *exo*-OH, *exo*-β-Gal-OAc, and *exo*-β-Gal are 0.60, 0.37, and 0.24 min^–1^ respectively. This indicates that the monomer end group strongly influences the rate of polymerization (*exo*-OH > *exo*-β-Gal-OAc > *exo*-β-Gal). Surprisingly, sugar epitope also impacted on the rate of polymerization (Figure 2 **c-d**). The calculated *k*_p,app_ for *exo*-β-Gal, *exo*-β-Glc, and *exo*-α-Man are 0.24, 0.17, and 0.34 min^–1^ respectively (*exo*-α-Man > *exo*-β-Gal ≌ *exo*-β-Glc).

**Table 1:** SEC-MAL Data and Kinetic Data for *exo-*PGPs

Polymer	Target DP	Theo. *M*_n_(KDa)	*M*_n_ (KDa)	*M*_w_(KDa)	Đ	DP^a^	*K*_p_^app^(min^–1^)
*exo*-OH	35	8.79	7.1	7.2	1.01	29	0.60
*exo*-β-Gal	35	17.31	16.0	16.4	1.02	33	0.24
*exo*-Gal-OAc	35	23.19	21.1	21.6	1.02	32	0.37
*exo*-β-Glc	35	17.31	21.1	25.0	1.17	42	0.17
*exo*-α-Man	35	17.31	19.2	19.9	1.03	38	0.34

a DP calculated as *M*_n_/MW_monomer_

**Figure 2:**
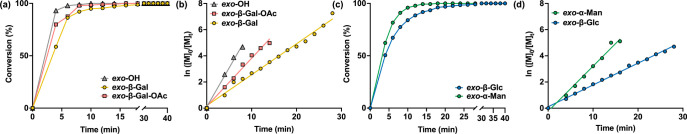
(a) Conversion of *exo*-monomers with different end groups as a function of time, as measured by ^1^H NMR. (b) Log plots of the conversion of the *exo*-monomer. Lines represent lines of best fit, using linear least-squares fitting. (c) Conversion of *exo*-PGPs with different sugars (β-Gal, β-Glc, and α-Man) as a function of time, as measured by ^1^H NMR. (d) Log plots of the conversion of *exo*-PGPs. Lines represent lines of best fit, using linear least-squares fitting.

As expected, all *endo*-norbornene monomers polymerized significantly slower than their *exo*-norbornene counterparts under the same conditions (Figure S1a). For instance, *endo*-OH polymerizes 30-fold more slowly than *exo*-OH. This observation aligns with prior literature,^14–16^ which has noted that *exo*-monomers for ROMP display enhanced reactivity and polymerization control as compared to their *endo*-counterparts, owing to their reduced steric hindrance. Moreover, while *endo*-OH reached complete conversion, at room temperature, neither *endo*-Gal-OAc-RT nor *endo*-β-Gal-RT showed complete conversion, stalling at degree of polymerization ≌ 40. This suggests that the *endo*-norbornene stereochemistry and presence of bulky, multifunctional sugar moieties may allow the monomers to adapt a conformation that interferes with the Ru catalyst, thus inhibiting polymerization.^15,16^ To remedy this, we optimized several parameters (Table S1), including catalyst concentration (0.06 mol/L), polymerization temperature (60 °C), and choice of Ru initiator, until we arrived at a set of conditions that afforded *endo*-PGPs with low dispersity and calculated DPs that closely match the target DP (Table 2). As was observed in their *exo*-norbornene analogues, the rate of polymerization (Figure S1b-d) of *endo*-PGPs was also dependent on sugar epitope identity. Thus, it is evident that sugar stereochemistry significantly influences rate of polymerization along with the stereochemistry of backbone.

**Table 2:** SEC-MAL Data and Kinetic Data for *endo-*PGPs

PGP	Target DP	Theo. *M*_n_ (KDa)	*M*_n_ (KDa)	*M*_w_ (KDa)	Temp	Đ	DP^a^	*K*_p_^app^ (min^–1^)
*endo*-OH	35	8.79	7.0	7.4	rt	1. 06	29	0.019
*endo*-β-Gal-RT	35	17.31	7.2	6.2	rt	1.15	14	0.004
*endo*-Gal-OAc-RT	35	23.19	13.0	26.0	rt	1.11	20	0.009
*endo*-β-Gal	35	17.31	18.0	19.0	60 °C	1.09	36	
*endo*-β-Glc	35	17,31	18.7	20.9	60 °C	1.16	36	
*endo*-α-Man	35	17.31	16.6	18.7	60 °C	1.03	34	

a DP calculated as *M*_n_/MW_monomer_

### Physical Analyses of PGPs

To understand how the monomer-level stereochemistry and macromolecular conformation dictate glycopolymers properties, we first investigated the secondary structure of all PGPs using circular dichroism spectroscopy (CD) and Nuclear Overhauser Effect Spectroscopy (NOESY) NMR. Differing CD spectra were observed for PGPs depending on sugar epitope identity in both water and phosphate-buffered saline (Figure 3), yet no dependence was observed as a function of ROMP backbone chirality. Instead, a strong dependency on the sugar linkage (alpha vs beta) was observed. In the CD spectra of peptides, β-sheets commonly display maxima at 198 and minima at 217 nm have been attributed to π–π* and n−π* transitions consistent with the aromatic interactions and hydrogen bonds of containing β-sheets.^17–19^ Here, PGPs synthesized from both Gal and Glc with β-linkages (*exo*-β-Gal, *endo*-β-Gal, *exo*-β-Glc, *endo*-β-Glc) showed narrower peaks at ∼ 217 nm and higher intensity peaks at ∼ 198 nm (Figure 3 **a-b**), indicating a highly ordered structure.^17,20^ Conversely, neither *exo*-α-Man nor *endo*-α-Man showed evidence of secondary structure by CD (Figure 3c). To probe this stereochemical dependency, we synthesized additional glycomonomers and subsequent PGPs (Scheme S8–S9) using α-linked sugars (*exo*-α-Gal and *exo*-α-Glc). As with their α-Man PGP counterparts, they also showed aqueous CD absorption spectra analogous to disordered, random-coil conformations observed for peptides (Figure 3d).^21^ In peptide science, CD is a well-established technique that provides a characteristic fingerprint for each secondary structure due to the intra- and interchain hydrogen bonding along the amino acid peptide backbone. However, in glycoscience, the use of this technique is more limited due to the absence of chromophores in many polysaccharides Therefore, while we observe clear evidence of secondary structure as a function of glycan epitope using CD, we cannot directly correlate our observations with established peptide literature. Nevertheless, to further investigate the origins of the CD signals observed in our β-linked glycopolymers, we synthesized control polymers bearing the same sugar epitopes but lacking the triazole linkage. These polymers did not exhibit any CD activity (Figure S2), suggesting that the emergence of secondary structure is dependent on the specific chemical configuration of the sugar-triazole motif. Although the origin of this stereochemical dependence remains unclear, these findings highlight the potential for specific glycan-linker configurations to drive ordered conformations in synthetic macromolecules.

**Figure 3:**
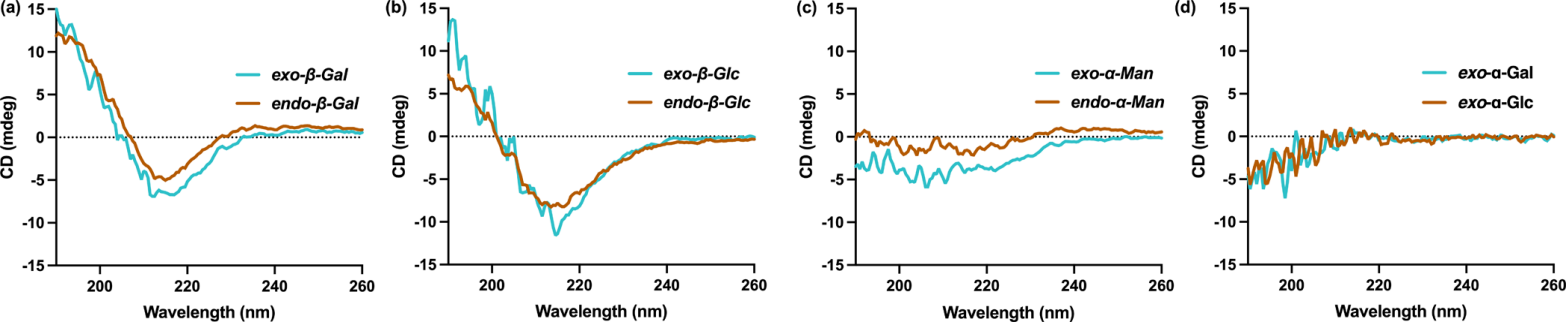
(a) Circular dichroism (CD) spectra of *exo*-β-Gal (blue) and *endo*-β-Gal (orange). (b) CD spectra of *exo*-β-Glc (blue) and *endo*-β-Glc (orange). (c) CD spectrum of *exo*-α-Man (blue) and *endo*-α-Man (orange). (d) CD spectra of *exo*-α-Gal (blue) and *exo*-α-Glc (orange).

We next utilized NOESY-NMR to determine whether through-space interactions were occurring within the pendant sugars of PGPs; here, spectral information may determine intramolecular associations. As a representative system, we evaluated the spectrum for *endo*-β-Gal PGPs. Several cross peaks were observed, particularly peaks at ∼ 5.16 ppm as well as ∼ 4.63 ppm and ∼ 4.55 ppm. These correspond to labeled sites 7, 10, and 9, respectively, on the NMR spectrum (Figure S3). These cross peaks indicate that through-space molecular associations are occurring within the PGPs. Moreover, the correlation between site 7, as well as 10 and 9, indicates increased intramolecular associations within the *endo*-β-Gal pendant groups, leading to the formation of strong intramolecular hydrogen bonding. A strong interaction between these sites may be an indication of a regular arrangement, which corroborates the formation of secondary structures as observed in CD analysis of PGPs.

### Evaluation of Stereochemistry-Driven Hydrophobicity

We next sought to understand whether differences in backbone stereochemistry led to differences in local hydrophobicity. To accomplish this, we evaluated the incorporation of Nile red, a hydrophobic guest molecule,^22^ into each PGP. In a typical experiment, a solution of Nile red in tetrahydrofuran (THF) was added to deionized water to achieve a targeted final Nile red concentration of 20 μg/mL. In the absence of polymer, Nile red precipitates into a red-pink color within an otherwise colorless or faintly pink aqueous phase (Figure S4a-c) upon evaporation of the THF cosolvent. This observation aligns with the reported negligible solubility of Nile red in water (<1 μg/mL).^22–24^ In contrast, the addition of Nile red to PGPs at 1.0 mg/mL resulted in pink solutions, with the degree of solubilization depending on both the backbone stereochemistry of the PGP and linkage between sugar and norbornene (Figure 4). All *endo*-PGPs exhibited lower fluorescence intensity as compared to *exo-*PGPs, indicating that *exo*-PGPs have higher local hydrophobicity. The amount of dye added was the same for all samples, and thus the observed increase in fluorescence may be attributed to the disruption of nonemissive dye aggregates, which Nile red can form both in aqueous solution and within the hydrophobic domains of polymer carriers.^25,26^ Nile red is renowned for its characteristic blue shift in emission maximum as the surrounding microenvironment becomes less polar. In our experiments, a clear shift in the UV–vis absorption from 650 nm to 642 nm was observed for all PGPs, indicating the presence of Nile red in a more hydrophobic environment. These findings are consistent with earlier studies by the Alexiev and Mohr groups,^26,27^ where a similar blue shift in fluorescence emission was linked to the incorporation of the dye within the hydrophobic domains of their respective systems. Together, these observations suggest that PGPs behave like a semiflexible chain in water and can capture hydrophobic molecules due to the presence of hydrophobic pockets (both the norbornene backbone and triazole ring), albeit to a greater extent by *exo*-PGPs.

**Figure 4:**
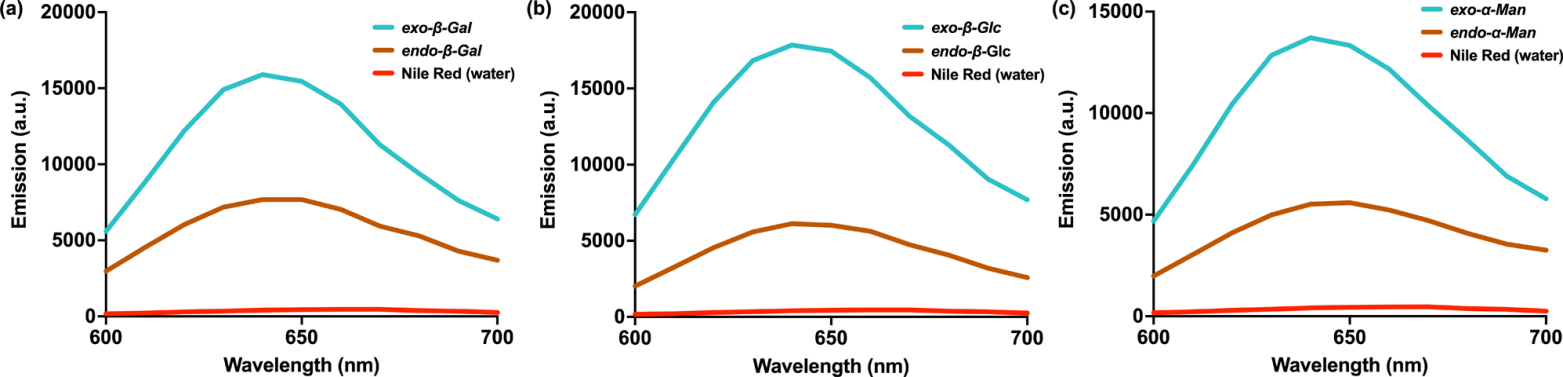
Nile red assay to probe the hydrophobicity of *exo* and *endo*-PGPs. (a) Fluorescence spectra of *exo*-β-Gal (blue), *endo*-β-Gal (orange), and Nile red in water (red). (b) Fluorescence spectra of *exo*-β-Glc (blue), *endo*-β-Glc (orange), and Nile red in water (red). (c) Fluorescence spectra of *exo*-α-Man (blue), *endo*-α-Man (orange), and Nile red in water (red).

### Molecular Dynamics Simulations of *exo*- and *endo*-PGPs

To probe the underlying mechanism that leads to differences in local hydrophobicity between these macromolecules, we employed all-atom (AA) molecular dynamics (MD) simulations. To closely replicate experimental conditions, molecular dynamics simulations were performed on 35-mer PGPs, matching the polymer chain length used in experiments. In the presence and absence of Nile red molecules, these simulations were conducted for both *exo*- and *endo*-β-Gal PGPs at 298 K. Further details on the MD simulation methods are provided in the Supporting Information (SI) (Figure S5). A visual inspection of the simulation trajectories revealed no apparent differences in the conformations of PGPs, regardless of their stereochemistry or the presence of Nile red (Movies M1–M4 show the dynamic evolution of the PGP systems during the simulations). Quantitative analysis based on the radius of gyration (*R*_g_) further confirmed that the conformational differences between *exo*- and *endo*-β-Gal PGPs were negligible. The last 100 ns of simulation data indicate that the backbone *R*_g_ for *endo*-β-Gal PGPs was 16.03 ± 0.35 Å, which slightly decreased to 15.21 ± 0.58 Å in the presence of Nile red. In contrast, the *exo*-β-Gal PGPs had a backbone *R*_g_ of 15.04 ± 0.65 Å, which increased to 16.29 ± 0.45 Å upon Nile red binding. The *R*_g_ of side chains was similar for all the systems (Table S2). Despite their similar *R*_g_ values, radial distribution function (RDF) analysis of backbone atoms, both with and without Nile red, revealed that *exo*-β-Gal PGPs exhibited a weaker structural correlation with water compared to *endo*-β-Gal PGPs (Figure S6–S9). This suggests that *exo*-β-Gal PGPs were relatively less hydrated than *endo*-β-Gal PGPs. Interestingly, RDF analysis of side chain atoms with water showed similar structural correlations for both *exo*- and *endo*-β-Gal PGPs. However, these side chains were generally more hydrated than backbone atoms, indicating that they were slightly more exposed to the solvent. Analysis of the MD simulations showed that Nile red molecules remained near the backbone and side chains of *exo*-β-Gal PGPs for a longer duration than *endo*-β-Gal PGPs (Figure S10, Table S3–S6). This can be attributed to the reduced hydration of *exo*-β-Gal PGPs, which may facilitate the absorption of hydrophobic Nile red molecules. The nonbonded interaction energies between Nile red and *exo*-β-Gal PGPs backbone and between Nile red and *exo*-β-Gal PGPs side chains were more favorable (more negative values) than those with *endo*-β-Gal PGPs (Figure 5, Figure S11, Table S7). This may explain the experimentally observed increase in Nile red adsorption on *exo*-PGPs.

**Figure 5:**
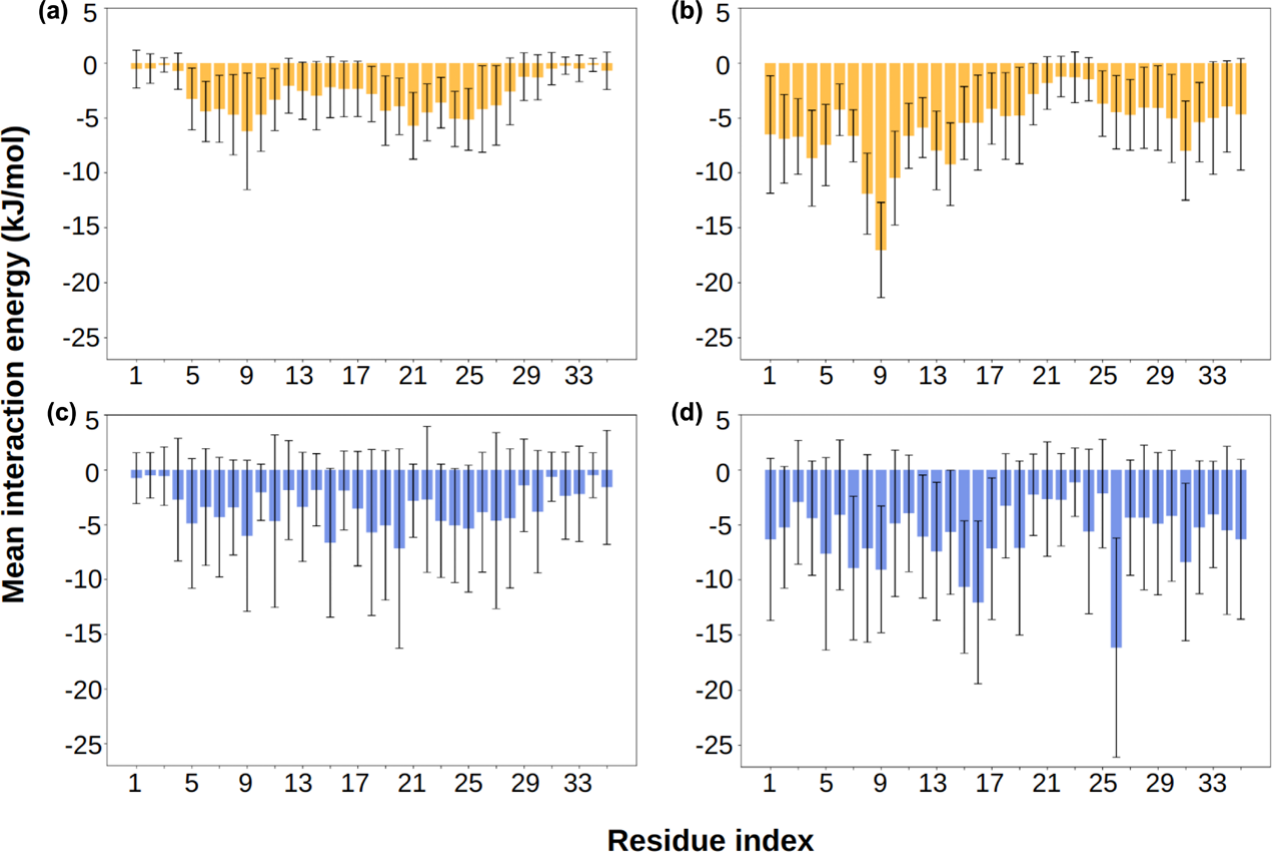
Nonbonded interaction energies between Nile red molecules and the backbones of (a) *endo*-PGPs, (b) *exo*-PGPs, and side chains of (c) *endo*-PGPs and (d) *exo*-PGPs. Error bars represent an average over 290 ns and three independent runs.

### Lectin Binding as a Function of Stereochemistry

To understand how the conformational differences between *exo*-PGPs and *endo*-PGPs impact their biological activity, we first assessed the binding activity of our polymers with both plant and mammalian lectins, using a dynamic light scattering (DLS) assay that has been previously employed for glycopolymer-lectin interactions.^28^ In general, lectin binding of macromolecules leads to the formation of clusters or aggregates, which can be readily measured over time using DLS. To evaluate the effect of backbone stereochemistry on PGP binding behavior, we investigated the interaction of galactose-based PGPs (*exo*-β-Gal and *endo*-β-Gal) with Peanut agglutinin (PNA), a well-studied plant lectin,^13,29,30^ as well as Galectin-3 (Gal-3), a mammalian lectin with significant implications in myriad biological processes, including cancer.^13,31,32^ Prior to the start of the assay, both *exo*-β-Gal PGPs and *endo*-β-Gal PGPs possessed a hydrodynamic diameter (*D*_h_) of ∼ 6 nm, while the *D*_h_ of PNA is 8.7 nm, respectively (Figure 6a, left side, Table S8). Upon addition of PNA to the solution of *exo*-β-Gal PGPs, a rapid change in size was observed by DLS (Figure 6a) indicating the formation of clusters and lectin binding.^33–35^ Subsequently, a gradual increase in size was observed over time, until reaching equilibrium at 10 min. This interaction was qualitatively verified by the formation of white precipitates upon lectin addition (Figure S12–S13). Conversely, when PNA was added to *endo*-β-Gal PGPs, no change in size, nor cluster formation was observed over the same incubation period (Figure 6b). To evaluate these differences more quantitatively, we employed microscale thermophoresis (MST) to evaluate the binding affinities between both *exo*-β-Gal PGPs and *endo*-β-Gal PGPs with PNA. Consistent with our qualitative DLS assay, *exo*-β-Gal PGPs associated significantly more strongly than *endo*-β-Gal PGPs with PNA (Table S9). The observed K_d_ value for *exo*-β-Gal PGPs binding to PNA was determined to be 1.54 μM, which is ca. 1000-fold higher than *endo*-β-Gal PGPs (K_d_ ∼ 1.5 mM).

**Figure 6:**
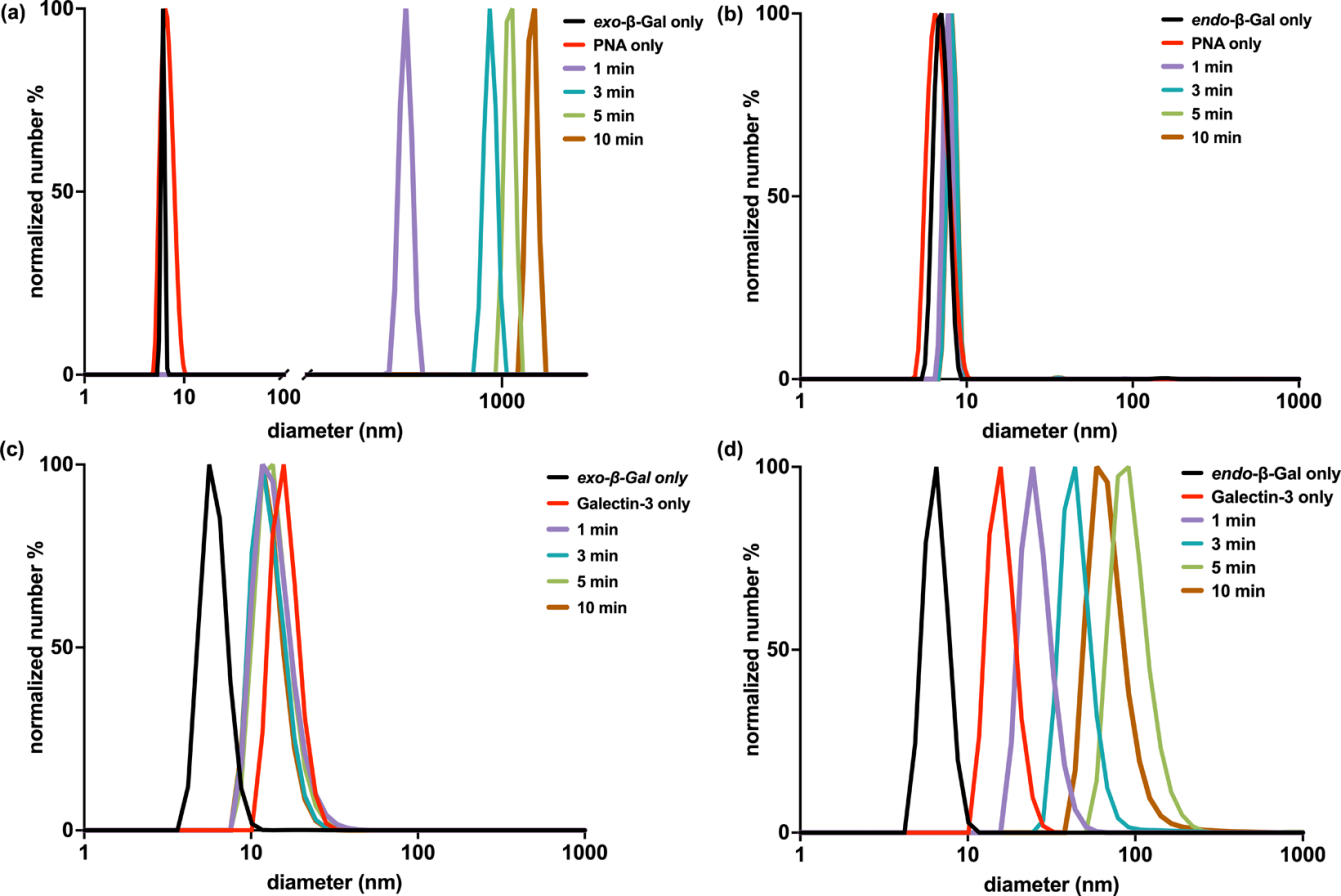
Protein binding, as evaluated by DLS. (a) Hydrodynamic diameter of *exo*-β-Gal PGPs prior to the addition of PNA (black) or following 1 min (purple), 3 min (blue), 5 min (green) and 10 min (orange) of incubation with the lectin. PNA without polymer is shown in red. (b) Hydrodynamic diameter of *endo*-β-Gal PGPs prior to the addition of (black) or following 1 min (purple), 3 min (blue), 5 min (green) and 10 min (orange) of incubation with the lectin. PNA without polymer is shown in red. (c) Hydrodynamic diameter of *exo*-β-Gal PGPs prior to the addition of PNA (black) or following 1 min (purple), 3 min (blue), 5 min (green) and 10 min (orange) of incubation with the lectin. Galectin-3 without polymer is shown in red. (d) Hydrodynamic diameter of *endo*-β-Gal PGPs prior to the addition of (black) or following 1 min (purple), 3 min (blue), 5 min (green) and 10 min (orange) of incubation with the lectin. Galectin-3 without polymer is shown in red).

Surprisingly, the trends observed for plant lectins were exactly opposite from those observed when analyzing the binding between PGPs and Gal-3. Incubation of *exo*-β-Gal PGPs with the lectin resulted in no obvious increase in size over the course of 10 min (Figure 6c), whereas incubating Gal-3 with *endo*-β-Gal PGPs induced a rapid increase in hydrodynamic diameter (Figure 6d), indicating a distinct difference in the rate of lectin binding and overall binding behavior. This may be due to the size of the binding pockets for mammalian lectins as compared to those of plants, as well as the structural complexity of Gal-3 as compared to PNA. Gal-3 is also significantly larger (hydrodynamic diameter ∼ 20 nm vs ∼ 9 nm for PNA), which may further contribute to differences in how these proteins engage with glycopolymers. These observations highlight the importance of carefully selecting relevant protein targets when evaluating glycopolymer-lectin interactions.

### Influence of Stereochemistry on Mammalian Cellular Uptake

Given our initial results, wherein *endo*-PGPs bound mammalian lectins better than their *exo*- counterparts, we evaluated whether this trend extended to differences in their biological behavior in mammalian cells. Toward this end, we synthesized six PGPs that were end-labeled (Scheme S10) with a near-infrared fluorophore (*endo-*β-Gal, *exo*-β-Gal, *endo*-β-Glc, *exo*-β-Glc, *endo*-α-Man, and *exo*-α-Man) and incubated each with 4T1 triple negative breast cancer cells for 24 h. Within each glycan series, *endo*- and *exo*-PGPs exhibited comparable cytotoxicity profiles, with no significant differences observed at any tested concentration (Figure S14). Additionally, no differences in surface charge were observed between *endo*- and *exo*-PGPs bearing the same glycan epitope (Figure S15).We then quantified cellular uptake using flow cytometry (Figure 7a, Table S10). Importantly, all *endo*-PGPs showed significantly higher cellular uptake than their *exo*- counterparts, which matched our observations of *in vitro* mammalian lectin binding. To probe potential mechanisms for why this may occur, we incubated the polymers with cells at 4 °C for 30 min to limit energy-dependent cell uptake.^36,37^ However, this reduced the uptake of both *endo*- and *exo*-PGPs (Table S11), indicating that both polymers are being taken up by energy-dependent mechanisms, and that the privileged entry by *endo*-PGPs are not due to simple conformational differences that enable passive cellular entry.

**Figure 7:**
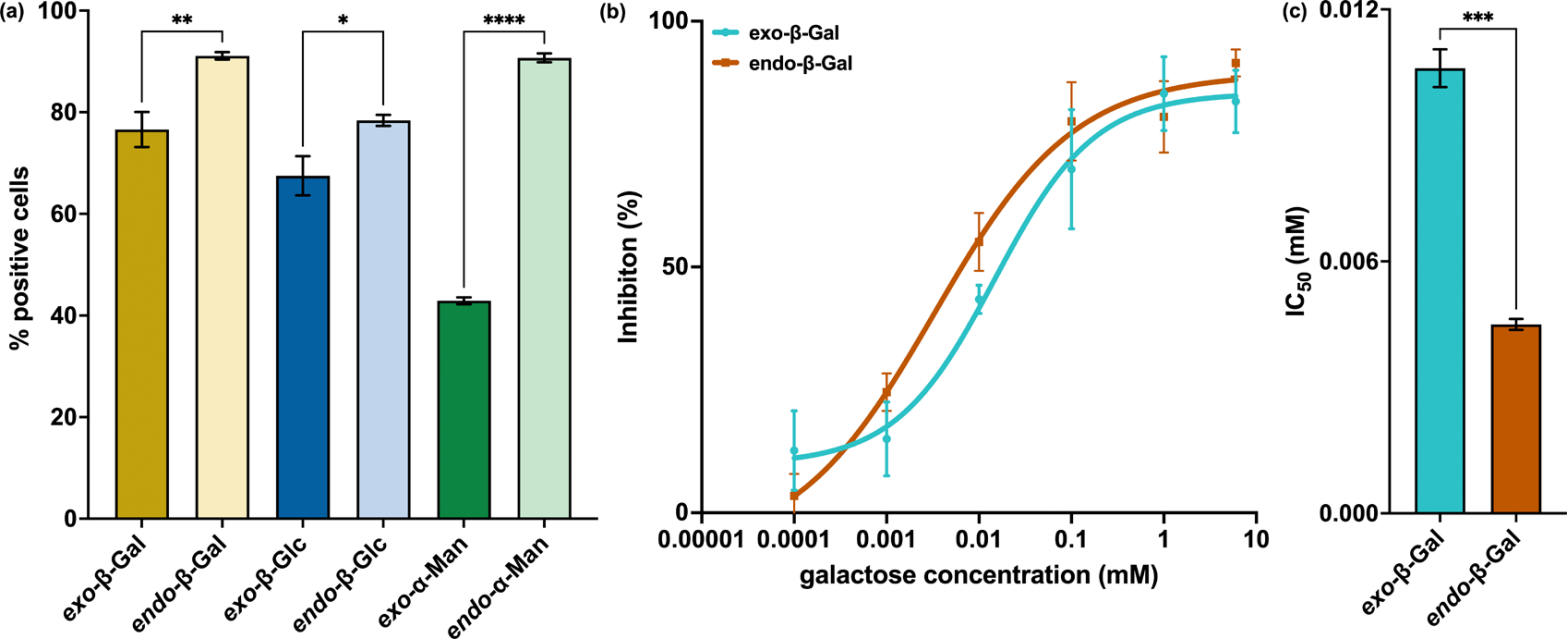
Biological behavior of PGPs. (a) Cell internalization of all PGPs by 4T1 cells, as measured by flow cytometry. (b) Inhibition of Ctx binding to GM1 ganglioside by *exo*-β-Gal PGPs (blue) and *endo*-β-Gal PGPs (orange) as a function of concentration with respect to galactose. Statistical analysis was performed using an ordinary one-way ANOVA, where “*” represents a *P* value of <0.05, “**” represents a *P* value of <0.01, “***” represents a *P* value of <0.001, and “****” represents a P value of <0.0001.

### Influence of Stereochemistry on Cholera Toxin Inhibition

Finally, we hypothesized that the stereochemistry of *exo*- and *endo*-PGPs would extend to their interactions with clinically relevant proteins. Inspired by the work of Kiessling and co-workers,^9^ which showed a stereochemical dependence on the binding of Ctx, a virulence factor produced by the bacterium *Vibrio cholerae*, which plays a crucial role in promoting infection in the host upon binding to the GM1 ganglioside receptor.^38^ We incubated fluorescein-labeled Ctx with increasing concentrations of either *exo*-β-Gal PGPs or *endo*-β-Gal PGPs (Table S12). We then added these solutions to individual wells of a 96-well plate coated with GM1 ganglioside and measured the fluorescence intensity as a function of concentration (Figure 7b). From these analyses, we determined the IC_50_ values of each polymer (Figure 7c), where *endo*-β-Gal PGPs exhibited IC_50_ values three times lower than that of *exo*-β-Gal PGPs (*P* < 0.001), indicating that *endo*-PGPs have higher affinity for bacterial lectins.

## Conclusion

This work shows that stereochemistry plays a central role in shaping the behavior of synthetic glycopolymers. By varying glycosidic linkage and backbone configuration, while keeping monomer composition constant, we observed clear and reproducible differences in polymer conformation, local chemical environment, and biological activity. *Endo*- and *exo*-derived polymers behaved differently across every analysis, from lectin binding to cellular uptake, despite having matched sugar content. Similarly, α- versus β-linkages determined whether the polymers exhibited signatures of secondary structure in solution.

These findings highlight that glycopolymer function is not just a product of sugar identity or density, but also how sugar epitopes are arranged at the molecular level. Small changes in stereochemistry, whether in the glycan or the polymer backbone, translate to measurable differences in how these materials interact with proteins, toxins, and cells. This level of sensitivity suggests that stereochemistry should not be treated as an afterthought in glycopolymer design, but rather as an active and tunable design element.

More broadly, this work provides a framework for how precision polymer chemistry can be used to dissect structure–function relationships in biologically relevant systems. The materials herein offer a controlled platform for isolating the impact of stereochemistry in ways that are not possible with larger, more heterogeneous systems. As interest continues to grow in using synthetic polymers to modulate biological interactions, these results point to new opportunities to program function not just through composition or sequence, but through the spatial arrangement of atoms in three dimensions. Ultimately, this level of stereochemical control will be important for developing glycomaterials that exhibit not just binding, but defined and selective biological activity that is informed by a clearer understanding of how molecular structure governs function.

## Supplementary Material











## Data Availability

Github link for simulation code: https://github.com/Deshmukh-Group/Stereochemistry-drives-macromolecular-conformations

## Notes

*exo*-β-Galactose-PGP. backbone, sidechain shown in orange, blue, respectively (MP4)
*endo*-β-Galactose-PGP with Nile red. backbone, sidechain, Nilered shown in orange, blue, green, respectively (MP4)
*endo*-β-Galactose-PGP. backbone, sidechain shown in orange, blue, respectively (MP4)
*exo*-β-Galactose-PGP with Nile red. backbone, sidechain, Nilered shown in orange, blue, green, respectively (MP4)
Synthetic schemes, experimental details, auxiliary tables, molecular dynamics simulations procedures, NMR spectra, CD spectra, and biological assay data (PDF)

## References

[1] Stank A., Kokh D. B., Fuller J. C., Wade R. C. (2016). Protein Binding
Pocket Dynamics. Acc. Chem. Res..

[2] Atanasov A. G., Zotchev S. B., Dirsch V. M., Orhan I. E., Banach M., Rollinger J. M., Barreca D., Weckwerth W., Bauer R., Bayer E. A. (2021). Natural products in
drug discovery: advances and opportunities. Nat. Rev. Drug Discovery.

[3] McConathy J., Owens M. J. (2003). Stereochemistry in Drug Action. Prim Care Companion J. Clin Psychiatry.

[4] McVicker R. U., O’Boyle N. M. (2024). Chirality
of New Drug Approvals (2013–2022):
Trends and Perspectives. J. Med. Chem..

[5] Miura Y. (2012). Design and
synthesis of well-defined glycopolymers for the control of biological
functionalities. Polym. J..

[6] Richards S.-J., Gibson M. I. (2021). Toward Glycomaterials
with Selectivity as Well as Affinity. JACS Au.

[7] Yilmaz G., Becer C. R. (2013). Precision glycopolymers
and their interactions with
lectins. Eur. Polym. J..

[8] Hartweg M., Jiang Y., Yilmaz G., Jarvis C. M., Nguyen H. V. T., Primo G. A., Monaco A., Beyer V. P., Chen K. K., Mohapatra S. (2021). Synthetic Glycomacromolecules of Defined Valency,
Absolute Configuration, and Topology Distinguish between Human Lectins. JACS Au.

[9] Kruger A. G., Brucks S. D., Yan T., Cárcarmo-Oyarce G., Wei Y., Wen D. H., Carvalho D. R., Hore M. J. A., Ribbeck K., Schrock R. R., Kiessling L. L. (2021). Stereochemical Control Yields Mucin
Mimetic Polymers. ACS Central Science.

[10] Nguyen H. V. T., Jiang Y., Mohapatra S., Wang W., Barnes J. C., Oldenhuis N. J., Chen K. K., Axelrod S., Huang Z., Chen Q. (2022). Bottlebrush polymers with flexible enantiomeric side
chains display differential biological properties. Nat. Chem..

[11] Jeon S., Haynie T., Chung S., Callmann C. E. (2024). Bioinspired,
Carbohydrate-Containing
Polymers Efficiently and Reversibly Sequester Heavy Metals. ACS Central Science.

[12] Jeon S., Odom T. L., Williams C. A., Callmann C. E. (2025). Glycopolymer-Mediated
Selective Separation of Middle Rare Earth Elements. Angew. Chem., Int. Ed..

[13] Williams C. A., Stone D. J., Joshi S. Y., Yilmaz G., Farzeen P., Jeon S., Harris-Ryden Z., Becer C. R., Deshmukh S. A., Callmann C. E. (2024). Systematic Evaluation of Macromolecular Carbohydrate-Lectin
Recognition Using Precision Glycopolymers. Biomacromolecules.

[14] Blosch S. E., Scannelli S. J., Alaboalirat M., Matson J. B. (2022). Complex Polymer
Architectures Using Ring-Opening Metathesis Polymerization: Synthesis,
Applications, and Practical Considerations. Macromolecules.

[15] Scannelli S. J., Paripati A., Weaver J. R., Vu C., Alaboalirat M., Troya D., Matson J. B. (2023). Influence of the
Norbornene Anchor
Group in Ru-Mediated Ring-Opening Metathesis Polymerization: Synthesis
of Linear Polymers. Macromolecules.

[16] Slugovc C. (2004). The Ring Opening
Metathesis Polymerisation Toolbox. Macromol.
Rapid Commun..

[17] Cheng R. P., Gellman S. H., DeGrado W. F. (2001). β-Peptides: From Structure
to Function. Chem. Rev..

[18] Schmitt M. A., Choi S. H., Guzei I. A., Gellman S. H. (2006). New Helical Foldamers:
Heterogeneous Backbones with 1:2 and 2:1 α:β-Amino Acid
Residue Patterns. J. Am. Chem. Soc..

[19] Seebach D., Ciceri P. E., Overhand M., Jaun B., Rigo D., Oberer L., Hommel U., Amstutz R., Widmer H. (1996). Probing the
Helical Secondary Structure of Short-Chain β-Peptides. Helv. Chim. Acta.

[20] Mándity I. M., Fülöp L., Vass E., Tóth G. K., Martinek T. A., Fülöp F. (2010). Building β-Peptide H10/12 Foldamer
Helices with Six-Membered Cyclic Side-Chains: Fine-Tuning of Folding
and Self-Assembly. Org. Lett..

[21] Becker J., Terracciano R., Yilmaz G., Napier R., Becer C. R. (2023). Step-Growth
Glycopolymers with a Defined Tacticity for Selective Carbohydrate–Lectin
Recognition. Biomacromolecules.

[22] Madeira
do O J., Foralosso R., Yilmaz G., Mastrotto F., King P. J. S., Xerri R. M., He Y., van der
Walle C. F., Fernandez-Trillo F., Laughton C. A. (2019). Poly­(triazolyl
methacrylate) glycopolymers as potential targeted unimolecular nanocarriers. Nanoscale.

[23] Jose J., Burgess K. (2006). Syntheses and Properties of Water-Soluble Nile Red
Derivatives. Journal of Organic Chemistry.

[24] Greenspan P., Fowler S. D. (1985). Spectrofluorometric
studies of the lipid probe, nile
red. J. Lipid Res..

[25] Eisfeld A., Briggs J. S. (2006). The J- and H-bands
of organic dye aggregates. Chem. Phys..

[26] Kurniasih I. N., Liang H., Mohr P. C., Khot G., Rabe J. P., Mohr A. (2015). Nile Red Dye in Aqueous
Surfactant and Micellar Solution. Langmuir.

[27] Boreham A., Pfaff M., Fleige E., Haag R., Alexiev U. (2014). Nanodynamics
of Dendritic Core–Multishell Nanocarriers. Langmuir.

[28] Wang X., Ramström O., Yan M. (2011). Dynamic light scattering as an efficient
tool to study glyconanoparticle–lectin interactions. Analyst.

[29] Rudra S., Mondal S., Chakraborty M., Swamy M. J., Jana T. (2024). Galactose
Glycopolymer-Grafted Silica Nanoparticles: Synthesis and Binding Studies
with Lectin. ACS Applied Bio Materials.

[30] Ambrosi M., Cameron N. R., Davis B. G., Stolnik S. (2005). Investigation of the
interaction between peanut agglutinin and synthetic glycopolymeric
multivalent ligands. Organic & Biomolecular
Chemistry.

[31] Liu F.-T., Stowell S. R. (2023). The role of galectins
in immunity and infection. Nature Reviews Immunology.

[32] Joeh E., O’Leary T., Li W., Hawkins R., Hung J. R., Parker C. G., Huang M. L. (2020). Mapping
glycan-mediated galectin-3
interactions by live cell proximity labeling. Proc. Natl. Acad. Sci. U. S. A..

[33] Kanai M., Mortell K. H., Kiessling L. L. (1997). Varying
the Size of Multivalent Ligands:
The Dependence of Concanavalin A Binding on Neoglycopolymer Length. J. Am. Chem. Soc..

[34] Cairo C. W., Gestwicki J. E., Kanai M., Kiessling L. L. (2002). Control
of Multivalent Interactions by Binding Epitope Density. J. Am. Chem. Soc..

[35] Kiessling L. L., Grim J. C. (2013). Glycopolymer probes of signal transduction. Chem. Soc. Rev..

[36] Callmann C. E., Cole L. E., Kusmierz C. D., Huang Z., Horiuchi D., Mirkin C. A. (2020). Tumor cell lysate-loaded immunostimulatory spherical
nucleic acids as therapeutics for triple-negative breast cancer. Proc. Natl. Acad. Sci. U. S. A..

[37] Liu L., Bai X., Martikainen M.-V., Kårlund A., Roponen M., Xu W., Hu G., Tasciotti E., Lehto V.-P. (2021). Cell membrane coating integrity affects
the internalization
mechanism of biomimetic nanoparticles. Nat.
Commun..

[38] Turnbull W. B., Precious B. L., Homans S. W. (2004). Dissecting
the Cholera Toxin–Ganglioside
GM1 Interaction by Isothermal Titration Calorimetry. J. Am. Chem. Soc..

