# Supplementary material for: Stereochemistry Drives the Macromolecular Conformation and Biological Activity of Glycopolymers
Source: ACS Cent Sci. 2025 Aug 6;11(9):1573–80. doi: 10.1021/acscentsci.5c00768 (PMC12464773; doi:10.1021/acscentsci.5c00768)
Supplement: Supplementary file 5 [file oc5c00768_si_005.pdf]

Supporting Information

for

Stereochemistry Drives the Macromolecular Conformation and Biological Activity of Glycopolymers

Muhammad Waqas Ishaq[1], Parisa Farzeen[2], Lindsay Vaughn[1], Daniel J. Stone[1], Sanket A. Deshmukh[2], and Cassandra E. Callmann[1,*]

[1]Department of Chemistry, University of Texas at Austin, Austin Texas 78712, USA.

[2]Department of Chemical Engineering, Virginia Tech, Blacksburg, VA 24061, USA.

*Email: ccallmann@utexas.edu

Material and Reagents: All reagents were of the highest commercial quality and used as received without further purification. Anhydrous dichloromethane (DCM) and N,N-dimethylformamide (DMF) were obtained from distillation of HPLC grade dichloromethane and dimethylformamide respectively. Cis-5-norbornene-*exo*-2,3-dicarboxylic anhydride (Purity 98%) was obtained from Oakwood Chemical. Triethylamine (TEA), anhydrous pyridine, potassium carbonate, Maxisorb plates, syringe filters (0.45 µm), and cis-5-norbornene-*endo*-2,3-dicarboxylic anhydride (Purity 97%) were obtained from Thermo Fisher Scientific. Sodium hydroxide, calcium chloride, manganese chloride and potassium chloride were obtained from Beantown Chemical. Galectin-3 was sources from Acros. 4T1 cells were obtained from American Type Culture Collection (ATCC) and were obtained at 37°C in 5% CO_2. Silica flash column chromatography was performed using silica gel (40−63 µm), which was supplied from Sorbtech. Aqueous solutions were freshly prepared with ultra-pure deionized water from a water purification system. Dialysis was performed with Snakeskin dialysis tubing, 3.5K MWCO.Glucose, galactose, mannose, anhydrous toluene, anhydrous tetrahydrofuran (THF), NH_2-PEG_2-OH, sodium methoxide, sodium hydride 60% dispersion in mineral oil, trimethylsilyl azide, 1M tetrabutylammonium fluoride (TBAF) trihydrate, N,N,N,N”,N”-pentamethyldiethylenetriamine (PMDETA), tin (IV) chloride, glacial acetic acid, anhydrous methanol, HBr in acetic acid, deuterated dimethylformamide (d_6-DMF), Grubbs 2nd generation (M204), propargyl bromide, acetic anhydride, ethyl vinyl ether (EVE), sodium azide, aluminum trichloride, copper bromide, amicon ultra – 4 centrifugal filters ultracel - 3K, peanut agglutinin (PNA), Phosphate buffer saline (PBS), trypsin-EDTA, Roswell Park Memorial Institute 1640 (RPMI-1640), paraformaldehyde (PFA), fetal bovine serum (FBS), penicillin-streptomycin (pen-strep), and all chemicals not mentioned were obtained from Sigma-Aldrich.

General Procedures

Synthetic manipulations that required an inert atmosphere (where noted) were carried out under nitrogen using standard Schlenk techniques. NMR (^{1}H, ^{13}C, NOESY) spectra were recorded on Bruker Prodigy 500 MHz, Varian 400 MHz and Bruker Advance Neo 400 MHz spectrometer. The 1H, and 13C chemical shifts were reported as δ in units of parts per million (ppm), referenced to the residual solvent. Splitting patterns are denoted as s (singlet), d (doublet), t (triplet), q (quartet), m (multiplet), and br (broad). High-resolution electrospray ionization (ESI) mass spectra were obtained at the mass spectrometry facility (the University of Texas at Austin). Polymer particle size was measured by Dynamic Light Scattering Zetasizer Nano ZS. GPC data was measured by using TOSOH EcoSEC Elite HLC-8420GPC.

Dynamic Light Scattering (DLS) Analysis: Each polymer was dissolved in 18.2 MΩ water and placed on a rocker for at least 12 hours. The sample was then transferred to a disposable plastic cuvette and was analyzed using a Zetasizer Nano ZS (Dispersant: H_2O RI = 1.330, Viscosity = 0.8872; Material: RI = 1.45, Absorption = 0.001; T = 25 °C) with five runs per measurement, 10 s per run, and at least 3 measurements averaged together. Zeta potential was measured in deionized (DI) water by Dynamic Light Scattering on a Zetasizer Nano ZS.

Circular dichroism (CD): CD measurements were performed on a Jasco J-1500 spectropolarimeter where the sensitivity, time constant, and scan rate were chosen appropriately. The temperature was kept constant at 293 K. Cells with an optical path length

of 1 cm were used. Solutions were prepared in deionized water at a set concentration of 30 μM. Data acquisition and analysis were performed with JASCO Spectra Manager and OriginPro 9, respectively.

Nile Red Assay: To perform the Nile Red assay, glycopolymer (*exo*-β-Gal, *exo*-β-Glc, *exo*-α-Man, *endo*-β-Gal, *endo*-β-Glc, and *endo*-α-Man, 50-60 μM) stock solutions were prepared in DI water To a fixed volume (323.4 μL) of each stock solution, Nile Red (20 μg/mL) dissolved in THF was added.. The solutions were left open under an aspirated fume-hood protected from light for 5 hours, to allow the THF to evaporate. Each solution(100 μL) was transferred into a Corning 96-black well plate and analyzed using a Biotek Synergy H1 microplate reader. Fluorescence was read from 600 to 700 nm, with λ_{ex}=550 nm. Samples were analyzed in triplicate (n=3).

Microscale Thermophoresis (MST): To generate fluorophore-tagged peanut agglutinin (PNA) for MST analysis, NHS-fluorescein (2.1 mM) dissolved in PBS buffer (pH 8.0) was added to PNA (~5 nM) and incubated for 24 hours at room temperature. The solution was filtered using a 3 kDa Amicon spin filter to remove unreacted NHS-fluorescein and the filtrate was dialyzed (x8) into Milli-Q water and stored at 4ºC prior to use. For the MST assay itself, each glycopolymer (*exo*-β-Gal, *exo*-β-Glc, *exo*-α-Man, *endo*-β-Gal, *endo*-β-Glc, and *endo*-α-Man, 5 mM – 0.3 M) was dissolved in 1x PBS and added to NHS-fluorescein (20 nM) to create a 1:1 (v:v, glycopolymer:PNA-fluorescein) solution. The 1:1 solution was incubated for 1.5-2 hours at room temperature in the absence of light and each MST experiment was run in triplicate.

The data was fit using the Nanotemper K_d model with data originating from 5 or 10 seconds post-laser ignition. The resulting K_d values were then averaged with standard deviation determined. MST data was taken at 25 °C by using 9% Nanoblue (ex. 465-490 nm, em. 500-550 nm).

Cell Viability Assays: 4T1 cells were seeded (2,500 cells/well) onto a 96-well plate in RPMI-1640 media (10% FBS, 1% pen-strep) and grown overnight at 37 °C. The cells were treated with the glocyopolymer (*exo*-β-Gal, *exo*-β-Glc, *exo*-α-Man, *endo*-β-Gal, *endo*-β-Glc, and *endo*-α-Man, 5-0.5 μM) or PBS for 24 hours at 37 °C. A commercially available CCK-8 solution (10 μL) was added to each well and incubated for 4 hours at 37 °C. The absorbance at 450 nm of each sample was recorded on a BioTek Synergy H1 microplate reader.

Flow Cytometry: 4T1 cells were seeded (500,000 cells / well) onto a 6 well plate with RPMI-1640 media (10% FBS, 1% pen-strep) and grown overnight at 37 °C . Cells were then incubated with either glycopolymer (*exo*-β-Gal, *exo*-β-Glc, *exo*-α-Man, *endo*-β-Gal, *endo*-β-Glc, and *endo*-α-Man, 5 μM) or PBS for 4 hours at 37 °C. Following incubation, the cells were washed with PBS (x3), detached with trypsin-EDTA (0.05%), and pelleted at 1000xg for 5 minutes. The pellets were fixed with 4% paraformaldehyde (PFA) for 20 minutes at room temperature. The cells were pelleted at 1000xg for 5 minutes, and the pellet was resuspended in 1 mL of PBS. Flow cytometry measurements were then collected on a (BD LSR Fortessa).

Material and Methods

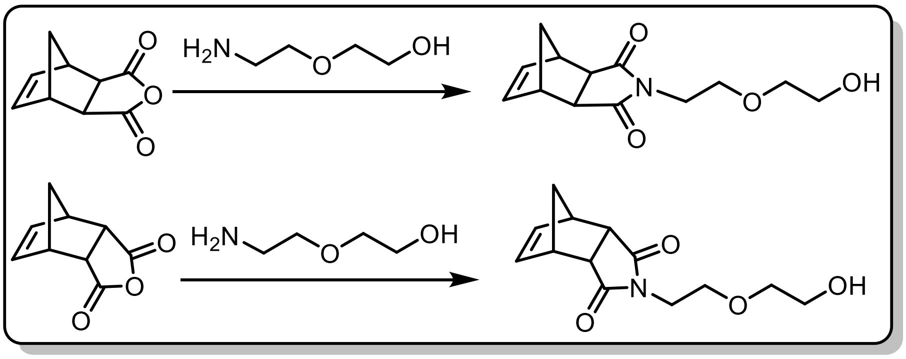

Scheme S1: Synthesis of norbornene (*exo*- and *endo*) with polyethylene glycol (PEG_2) linker named as Exo-OH and Endo-OH, respectively.

Synthesis of PEG linked monomers.

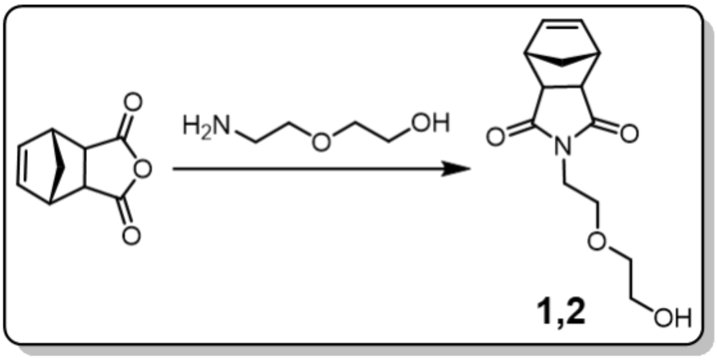

Synthesis of 1 and 2: To a stirred solution of either Exo- or Endo-Norbornene dicarboxylic acid (5 g, 30.34 mmol) in dry toluene (10 mL), was added NH_2-PEG_2-OH (3.02 mL, 30.34 mmol) and TEA (4.23 mL, 30.34 mmol) sequentially. The reaction was refluxed under a nitrogen atmosphere overnight and then concentrated under reduced pressure using a rotary evaporator. The crude product was extracted with ethyl acetate (EtOAc, 200 mL) and the organic layer was washed with water (300 mL), a saturated solution of sodium bicarbonate ($NaHCO_3$, 300 mL), and brine (300 mL), sequentially. The organic phase was dried over sodium sulfate (Na_2SO_4) and concentrated under reduced pressure to obtain a clear viscous oil. The crude product was purified by column chromatography (SiO_2, DCM/Acetone 60:40) to afford **1** (Exo-OH, 91%) or **2** (Endo-OH, 85%) as a white powder.

Exo-OH (**1**) NMR and HRMS Analysis:

^{1}H NMR (400 MHz, $CDCl_3$) δ 6.29 (t, *J* = 1.9 Hz, 1H), 3.71 – 3.55 (m, 4H), 3.31 – 3.21 (m, 1H), 2.70 (d, *J* = 1.4 Hz, 1H), 1.50 (dt, *J* = 9.9, 1.7 Hz, 1H), 1.34 (d, *J* = 9.9 Hz, 1H).
^{13}C NMR (100 MHz, $CDCl_3$) δ: 178.15, 137.81, 72.03, 67.27, 61.79, 47.90, 45.08, 42.44, 37.91.
HRMS for $C_{13}H_{17}NO_4$: $[M + Na]^+$ calc. = 274.1050; found = 274.1045.

Endo-OH (**2**) NMR and HRMS Analysis:

^{1}H NMR (400 MHz, $CDCl_3$) δ 6.08 (q, *J* = 1.7 Hz, 2H), 3.65 (t, *J* = 4.5 Hz, 2H), 3.52 – 3.43 (m, 4H), 3.37 (dp, *J* = 3.3, 1.6 Hz, 2H), 3.26 (dt, *J* = 2.7, 1.3 Hz, 2H), 1.72 (dt, *J* = 8.8, 1.6 Hz, 1H), 1.52 (d, *J* = 8.4 Hz, 1H).
^{13}C NMR (100 MHz, $CDCl_3$) δ: 177.94, 134.32, 72.08, 67.78, 61.66, 52.12, 45.76, 44.89, 37.81.
HRMS for $C_{13}H_{17}NO_4$: $[M + Na]^+$ calc. = 274.1050; found = 274.1046.

Propargylation of PEG linked monomers.

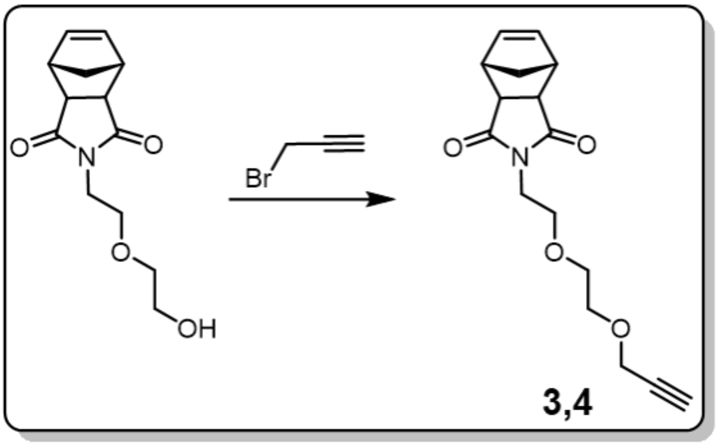

3,4

Synthesis of 3 and 4: A stirred solution of **1** or **2** (3 g, 11.90 mmol) in dry DCM (30 mL) was cooled to -78 °C. Under a nitrogen atmosphere, sodium hydride (NaH dispersed in 60% mineral oil, 0.57 g, 14.28 mmol) and propargyl bromide (1.08 mL, 14.28 mmol) were added sequentially. The reaction mixture was stirred at room temperature overnight, then redissolved in DCM (100 mL) and washed with water (100 mL x3), a), saturated solution of $NaHCO_3$ (100 mL), and brine (100 mL) and dried over Na_2SO_4. Rotary evaporation afforded a clear viscous oil, which was purified by column chromatography (SiO_2, EtOAc/Hexanes, 50:50) to afford **3** (Exo-Alk, 78% yield) as a whitish powder or **4** (Endo-Alk, 69%) as a clear liquid.

Exo-Alk (**3**) NMR and HRMS Analysis:

^{1}H NMR (400 MHz, $CDCl_3$) δ 6.26 (d, *J* = 1.9 Hz, 2H), 4.13 (d, *J* = 2.4 Hz, 2H), 3.75 – 3.61 (m, 4H), 3.59 (s, 5H), 3.24 (s, 2H), 2.66 (s, 2H), 2.40 (t, *J* = 2.4 Hz, 1H), 1.53 – 1.27 (m, 2H).
^{13}C NMR (100 MHz, $CDCl_3$) δ: 178.00, 137.84, 79.62, 77.07, 74.50, 69.67, 69.03, 66.88, 58.38, 47.82, 45.28, 42.74, 37.69
HRMS for $C_{16}H_{19}NO_4$: $[M + Na]^+$ calc. = 312.1206; found = 312.1210.

Endo-Alk (**4**) NMR and HRMS Analysis:

^{1}H NMR (400 MHz, $CDCl_3$) δ 6.10 (d, *J* = 2.0 Hz, 2H), 4.17 (d, *J* = 2.4 Hz, 2H), 3.76 – 3.43 (m, 8H), 3.44 – 3.32 (m, 2H), 3.25 (s, 2H), 2.42 (t, *J* = 2.4 Hz, 1H), 1.72 (dd, *J* = 8.7, 1.8 Hz, 1H), 1.51 (s, 1H). ^{13}C NMR (100 MHz, $CDCl_3$) δ: 177.94, 134.32, 72.08, 67.78, 61.66, 52.12, 45.76, 44.89, 37.81.
HRMS for $C_{16}H_{19}NO_4$: $[M + Na]^+$ calc. = 312.1206; found = 312.1203.

Installation of Protecting Groups on Monosaccharides (Gal, Glc, Man).

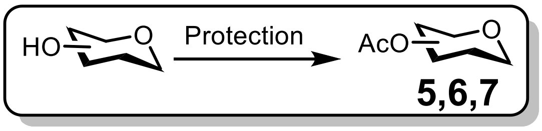

Synthesis of 5,6, and 7: A stirred solution of D-monosaccharide (10 g, 55.5 mmol) in pyridine (100 mL) was cooled to 0 °C. Dry acetic anhydride (141.7 g, 1.39 mol) was added slowly over the period of 1 h and the reaction mixture was stirred at room temperature overnight. The reaction mixture was concentrated under reduced pressure. The reaction mixture was extracted in DCM (200 mL) and the organic layer was washed sequentially with HCl (1M, 100 mL x3), a saturated solution of $NaHCO_3$ (75 mL x3), water (75 mL), and brine (75 mL). The organic layer was dried over Na_2SO_4 and concentrated under reduced pressure to obtain a clear viscous oil. The crude product was purified by column chromatography (SiO_2, EtOAc/Hexanes 1:1) afford **5** (Mannose, 85%), **6** (Glucose, 92%), or **7** (Galactose, 95%) as a clear liquid.

^{1}H NMR (400 MHz, cdcl$_3$) δ 6.05 (d, *J* = 1.9 Hz, 1H), 5.83 (d, *J* = 1.2 Hz, 0H), 5.45 (dd, *J* = 3.3, 1.1 Hz, 0H), 5.37 – 5.20 (m, 4H), 5.10 (dd, *J* = 10.0, 3.3 Hz, 0H), 4.26 (ddd, *J* = 12.4, 10.1, 5.1 Hz, 2H), 4.15 – 3.97 (m, 3H), 2.26 – 1.88 (m, 24H). Note: mixture of α/β.

Glc-OAc$_5$ (**6**)

^{1}H NMR (400 MHz, cdcl$_3$) δ 5.68 (d, *J* = 8.3 Hz, 1H), 5.22 (t, *J* = 9.4 Hz, 1H), 5.15 – 5.05 (m, 2H), 4.26 (dd, *J* = 12.5, 4.5 Hz, 1H), 4.08 (dd, *J* = 12.5, 2.2 Hz, 1H), 3.81 (ddd, *J* = 10.0, 4.5, 2.2 Hz, 1H), 2.14 – 1.91 (m, 15H).

Gal-OAc$_5$ (**7**)

^{1}H NMR (400 MHz, cdcl$_3$) δ 6.36 (d, *J* = 2.0 Hz, 1H), 5.48 (t, *J* = 1.5 Hz, 1H), 5.34 – 5.29 (m, 2H), 4.36 – 4.28 (m, 1H), 4.14 – 4.01 (m, 2H), 2.20 – 1.95 (m, 15H).

Synthesis of 1-azido sugar (Man, Glc) using SnCl$_4$.

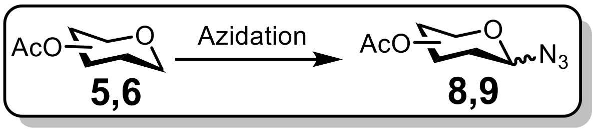

Synthesis of 8 and 9: To a stirred solution of **5** or **6** (4 g, 10.70 mmol, 1.0 eq.) in dry DCM (30 mL), azidotrimethylsilane (TMSiN$_3$, 2.01 mmol, 1.4 eq.) and tin tetrachloride (SnCl$_4$, 0.32 mL, 2.79 mmol, 0.26 eq.) were added sequentially under a nitrogen atmosphere. The reaction mixture was mixed for 2.5 hours at room temperature, then redissolved in DCM (200 mL). The crude product in DCM was extracted with water (100 mL × 3), NaHCO$_3$ (100 mL) and brine (100 mL), then dried over Na$_2$SO$_4$ and concentrated under reduced pressure. The crude product was purified by column chromatography (SiO$_2$, EtOAc/Hexanes 1:1) to afford **8** (Mannose-azide, 95%) or **9** (Glucose-azide, 100%) as a white powder.

N_3-Man-OAc_4 (**8**)

¹H NMR (400 MHz, cdcl$_3$) δ 5.36 (t, *J* = 2.4 Hz, 1H), 5.31 – 5.17 (m, 2H), 5.16 – 5.09 (m, 1H), 4.28 (ddd, *J* = 12.2, 5.4, 2.7 Hz, 1H), 4.18 – 4.03 (m, 3H), 2.15 – 1.93 (m, 14H).

N_3-Glc-OAc_4 (**9**)

¹H NMR (400 MHz, cdcl3) δ 5.20 (t, J = 9.5 Hz, 1H), 5.08 (t, J = 9.8 Hz, 1H), 4.93 (dd, J = 9.6, 8.8 Hz, 1H), 4.63 (d, J = 8.8 Hz, 1H), 4.25 (dd, J = 12.5, 4.8 Hz, 1H), 4.15 (dd, J = 12.5, 2.3 Hz, 1H), 3.77 (ddd, J = 10.1, 4.8, 2.3 Hz, 1H), 2.03 (dd, J = 28.0, 9.4 Hz, 12H).

Synthesis of 1-Bromo sugar (Gal) using HBR.

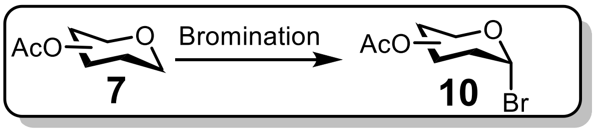

Synthesis of 10: Compound **7** (15 g, 55.5 mmol) was added to a solution of HBr in acetic acid (33% w/w, 30 mL) under a nitrogen atmosphere. The reaction mixture was stirred at room temperature for 90 minutes. The reaction mixture was extracted in DCM (200 mL) and the organic layer was washed sequentially with ice cold water (100 mL × 2), water (75 mL × 3) and $NaHCO_3$ (5% w/v, 100 mL). The organic layer was dried with Na_2SO_4 and concentrated under reduced pressure. The crude product was immediately used in the next step without further purification.

Synthesis of 1-azido sugar (β-Gal) using sodium azide.

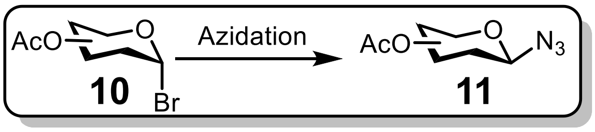

Synthesis of 11: Compound **10** (20 g, 55.5 mmol) and sodium azide (33% w/w, 30 mL, 0. moL) were added to dry DMC (10 mL) under a nitrogen atmosphere. The reaction mixture was stirred at room temperature overnight. The reaction mixture was concentrated under reduced

pressure and redissolved in EtOAc (200 mL). This solution was then extracted with water (100 mL × 3), $NaHCO_3$ (100 mL) and brine (100 mL), sequentially. The organic layer was dried over Na_2SO_4 and concentrated under vacuum to obtain **11** (86%) as a white crystalline powder.

N_3-Gal-OAc_4 (**11**)

^{1}H NMR (400 MHz, cdcl3) δ 5.42 (s, 1H), 5.16 (d, J = 19.2 Hz, 1H), 5.03 (ddd, J = 10.4, 3.3, 1.1 Hz, 2H), 4.58 (s, 1H), 4.09 (d, J = 64.2 Hz, 2H), 2.28 – 1.94 (m, 14H).

Synthesis of 1-Chloro sugar (Gal) by $AlCl_3$.

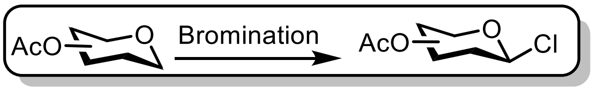

Synthesis of 12: Compound **7** (1 g, 2.56 mmol) and aluminum trichloride ($AlCl_3$, 171 mg, 1.28 mmol) were added to to DCM (6 mL) under a nitrogen atmosphere and stirred at room temperature for 2.5 hours. The reaction mixture was concentrated under reduced pressure, redissolved in cyclohexanes (100 mL), and filtered with celite. The reaction mixture was then concentrated under reduced pressure and extracted with DCM (6 mL). The crude product was then used in the next step without further purification.

Synthesis of 1-azido sugar (α-Gal) by TBAF.3H_2O:

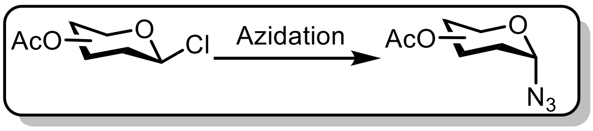

Synthesis of 13: Compound **12** (1.13 g, 3.58 mmol), trimethylsilyl azide (475 μL, 3.58 mmol), and tetrabutylammonium fluoride trihydrate (1.13 g, 3.58 mmol) was dissolved in THF (0.1 M,

25 mL) and stirred at at 65 °C for 24 hours. The solvent was evaporated under reduced pressure and the crude product was purified by column chromatography (SiO_2, EtOAc/Cy 1:3) to afford **13 (45**%) as a white crystalline powder.

N_3-α-Gal-OAc_4 (**12**)

^{1}H NMR (400 MHz, $cdcl_3$) δ 5.64 (d, *J* = 3.9 Hz, 1H), 5.43 (dd, *J* = 3.0, 1.4 Hz, 1H), 5.19 (qd, *J* = 10.8, 3.5 Hz, 2H), 4.37 – 4.29 (m, 1H), 4.19 – 4.02 (m, 2H), 2.24 – 1.91 (m, 13H).

^{13}C NMR (101 MHz, $cdcl_3$) δ 172.30 – 168.61 (m), 107.90, 86.68, 78.54 – 75.10 (m), 70.63 – 65.92 (m), 61.46, 31.75 – 23.12 (m), 21.56 – 18.42 (m).
HRMS for C14H19N3O9: $[M + Na]^+$ calc. = 396.1014; found = 396.1013.

N_3-α-Glc-OAc_4 (**13**)

^{1}H NMR (400 MHz, $cdcl_3$) δ 5.58 (d, *J* = 4.3 Hz, 1H), 5.36 (t, *J* = 9.8 Hz, 1H), 5.07 – 4.98 (m, 1H), 4.92 (dd, *J* = 10.2, 4.3 Hz, 1H), 4.28 – 4.18 (m, 1H), 2.12 – 1.92 (m, 13H).
^{13}C NMR (101 MHz, $cdcl_3$) δ 172.53 – 167.28 (m), 86.14, 78.54 – 75.10 (m), 71.73 – 68.04 (m), 67.84, 61.49, 22.58 – 17.87 (m).
HRMS for C14H19N3O9: $[M + Na]^+$ calc. = 396.1014; found = 396.1011.

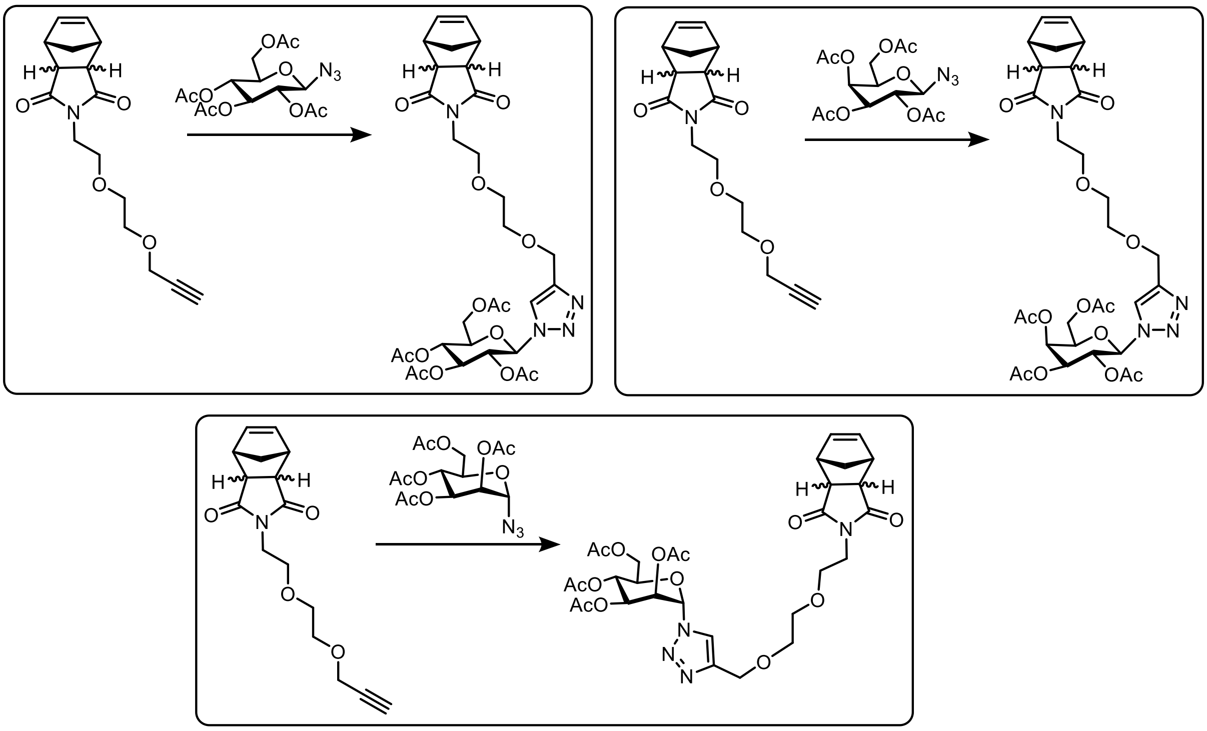

Schemes S2-S4: Alkyne-azide click reaction of both (*exo*, *endo*) norbornene-peg$_2$-alkyne with 1-azido sugars (β-gal-N_3, β-glc-N_3, and α-Man-N_3) to yield 6 “protected” glycomonomers: exo-β-Gal-OAc, endo-β-Gal-OAc, exo-β-Glc-OAc, endo-β-Glc-OAc, exo-α-Man-OAc, and endo-α-Man-OAc.

Synthesis of Protected Glycomonomers.

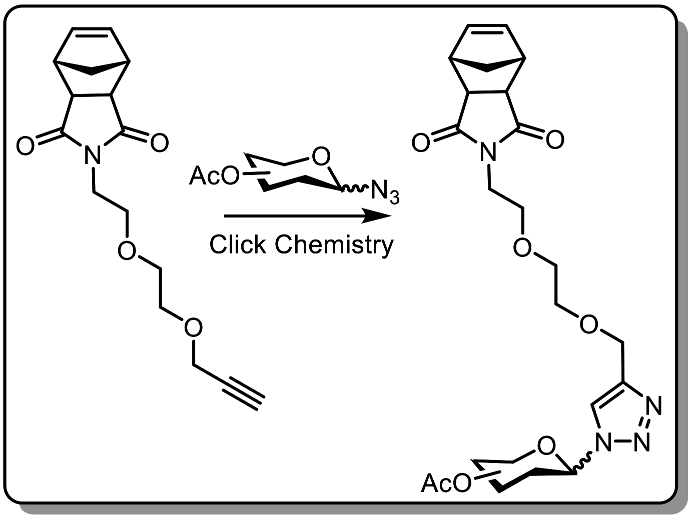

Synthesis of Protected Glycomonomers: To a solution of **3** or **4** (2.5 g, 8.64 mmol) and 1-azido sugar (3.55 g, 9.50 mmol) in dry DMF (3 mL), PMDETA (1.80 mL, 8.64 mmol) and Cu(I)Br (1.24 g, 8.64 mmol) were added sequentially. The reaction mixture was stirred overnight at room temperature, then concentrated under a reduced atmosphere and redissolved in EtOAc (100 mL). The crude product in EtOAc was extracted

with water (100 mL × 3), saturated solution of $NaHCO_3$ (100 mL), and brine (100 mL). The organic phase was dried over Na_2SO_4 and concentrated under reduced pressure to obtain a clear viscous oil. The crude product was purified by column chromatography (SiO_2, DCM/Acetone 60:40) to afford a white powder.

Exo-β-Gal-OAc_4

^{1}H NMR (400 MHz, $cdcl_3$) δ 7.82 (s, 1H), 5.82 (d, *J* = 9.3 Hz, 1H), 5.59 – 5.48 (m, 1H), 5.22 (d, *J* = 10.3 Hz, 1H), 4.62 (s, 2H), 4.14 (d, *J* = 35.2 Hz, 0H), 3.58 (s, 4H), 3.22 (s, 1H), 2.64 (s, 1H), 2.19 (s, 3H), 1.99 (d, *J* = 14.3 Hz, 5H), 1.85 (s, 2H), 1.43 (s, 0H), 1.33 (s, 1H), 1.22 (s, 1H).

^{13}C NMR (101 MHz, $cdcl_3$) δ 178.03, 170.31, 169.99, 168.97, 145.81, 137.79 (d, *J* = 2.5 Hz), 121.12, 86.15, 78.80 – 75.43 (m), 73.90, 70.82, 69.71 (d, *J* = 3.4 Hz), 67.84, 66.91, 64.52, 61.17, 60.35, 47.79, 45.22, 42.66, 37.67, 22.40 – 20.05 (m), 14.16.

HRMS for C30H38N4O13: $[M + Na]^+$ calc. = 685.2328; found = 685.2322.

Endo-β-Gal-OAc_4

^{1}H NMR (400 MHz, $cdcl_3$) δ 7.85 (s, 1H), 6.07 (t, *J* = 1.9 Hz, 2H), 5.83 (d, *J* = 9.3 Hz, 1H), 5.62 – 5.48 (m, 2H), 5.23 (dd, *J* = 10.2, 3.3 Hz, 1H), 4.66 (s, 2H), 4.26 – 4.06 (m, 4H), 3.65 – 3.45 (m, 9H), 3.35 (p, *J* = 1.9 Hz, 2H), 3.24 (dd, *J* = 3.1, 1.4 Hz, 2H), 2.21 (s, 3H), 2.01 (d, *J* = 14.2 Hz, 8H), 1.87 (d, *J* = 2.7 Hz, 6H), 1.70 (dt, *J* = 8.9, 1.7 Hz, 1H), 1.51 (d, *J* = 8.7 Hz, 1H).

^{13}C NMR (101 MHz, $cdcl_3$) δ 177.61, 171.28 – 168.56 (m), 162.48, 134.32, 121.17, 86.14, 73.89, 71.24 – 68.53 (m), 68.05 – 66.45 (m), 64.50, 61.16, 52.04, 45.29 (d, *J* = 90.7 Hz), 37.36, 31.38, 20.24.

HRMS for C30H38N4O13: $[M + Na]^+$ calc. = 685.2328; found = 685.2315.

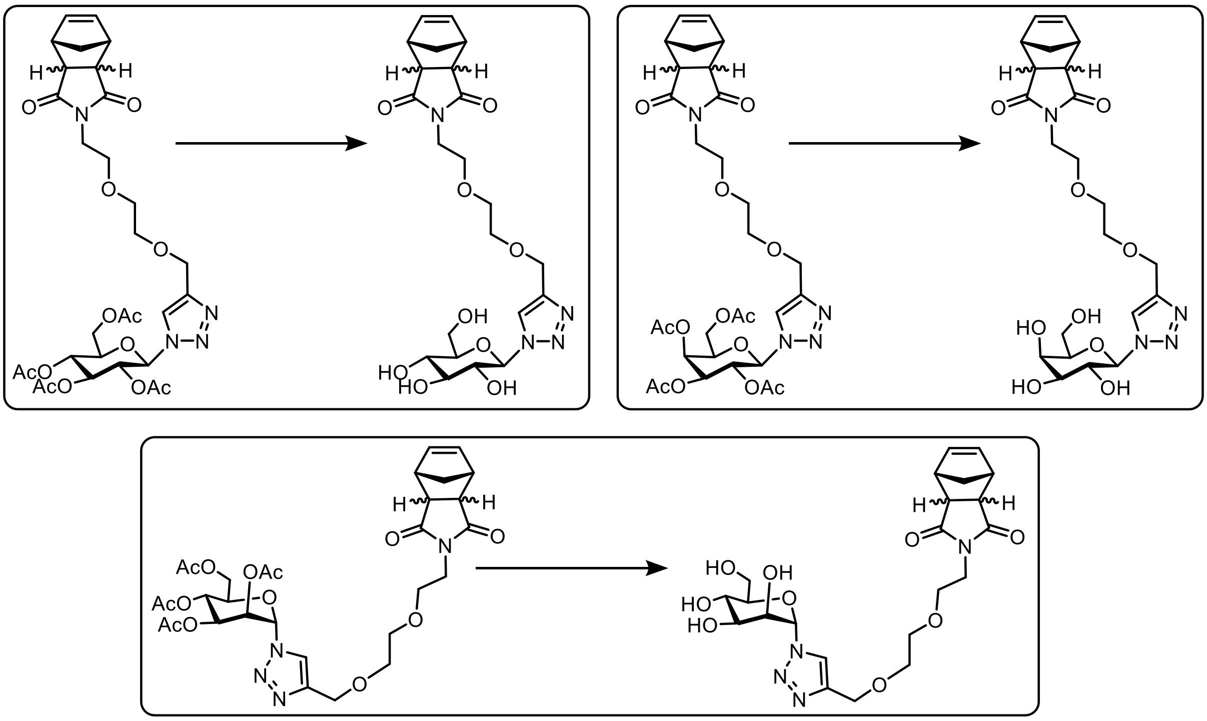

Scheme S5-S7: Deprotection of acetyl-protected glycomonomers results in 6 final, protecting-group-free glycomonomers: exo-β-Gal, endo-β-Gal, exo-β-Glc, endo-β-Glc, exo-α-Man, and endo-α-Man.

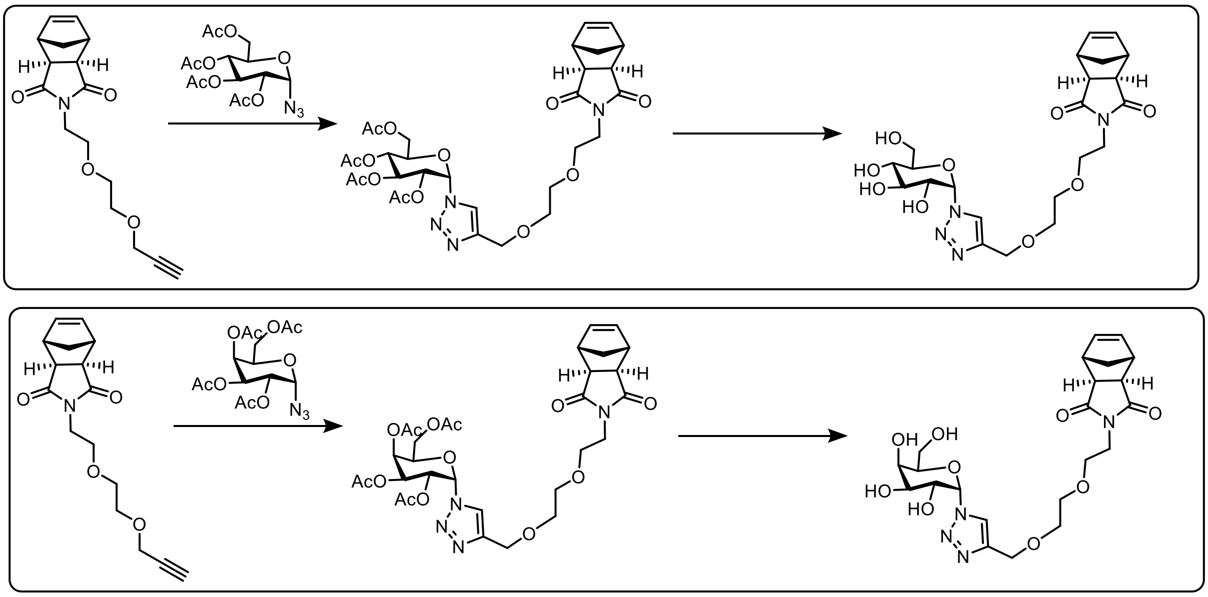

Scheme S8-9: Click reaction of Nor-alkyne with 1-azido-α-Gal or 1-azido-α-Glc and deprotection of acetyl-protected glycomonomers results in 2 final, protecting-group-free glycomonomers: exo-α-Gal, and exo-α-Glc.

Deprotection to afford final glycomonomers.

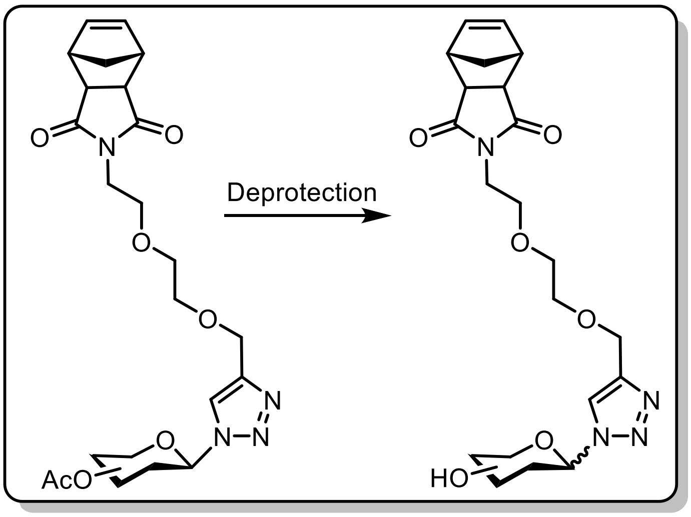

Synthesis of Unprotected Sugars: A solution of protected glycomonomer (1.0 g, 1.51 mmol) and sodium methoxide (NaOMe, 8 mg, 0.15 mmol) in methanol (3 mL) was stirred at room temperature for 2.5 hours. The reaction mixture was neutralized by Amberlite® MB and filtered (0.45 um syringe filter). The reaction mixture was concentrated under reduced pressure and was purified by reverse-phase column chromatography (C_{18}, Water/Acetonitrile 60:40) to afford a white powder.

Yields and composition of glycomonomers.

Monomer	Sugar	Anomer	Yield (%)	Glycomonomer
3	8	α	65	14
3	9	β	75	15
3	11	β	79	16
3	12	α	71	17
3	13	α	69	18
4	8	α	60	19
4	9	β	74	20
4	11	β	77	21

Exo-α-Man (**14**)

^{1}H NMR (400 MHz, d2o) δ 8.02 (s, 1H), 6.16 (t, J = 1.9 Hz, 2H), 5.95 (d, J = 2.2 Hz, 1H), 4.63 (s, 9H), 4.48 (s, 2H), 3.97 (dd, J = 9.1, 3.5 Hz, 1H), 3.70 – 3.55 (m, 3H), 3.56 – 3.40 (m, 9H), 3.14 (ddd, J = 8.9, 5.6, 2.8 Hz, 1H), 2.96 (p, J = 1.7 Hz, 2H), 2.62 (d, J = 1.3 Hz, 2H), 1.15 (dp, J = 10.0, 1.6 Hz, 1H), 1.07 (dt, J = 10.1, 1.6 Hz, 1H).

^{13}C NMR (101 MHz, d_2o) δ 181.48, 144.19, 137.66, 124.60, 86.63, 75.98, 68.98, 66.40 (d, *J* = 9.6 Hz), 60.31, 47.66, 37.76.

HRMS for C22H30N4O9: $[M + Na]^+$ calc. = 517.1905; found = 517.1902.

Exo-β-Glc (**15**)

^{1}H NMR (400 MHz, d_2o) δ 8.10 (s, 1H), 6.17 (t, *J* = 1.8 Hz, 2H), 5.60 (d, *J* = 9.2 Hz, 1H), 4.51 (s, 2H), 3.84 (t, *J* = 9.2 Hz, 1H), 3.80 – 3.70 (m, 1H), 3.68 – 3.42 (m, 11H), 2.98 (dq, *J* = 5.4, 1.6 Hz, 2H), 2.64 (d, *J* = 1.3 Hz, 2H), 1.22 (dq, *J* = 10.1, 1.6 Hz, 1H), 1.14 – 1.05 (m, 1H).

^{13}C NMR (101 MHz, d_2o) δ 181.55, 144.17, 137.68, 124.13, 87.37, 78.75, 75.81, 72.19, 68.99, 68.97, 68.81, 66.48, 62.89, 60.29, 47.70, 44.89, 41.93, 37.79.
HRMS for C22H30N4O9: $[M + Na]^+$ calc. = 517.1905; found = 517.1903.

Exo-β-Gal (**16**)

^{1}H NMR (400 MHz, $CDCl_3$) δ 8.05 (s, 1H), 6.24 (s, 2H), 5.30 (s, 2H), 4.96 (s, 1H), 4.50 (s, 3H), 4.25 (d, *J* = 11.5 Hz, 1H), 4.04 (s, 1H), 3.79 (s, 2H), 3.70 (s, 3H), 3.60 (d, *J* = 5.2 Hz, 2H), 3.54 (dd, *J* = 11.4, 4.9 Hz, 6H), 3.19 (d, *J* = 8.4 Hz, 5H), 2.65 (s, 2H), 2.16 (d, *J* = 1.0 Hz, 1H), 2.00 (s, 0H), 1.42 (d, *J* = 9.5 Hz, 1H), 1.26 (d, *J* = 9.5 Hz, 1H).

^{13}C NMR (101 MHz, $CDCl_3$) δ 178.45, 178.43, 144.62, 137.83, 123.60, 88.25, 77.81, 77.24, 73.79, 70.05, 69.72, 68.75, 66.88, 64.09, 61.03, 47.82, 45.22, 42.68, 37.84, 30.93.
HRMS for C22H30N4O9: $[M + Na]^+$ calc. = 517.1905; found = 517.1902.

Exo-α-Gal (**17**)

^{1}H NMR (400 MHz, $CDCl_3$) δ 7.87 (s, 1H), 6.17 (d, *J* = 7.9 Hz, 3H), 5.43 – 4.66 (m, 3H), 4.49 (d, *J* = 11.3 Hz, 3H), 4.35 (s, 1H), 4.15 (s, 1H), 3.87 (d, *J* = 6.4 Hz, 1H), 3.73 – 3.36 (m, 11H), 3.13 (s, 2H), 2.58 (s, 2H), 2.09 (s, 0H), 1.36 (d, *J* = 9.6 Hz, 1H), 1.25 – 1.15 (m, 1H).

^{13}C NMR (101 MHz, $CDCl_3$) δ 178.31, 143.90, 137.84, 85.67, 78.26 – 76.55 (m), 74.11, 69.80 (d, *J* = 7.2 Hz), 64.22, 47.82, 45.24, 42.70, 37.81.
HRMS for C22H30N4O9: $[M + Na]^+$ calc. = 517.1905; found = 517.1902.

Exo-α-Glc (**18**)

^{1}H NMR (400 MHz, $CDCl_3$) δ 7.86 (s, 1H), 6.15 (d, *J* = 19.9 Hz, 3H), 5.54 (s, 1H), 5.28 (s, 1H), 5.16 (s, 1H), 4.50 (s, 2H), 4.34 (s, 1H), 4.04 (s, 1H), 3.85 – 3.35 (m, 11H), 3.13 (s, 2H), 3.03 – 2.78 (m, 2H), 2.59 (s, 2H), 2.09 (d, *J* = 1.2 Hz, 9H), 1.36 (d, *J* = 9.7 Hz, 1H), 1.26 – 1.09 (m, 2H).

^{13}C NMR (101 MHz, $CDCl_3$) δ 206.98, 178.28, 143.95, 137.83, 125.98, 85.41, 78.78 – 75.80 (m), 75.45, 73.78, 69.79 (d, *J* = 4.4 Hz), 66.94, 64.31 (d, *J* = 18.6 Hz), 60.90, 47.82, 45.24, 42.69, 37.81, 30.93, 25.35.
HRMS for C22H30N4O9: $[M + Na]^+$ calc. = 517.1905; found = 517.1901.

Endo-α-Man (**19**)

^{1}H NMR (400 MHz, d_2o) δ 8.03 (d, *J* = 1.2 Hz, 1H), 5.96 (t, *J* = 1.7 Hz, 1H), 5.86 (d, *J* = 2.2 Hz, 2H), 4.63 (t, *J* = 1.1 Hz, 12H), 4.57 – 4.48 (m, 2H), 4.01 – 3.93 (m, 1H), 3.70 – 3.56 (m, 3H), 3.53 – 3.40 (m, 5H), 3.34 (h, *J* = 4.8 Hz, 5H), 3.29 – 3.22 (m, 2H), 3.18 – 3.09 (m, 3H), 2.85 – 2.80 (m, 0H), 2.67 (q, *J* = 1.0 Hz, 0H), 1.51 (dd, *J* = 8.9, 1.8 Hz, 1H), 1.41 (d, *J* = 8.9 Hz, 1H).

^{13}C NMR (101 MHz, d_2o) δ 181.49, 144.15, 134.15, 124.63, 75.98, 70.36, 68.94, 68.15, 66.78, 66.34, 60.30, 51.76, 45.11 (d, *J* = 103.9 Hz), 37.48.
HRMS for C22H30N4O9: $[M + Na]^+$ calc. = 517.1905; found = 517.1903.

Endo-β-Glc (**20**)

^{1}H NMR (400 MHz, $CDCl_3$) δ 8.01 (s, 1H), 6.03 (s, 2H), 5.69 – 5.59 (m, 2H), 5.46 (s, 1H), 5.39 (s, 1H), 4.54 (s, 2H), 4.48 (s, 1H), 4.01 (dd, *J* = 11.7, 6.1 Hz, 1H), 3.79 (d, *J* = 13.3 Hz, 1H), 3.71 (s, 3H), 3.62 – 3.53 (m, 4H), 3.52 – 3.37 (m, 7H), 3.30 (s, 2H), 3.23 (d, *J* = 3.0 Hz, 2H), 3.16 (s, 3H), 1.65 (d, *J* = 8.4 Hz, 1H), 1.49 (d, *J* = 8.5 Hz, 1H), 1.20 (d, *J* = 6.1 Hz, 1H).

^{13}C NMR (101 MHz, $CDCl_3$) δ 178.14, 144.61, 134.40, 123.60, 87.69, 78.94, 77.23, 72.52, 69.74, 69.08, 67.15, 64.43, 64.11, 61.06, 52.12, 45.78, 44.87, 37.51, 25.35.
HRMS for C22H30N4O9: $[M + Na]^+$ calc. = 517.1905; found = 517.1905.

Endo-β-Gal (**21**)

^{1}H NMR (400 MHz, $CDCl_3$) δ 8.07 (s, 1H), 6.02 (s, 2H), 5.56 (d, *J* = 8.4 Hz, 1H), 5.31 (s, 2H),

4.95 (s, 1H), 4.52 (s, 3H), 4.25 (s, 1H), 4.03 (s, 1H), 3.77 (d, *J* = 13.8 Hz, 2H), 3.70 (s, 2H), 3.58 – 3.43 (m, 6H), 3.41 (d, *J* = 4.9 Hz, 2H), 3.36 (s, 2H), 3.30 (s, 2H), 3.23 (d, *J* = 3.0 Hz, 2H), 1.64 (d, *J* = 8.4 Hz, 1H), 1.48 (d, *J* = 8.5 Hz, 1H).

^{13}C NMR (101 MHz, $CDCl_3$) δ 178.21, 178.18, 144.63, 134.40, 123.62, 88.27, 77.85, 77.24, 73.79, 70.06, 69.71, 68.76, 67.14, 64.05, 61.05, 52.12, 45.78, 44.87, 37.51.
HRMS for C22H30N4O9: $[M + Na]^+$ calc. = 517.1905; found = 517.1902.

Synthesis of Grubbs 3rd Generation Catalyst:

Grubbs 2nd generation catalyst (1.18 mmol, 1 equiv.), toluene (20 mL) and pyridine (0.1 mol, 85 equiv., 8 mL) were stirred at room temperature for 45 minutes under a nitrogen atmosphere. The reaction mixture was then cooled to -80 °C and hexanes (40 mL) were added. The product was dried under nitrogen and stored at -80 °C.

Grubbs 3rd Generation Catalyst NMR

^{1}H NMR (400 MHz, $cdcl_3$) δ 8.62 (s, 1H), 7.82 (s, 1H), 7.62 (d, *J* = 7.8 Hz, 1H), 7.45 (t, *J* = 7.4 Hz, 1H), 7.22 (s, 1H), 7.05 (t, *J* = 7.7 Hz, 1H), 7.00 (s, 1H), 6.92 (s, 1H), 6.73 (s, 1H), 4.15 (s, 1H), 4.02 (s, 1H), 2.62 (s, 3H), 2.30 (dd, *J* = 16.4, 9.8 Hz, 4H), 2.23 (s, 2H), 1.65 (s, 1H).

General Polymerization Procedure.

A solution of glycomonomer (2.13 mmol, 35 equiv.) in dry DMF (0.5 mL) was prepared in the glovebox. Grubbs 3rd generation catalyst (10.6 µmol, 1 equiv) in dry DMF (100 uL) was quickly added to the monomer solution and stirred at room temperature for 15-30 minutes. The reaction was quenched with ethyl vinyl ether (EVE, 100 µL) and stirred for an additional 15-30 minutes. The reaction mixture was precipitated with cold diethyl ether (10 mL), centrifuged at 100xg for 5 minutes, and decanted. The reaction mixture was dialyzed against DI water for 24 hours and the polymer was obtained as a white solid.

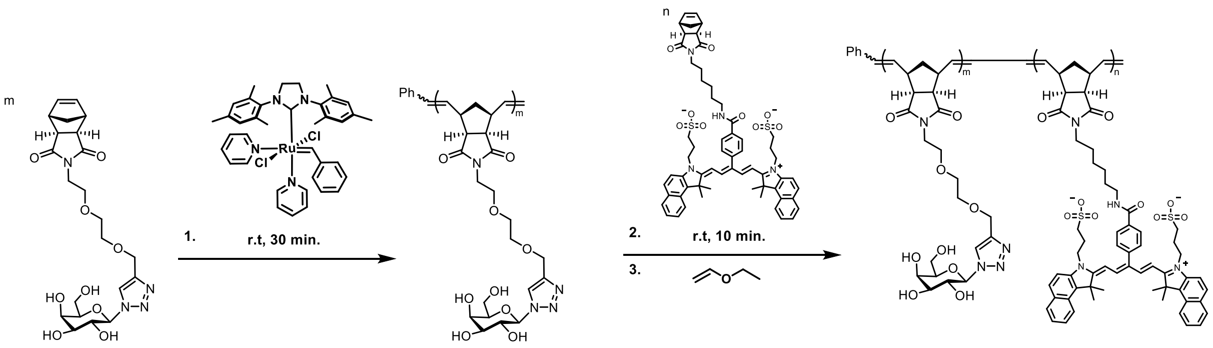

Scheme S10: Polymerization of *exo*-β-Gal with Cy 5 dye monomer at end and quench with diethyl ether. Similarly, other dye labelled glycopolymers were synthesized.

General Polymerization Procedure for Dye-Labelled Polymers.

A solution of glycomonomer (2.13 mmol, 35 equiv.) in dry DMF (0.5 mL) was prepared in the glovebox. Grubbs 3rd generation catalyst (10.6 µmol, 1 equiv) in dry DMF (100 uL) was quickly added to the monomer solution and stirred at room temperature for 30 minutes. The reaction

mixture was precipitated with cold diethyl ether, centrifuged at 100xg for 5 minutes, and decanted. Cy 5.5 dye (1 eq) was added and the reaction was stirred for an additional 15 minutes. The polymerization was quenched with EVE (200 μL) and stirred for an additional 30 minutes. The reaction mixture was precipitated with cold diethyl ether (10 mL), centrifuged at 100xg for 5 minutes, and decanted. The reaction mixture was dialyzed against DI water for 24 hours and the polymer was obtained as a dark green solid product.

General Procedure for Polymerization Kinetics (Room Temperature):

A solution of glycomonomer (10 g/L, 35 equiv.) in deuterated DMF (d_6-DMF) was prepared in a J-young NMR tube and ^{1}H-NMR was obtained as a starting point. Then, Grubbs 3rd generation catalyst (0.5 g/L, 1.0 equiv.) was quickly added to the tube, shaken, and ^{1}HNMR of the polymerization was obtained every 2 minutes for 2 hours at room temperature or until the monomer peak (6.20 ppm) had disappeared. The addition of EVE was added to quench the reaction.

General Procedure for Polymerization Kinetics (60 °C):

To a solution of monomer (30 g/L, 35 equ) in dry DMF in a glovebox, Grubbs 3rd generation catalyst (5 g/L, 1.0 equ.) dissolved in dry DMF was added and stirred. Every 1-3 hours for a total of 24 hours, an aliquot (50 μL) of the reaction mixture was removed, quenched with excess EVE (100 μL), and analyzed via gel permeation chromatography (GPC).

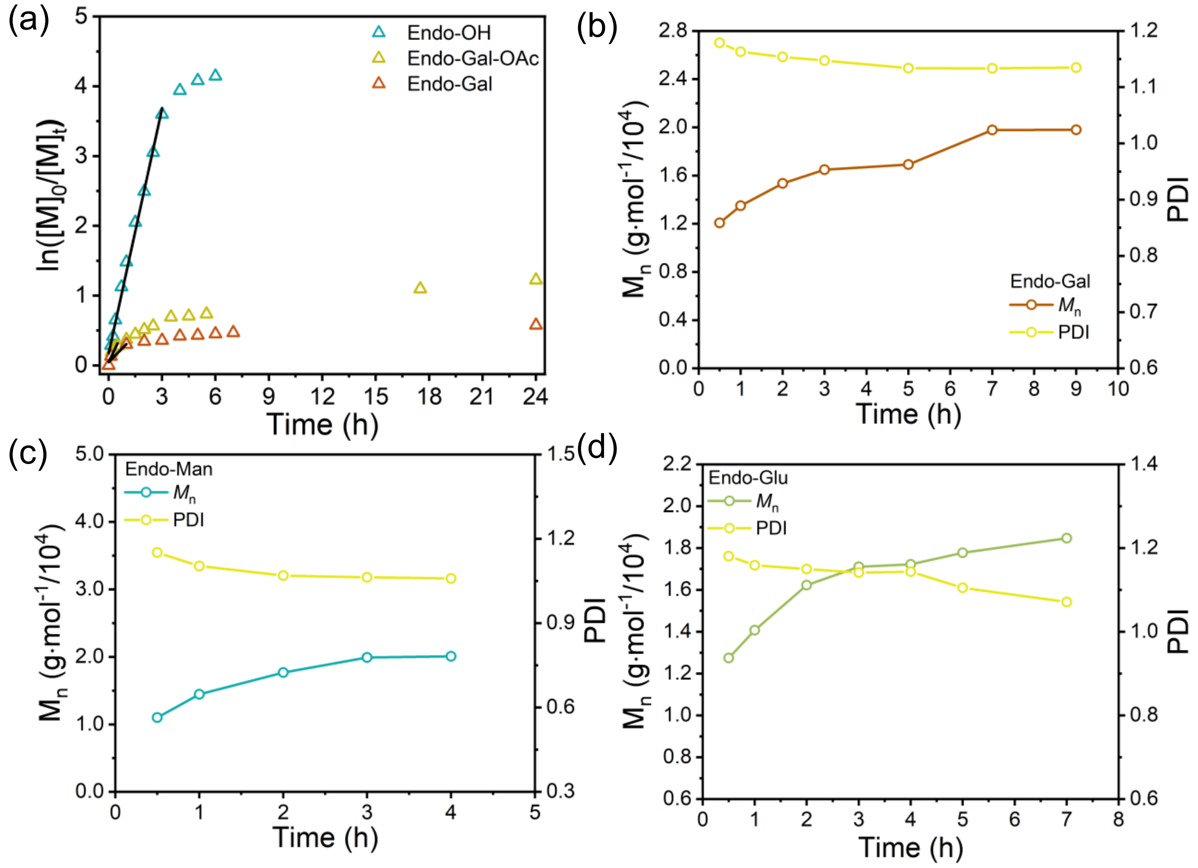

Figure S1: (a) Log plots of endo-monomers with different end-groups as a function of time, as measured by ^{1}H NMR, Lines represent lines of best fit, using linear least-squares fitting, and (b-d) Conversion of endo-PGPs with different sugars (β-Gal, β-Glc, and α-Man) as a function of time, as measured by GPC as the function of weight number average (M_n) and Dispersity (PDI).

Table S1: Polymerization conditions for Endo-PGPs.

PGPs	Conditions	Catalyst Conc. (mol/L)	Solvent	Target DP	M_n (KDa)	M_w (KDa)	Time	Temp (°C)	DP*	Đ
Endo-Gal-OH	Solvent	0.01	DCM	35	-	-	12	r.t	-	-
		0.01	DMF	35	8.9	11.2	12	r.t	18	1.26
		0.01	THF	35	-	-	12	r.t	-	1.31
	Catalyst Conc. G-2*	0.01	DMF	35	7.9	9.2	12	r.t	16	1.16
		0.03	DMF	35	8.9	10.0	12	r.t	18	1.12
		0.06	DMF	35	10.9	11.6	12	r.t	22	1.06
	Temp.	0.06	DMF	35	17.9	19.1	12	35	35	1.07
		0.06	DMF	35	18.1	19.5	9	45	36	1.08
		0.06	DMF	35	19.0	20.4	6	60	38	1.07
	Catalyst	G 3 - 0.6	DMF	35	13.1	17.3	12	60	26	1.32
		HG - 0.6	DMF	35	7.4	10.7	12	60	15	1.44

- *DP calculated as Mn/Mw,monomer
- - No Polymerization
- G-2* (Grubbs 2nd generation Catalyst® M204 Modified with pyridine)
- G 3 (Grubbs 3rd generation Catalyst® M300)
- HG (Hoveyda-Grubbs Catalyst® M720)

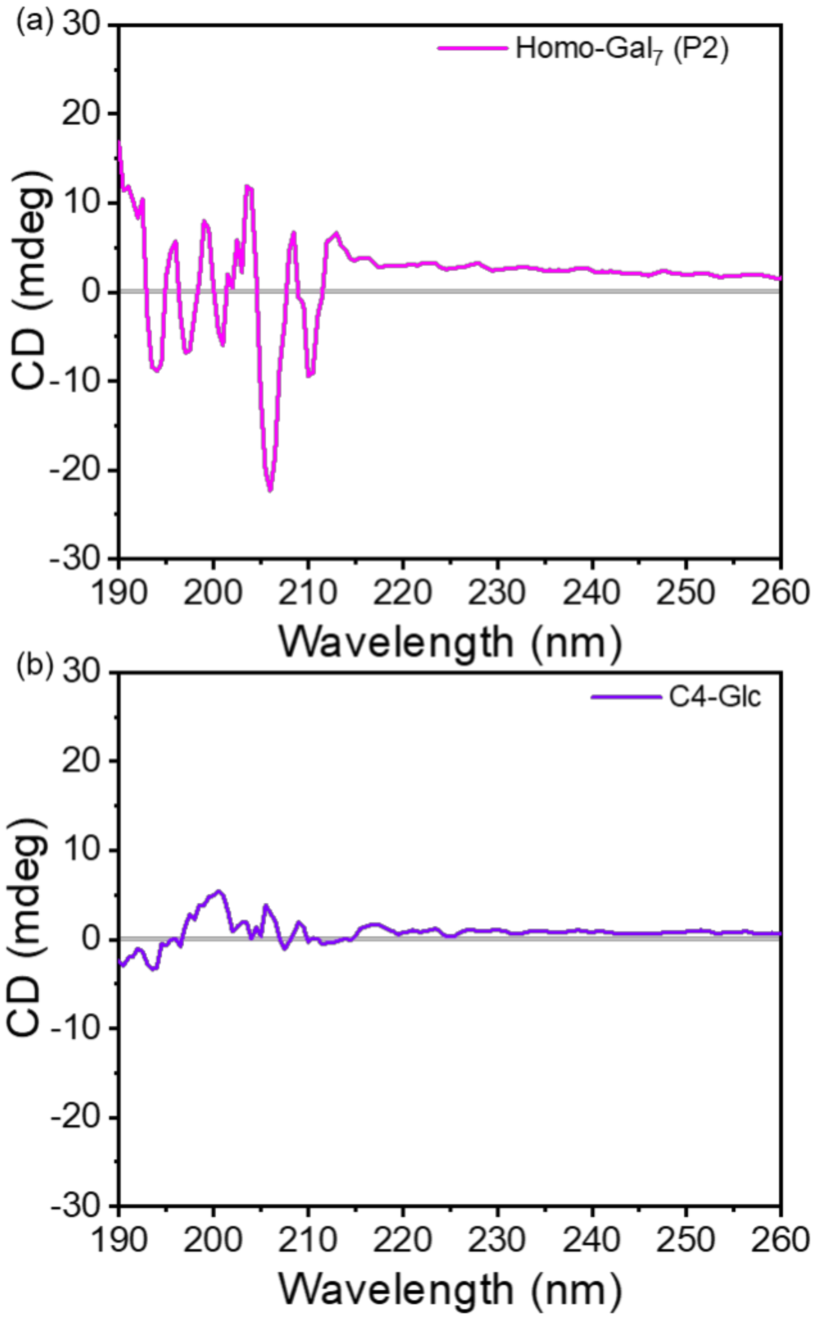

Figure S2: Circular dichroism (CD) spectrum of Homo-Gal7 (P2), (b) CD spectrum of C4-Glc and (c) CD spectrum of P4-OH.

Note: Homo-Gal7 (P2) and C4-Glc were synthesized previously in our lab by using exo-norbornene precursor and synthesis details can be found in references 1 and 2 below.[1,2]

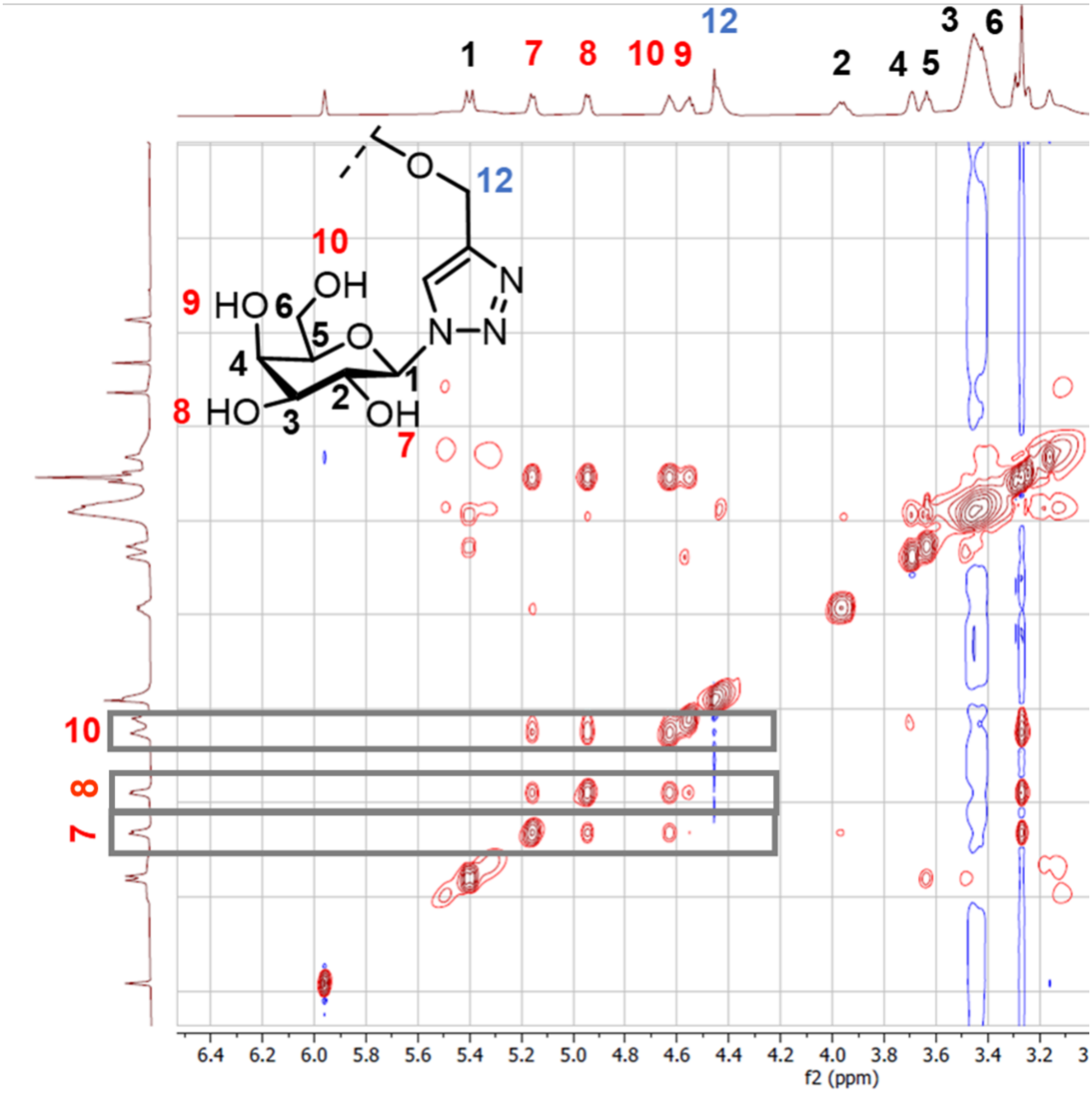

Figure S3: Nuclear Overhauser Effect Spectroscopy (NOESY) NMR to determine the spatial hydrogen interactions in *endo*-β-Gal PGPs.

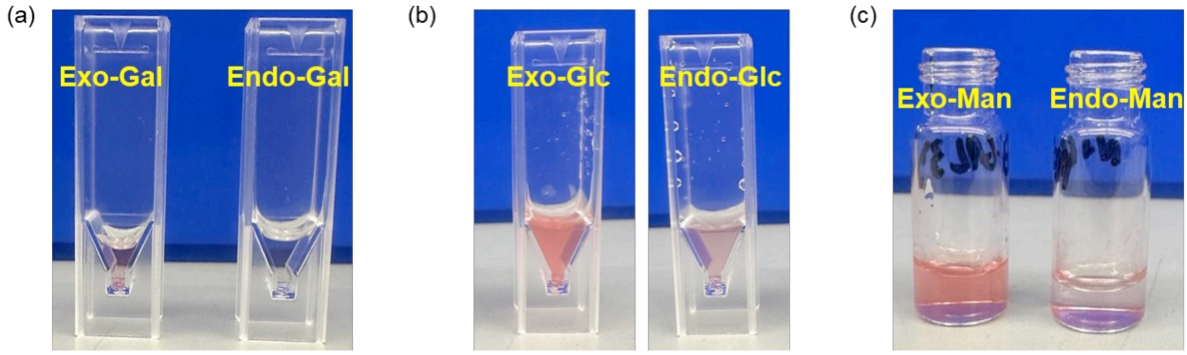

Figure S4: Visual observation of Nile red interaction with exo- and endo-PGPs and formation of pink precipitates: (a) *exo*-β-Gal and *endo*-β-Gal, (b) *exo*-β-Glc and *endo*-β-Glc, and (c) *exo*-α-Man and *endo*-α-Man.

Simulation Setup:

All-atom molecular dynamics (MD) simulations of the PGP systems (35-mer endo-βgal, exo-βgal, endo-αgal, and exo-αgal) were performed using the GROMACS 2020.4 program.[1] To closely replicate experimental conditions, molecular dynamics simulations were performed on 35-mer PGPs, matching the polymer chain length used in experiments. The Initial structures of the PGP monomeric unit (**Figure S5 (i)**) and Nile red molecule were generated using Shrödinger Release 2024-4: MAESTRO.[2] The CHARMM General Force Field (CGenFF)[3] and CHARMM36[4] were used to obtain bonded and non-bonded parameters for the monomer and also bonded parameters for the linkage between adjacent monomers. Nile red was also represented using CGenFF parameters. The 35-mer polymer structure for exo and endo was then generated, such that the next monomer was placed at an equilibrium bond-distance (atom C1 and C2, see SI) value of 1.34 Å as described by CGenFF parameters. The new monomer was randomly rotated along the backbone, which was progressively grown along the y-direction (**Figure S5 (ii)**).(Github link in main manuscript) Three independent configurations for each system were prepared to minimize statistical errors and improve the reliability of the simulation results. The 35-mer structures were first relaxed in a vacuum using NVT MD simulations for 15 ps (**Figure S5 (iii)**). These structures were then solvated in a cubic box with sides of 145 Å with TIP3P explicit water (~99,000 water molecules) such that the resulting density was close to 1 g/cc. For PGPs with βgal, we conducted simulations both in the presence and absence of Nile red, where 10 molecules of Nile red were randomly added to the system, such that they were within ~12 Å of the PGP structure. Periodic boundary conditions (PBC) with a 12 Å cutoff for van der Waals and electrostatic interactions were applied, and the Particle-Mesh Ewald (PME)[5] method was used for long-range electrostatics.[6] The LINCS[7] algorithm was used to constrain bond lengths involving hydrogen atoms, and energy minimization was conducted using the steepest descent algorithm.[8,9] The O-H bonds and the fictitious H-H bond in water were fixed using the SETTLE algorithm.[10] The Velocity-Verlet integration algorithm was used with a timestep of 1 fs to advance trajectories.[11] Initially, the systems were equilibrated for 100 ps in the NVT and NPT ensembles. This was followed by a 300 ns simulation in the NPT ensemble for all studied systems. The temperature was controlled at 298 K using the Nosé-Hoover thermostat with a 1 ps coupling constant[12], while pressure was maintained at 1 bar using the Parrinello-Rahman barostat with a 2 ps coupling constant.(Parrinello and Rahman 1981; Peesapati et al. 2021) The atomic trajectories, including positions, velocities, and forces, were recorded every 1 ps and visualized using visual molecular dynamics (VMD).[13] The trajectories from all three starting configurations were used to perform structural and dynamic analysis of systems, with results averaged over these three simulations.

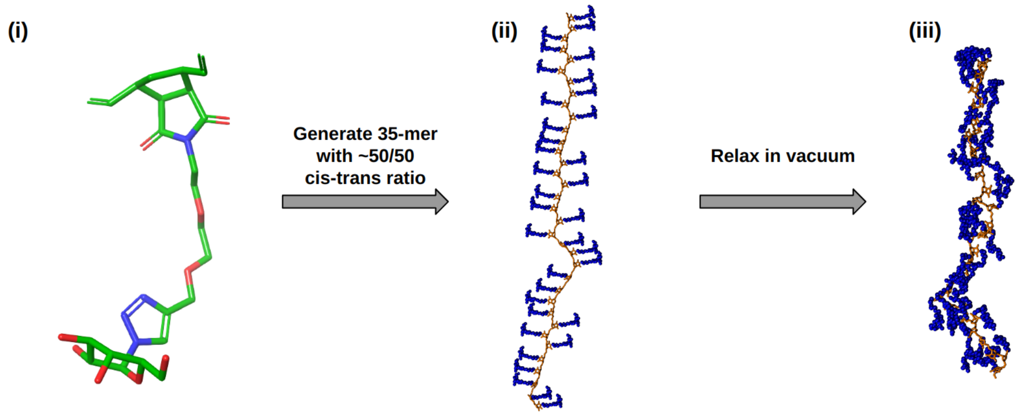

Figure S5: 3D representation of (i) monomeric unit of *endo*-β-Gal PGP and (ii) 35-mer PGP structure. (iii) shows the relaxed structure used as a starting configuration for MD simulations.

Table S2: R_g (Å) of backbone and sidechains of PGPs at different timesteps

	System	10-100ns	100-200ns	200-300ns
Backbone	*endo*-β-Gal PGP with Nile red	16.70±1.85	15.77±0.97	15.21±0.58
	endo-β-Gal PGP without Nile red	17.47±1.93	16.40±0.52	16.03±0.35
	exo-β-Gal PGP with Nile red	17.24±1.98	16.50±0.68	16.29±0.45
	exo-β-Gal PGP without Nile red	15.45±1.09	14.99±0.55	15.04±0.65
Sidechain	*endo*-β-Gal PGP with Nile red	4.29±0.02	4.29±0.02	4.29±0.02
	endo-β-Gal PGP without Nile red	4.27±0.02	4.28±0.02	4.28±0.02
	exo-β-Gal PGP with Nile red	4.26±0.02	4.26±0.02	4.25±0.02
	exo-β-Gal PGP without Nile red	4.26±0.02	4.26±0.02	4.26±0.02

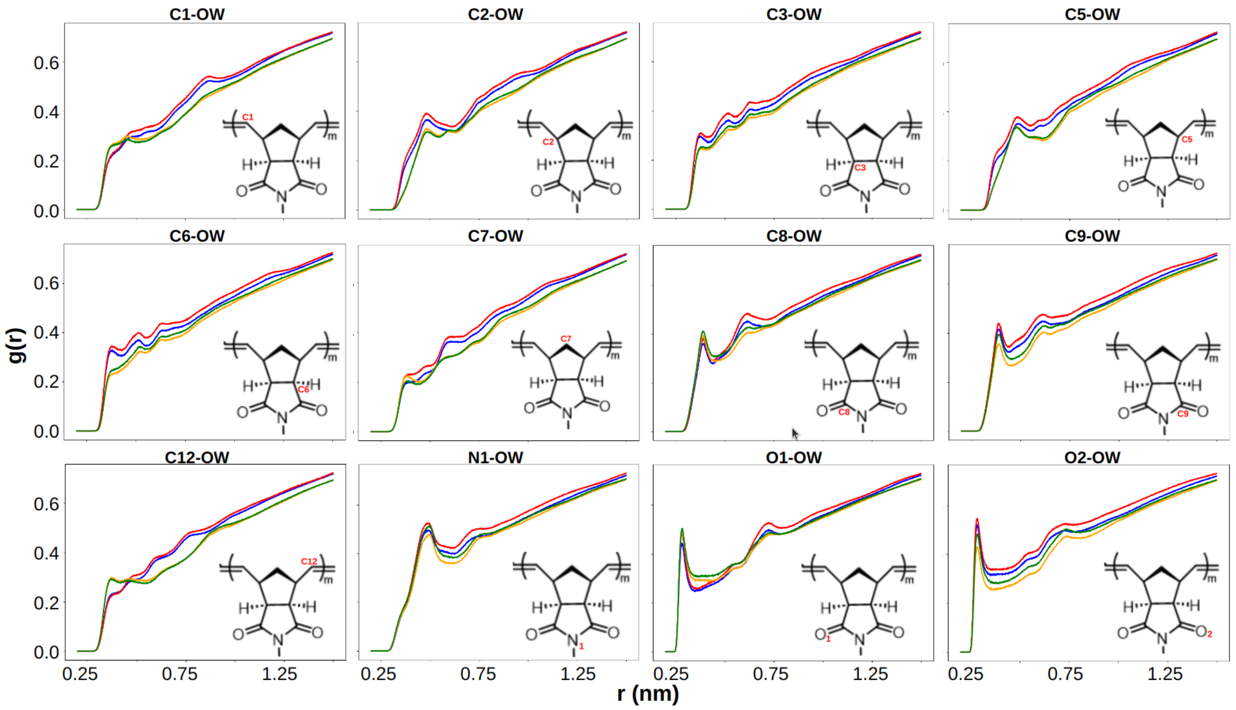

Figure S6: RDF between backbone heavy atoms and water oxygen for *endo*-β-Gal PGP (blue) and *exo*-β-Gal PGP (yellow) in the presence of Nile red and *endo*-β-Gal PGP (red) and *exo*-β-Gal PGP (green) in the absence of Nile red.

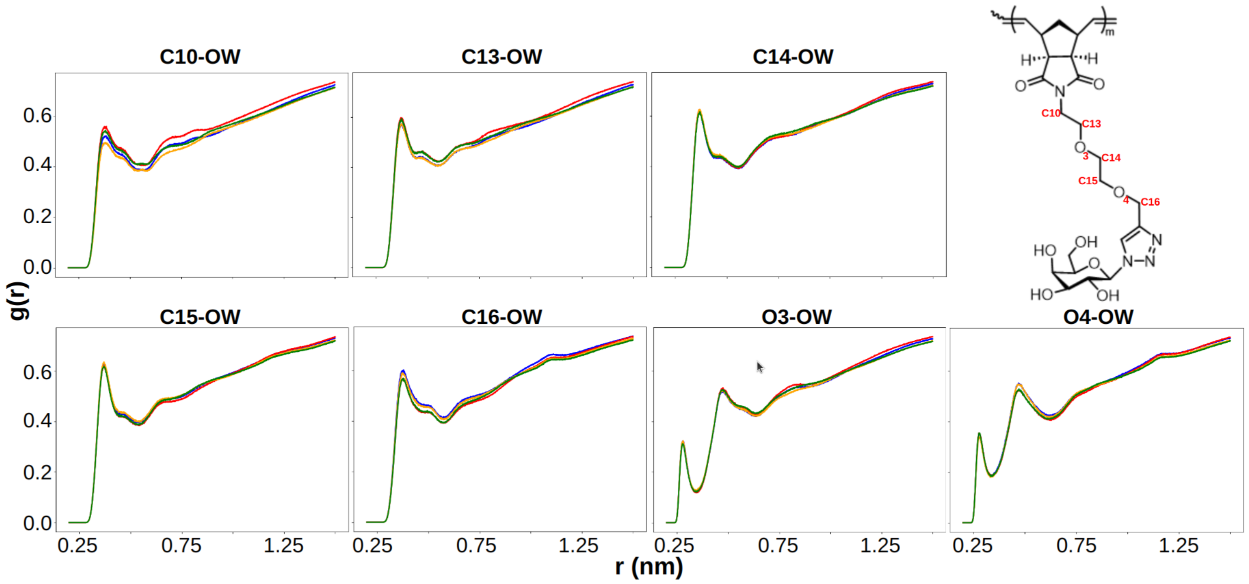

Figure S7: RDF between PEG linker heavy atoms and water oxygen for *endo*-β-Gal PGP (blue) and *exo*-β-Gal PGP (yellow) in the presence of Nile red and *endo*-β-Gal PGP (red) and *exo*-β-Gal PGP (green) in the absence of Nile red.

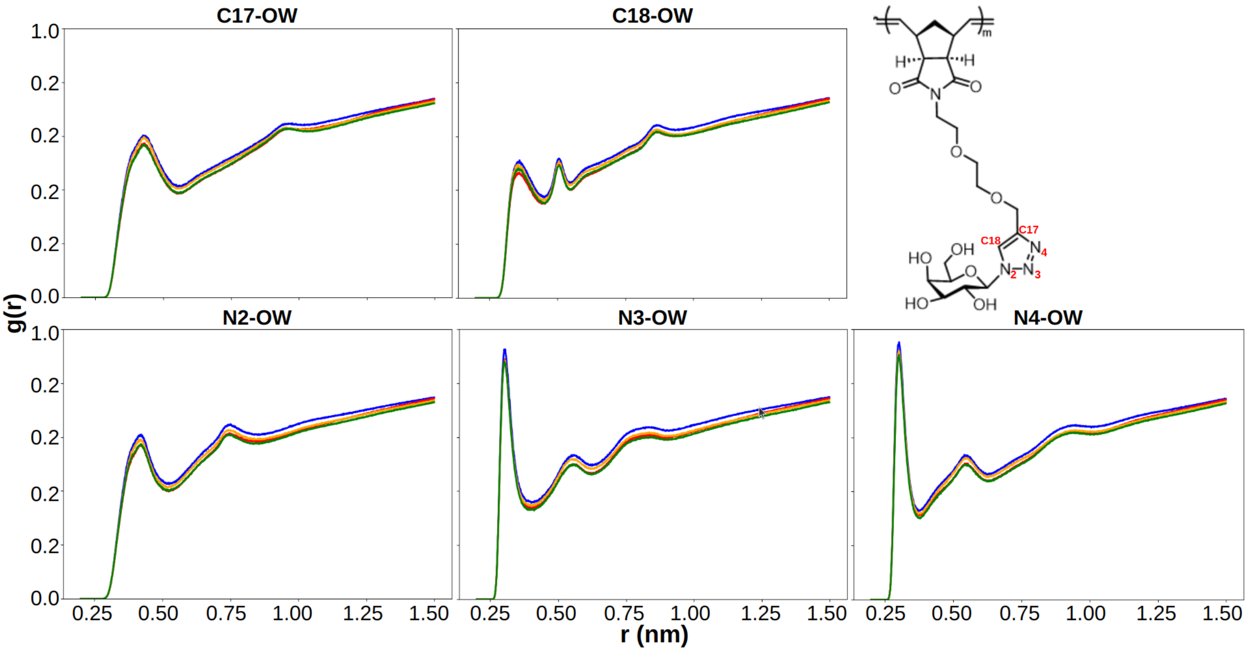

Figure S8: RDF between Triazole moiety heavy atoms and water oxygen for *endo*-β-Gal PGP (blue) and *exo*-β-Gal PGP (yellow) in the presence of Nile red and *endo*-β-Gal PGP (red) and *exo*-β-Gal PGP (green) in the absence of Nile red.

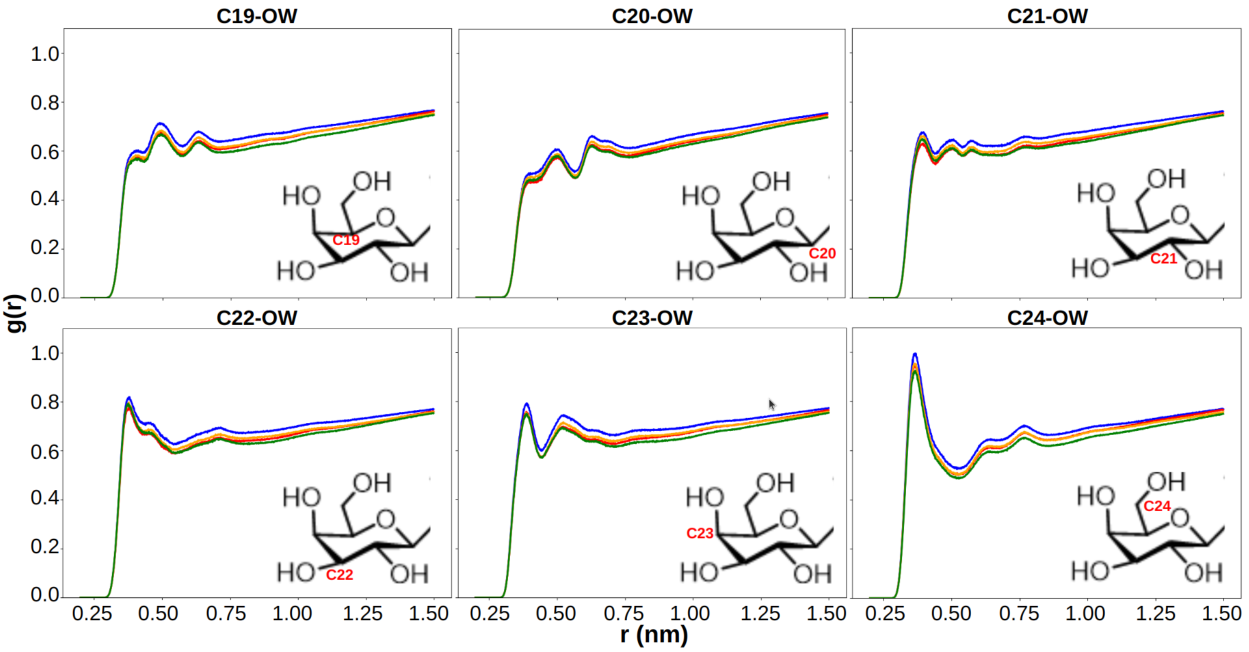

Figure S9: RDF between Galactose carbon atoms and water oxygen for *endo*-β-Gal PGP (blue) and *exo*-β-Gal PGP (yellow) in the presence of Nile red and *endo*-β-Gal PGP (red) and *exo*-β-Gal PGP (green) in the absence of Nile red.

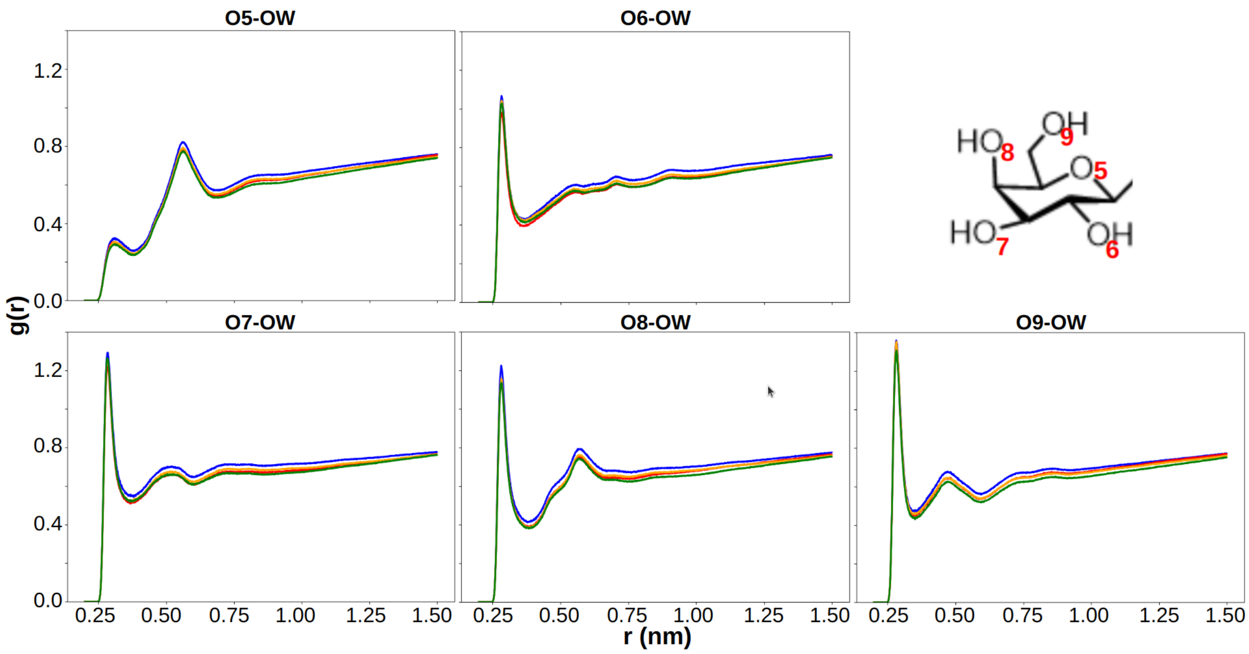

Figure S10: RDF between galactose oxygen atoms and water oxygen for *endo*-β-Gal PGP (blue) and *exo*-β-Gal PGP (yellow) in the presence of Nile red and *endo*-β-Gal PGP (red) and *exo*-β-Gal PGP (green) in the absence of Nile red.

Spatial distribution of Nile red around PGP

The frequency of Nile red molecules within 15 Å of both the PGP backbone and sidechain was analyzed over the 290 ns of the trajectory. Note that the 15 Å threshold distance for counting Nile red molecules near PGP was based on radial distribution function (RDF) analysis between PGP heavy atoms and Nile red. For both endo and exo-PGP, the RDF revealed that ~15 Å distance from PGP corresponds to the trough of the first shell, marking the boundary beyond which the probability of direct interactions significantly decreases. To ensure consistency, this distance threshold was applied uniformly when analyzing the proximity of Nile red to both the PGP backbones and sidechains. Note, when performing this analysis, the PGP backbone and sidechain atoms were analyzed separately, as a result, the same Nile red molecule could be counted in both categories if it was within the 15 Å range of both the backbone and side chain simultaneously.

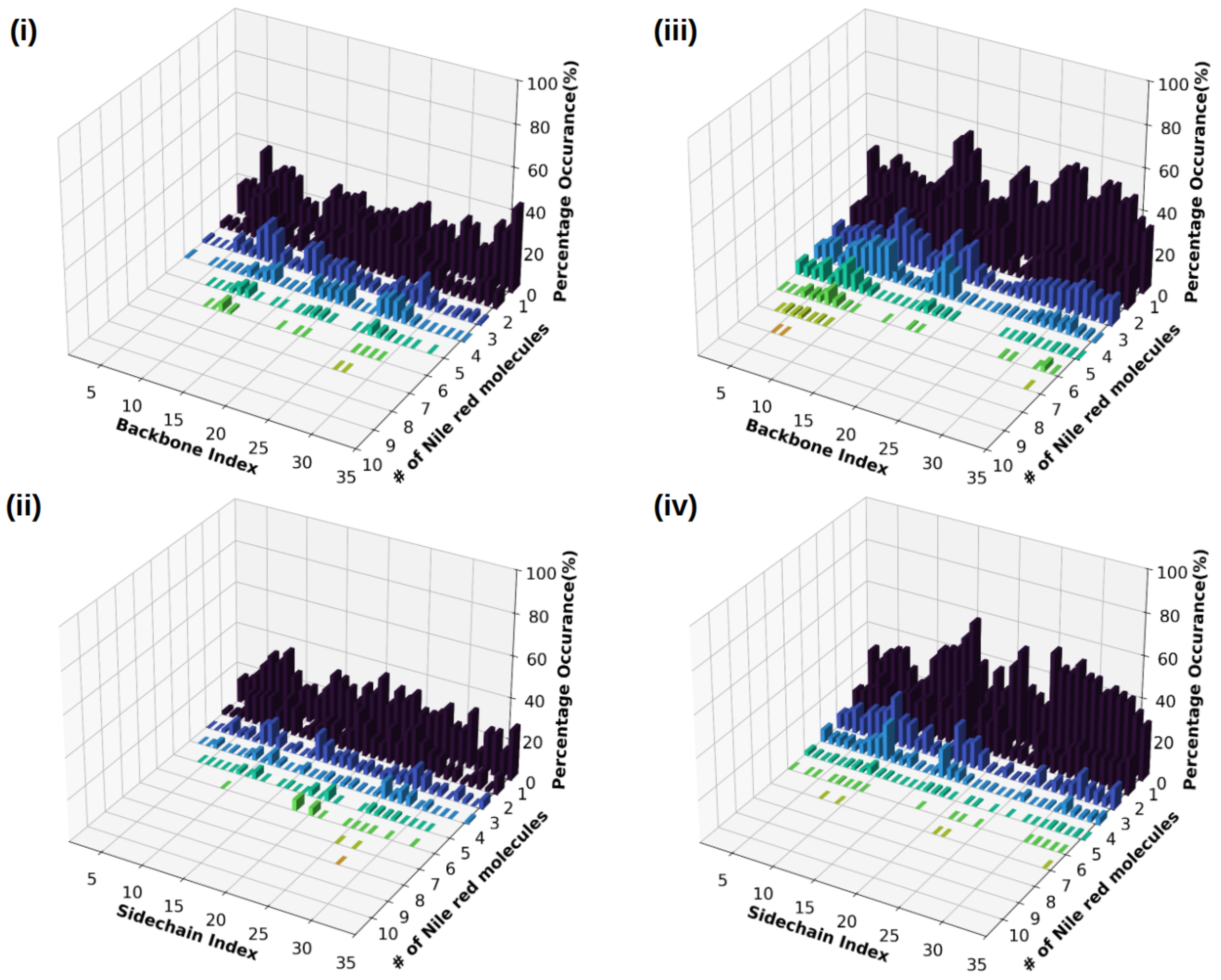

Figure S11: Nile red absorption onto the backbone and side chains of (i and ii) *endo*-β-Gal PGP and (iii and iv) *exo*-β-Gal PGP, respectively. The 35mer backbone and sidechain indices are shown, along with the percentage of simulation time that a given number of Nile red molecules were within the 15 Å threshold of a specific backbone or sidechain group.

Table S3: Percentage of the 290 ns simulation time that different numbers of Nile red molecules were within close proximity of different backbone indices of endo-PGP.

	Nile red molecules									
Backbone indices	1	2	3	4	5	6	7	8	9	10
1	14.07	2.98	1.30	0.50	0.00	0.00	0.00	0.00	0.00	0.00
2	15.47	2.83	0.13	0.00	0.00	0.00	0.00	0.00	0.00	0.00
3	14.57	1.92	0.00	0.00	0.00	0.00	0.00	0.00	0.00	0.00
4	33.85	7.79	0.17	0.01	0.00	0.00	0.00	0.00	0.00	0.00
5	22.76	19.14	6.29	0.20	0.00	0.00	0.00	0.00	0.00	0.00
6	26.12	22.47	4.63	0.30	0.00	0.00	0.00	0.00	0.00	0.00
7	26.73	23.77	3.17	0.88	0.03	0.00	0.00	0.00	0.00	0.00

8	23.60	12.16	14.03	0.83	0.00	0.00	0.00	0.00	0.00	0.00
9	13.55	8.32	18.36	3.73	1.81	0.01	0.00	0.00	0.00	0.00
10	12.64	3.80	14.36	2.89	4.03	4.12	0.00	0.00	0.00	0.00
11	6.95	10.40	5.90	5.11	3.90	1.20	0.00	0.00	0.00	0.00
12	17.71	3.91	2.08	7.17	0.00	0.00	0.00	0.00	0.00	0.00
13	24.52	8.51	2.73	0.63	0.00	0.00	0.00	0.00	0.00	0.00
14	21.72	8.66	12.04	0.49	0.00	0.00	0.00	0.00	0.00	0.00
15	22.24	14.33	13.27	0.65	0.00	0.00	0.00	0.00	0.00	0.00
16	24.24	11.39	8.06	0.37	0.00	0.00	0.00	0.00	0.00	0.00
17	18.16	15.08	6.98	7.32	0.40	0.01	0.00	0.00	0.00	0.00
18	17.04	26.63	8.61	5.45	0.86	0.00	0.00	0.00	0.00	0.00
19	19.40	24.73	8.19	6.38	1.98	0.10	0.00	0.00	0.00	0.00
20	20.76	19.16	5.41	6.18	1.97	0.02	0.00	0.00	0.00	0.00
21	20.14	21.45	4.72	7.67	0.55	0.00	0.00	0.00	0.00	0.00
22	22.72	23.83	3.60	1.18	0.00	0.00	0.00	0.00	0.00	0.00
23	26.56	17.11	1.86	0.02	0.00	0.00	0.00	0.00	0.00	0.00
24	28.39	17.79	3.65	0.08	0.00	0.00	0.00	0.00	0.00	0.00
25	18.10	20.33	7.93	8.00	0.12	0.00	0.00	0.00	0.00	0.00
26	21.41	14.38	5.55	10.02	3.96	0.07	0.04	0.00	0.00	0.00
27	20.48	10.98	7.15	9.46	2.27	0.16	0.02	0.00	0.00	0.00
28	15.95	10.87	13.44	6.03	1.50	0.06	0.00	0.00	0.00	0.00
29	27.13	6.39	5.93	2.03	0.03	0.00	0.00	0.00	0.00	0.00
30	24.81	4.48	2.73	0.36	0.03	0.00	0.00	0.00	0.00	0.00
31	15.96	4.46	0.56	0.10	0.01	0.00	0.00	0.00	0.00	0.00
32	17.50	5.46	1.88	0.06	0.00	0.00	0.00	0.00	0.00	0.00
33	29.59	9.66	1.67	0.08	0.01	0.00	0.00	0.00	0.00	0.00
34	28.33	11.89	2.85	0.11	0.00	0.00	0.00	0.00	0.00	0.00
35	40.06	7.72	1.06	0.00	0.00	0.00	0.00	0.00	0.00	0.00

Table S4: Percentage of the 290 ns simulation time that different numbers of Nile red molecules were within close proximity of different backbone indices of exo-PGP.

	Nile red molecules									
Backbone indices	1	2	3	4	5	6	7	8	9	10
1	31.19	10.72	8.48	6.30	6.14	0.17	0.00	0.00	0.00	0.00
2	22.22	14.06	6.53	8.50	4.92	0.19	0.00	0.00	0.00	0.00

3	22.08	14.60	8.19	9.79	4.63	0.22	0.01	0.00	0.00	0.00
4	29.93	15.29	9.16	4.21	8.31	2.92	1.21	0.00	0.00	0.00
5	27.15	19.64	12.49	9.79	2.90	2.42	1.08	0.03	0.00	0.00
6	23.72	9.70	11.53	12.41	7.66	3.15	1.73	0.01	0.00	0.00
7	22.78	9.37	6.73	12.42	12.10	6.73	0.31	0.00	0.00	0.00
8	20.50	14.70	14.83	16.35	8.15	3.54	0.83	0.00	0.00	0.00
9	22.08	14.08	23.40	15.00	4.32	0.85	0.22	0.00	0.00	0.00
10	26.68	14.66	19.09	16.72	2.67	0.07	0.00	0.00	0.00	0.00
11	32.09	29.89	15.39	5.47	0.01	0.00	0.00	0.00	0.00	0.00
12	47.63	23.33	14.19	3.04	0.12	0.00	0.00	0.00	0.00	0.00
13	51.09	28.27	7.04	1.74	0.36	0.00	0.00	0.00	0.00	0.00
14	43.57	32.58	8.34	2.44	0.11	0.02	0.00	0.00	0.00	0.00
15	29.10	35.34	15.20	4.48	0.00	0.00	0.00	0.00	0.00	0.00
16	21.08	13.69	20.72	10.41	1.33	0.00	0.00	0.00	0.00	0.00
17	18.15	14.03	14.22	17.24	3.53	0.60	0.00	0.00	0.00	0.00
18	24.22	15.89	17.84	12.45	1.72	0.00	0.00	0.00	0.00	0.00
19	36.37	32.58	7.39	1.28	1.55	0.00	0.00	0.00	0.00	0.00
20	41.43	21.62	4.97	1.28	0.83	0.00	0.00	0.00	0.00	0.00
21	35.22	7.78	1.73	0.22	0.00	0.00	0.00	0.00	0.00	0.00
22	26.45	2.58	2.00	0.06	0.00	0.00	0.00	0.00	0.00	0.00
23	28.02	7.21	3.14	0.64	0.00	0.00	0.00	0.00	0.00	0.00
24	39.87	10.43	5.33	1.40	0.00	0.00	0.00	0.00	0.00	0.00
25	47.24	16.48	6.94	1.30	0.00	0.00	0.00	0.00	0.00	0.00
26	49.46	16.95	7.98	1.02	0.00	0.00	0.00	0.00	0.00	0.00
27	48.44	16.81	10.12	1.65	0.10	0.00	0.00	0.00	0.00	0.00
28	35.28	21.39	10.62	3.70	0.72	0.41	0.00	0.00	0.00	0.00
29	36.98	14.97	10.99	3.95	1.09	0.25	0.00	0.00	0.00	0.00
30	45.54	11.24	11.05	5.46	0.33	0.00	0.00	0.00	0.00	0.00
31	45.62	14.39	13.93	4.32	0.05	0.00	0.00	0.00	0.00	0.00
32	39.47	20.43	14.32	5.62	0.64	0.01	0.00	0.00	0.00	0.00
33	42.02	17.40	11.54	2.59	1.09	2.78	0.05	0.00	0.00	0.00
34	32.61	14.32	10.20	1.75	0.49	0.20	0.00	0.00	0.00	0.00
35	29.50	19.01	12.51	0.69	0.00	0.00	0.00	0.00	0.00	0.00

Table S5: Percentage of the 290 ns simulation time that different numbers of Nile red molecules were within close proximity of different side-chain indices of endo-PGP.

	Nile red molecules									
Backbone indices	1	2	3	4	5	6	7	8	9	10
1	11.28	1.86	0.00	0.00	0.00	0.00	0.00	0.00	0.00	0.00
2	11.71	1.63	0.09	0.00	0.00	0.00	0.00	0.00	0.00	0.00
3	15.64	3.40	1.50	0.13	0.00	0.00	0.00	0.00	0.00	0.00
4	21.47	13.40	5.83	2.30	0.00	0.00	0.00	0.00	0.00	0.00
5	27.26	13.86	2.04	0.13	0.12	0.00	0.00	0.00	0.00	0.00
6	24.99	14.98	3.41	0.74	0.03	0.00	0.00	0.00	0.00	0.00
7	30.87	15.65	1.48	0.15	0.02	0.00	0.00	0.00	0.00	0.00
8	20.65	9.56	9.08	1.41	0.11	0.00	0.00	0.00	0.00	0.00
9	14.63	16.67	11.24	3.65	1.64	0.01	0.00	0.00	0.00	0.00
10	16.00	13.25	5.32	0.83	0.20	0.00	0.00	0.00	0.00	0.00
11	12.60	2.61	0.74	5.35	3.48	0.00	0.00	0.00	0.00	0.00
12	18.20	6.24	2.54	1.94	0.24	0.00	0.00	0.00	0.00	0.00
13	26.41	7.68	0.24	0.02	0.00	0.00	0.00	0.00	0.00	0.00
14	24.75	11.17	1.93	0.08	0.05	0.00	0.00	0.00	0.00	0.00
15	21.49	10.87	12.69	2.61	0.60	0.00	0.00	0.00	0.00	0.00
16	16.41	6.58	8.42	0.36	0.00	0.00	0.00	0.00	0.00	0.00
17	24.67	9.54	3.80	0.59	0.00	0.00	0.00	0.00	0.00	0.00
18	22.66	20.39	5.54	1.72	3.08	5.29	0.00	0.00	0.00	0.00
19	31.54	15.51	5.81	0.79	0.08	0.00	0.00	0.00	0.00	0.00
20	19.53	9.61	2.64	1.48	4.72	3.79	0.00	0.00	0.00	0.00
21	27.41	8.18	4.02	1.84	0.25	0.00	0.00	0.00	0.00	0.00
22	23.72	17.00	2.71	0.71	0.00	0.00	0.00	0.00	0.00	0.00
23	27.65	10.81	2.40	0.03	0.00	0.00	0.00	0.00	0.00	0.00
24	20.00	17.43	5.10	1.28	0.14	0.01	0.00	0.00	0.00	0.00
25	19.64	20.50	4.24	8.09	1.76	0.01	0.00	0.00	0.00	0.00
26	21.42	14.36	5.69	2.88	1.38	0.00	0.00	0.00	0.00	0.00
27	25.59	10.62	10.26	6.31	0.91	0.10	0.02	0.00	0.00	0.00
28	17.53	9.73	7.12	4.71	1.21	0.00	0.00	0.00	0.00	0.00
29	17.08	5.17	2.29	0.48	0.08	0.01	0.00	0.00	0.00	0.00
30	26.64	5.40	3.19	2.20	0.01	0.00	0.00	0.00	0.00	0.00

31	8.49	1.49	0.21	0.14	0.00	0.00	0.00	0.00	0.00	0.00
32	19.59	4.61	4.61	0.15	0.04	0.01	0.00	0.00	0.00	0.00
33	19.77	8.03	1.86	0.06	0.00	0.00	0.00	0.00	0.00	0.00
34	12.33	1.68	0.17	0.00	0.00	0.00	0.00	0.00	0.00	0.00
35	24.32	8.31	4.30	1.01	0.00	0.00	0.00	0.00	0.00	0.00

Table S6: Percentage of the 290 ns simulation time that different numbers of Nile red molecules were within close proximity of different side-chain indices of exo-PGP.

	Nile red molecules									
Backbone indices	1	2	3	4	5	6	7	8	9	10
1	25.60	12.10	6.90	6.00	1.89	0.02	0.00	0.00	0.00	0.00
2	20.83	13.20	8.60	3.10	0.85	0.00	0.00	0.00	0.00	0.00
3	25.94	15.29	11.94	3.91	0.32	0.00	0.00	0.00	0.00	0.00
4	28.16	21.83	8.30	3.86	1.01	0.05	0.00	0.00	0.00	0.00
5	29.08	18.03	13.24	2.74	0.32	0.00	0.00	0.00	0.00	0.00
6	20.74	14.05	13.47	4.73	0.97	0.09	0.00	0.00	0.00	0.00
7	14.83	16.92	15.35	7.81	1.55	0.53	0.01	0.00	0.00	0.00
8	17.99	18.79	23.08	9.72	1.01	0.00	0.00	0.00	0.00	0.00
9	29.67	15.15	13.16	17.54	3.77	0.26	0.02	0.00	0.00	0.00
10	35.12	17.92	11.99	3.42	0.91	0.01	0.00	0.00	0.00	0.00
11	36.29	10.38	5.35	2.61	0.89	0.00	0.00	0.00	0.00	0.00
12	37.67	19.00	11.19	4.77	0.23	0.00	0.00	0.00	0.00	0.00
13	44.51	16.28	4.26	1.22	0.09	0.00	0.00	0.00	0.00	0.00
14	52.84	23.55	2.46	0.42	0.00	0.00	0.00	0.00	0.00	0.00
15	33.55	30.05	6.57	2.05	0.00	0.00	0.00	0.00	0.00	0.00
16	21.42	9.66	18.26	13.58	1.11	0.00	0.00	0.00	0.00	0.00
17	32.32	21.51	10.19	5.39	1.05	0.01	0.00	0.00	0.00	0.00
18	23.89	15.43	12.58	4.65	1.26	0.00	0.00	0.00	0.00	0.00
19	37.24	22.40	7.61	2.00	0.05	0.00	0.00	0.00	0.00	0.00
20	45.47	12.42	1.69	0.25	0.00	0.00	0.00	0.00	0.00	0.00
21	27.82	5.14	0.73	0.82	0.44	0.10	0.00	0.00	0.00	0.00
22	25.00	6.75	4.01	0.89	0.51	0.05	0.00	0.00	0.00	0.00
23	21.75	3.67	0.67	0.04	0.00	0.00	0.00	0.00	0.00	0.00

24	49.36	15.62	1.56	0.31	0.02	0.00	0.00	0.00	0.00	0.00
25	41.81	9.70	1.55	0.05	0.00	0.00	0.00	0.00	0.00	0.00
26	41.25	9.92	7.56	2.68	0.00	0.00	0.00	0.00	0.00	0.00
27	46.74	8.98	1.26	0.00	0.00	0.00	0.00	0.00	0.00	0.00
28	41.20	14.90	5.29	0.58	0.00	0.00	0.00	0.00	0.00	0.00
29	40.89	16.99	4.13	1.10	0.04	0.00	0.00	0.00	0.00	0.00
30	35.48	14.09	6.28	0.87	0.00	0.00	0.00	0.00	0.00	0.00
31	38.38	14.76	12.25	5.51	0.87	0.31	0.00	0.00	0.00	0.00
32	36.85	14.64	6.77	2.31	1.00	0.01	0.00	0.00	0.00	0.00
33	35.72	14.06	6.06	1.41	0.18	0.00	0.00	0.00	0.00	0.00
34	30.45	13.97	4.38	2.43	0.65	0.02	0.00	0.00	0.00	0.00
35	26.11	17.45	10.48	3.26	0.17	0.00	0.00	0.00	0.00	0.00

Non-bonded Interaction energy

To quantify the interactions between Nile Red and PGP, we computed the non-bonded interaction energy between Nile Red and each of the 35 monomeric units of PGP using the "*gmx_energy*" tool.(Tewari et al. 2024) We excluded the first 10 ns of the 300 ns trajectory and got an average over the 290 ns and an average over three independent runs. To further pinpoint the preferred binding regions of Nile red on PGP, we decomposed the interaction energy based on specific functional groups within the PGP sidechain. We analyzed the interactions of Nile red with the PEG, triazole, and monosaccharide moieties separately. This helped us identify which chemical features of PGP contribute the most to Nile red adsorption. **Figures S12** and **Table S14** show the interaction energies between different moieties with Nile red. For all moieties analyzed, the exo conformation exhibits more favorable interaction energies compared to the endo conformation. Additionally, the different moieties show comparable interaction energies.

Table S7: Non-bonded interaction energies between Nile red and different groups/moieties of *exo*- and *endo*-β-Gal PGPs over 290 ns

	Endo				Exo			
Residue Index	Backbone	PEG	Triazole	Galactose	Backbone	PEG	Triazole	Galactose
1	-0.56±1.73	1376±29	1377±22	1376±22	-6.51±5.35	1374±23	1375±22	1377±22
2	-0.50±1.34	-0.22±0.89	-0.12±0.70	-0.22±1.55	-6.92±4.03	-2.86±2.75	-1.33±2.14	-1.04±3.76

3	-0.19±0.65	-0.06±0.48	-0.18±0.85	-0.35±2.15	-6.70±3.44	-1.23±1.89	-1.22±2.62	-0.50±4.08
4	-0.75±1.65	-0.26±1.15	-0.61±1.46	-1.51±4.57	-8.67±4.36	-2.15±2.24	-0.93±1.74	-1.32±2.96
5	-3.29±2.82	-0.59±1.59	-1.14±2.05	-1.07±2.57	-7.47±3.70	-3.04±3.18	-2.31±3.49	-2.28±4.03
6	-4.43±2.75	-0.63±1.30	-1.06±1.95	-1.26±3.81	-4.26±2.35	-1.56±1.78	-1.75±2.93	-0.80±4.33
7	-4.18±3.06	-1.10±2.16	-1.55±2.56	-1.00±2.75	-6.63±2.37	-3.43±2.35	-2.60±2.83	-2.91±3.62
8	-4.72±3.66	-0.33±1.12	-1.00±1.64	-1.13±3.20	-11.90±3.69	-3.63±2.47	-1.31±2.81	-2.21±5.23
9	-6.22±5.30	-0.15±0.76	-1.95±2.46	0.12±5.46	-17.02±4.33	-5.59±2.53	-1.79±1.69	-1.67±4.48
10	-4.72±3.34	-0.14±0.88	-0.25±0.76	-0.39±1.60	-10.48±4.28	-2.32±2.86	-1.43±2.68	-1.12±3.63
11	-3.34±2.82	-0.09±0.60	-1.59±2.92	-0.74±3.61	-6.64±2.96	-1.51±1.63	-1.11±2.15	-1.35±3.19
12	-2.08±2.50	-0.34±1.73	-0.58±1.57	-0.59±2.97	-5.88±2.73	-2.25±2.04	-2.70±2.54	-1.11±3.43
13	-2.54±2.61	-0.22±0.99	-1.20±1.94	-1.03±2.71	-7.96±3.57	-3.12±2.34	-2.30±2.68	-2.00±4.21
14	-2.98±3.12	-0.10±0.38	-0.52±1.71	-0.28±1.80	-9.23±3.77	-2.58±2.81	-1.17±1.87	-1.92±3.73
15	-2.21±2.77	-0.42±1.38	-1.76±2.30	-3.39±4.19	-5.48±3.33	-4.04±2.97	-3.83±2.60	-2.78±3.32
16	-2.38±2.54	-0.76±2.06	-0.41±1.08	-0.93±2.77	-5.43±4.31	-4.37±3.16	-3.45±2.53	-4.23±5.81

17	-2.36±2.53	-1.34±2.85	-1.06±1.98	-1.28±3.00	-4.15±3.24	-3.75±2.90	-2.02±2.63	-1.40±4.12
18	-2.83±2.52	-3.62±5.10	-1.55±2.45	-2.14±5.38	-4.83±3.95	-1.46±1.70	-0.77±1.59	-1.06±3.09
19	-4.34±3.16	-2.07±3.04	-2.05±3.04	-1.22±4.11	-4.79±4.39	-2.17±2.03	-2.00±2.76	-2.95±5.21
20	-3.96±2.57	-0.78±1.56	-3.77±5.28	-1.25±4.35	-2.82±2.81	-1.14±1.50	-0.55±1.18	-0.59±2.78
21	-5.73±3.04	-2.21±2.70	-0.45±1.05	-0.57±1.95	-1.83±2.41	-0.71±1.39	-0.89±1.92	-1.07±3.46
22	-4.50±2.59	-2.49±5.08	-0.76±1.86	-0.53±4.72	-1.24±1.85	-0.54±0.93	-0.78±1.49	-1.42±3.29
23	-3.62±2.30	-2.95±3.06	-1.78±2.53	-1.22±2.90	-1.31±2.31	-0.46±1.13	-0.29±0.91	-0.39±2.00
24	-5.11±2.51	-4.97±4.57	-1.30±1.70	-1.12±3.15	-1.49±1.97	-1.49±1.92	-2.33±3.51	-1.79±4.09
25	-5.14±2.81	-2.40±3.01	-1.85±2.85	-1.20±2.96	-3.70±2.97	-0.95±1.31	-0.56±1.68	-0.66±3.53
26	-4.20±3.94	-1.54±2.52	-1.01±1.88	-1.04±3.46	-4.49±3.34	-2.39±1.74	-6.39±4.00	-7.38±5.74
27	-3.86±3.62	-0.17±1.03	-1.94±3.24	-0.79±6.07	-4.72±3.22	-1.97±1.91	-1.23±2.09	-1.16±2.89
28	-2.60±3.04	-0.23±0.99	-1.23±2.49	-2.21±4.30	-4.08±3.70	-1.37±2.28	-0.96±2.06	-2.01±3.67
29	-1.26±2.17	-0.43±1.75	-0.47±1.43	-0.58±2.28	-4.11±3.85	-1.38±2.03	-1.03±2.21	-2.48±4.52
30	-1.31±2.05	-1.24±2.79	-0.85±1.78	-1.80±3.77	-5.05±4.00	-2.13±2.87	-1.13±2.21	-0.92±2.91

31	-0.52±1.47	-0.36±1.48	-0.20±0.86	-0.19±1.34	-7.98±4.51	-3.95±3.12	-1.86±2.26	-2.58±4.17
32	-0.26±0.79	-1.26±2.51	-0.86±1.71	-0.79±2.06	-5.39±3.61	-1.97±2.17	-1.44±2.48	-1.82±3.88
33	-0.51±1.22	-0.90±2.19	-0.62±1.49	-0.80±2.33	-5.01±5.13	-2.30±2.55	-0.96±1.73	-0.80±2.48
34	-0.18±0.60	-0.08±0.62	-0.13±0.71	-0.29±1.56	-3.97±4.16	-2.22±2.95	-1.78±3.17	-1.51±3.46
35	-0.72±1.71	-0.34±1.22	-0.43±1.06	-0.74±4.39	-4.68±5.08	-2.21±2.56	-1.84±2.42	-2.26±4.78

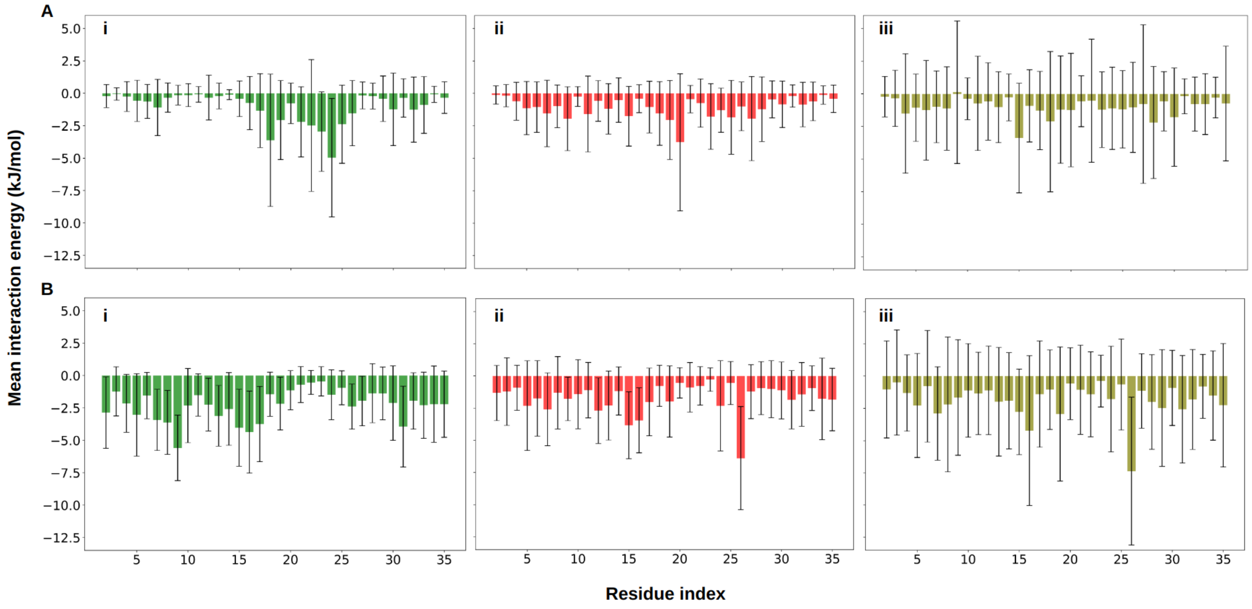

Figure S12: Non-bonded interaction energies between Nile red molecules and different residues of **(A)** *endo*-β-Gal PGP (i) PEG, (ii) Triazole, and (iii) Galactose moiety, and sidechains, and **(B)** *exo*-β-Gal PGP (i) PEG, (ii) Triazole, and (iii) Galactose moiety. The high error bar appears due to an averaging over 290 ns and three independent runs.

(a) (b)

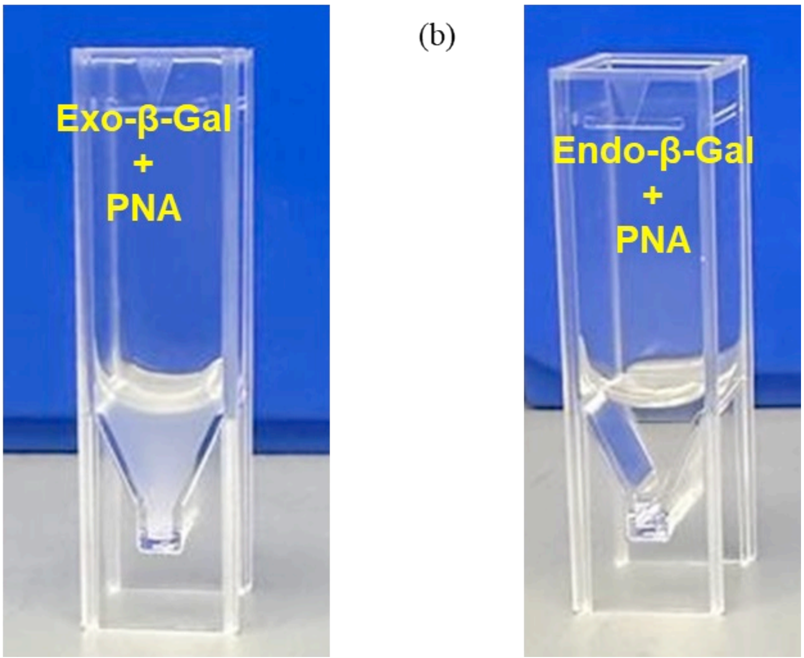

Figure S13: Lectin binding interaction of galactose PGPs with PNA; (a) *exo*-β-Gal with PNA and (b) *endo*-β-Gal with PNA.

Table S8: Dynamic light scattering (DLS) data of both *exo*- and *endo*-β-Gal PGPs with *peanut agglutinin* (PNA) and Galectin-3 (Gal-3) represents both number (%) and volume (%).

Samples	% Number	% Volume
Exo-β-Gal	6.11 ± 0.23	6.12 ± 0.26
Endo-β-Gal	7.30 ± 0.42	7.50 ± 0.46
PNA	6.64 ± 0.15	7.35 ± 0.53
Galectin-3	19.76 ± 2.44	19.07 ± 1.33

- SE: standard error values

Table S9: Microscale thermophoresis (MST) to evaluate the binding affinities (K_d) between both *exo*- and *endo*-β-Gal PGPs with *peanut agglutinin* (PNA).

PGPs	Lectin	K_d
exo-β-Gal	PNA	1.54 μM
endo-β-Gal	PNA	1.50 mM

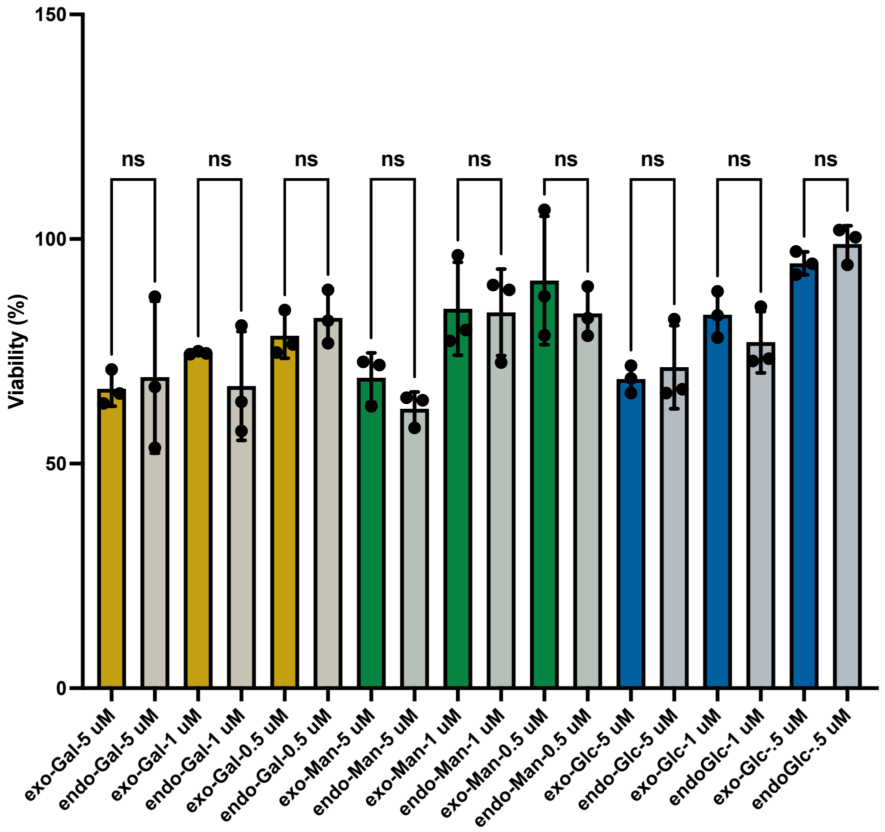

Figure S14: CCK8 Assay for cytotoxicity of both exo- and endo-PGPs: *endo*-β-Gal, *exo*-β-Gal, *endo*-β-Glc, *exo*-β-Glc, *endo*-α-Man, and *exo*-α-Man, at different studied concentrations (5.0 μM, 1.0 μM and 0.5 μM). Experiments were performed in biological triplicate, and statistical analysis was performed using an ordinary one-way ANOVA, where "ns" represents a P value of >0.05.

Table S10: Flow cytometry data of cellular uptake (%) of PGPs by 4T1 cell line at 37 °C.

PGPs	Cell Lines	Uptake (%)
exo-β-Gal	4T1	76.6 + 3.46
endo-β-Gal	4T1	91.1 + 0.74
exo-β-Glc	4T1	67.5 + 3.85
endo-β-Glc	4T1	78.4 + 1.08
exo-α-Man	4T1	42.9 + 0.68
endo-α-Man	4T1	90.7 + 0.87

Table S11: Flow cytometry data of cellular uptake (%) of PGPs by 4T1 cell line at 4 °C.

PGPs	Cell Lines	Uptake (%)
exo-β-Gal	4T1	3.87 + 0.55
endo-β-Gal	4T1	7.10 + 0.36
exo-β-Glc	4T1	8.70 + 1.94
endo-β-Glc	4T1	30.60 + 6.99
exo-α-Man	4T1	1.17 + 0.12
endo-α-Man	4T1	22.30 + 1.97

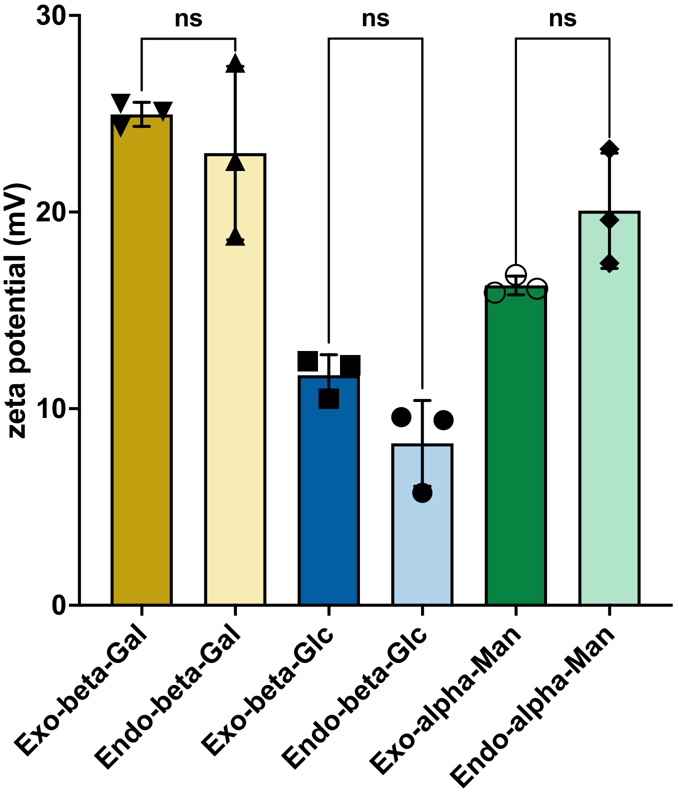

Figure S15: Zeta potential of Exo-β-Glc (cark blue), Endo-β-Glc (light blue), Exo-β-Gal (dark yellow), Endo-β-Gal (light yellow), Exo-α-Man (dark green) and Endo-α-Man (light green). All samples were analyzed in DI water at pH = 7. Error bars represent the standard deviation of the mean of triplicate samples. Statistical analysis was performed using an ordinary one-way ANOVA, where "ns" represents a P value of >0.05. Experiments were performed in triplicate, and statistical analysis was performed using an ordinary one-way ANOVA, where "ns" represents a P value of >0.05.

Fluorescence Inhibition Assay using (Cholera Toxin X (Ctx) Subunit)-FITC Conjugate: The fluorescence inhibition assay was performed as previously reported.[14,15] Briefly, stock solutions of galactose-containing glycopolymers *(i.e.*, *exo*-β-Gal and *endo*-β-Gal) were

prepared. To this end, 9.11 mg samples of each *exo*- and *endo*-PGP were individually dissolved in 369 µL of HEPES buffer, resulting in an 18.75 mM galactose-equivalent concentration. The dilution series were prepared accordingly:

Table S12: Series dilution for inhibition assay

Solution	Concentration w.r.t Galactose in solution	Concentration w.r.t to Galactose in assay	Volume for next dilution	Volume for dilution
**1**	18.75 mM	15 mM	738 µL - 1	0 µL
**2**	15 mM	12 mM	160 µL - 1	40 µL
**3**	11.3 mM	9 mM	120 µL - 1	80 µL
**4**	7.53 mM	6 mM	80 µL - 1	120 µL
**5**	3.77 mM	3 mM	80 µL - 1	320 µL
**6**	1.88 mM	1.5 mM	200 µL - 5	200 µL
**7**	942 µM	750 µM	200 µL - 6	200 µL
**8**	471 µM	375 µM	200 µL - 7	200 µL
**9**	235 µM	188 µM	200 µL - 8	200 µL
**10**	118 µM	94 µM	200 µL - 9	200 µL
**11**	58.8 µM	47 µM	200 µL - 10	200 µL
**12**	29.4 µM	23.5 µM	200 µL - 11	200 µL

A 500 µg aliquot of Cholera Toxin x (Ctx) subunit FITC conjugate was dissolved in 8 mL HEPES buffer (0.1 M, pH = 7.4) to give a 62.5 µg/mL stock solution.

At least 10 mL of a 1.0 g/L solution of GM1 ganglioside in PBS (0.1 M, pH = 7.4) was prepared, and 100 µL of this GM1 solution was added to each well of a Maxisorb 96-well, High Binding, flat bottom, flat black plate, and placed on a shaker overnight. After shaking overnight, each well was decanted and rinsed with 3 x 100 µL portions PBS. Next, at least 40 mL of a 1 wt.% solution of bovine serum albumin in PBS was prepared, and 100 µL of this solution was added

to each well. The plate was placed back on a shaker for 1 hour, and then each well was decanted and washed with 2 x 100 μL portion PBS. To a 400 μL aliquot of each glycopolymer, 100 μL of the 62.5 μg/mL Ctx stock was added. Each aliquot was then covered with foil and gently shaken on a shaker plate for 2 hours. After shaking, 100 μL of each polymer dilution + Ctx stock was distributed into the desired wells of the Maxisorb 96 plate in triplicate and the plate was incubated at 37 °C for 30 minutes. After incubation, the plate was decanted, and each well was rinsed with 3 x 100 μL portions HEPES buffer. The fluorescence was quantified immediately on a Biotek Synergy H1 microplate reader with excitation at 485 nm, emission at 528 nm. The fluorescence value was converted to a Ctx concentration from a standard curve, and inhibition was determined as a percentage relative to the starting Ctx concentration.

^{1}H-NMR and ^{13}C-NMR of Compound 1 (Exo-OH)

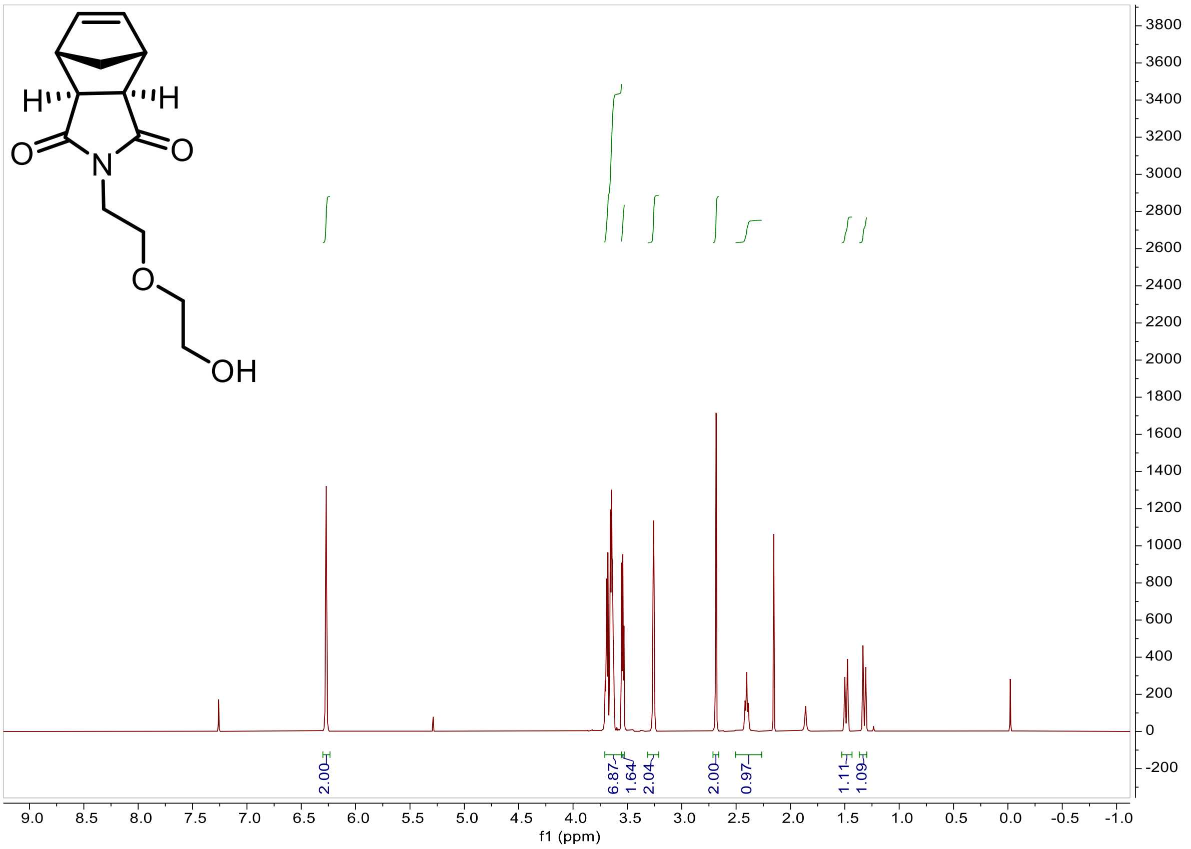

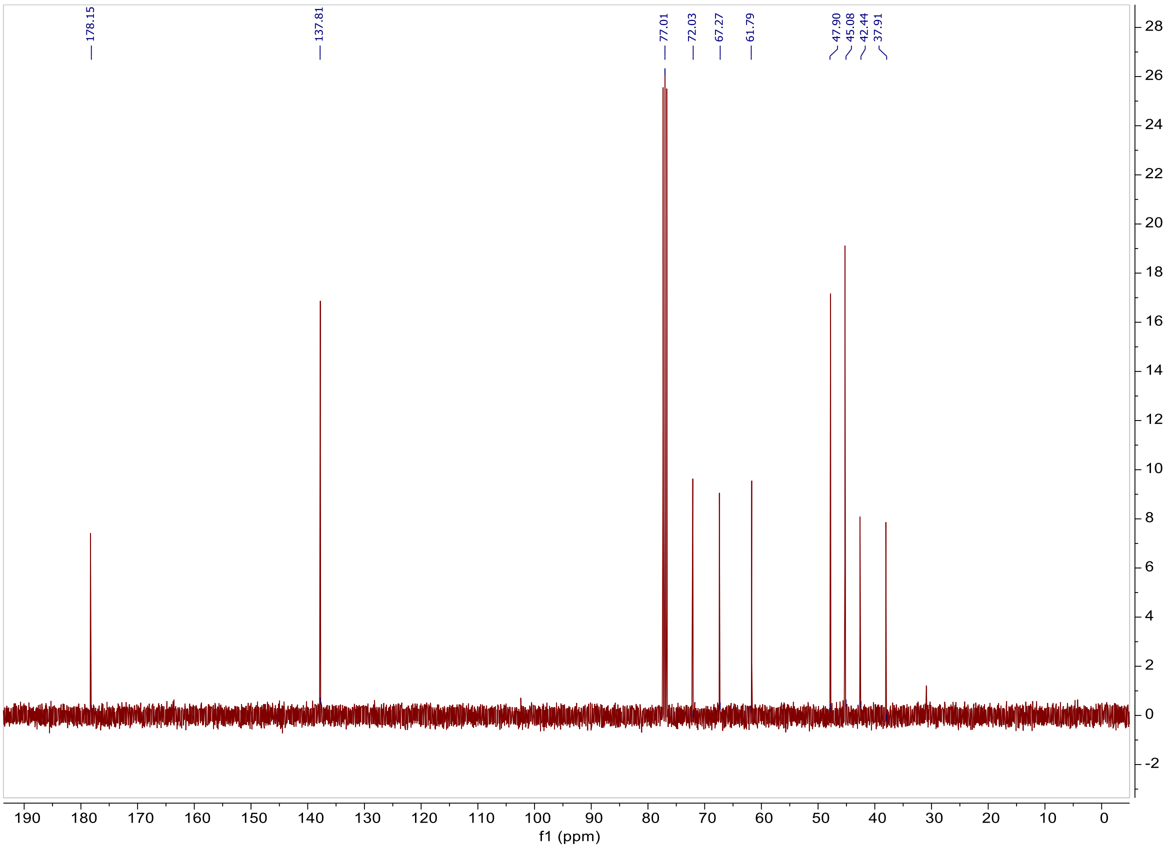

^{1}H-NMR and ^{13}C-NMR of Compound 2 (Endo-OH)

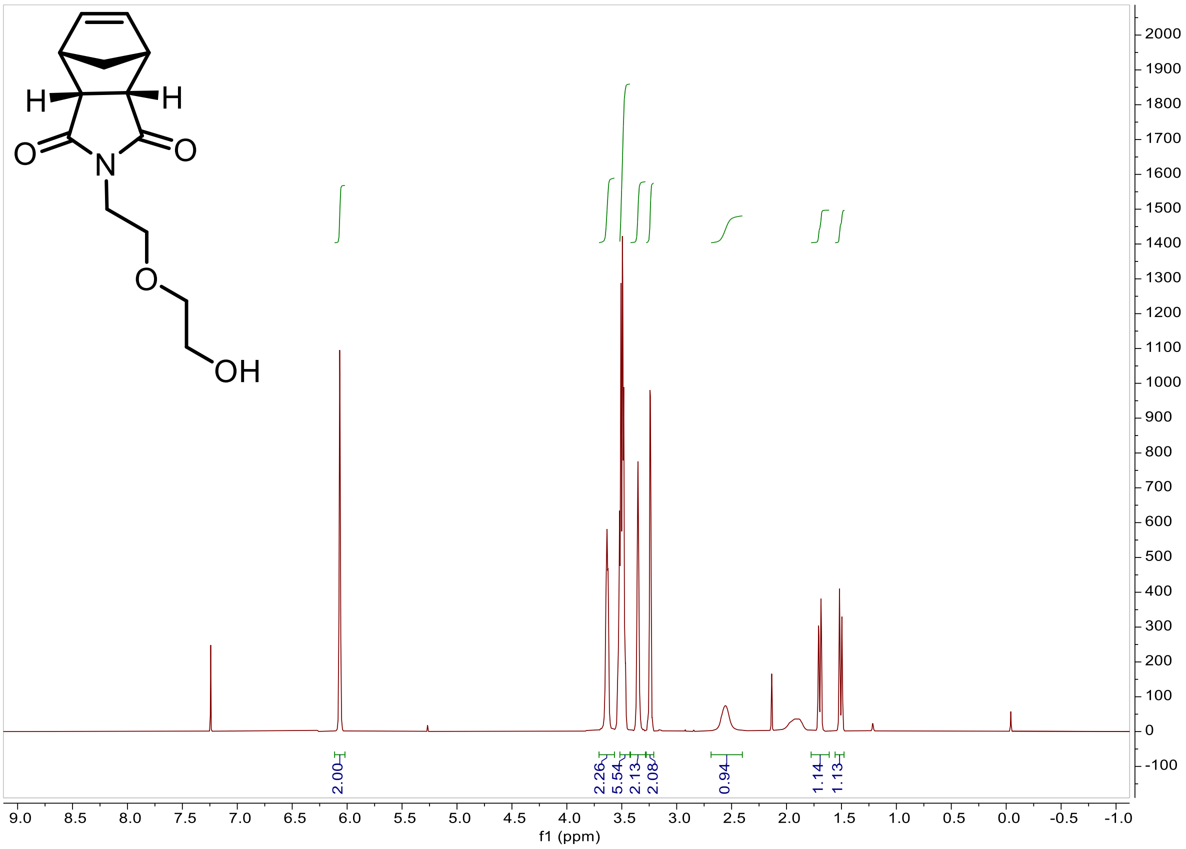

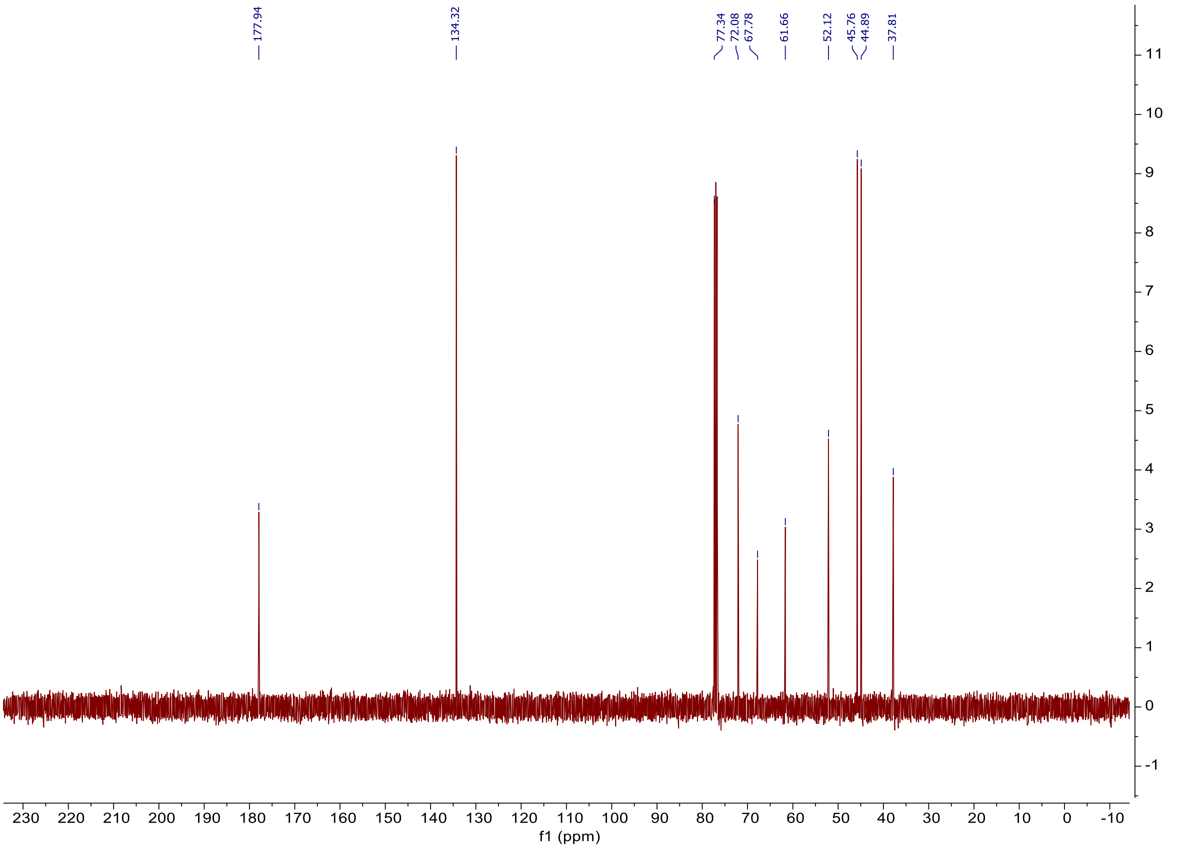

^{1}H-NMR and ^{13}C-NMR of Compound 3 (Exo-Alk)

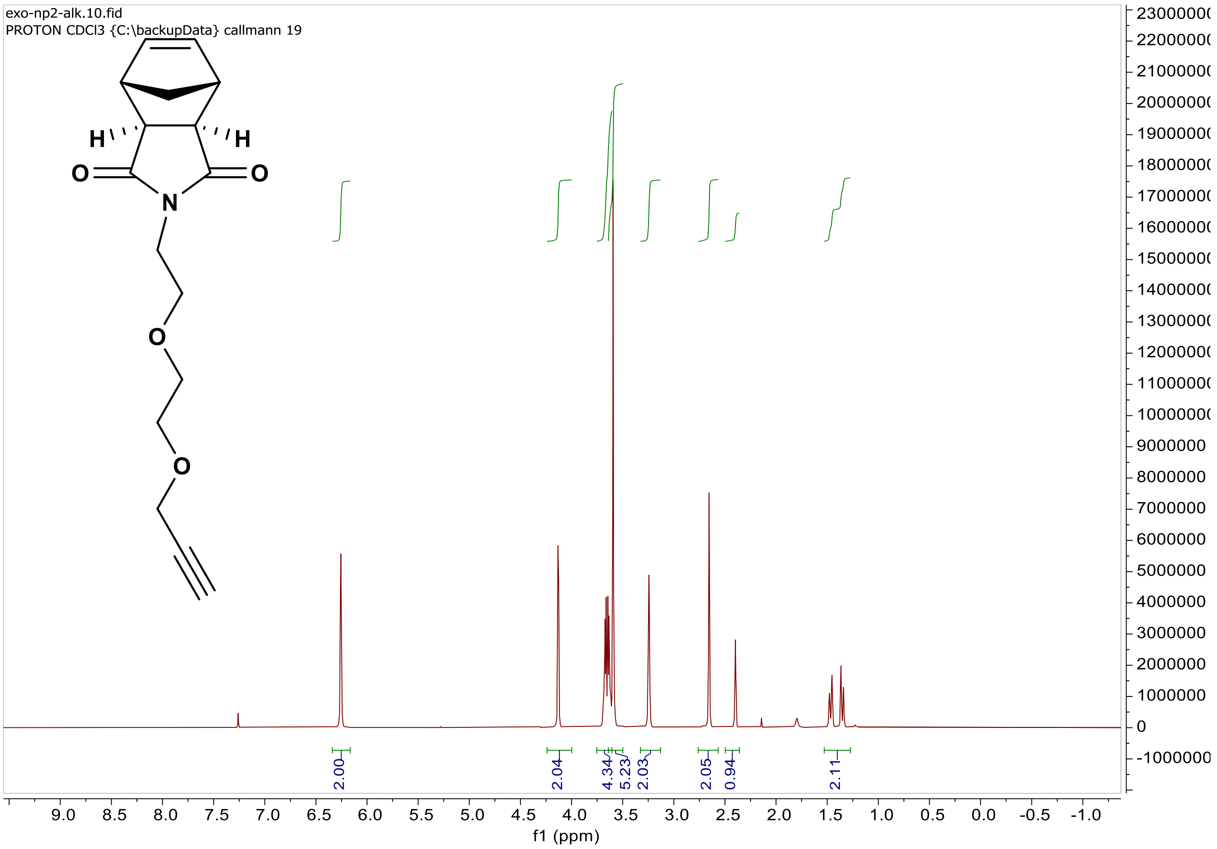

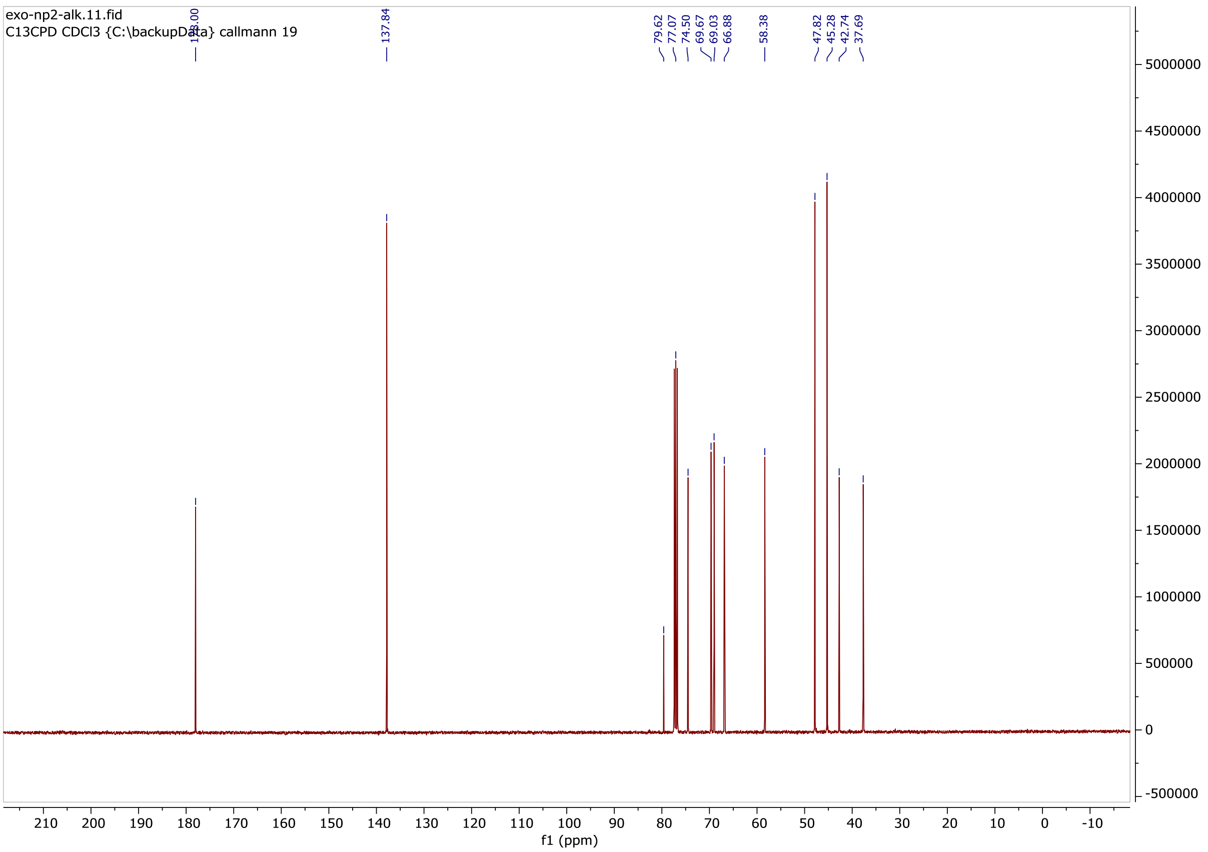

^{1}H-NMR and ^{13}C-NMR of Compound 4 (Endo-Alk)

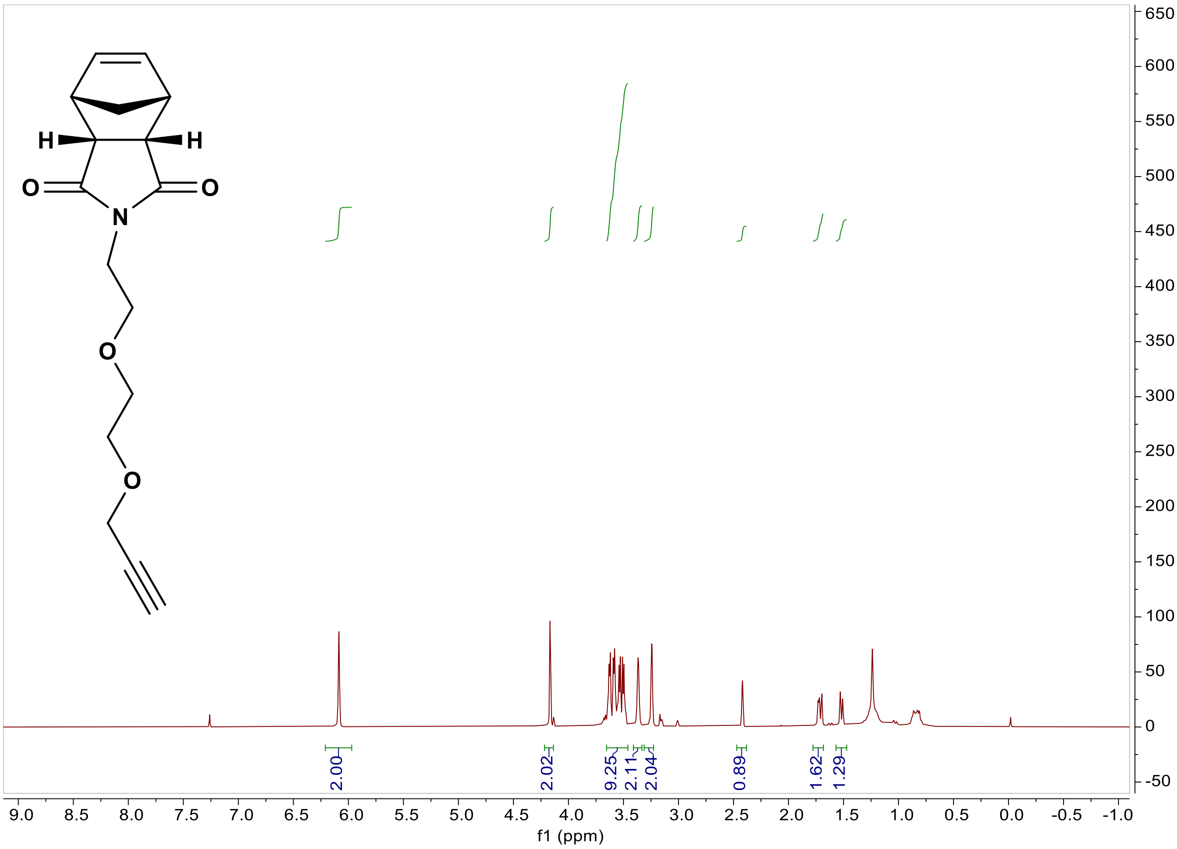

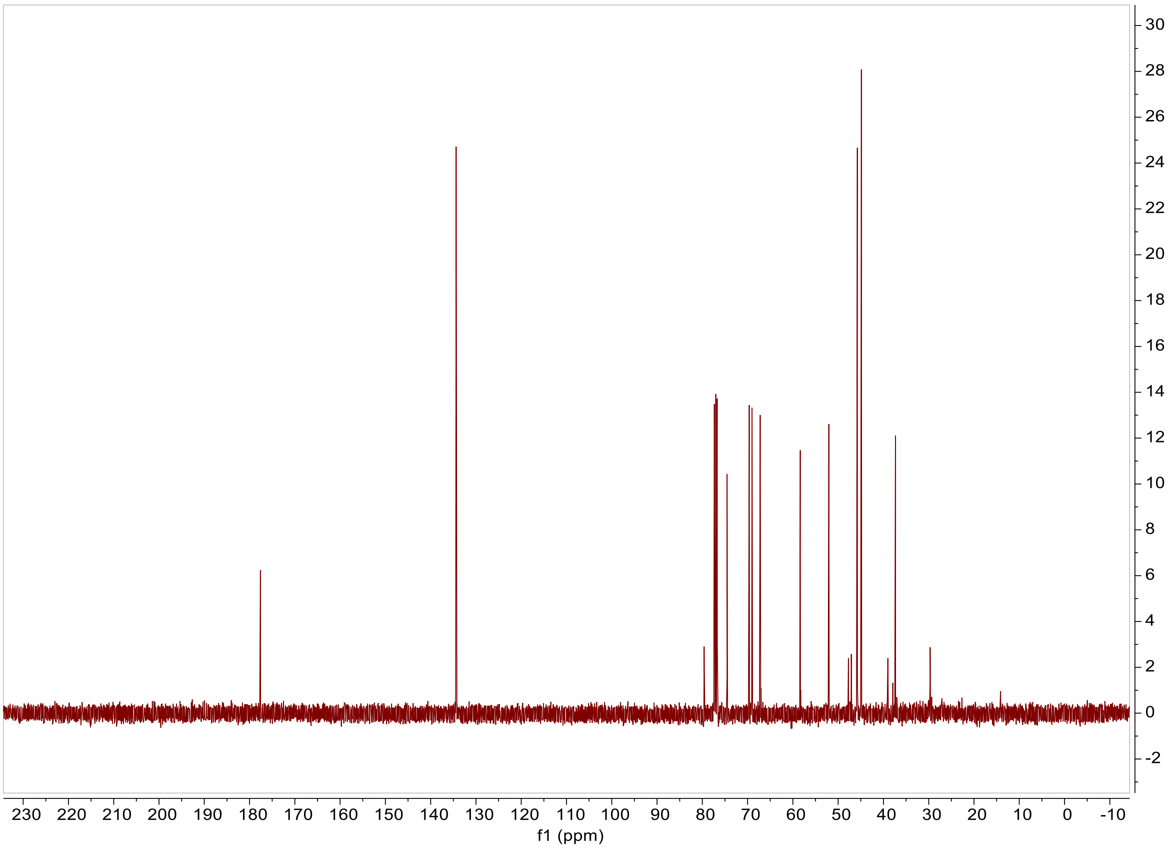

^{1}H-NMR of Compound 5 (Man-OAc$_5$)

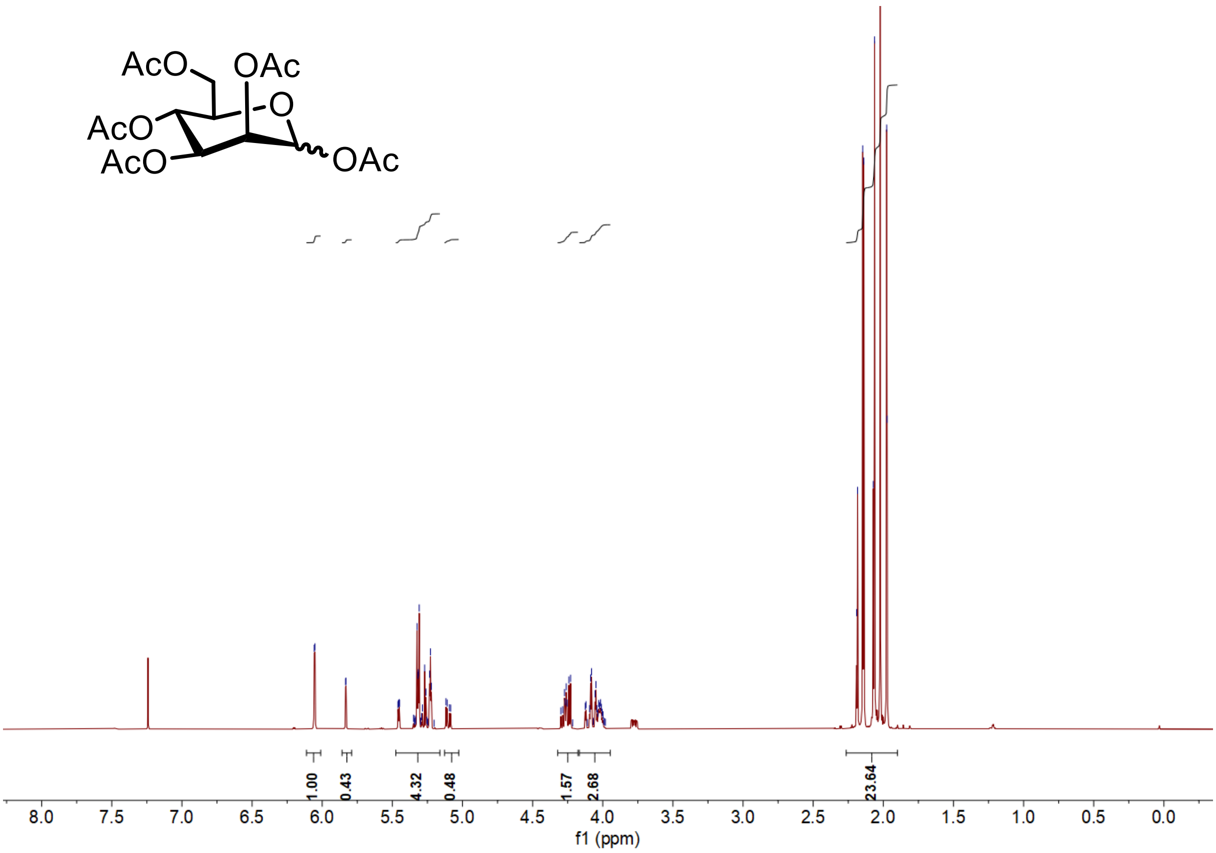

^{1}H-NMR of Compound 6 (Glc-OAc$_5$)

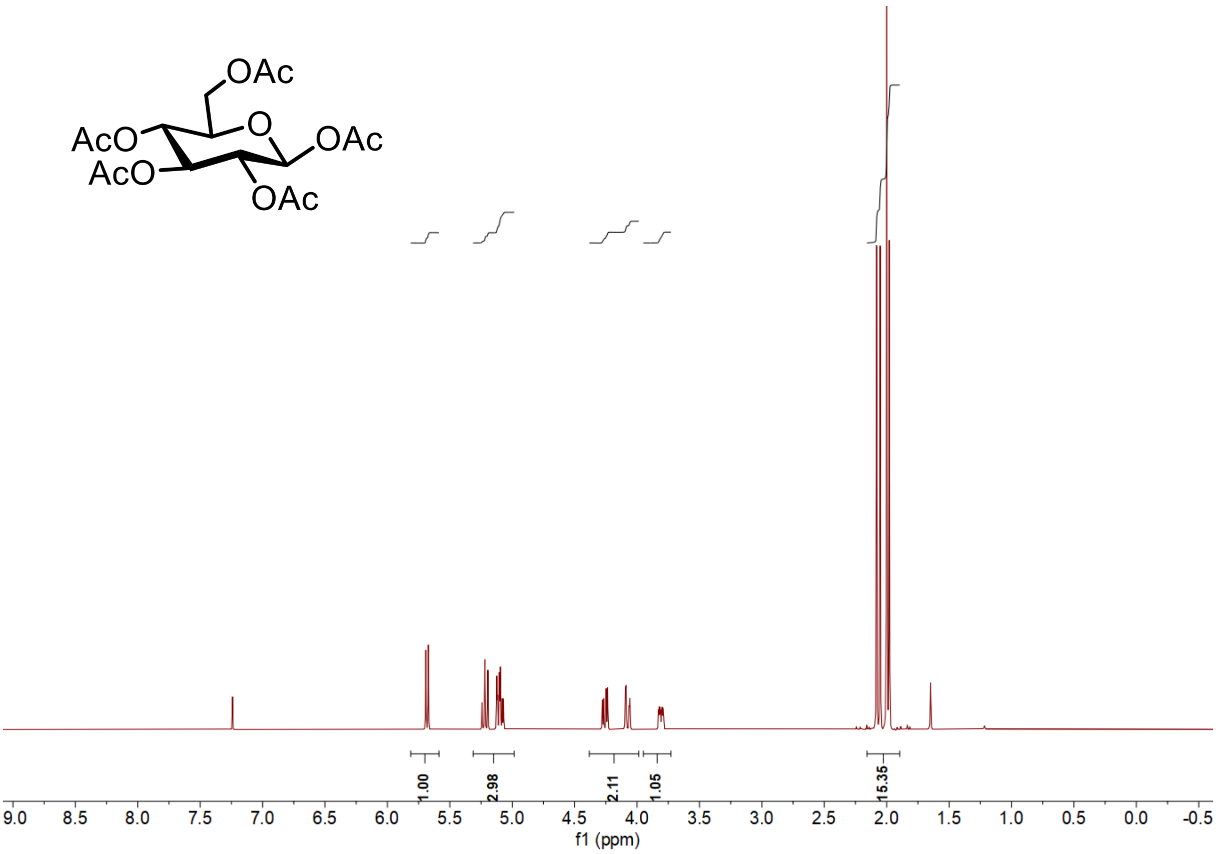

^{1}H-NMR of Compound 7 (Gal-OAc$_5$)

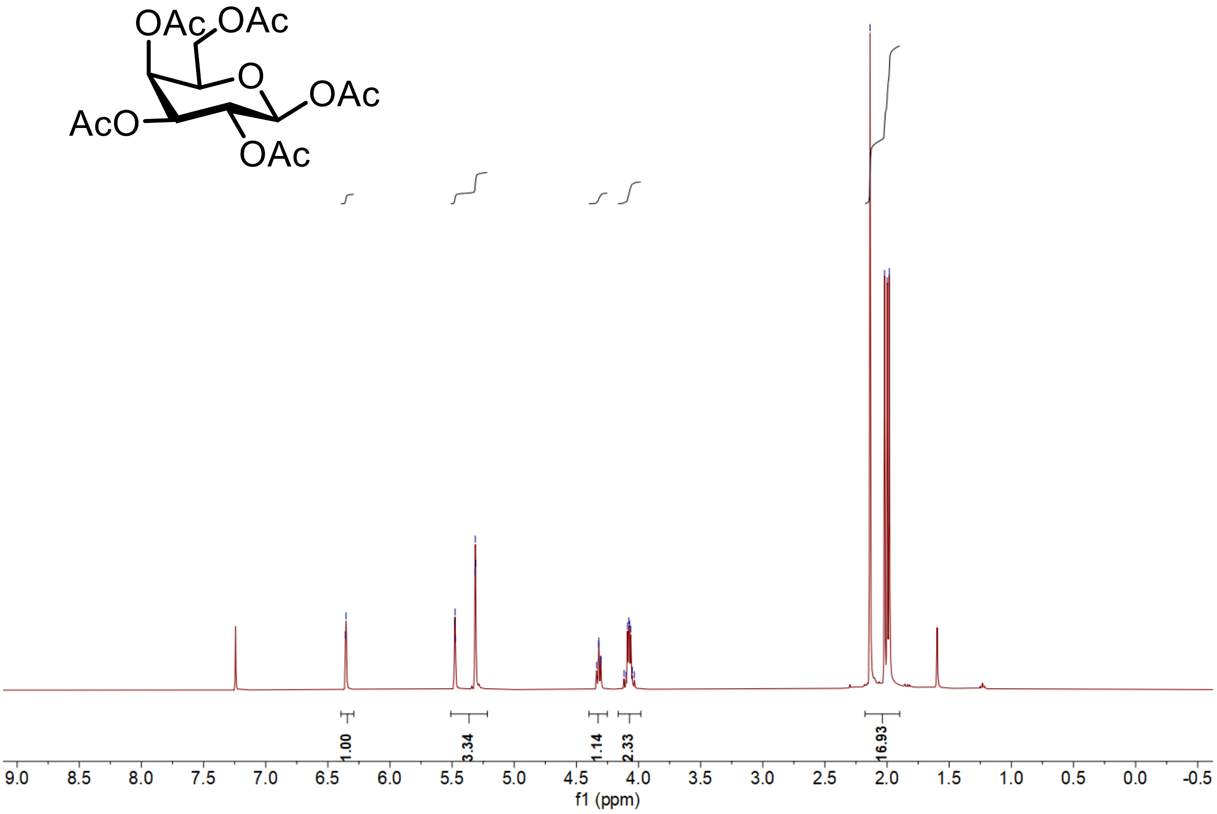

^{1}H-NMR of Compound 8 (Man-Azide)

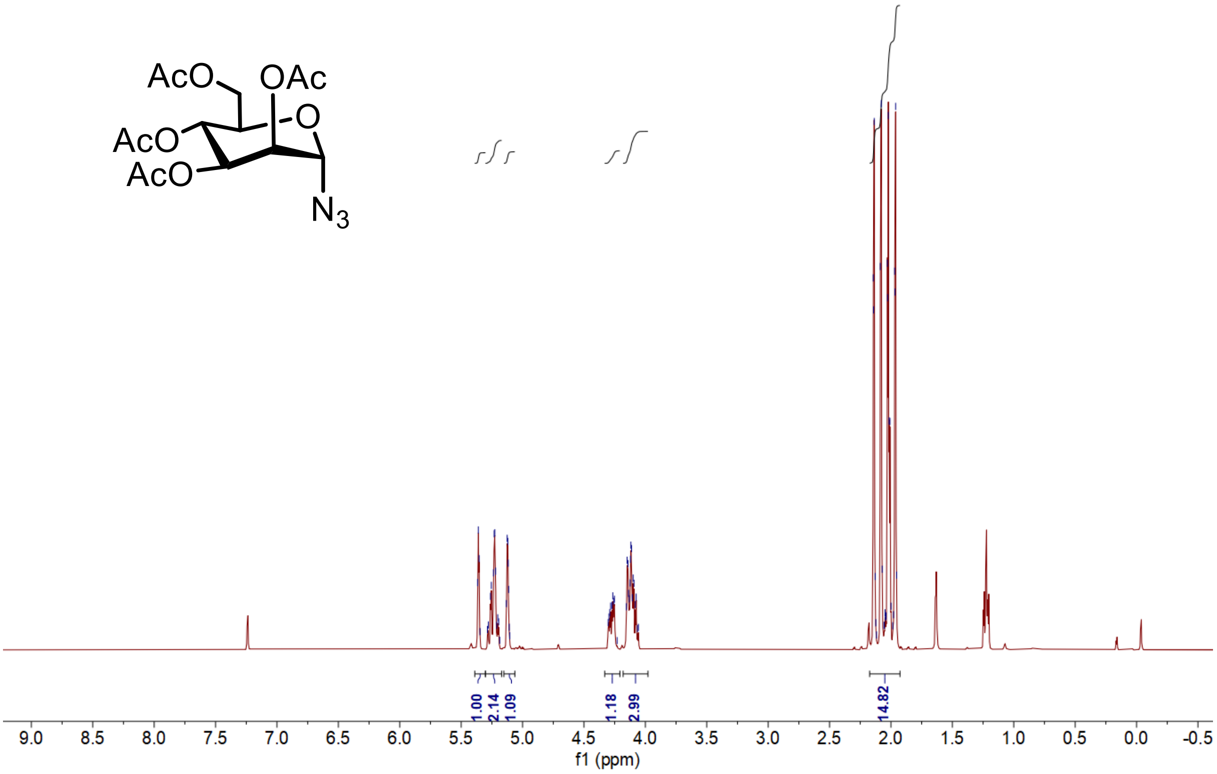

^{1}H-NMR of Compound 9 (Glc-Azide)

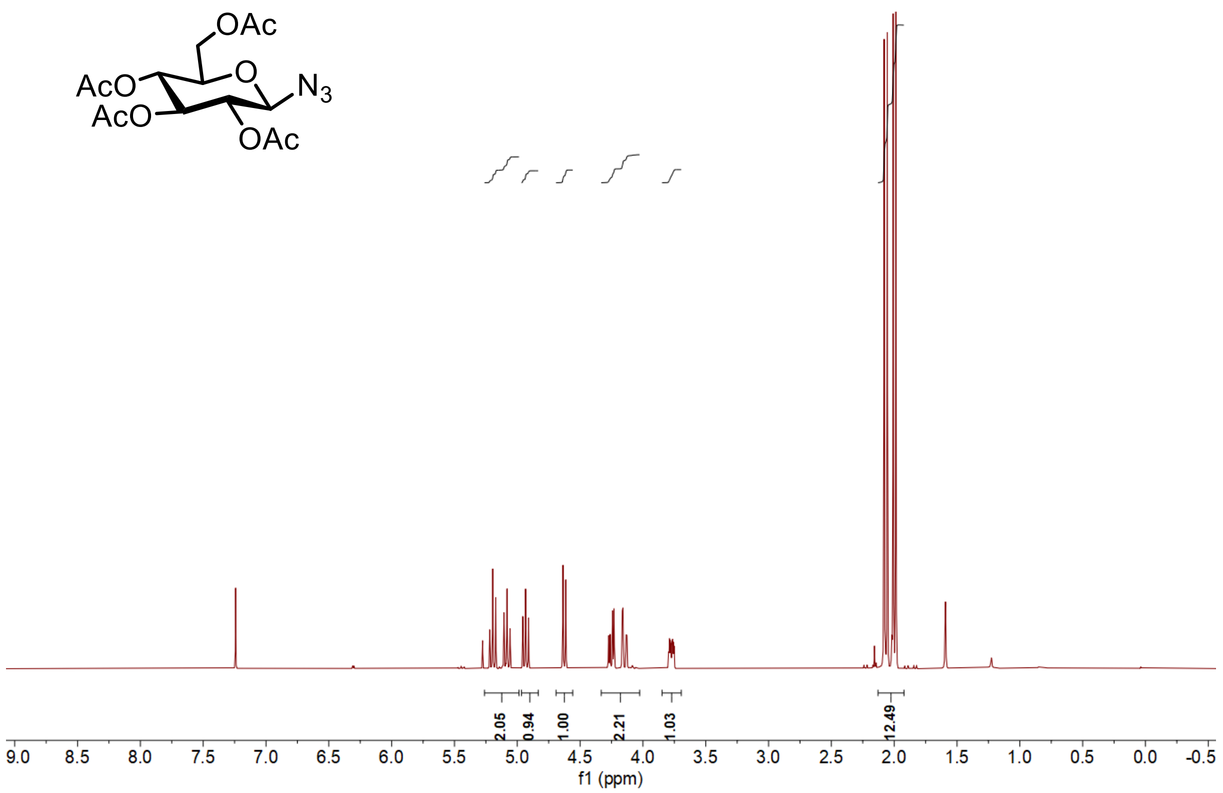

^{1}H-NMR of Compound 11 (Gal-Azide)

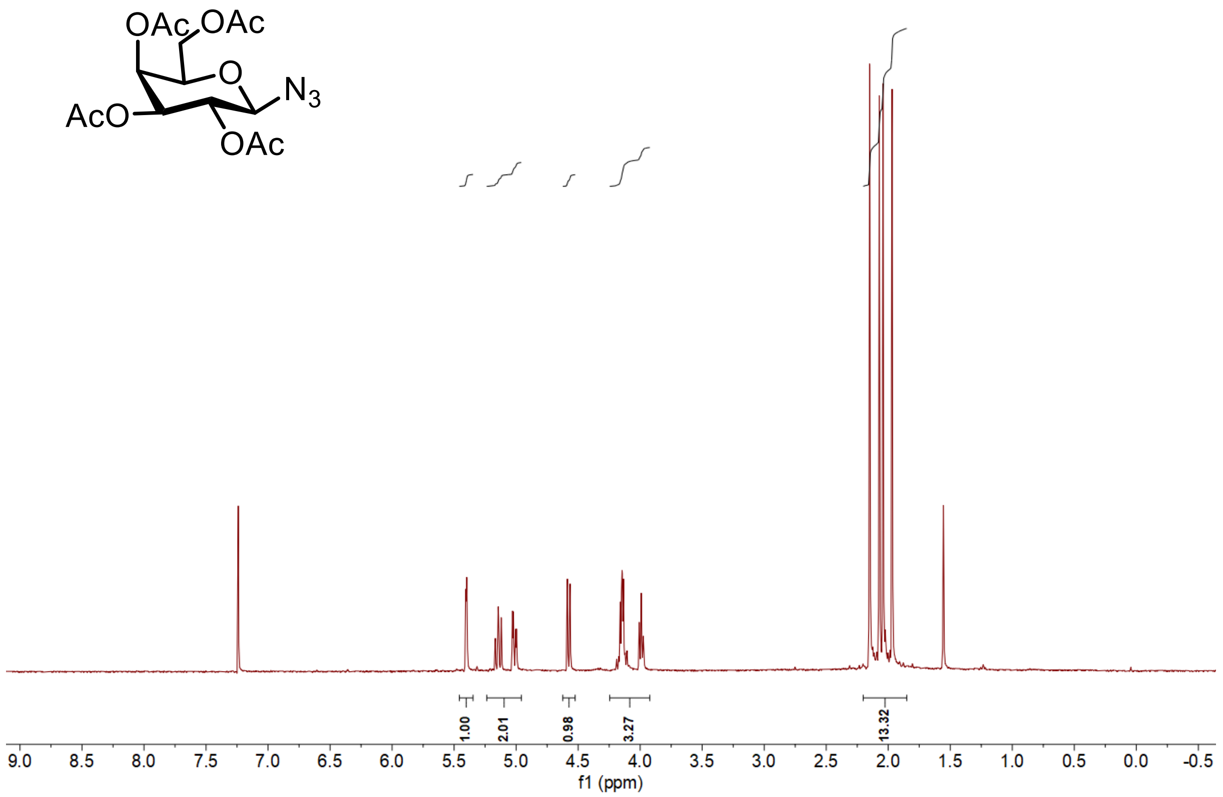

^{1}H-NMR, ^{13}C, COSY, HSQC of Compound 12 (α-Gal-Azide)

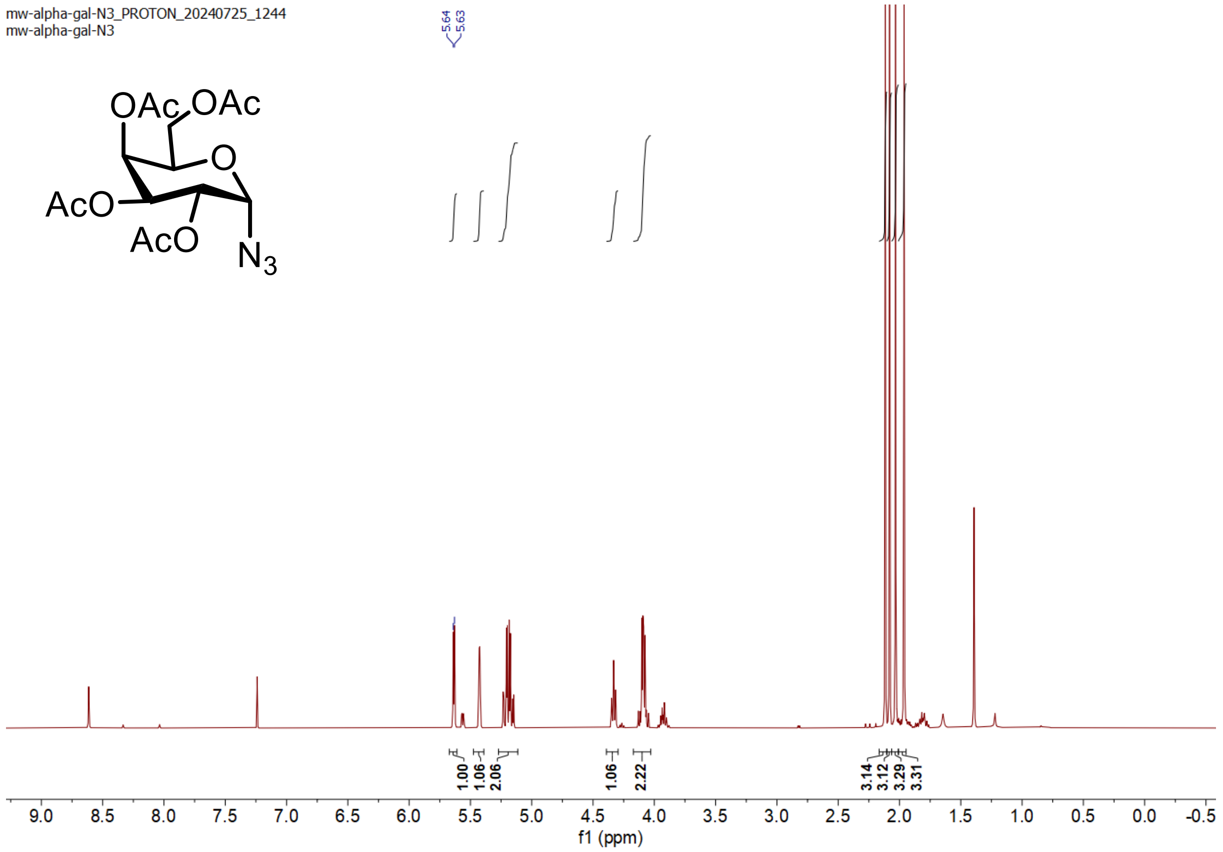

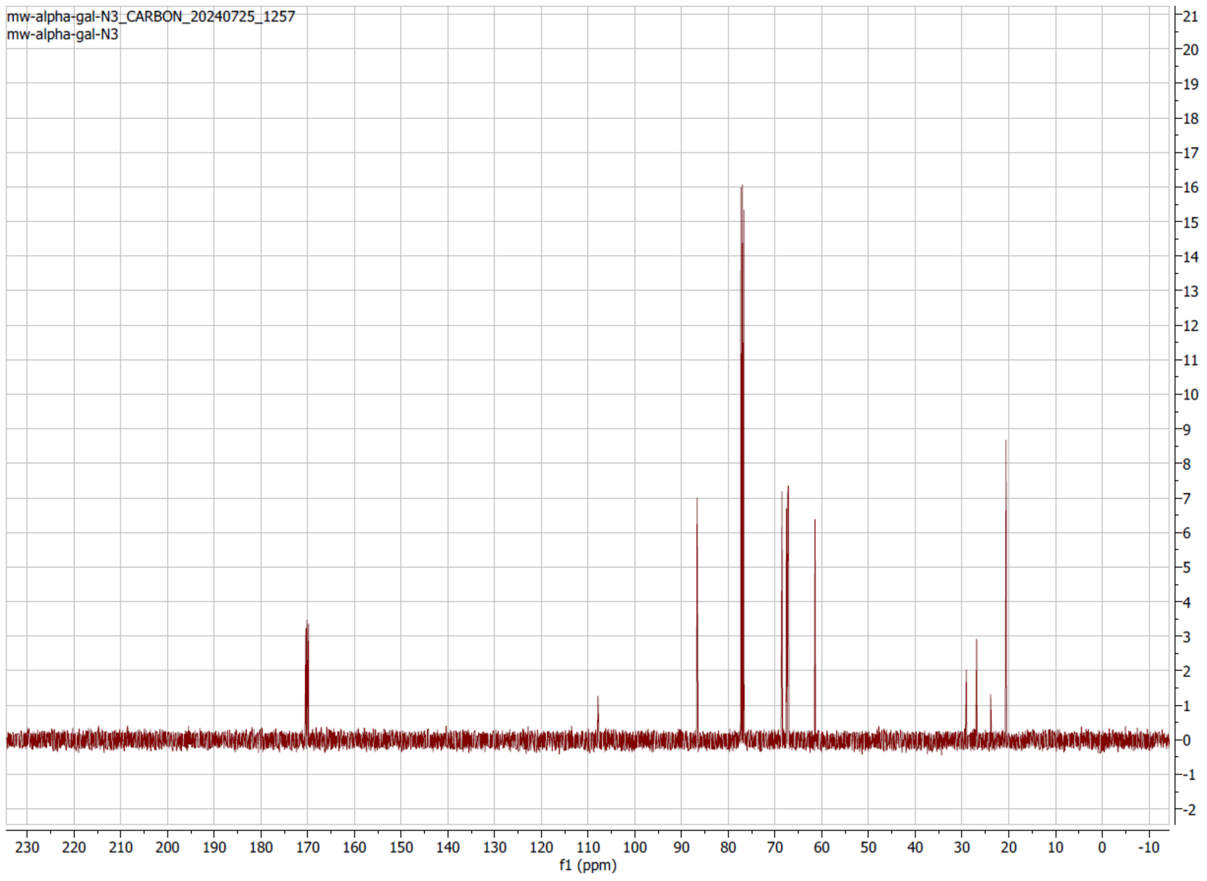

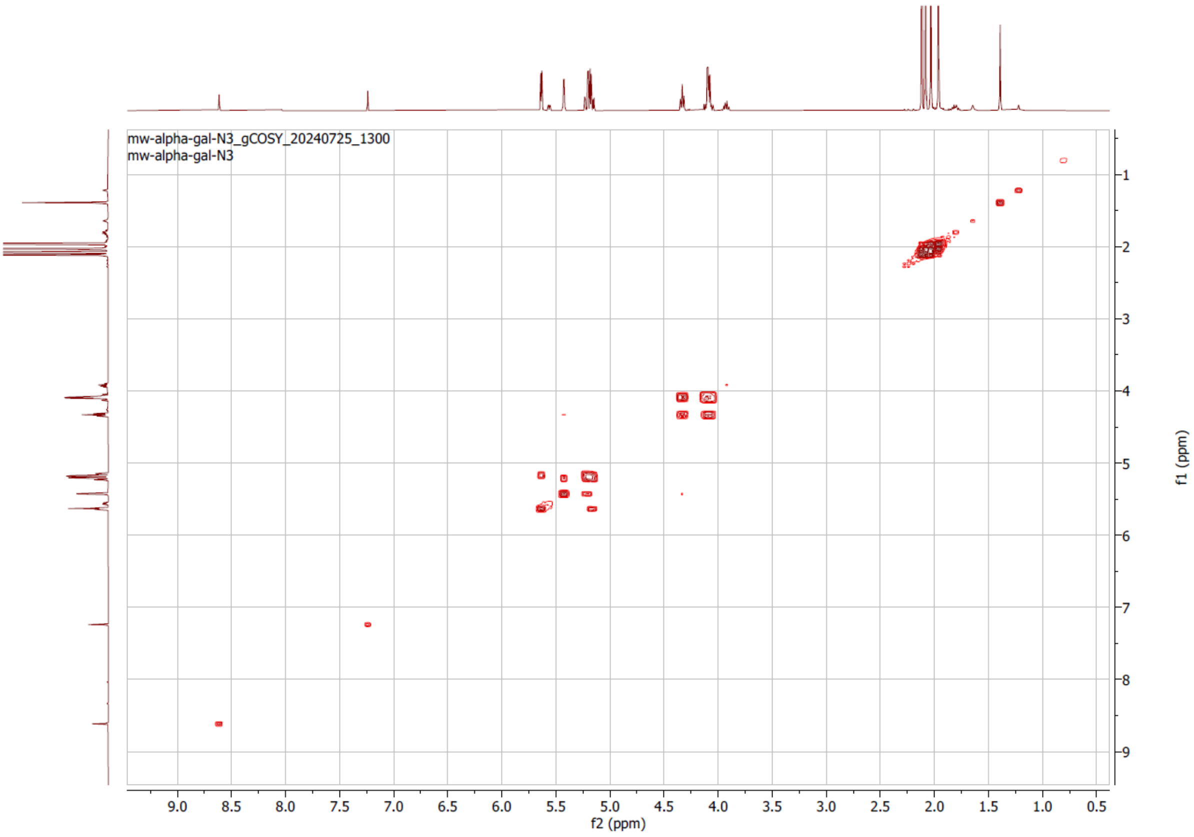
mw-alpha-gal-N3_gCOSY_20240725_1300
mw-alpha-gal-N3
f2 (ppm)
f1 (ppm)

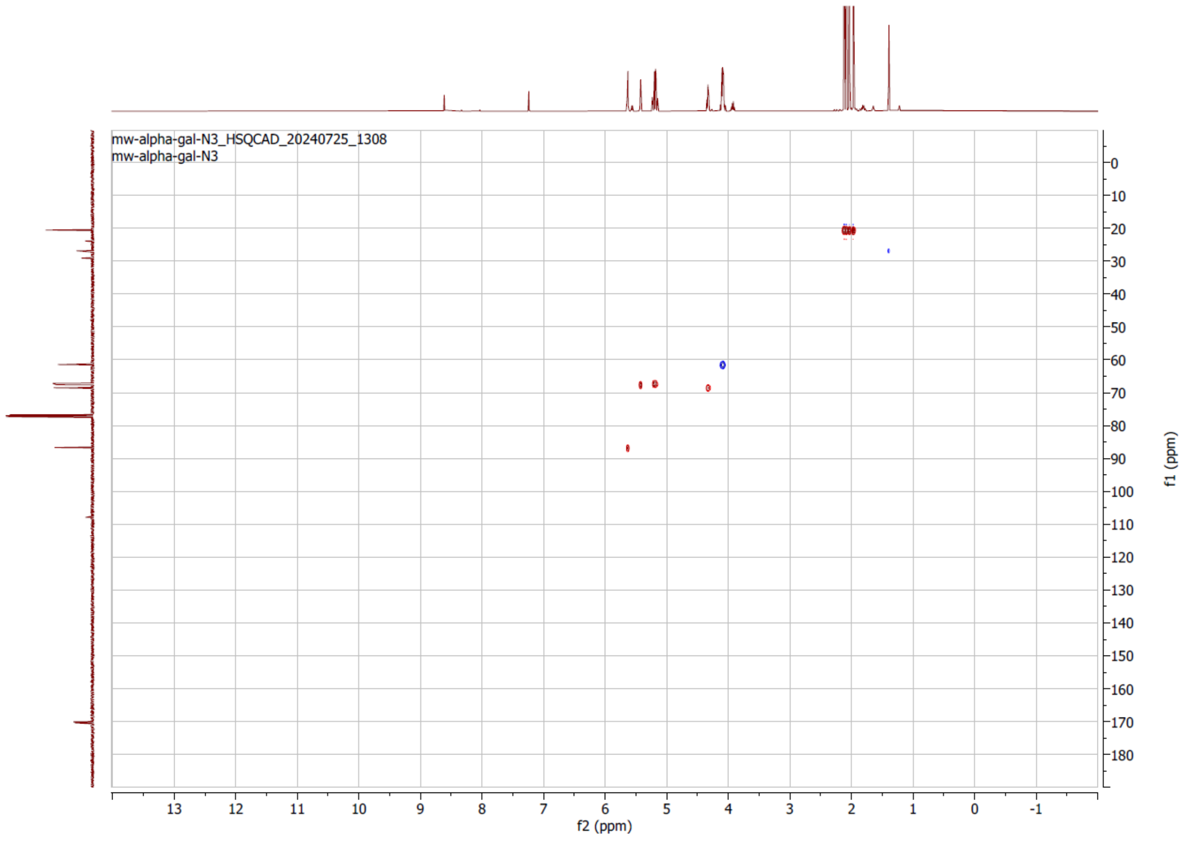
mw-alpha-gal-N3_HSQCAD_20240725_1308
mw-alpha-gal-N3
f2 (ppm)
f1 (ppm)

^{1}H-NMR, ^{13}C, COSY, HSQC of Compound 13 (α-Glc-Azide)

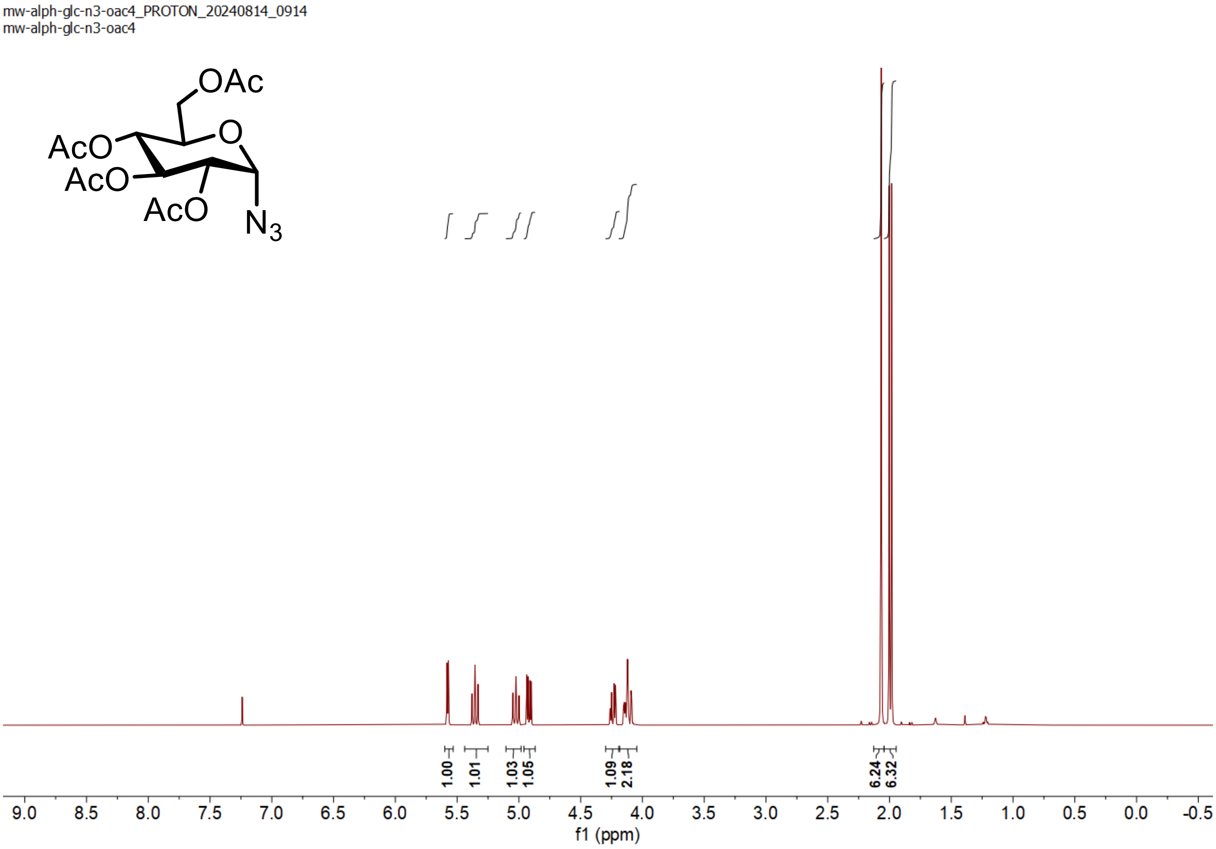

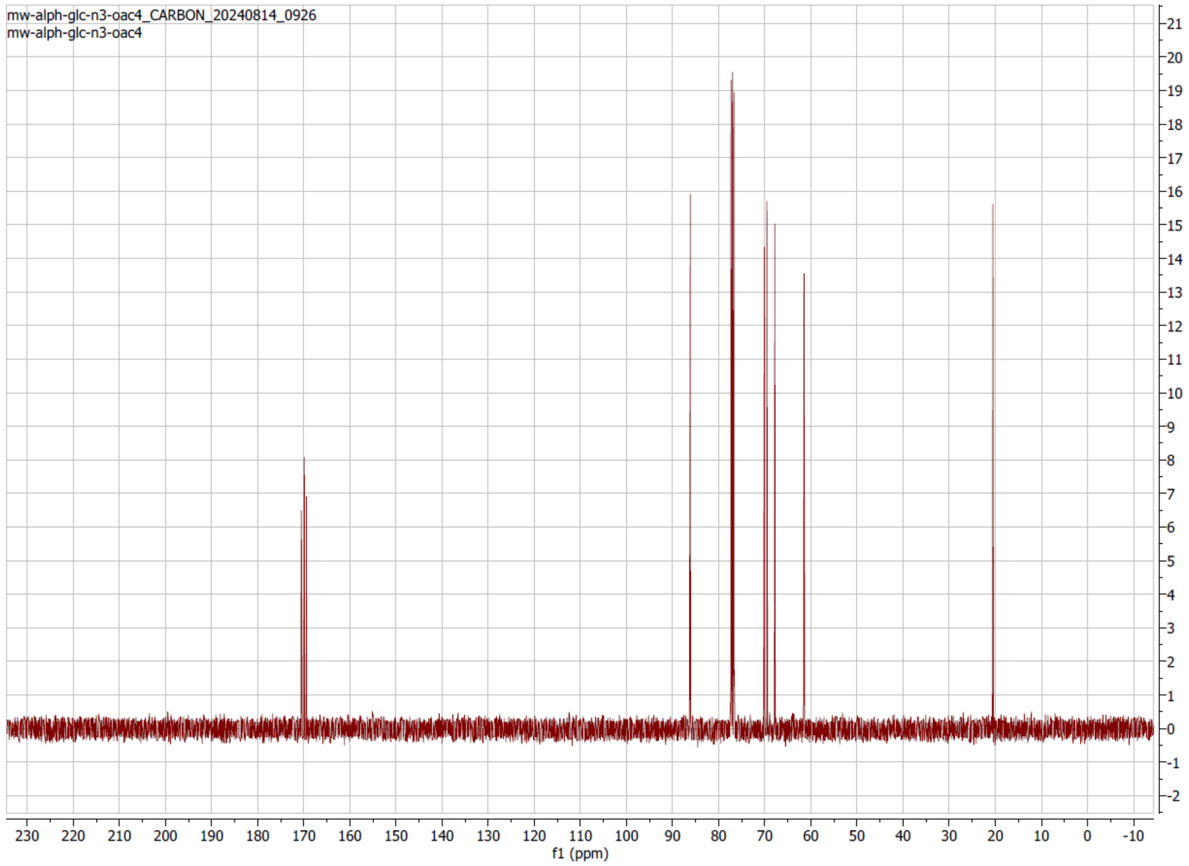

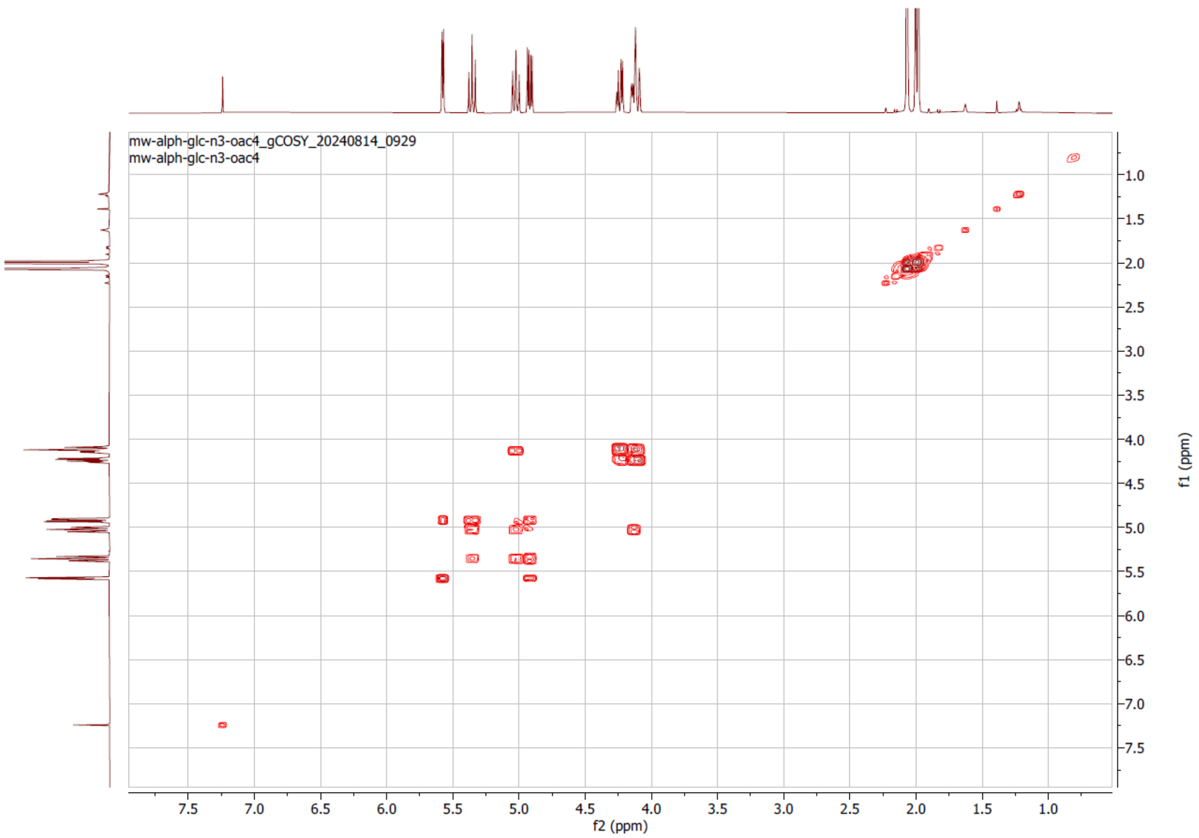
mw-alph-glc-n3-oac4_gCOSY_20240814_0929
mw-alph-glc-n3-oac4
f2 (ppm)
f1 (ppm)

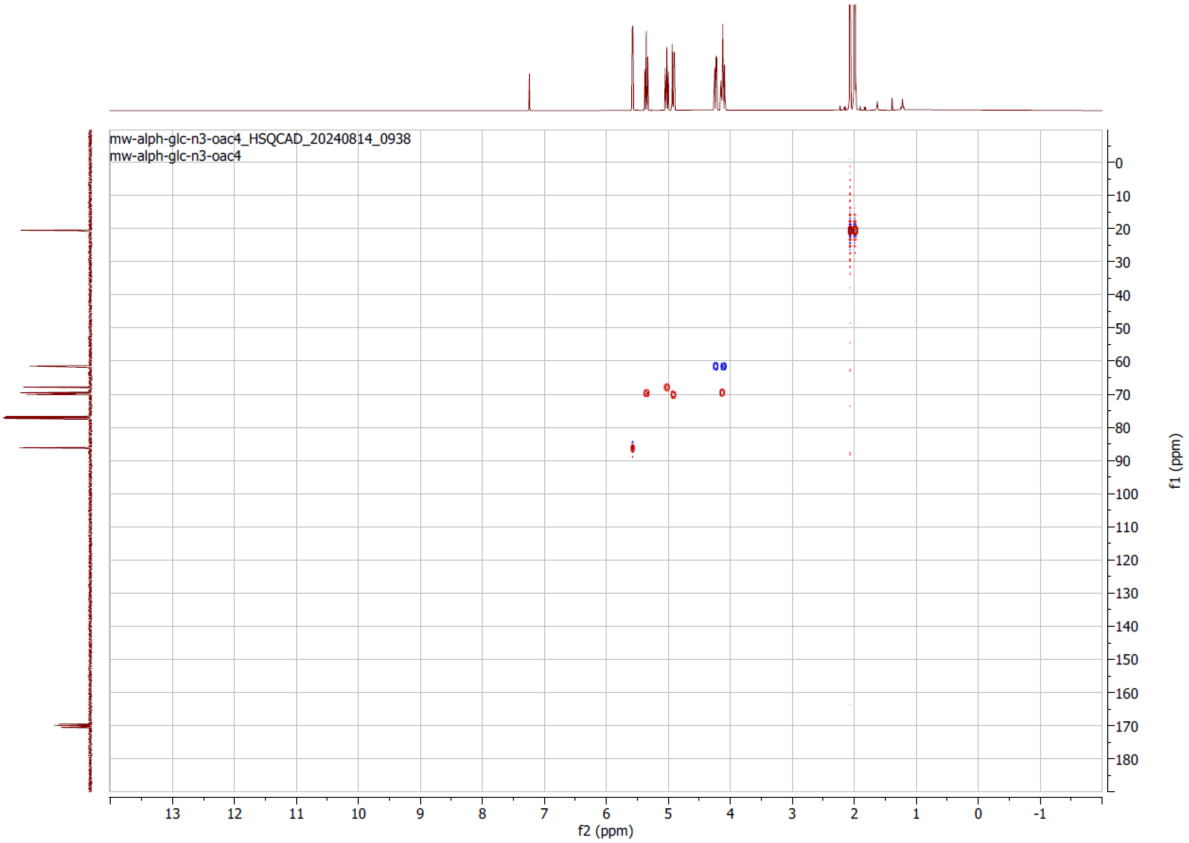
mw-alph-glc-n3-oac4_HSQCAD_20240814_0938
mw-alph-glc-n3-oac4
f2 (ppm)
f1 (ppm)

^{1}H-NMR, ^{13}C, COSY, HSQC of Compound *exo*-β-Gal-OAc$_4$

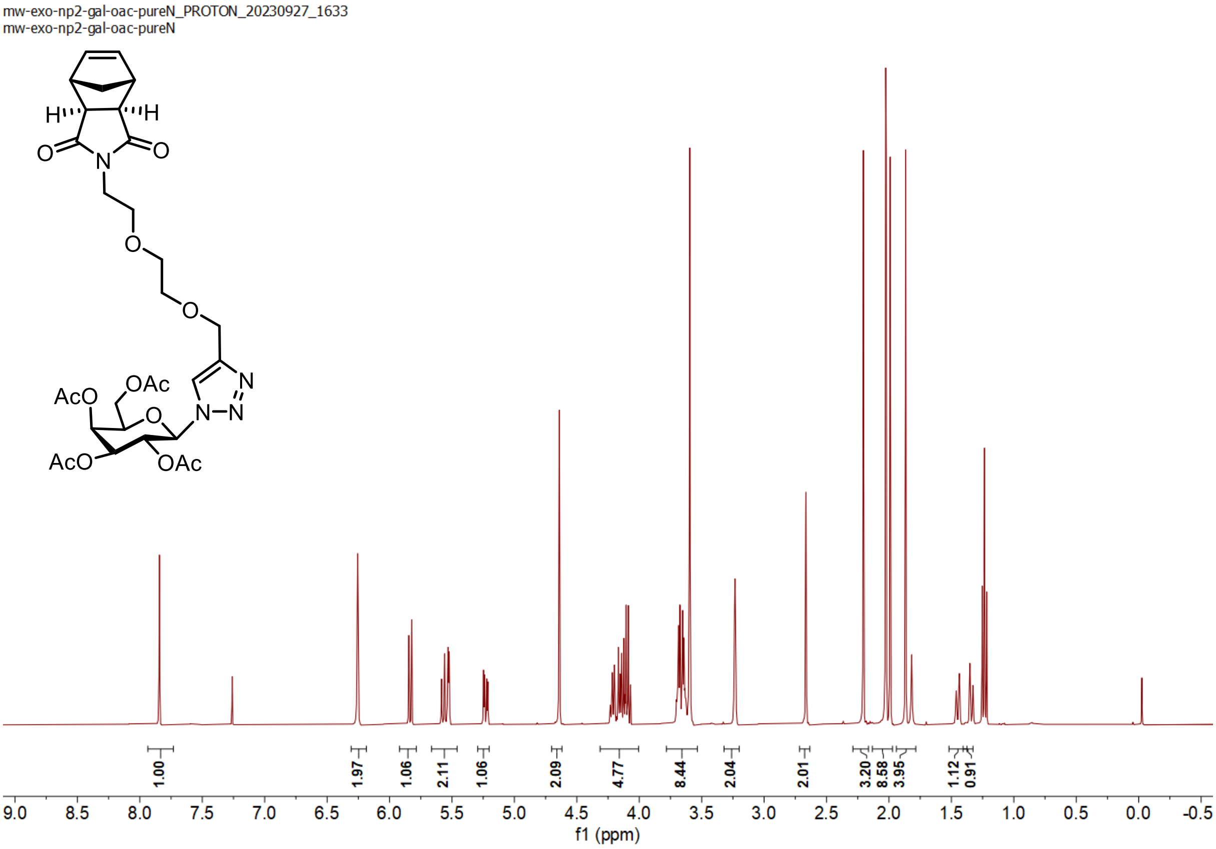

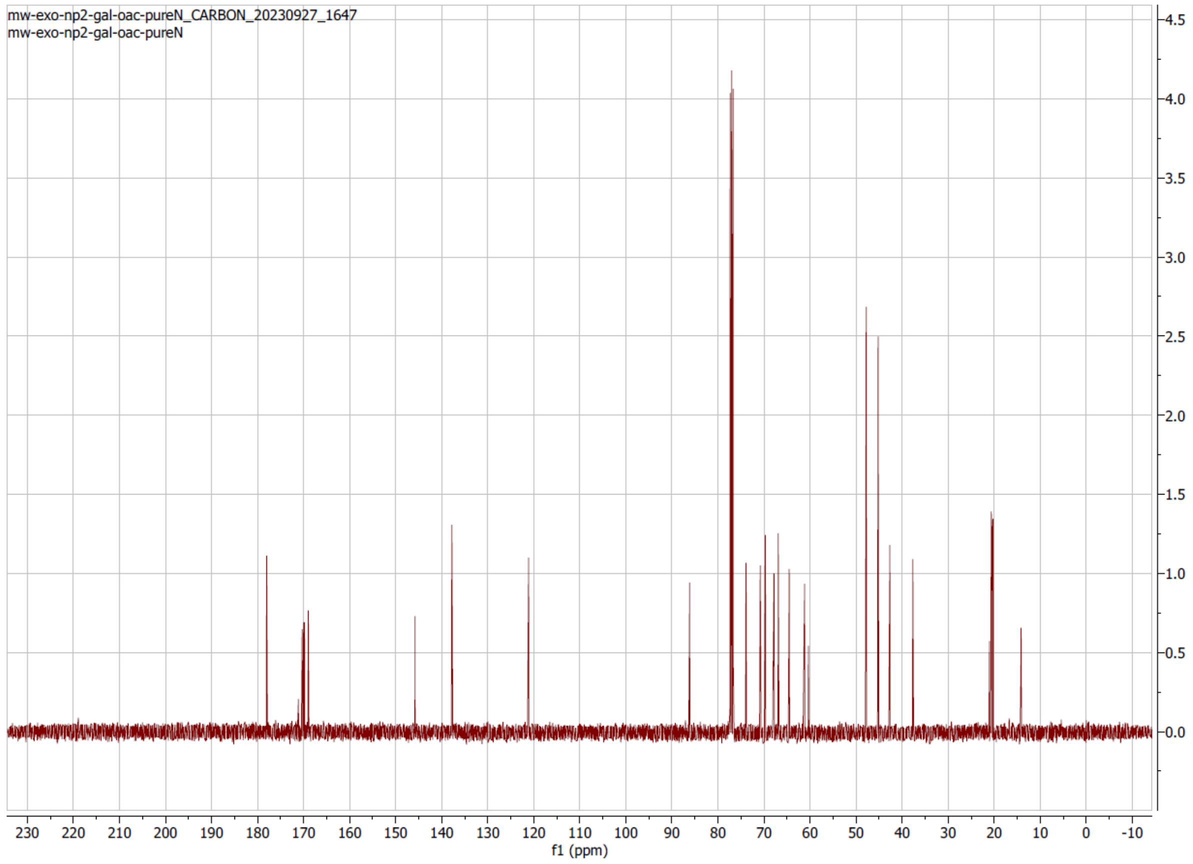

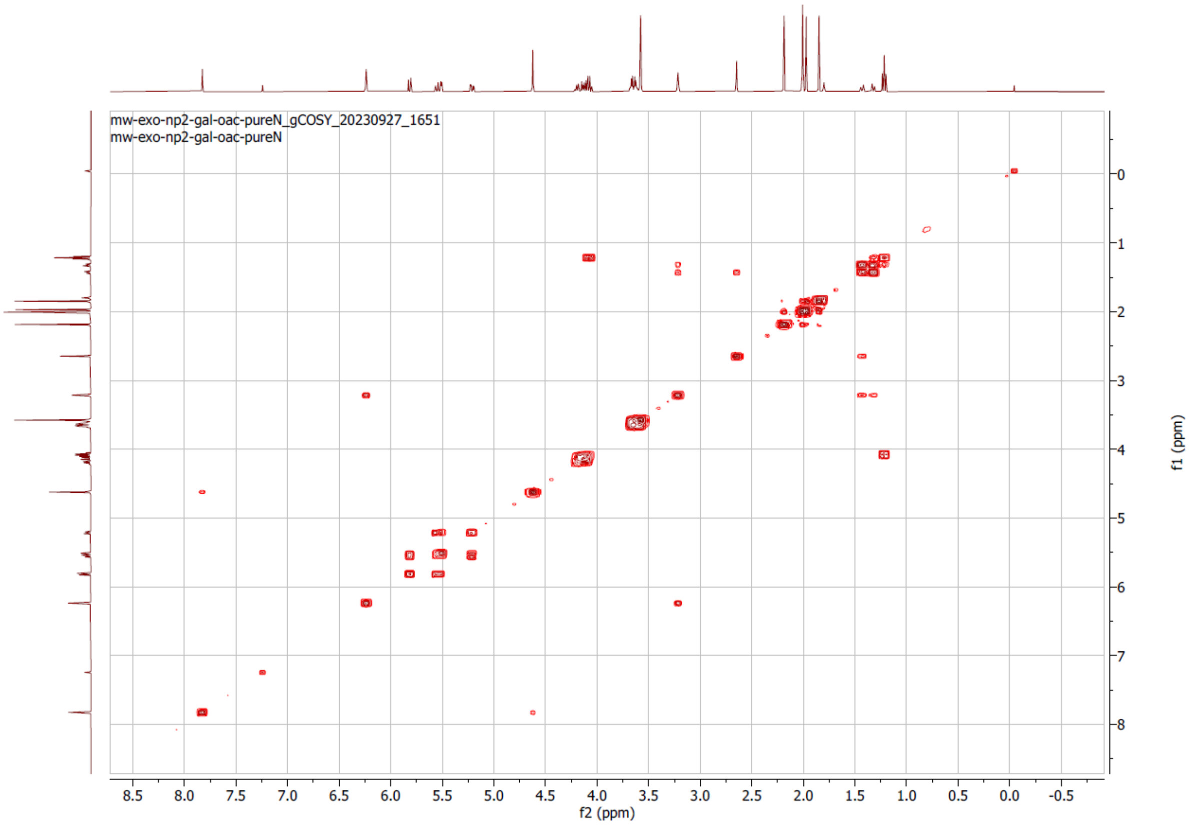
mw-exo-np2-gal-oac-pureN_gCOSY_20230927_1651
mw-exo-np2-gal-oac-pureN
f2 (ppm)
f1 (ppm)

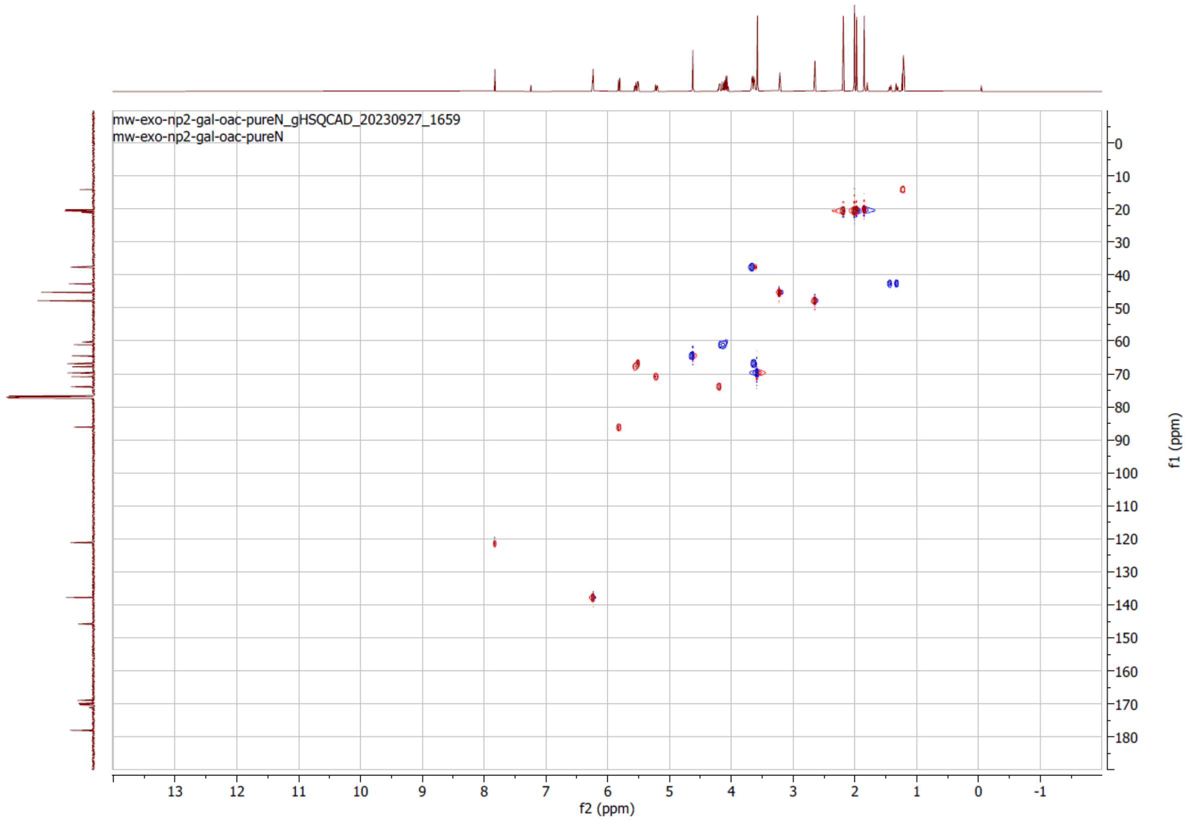
mw-exo-np2-gal-oac-pureN_gHSQCAD_20230927_1659
mw-exo-np2-gal-oac-pureN
f2 (ppm)
f1 (ppm)

^{1}H-NMR, ^{13}C, COSY, HSQC of Compound *endo*-β-Gal-OAc$_4$

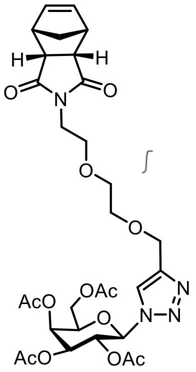

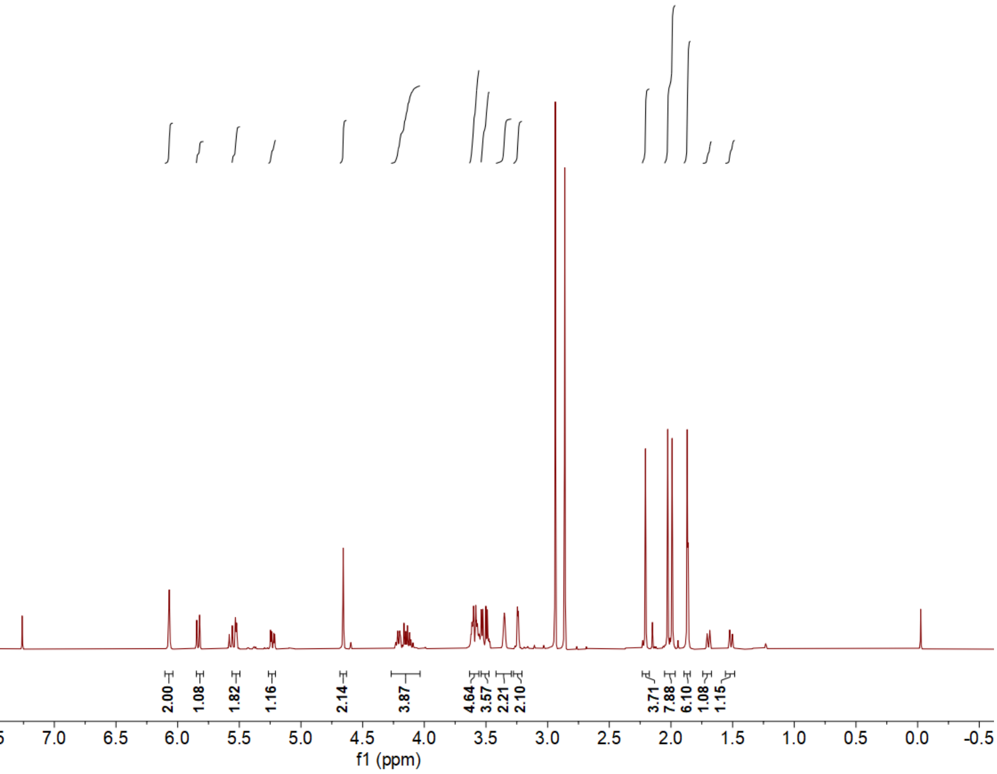

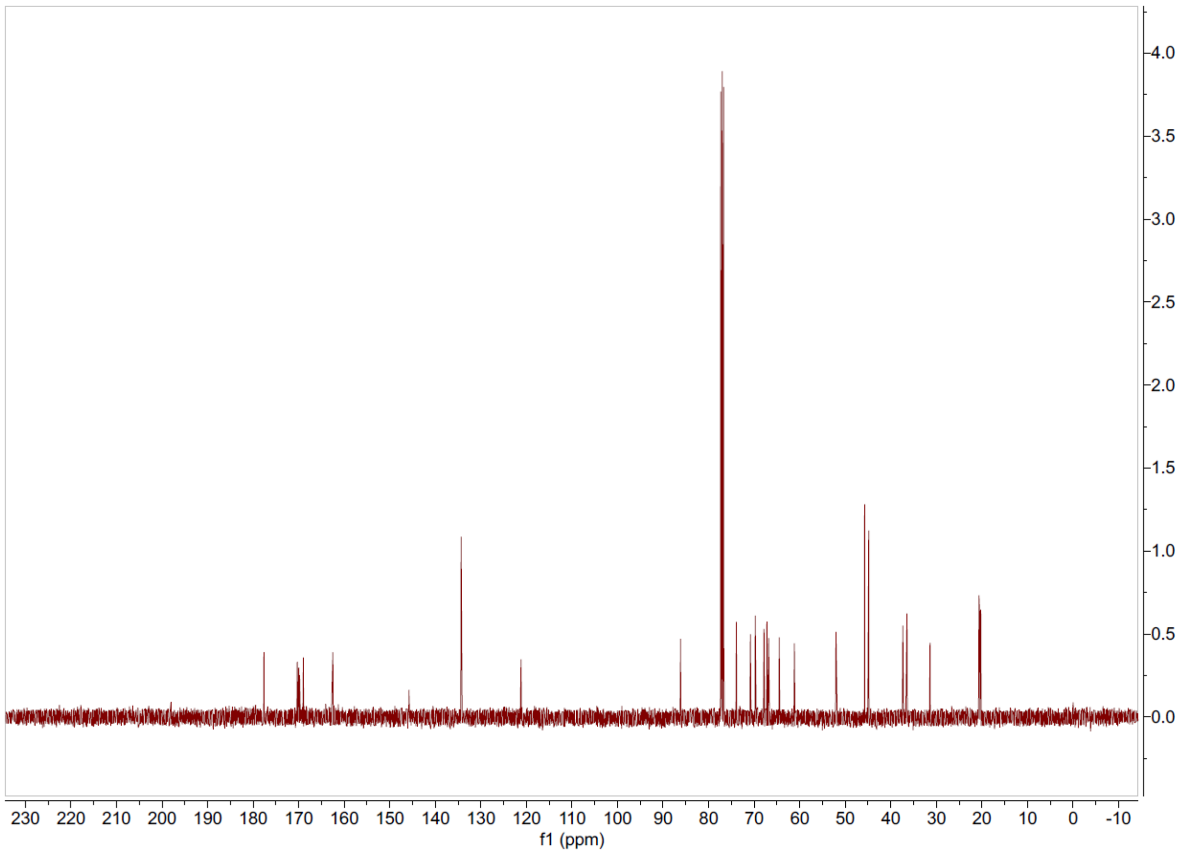

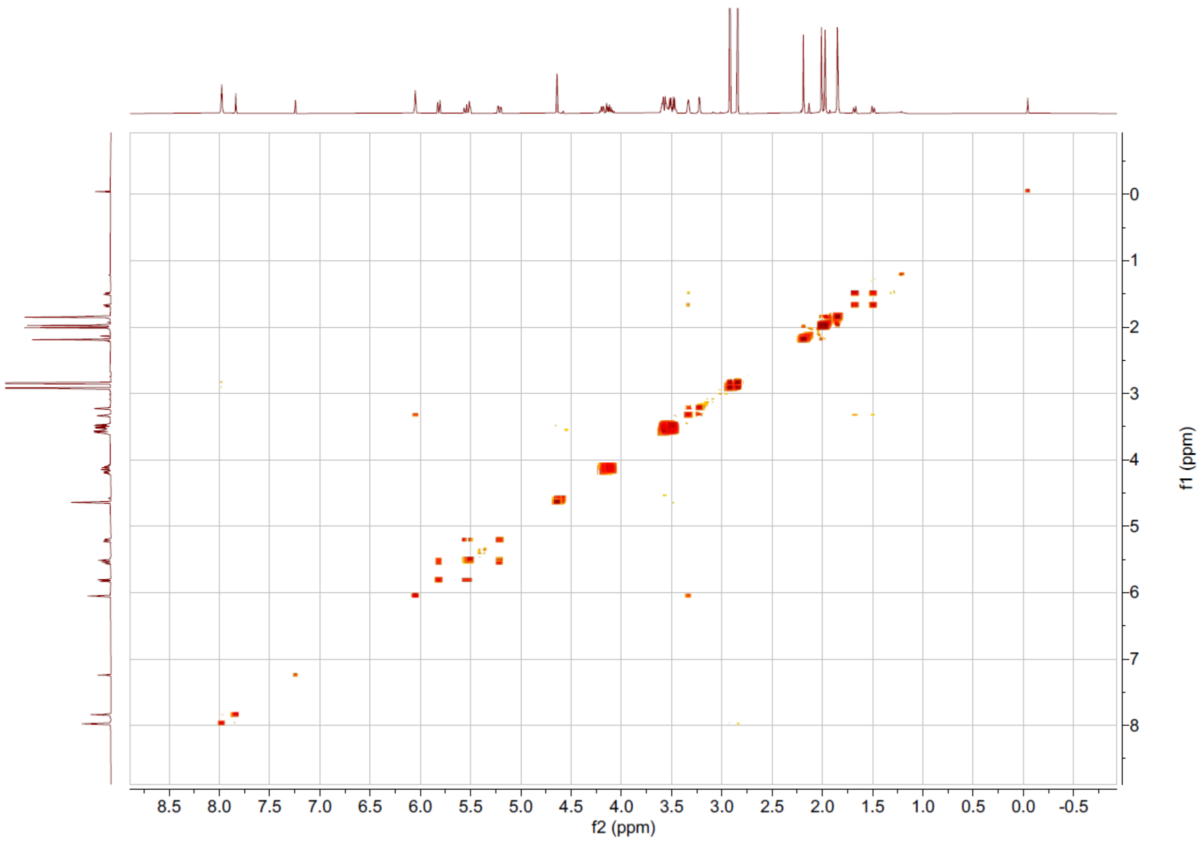

8.5
8.0
7.5
7.0
6.5
6.0
5.5
5.0
4.5
4.0
3.5
3.0
2.5
2.0
1.5
1.0
0.5
0.0
-0.5
f2 (ppm)
0
1
2
3
4
5
6
7
8
f1 (ppm)

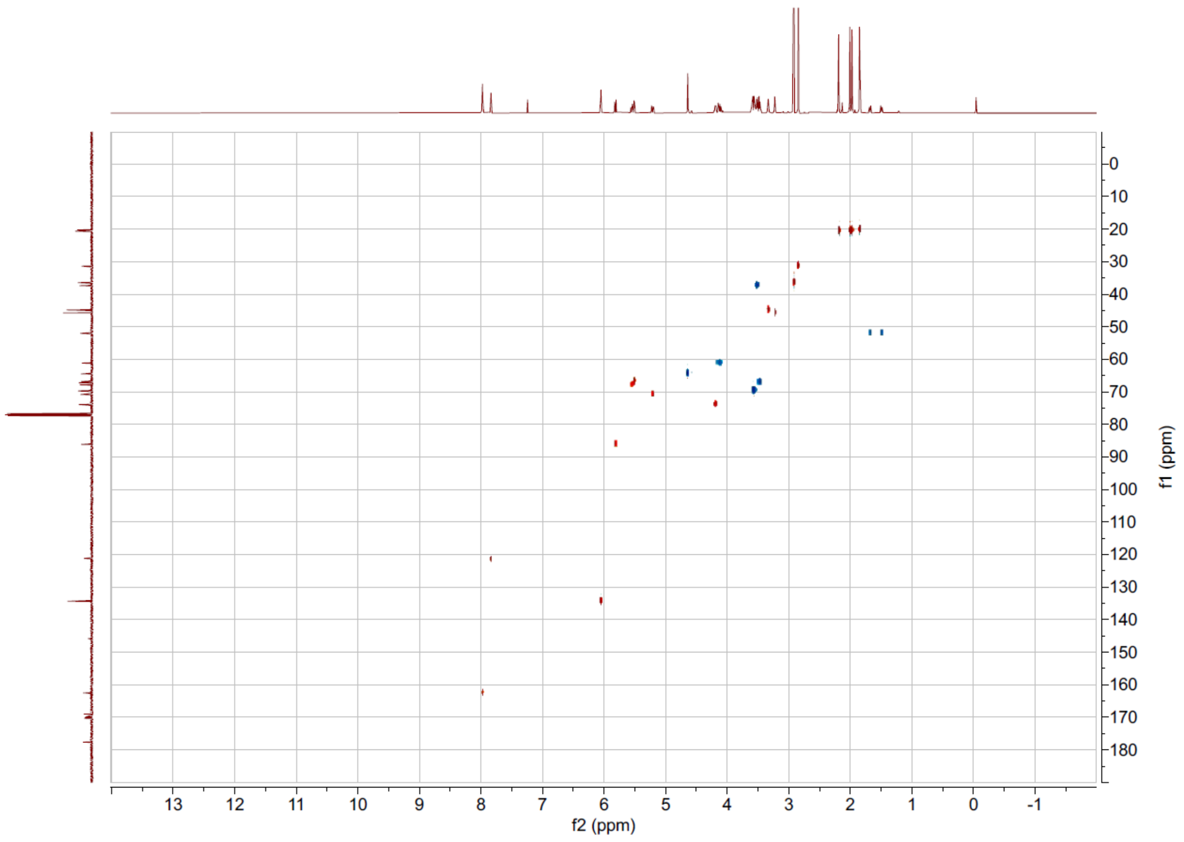

13
12
11
10
9
8
7
6
5
4
3
2
1
0
-1
f2 (ppm)
0
10
20
30
40
50
60
70
80
90
100
110
120
130
140
150
160
170
180
f1 (ppm)

^{1}H-NMR, ^{13}C, COSY, HSQC of Compound 14 (*exo*-α-Man)

mw-exo-np2-man-oh_PROTON_20240229_1233
mw-exo-np2-man-oh

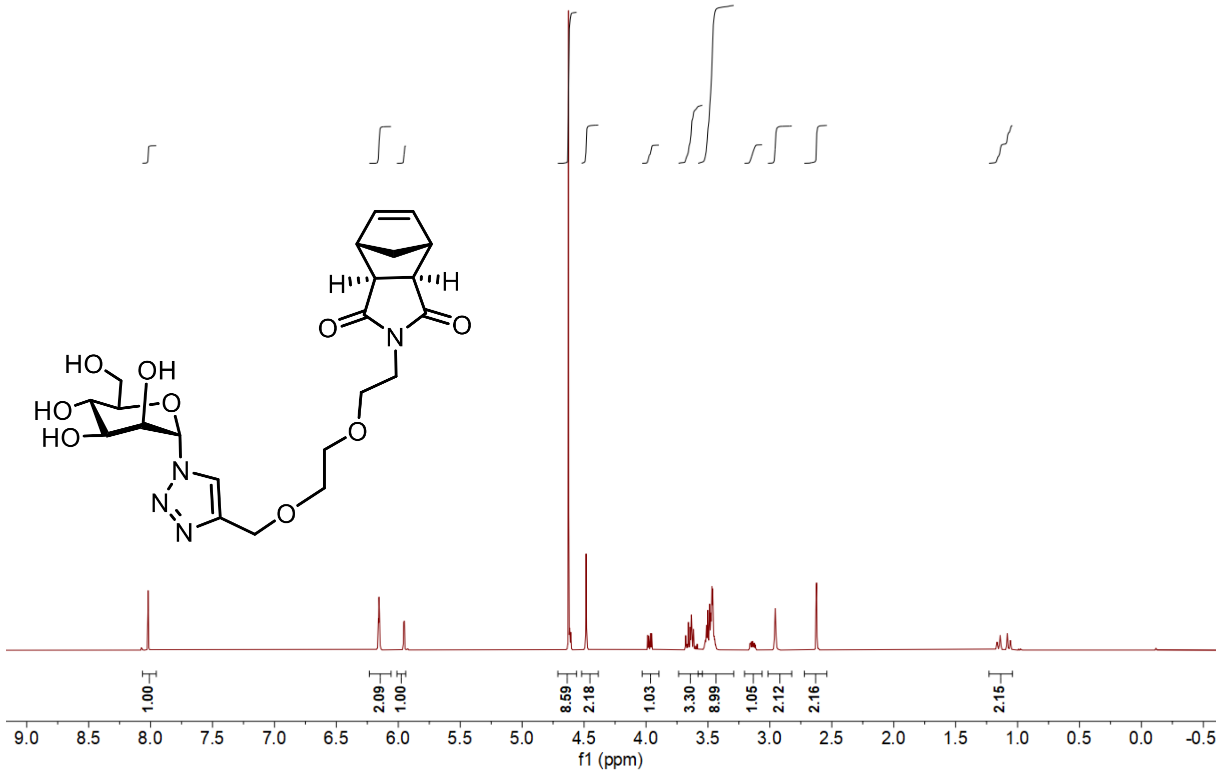

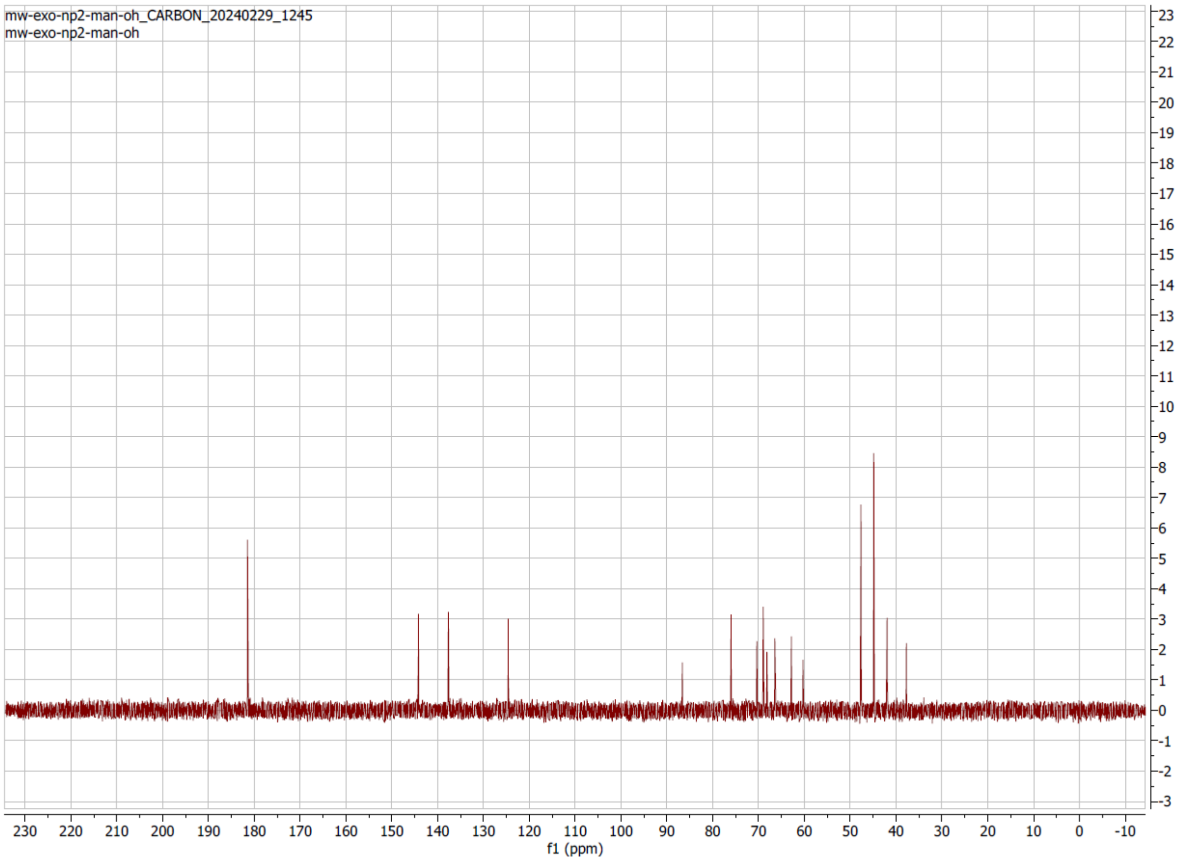

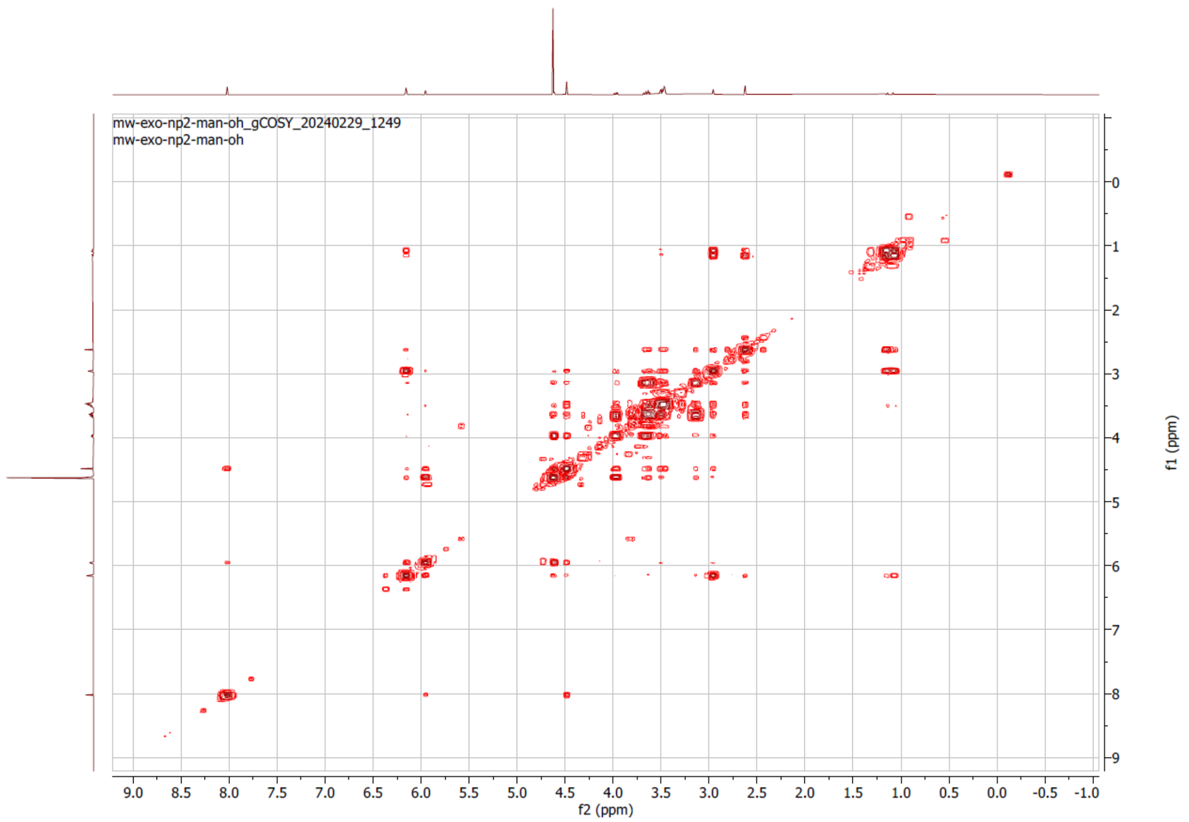
mw-exo-np2-man-oh_gCOSY_20240229_1249
mw-exo-np2-man-oh
f2 (ppm)
f1 (ppm)

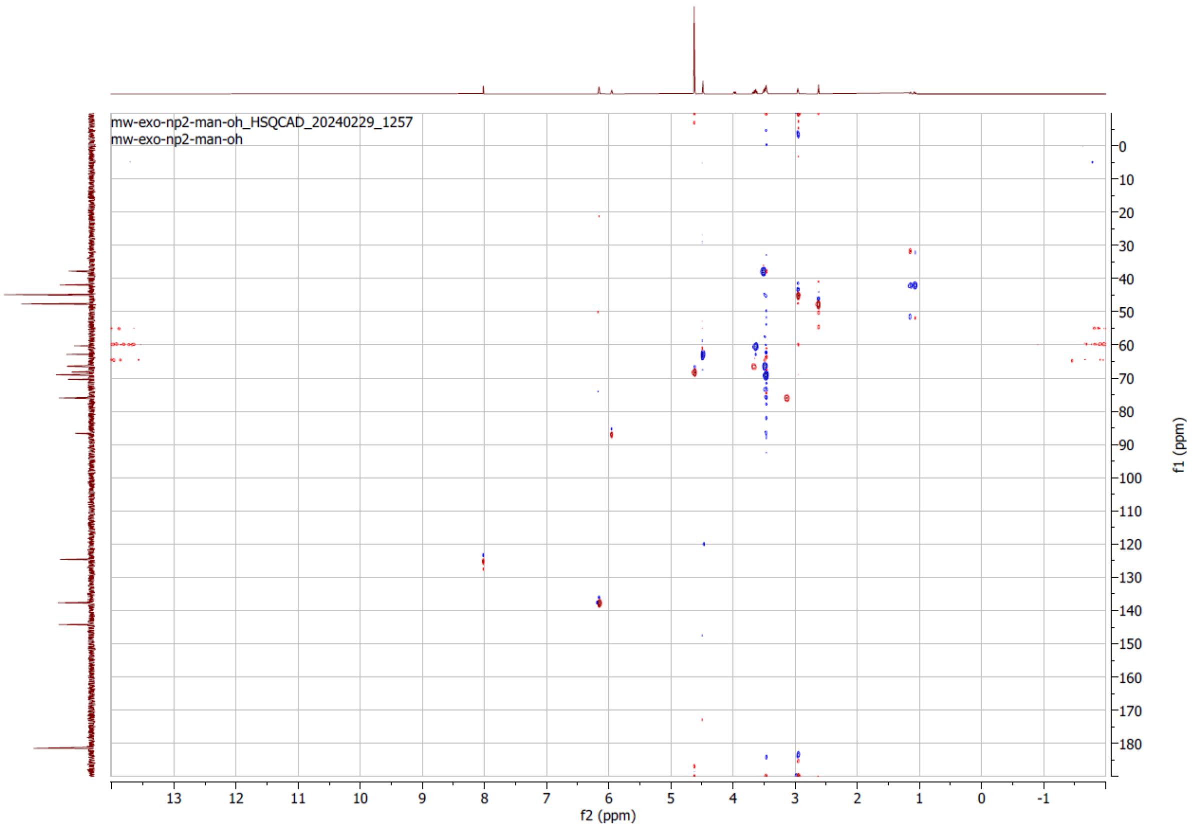
mw-exo-np2-man-oh_HSQCAD_20240229_1257
mw-exo-np2-man-oh
f2 (ppm)
f1 (ppm)

^{1}H-NMR, ^{13}C, COSY, HSQC of Compound 15 (*exo*-β-Glc)

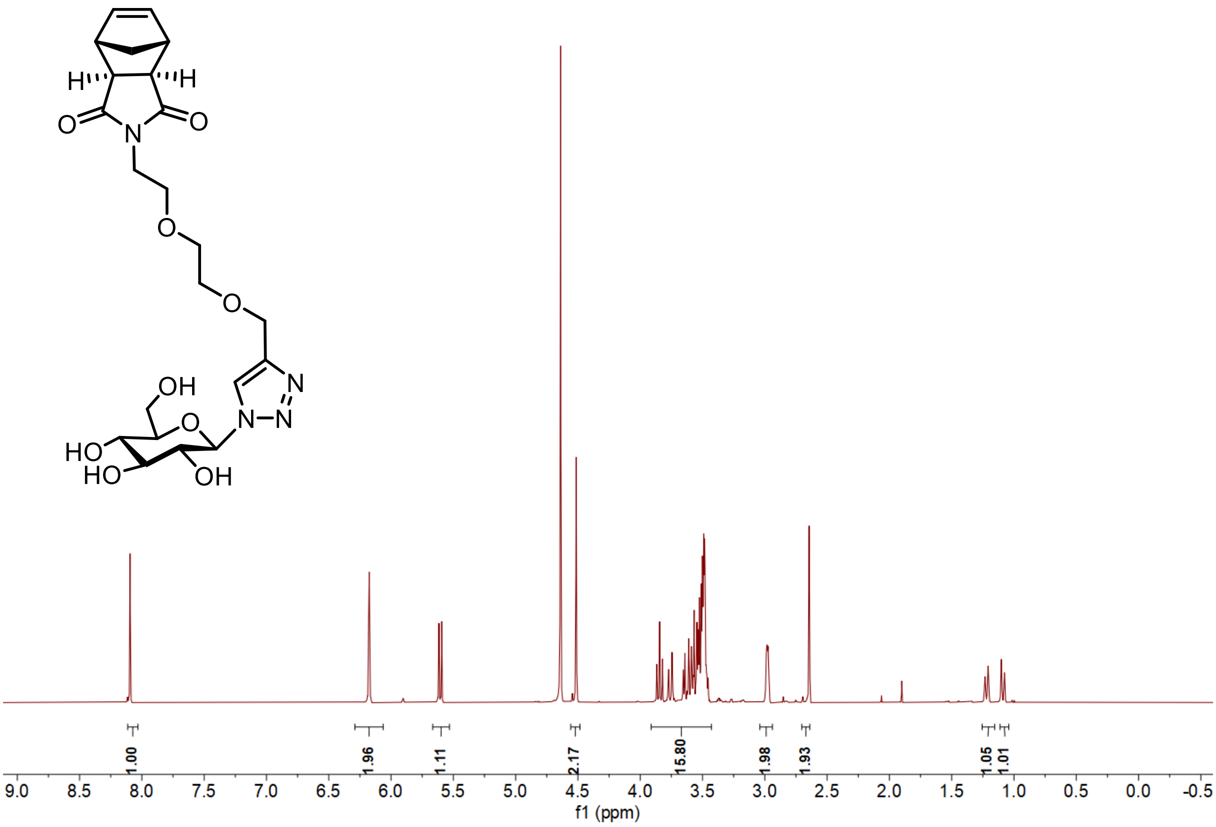

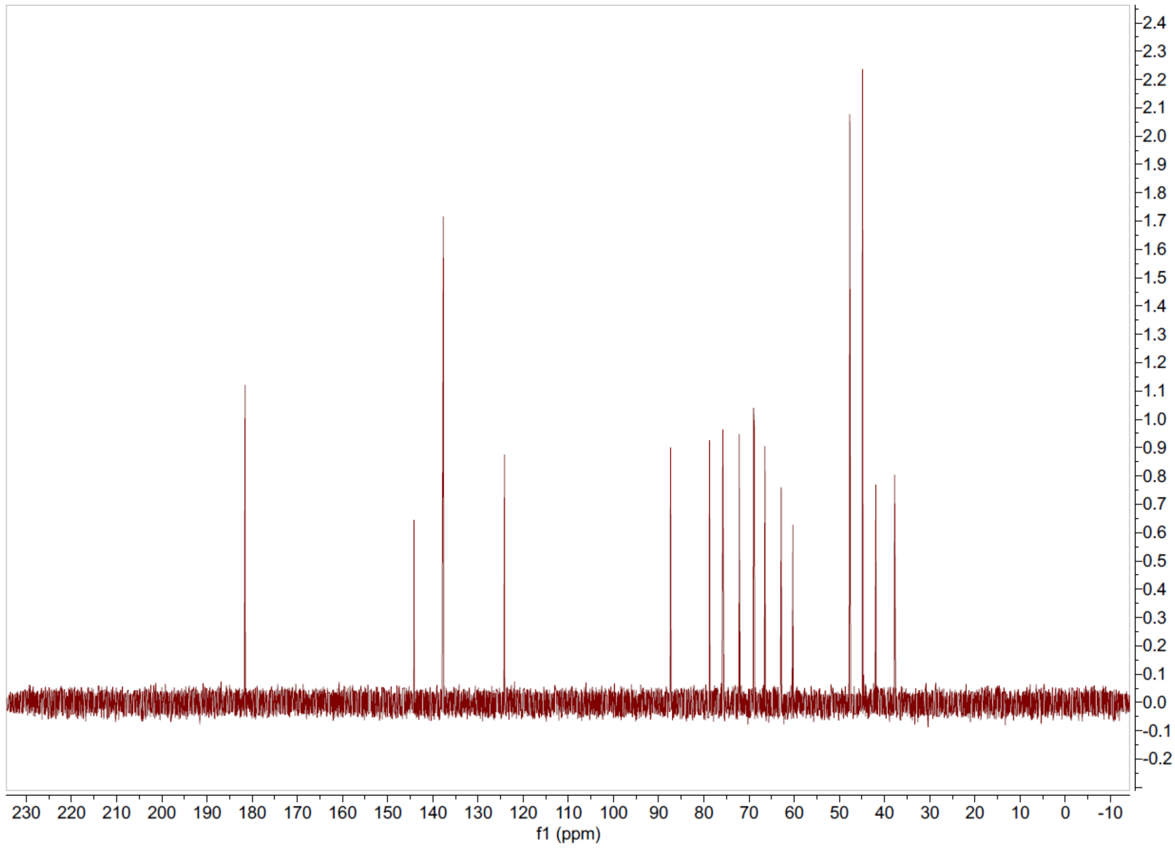

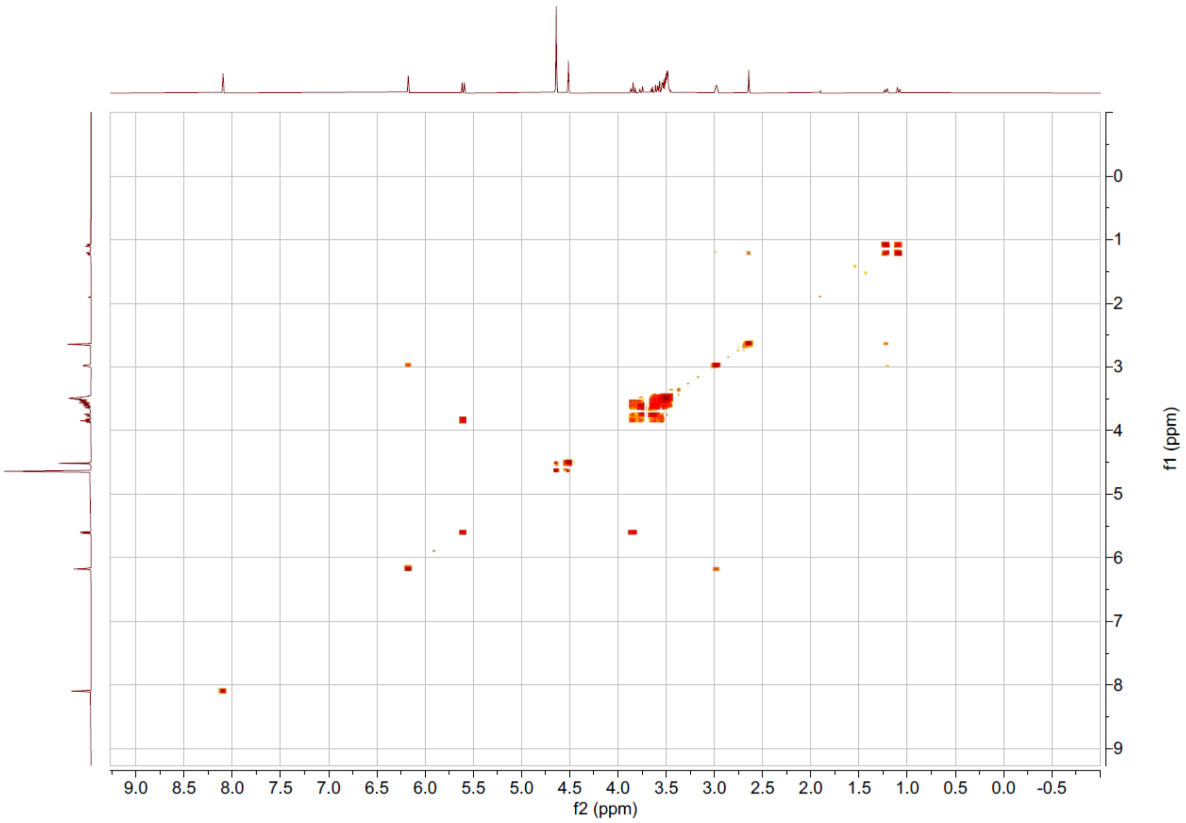
9.0
8.5
8.0
7.5
7.0
6.5
6.0
5.5
5.0
4.5
4.0
3.5
3.0
2.5
2.0
1.5
1.0
0.5
0.0
-0.5
f2 (ppm)
0
1
2
3
4
5
6
7
8
9
f1 (ppm)

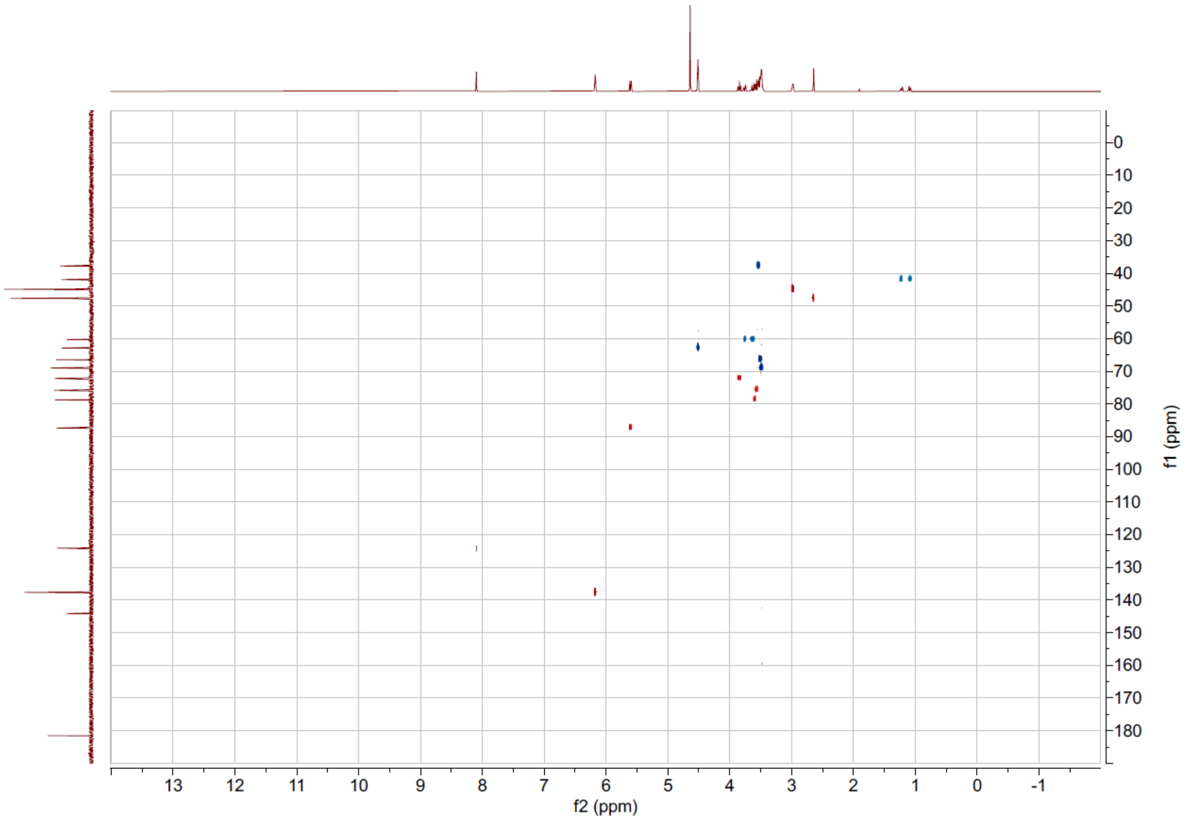
13
12
11
10
9
8
7
6
5
4
3
2
1
0
-1
f2 (ppm)
0
10
20
30
40
50
60
70
80
90
100
110
120
130
140
150
160
170
180
f1 (ppm)

^{1}H-NMR, ^{13}C, COSY, HSQC of Compound 16 (*exo*-β-Gal)

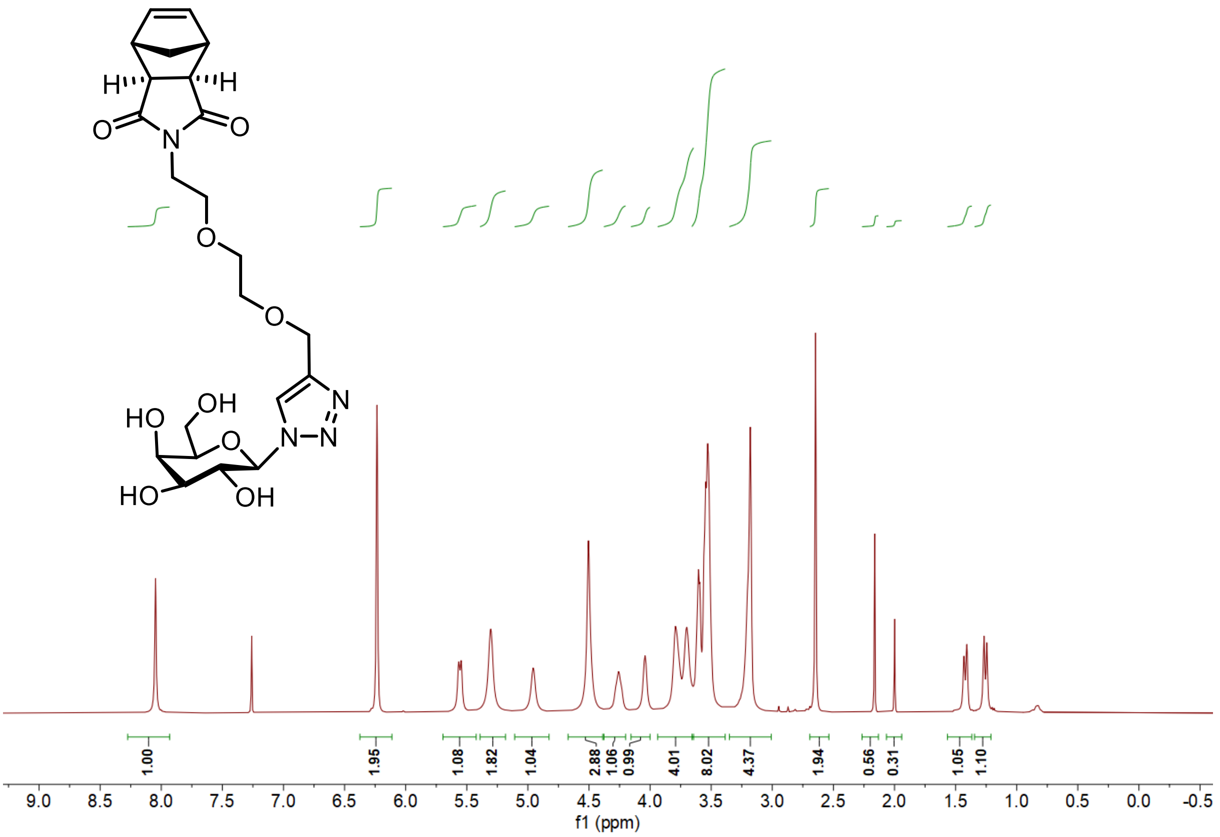

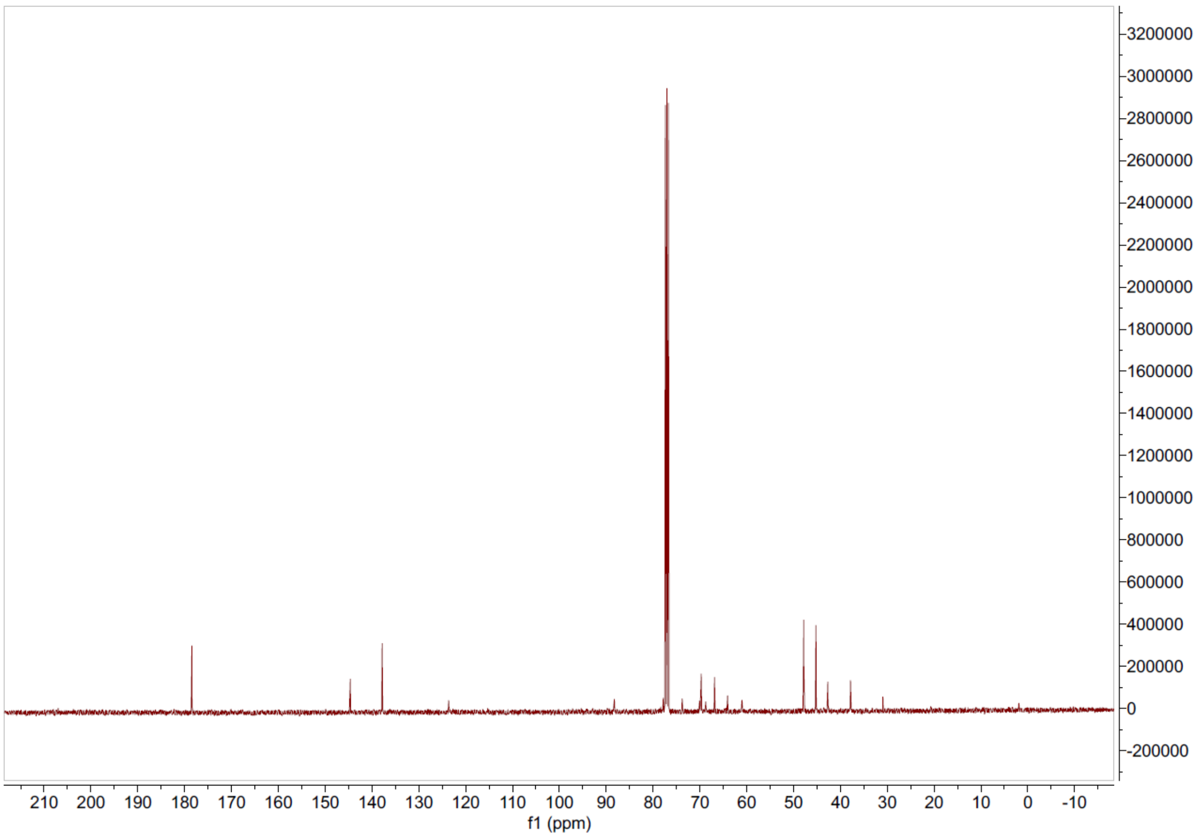

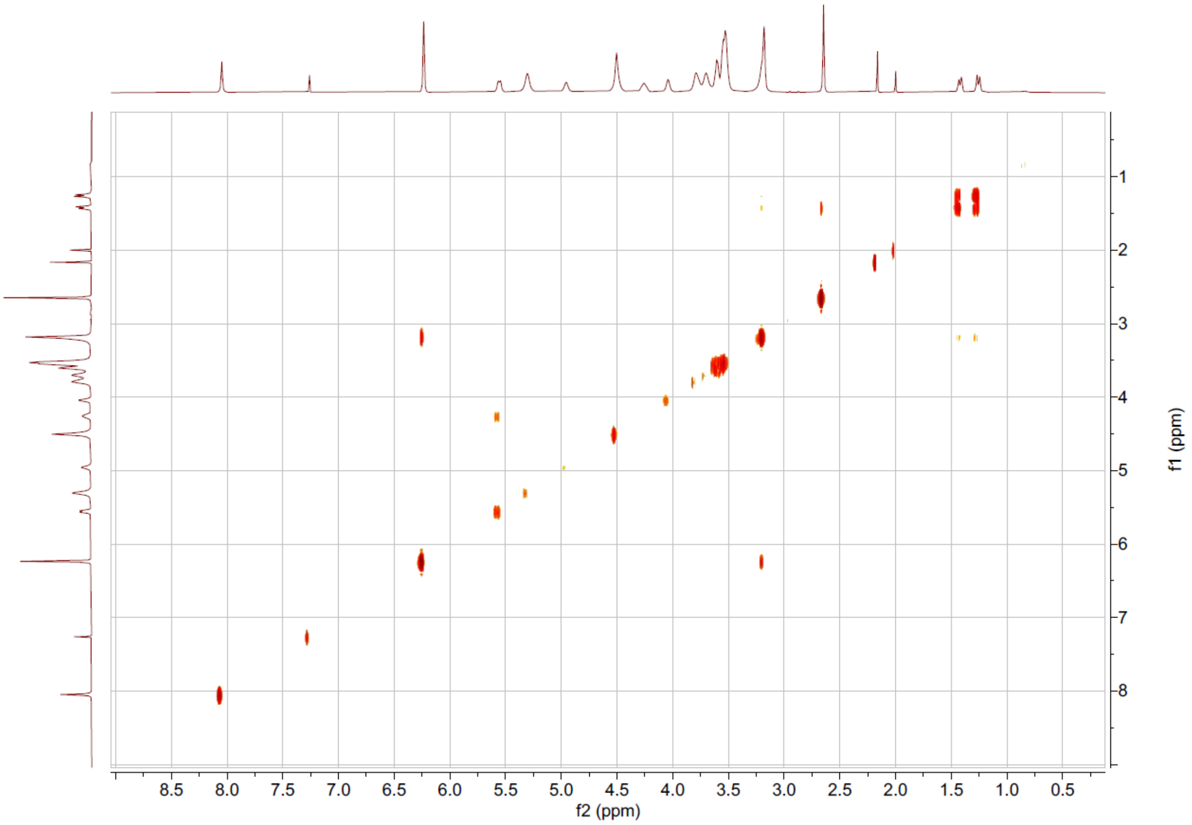
8.5
8.0
7.5
7.0
6.5
6.0
5.5
5.0
4.5
4.0
3.5
3.0
2.5
2.0
1.5
1.0
0.5
f2 (ppm)
1
2
3
4
5
6
7
8
f1 (ppm)

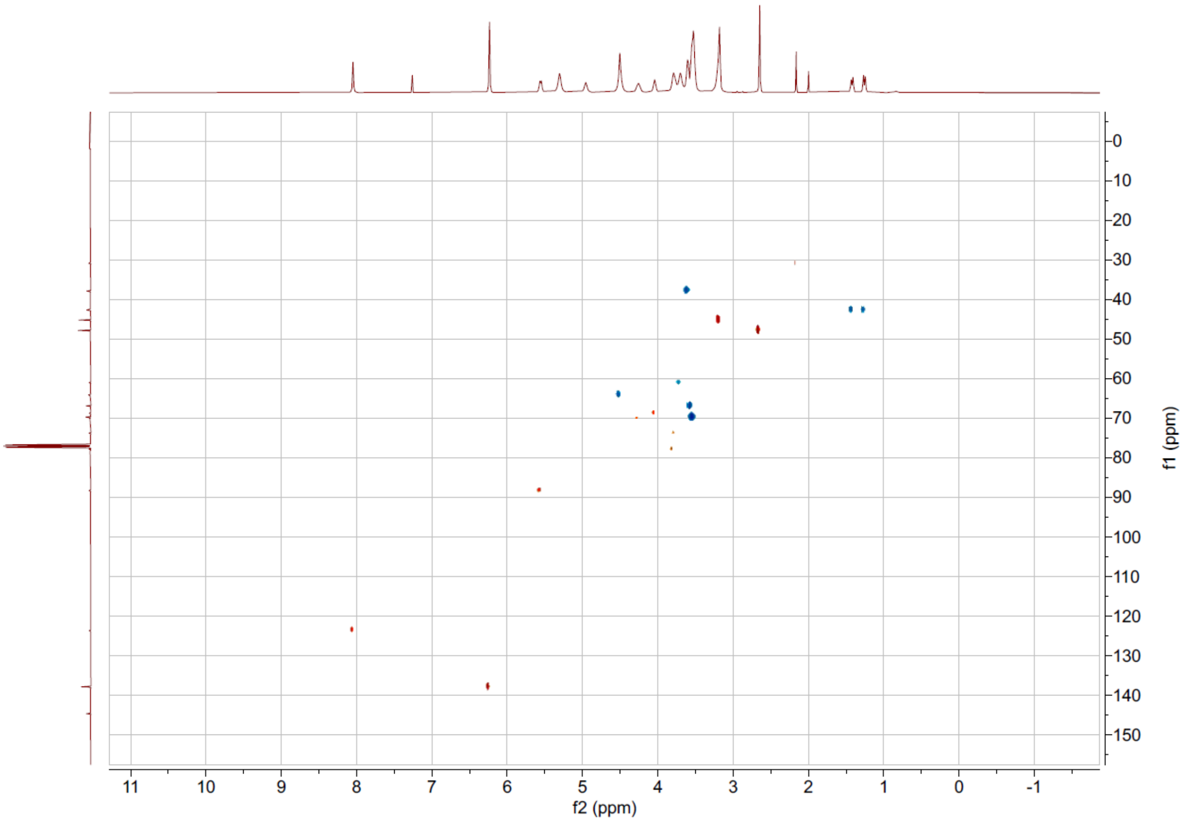
11
10
9
8
7
6
5
4
3
2
1
0
-1
f2 (ppm)
0
10
20
30
40
50
60
70
80
90
100
110
120
130
140
150
f1 (ppm)

^{1}H-NMR, ^{13}C, COSY, HSQC of Compound 17 (*exo*-α-Gal)

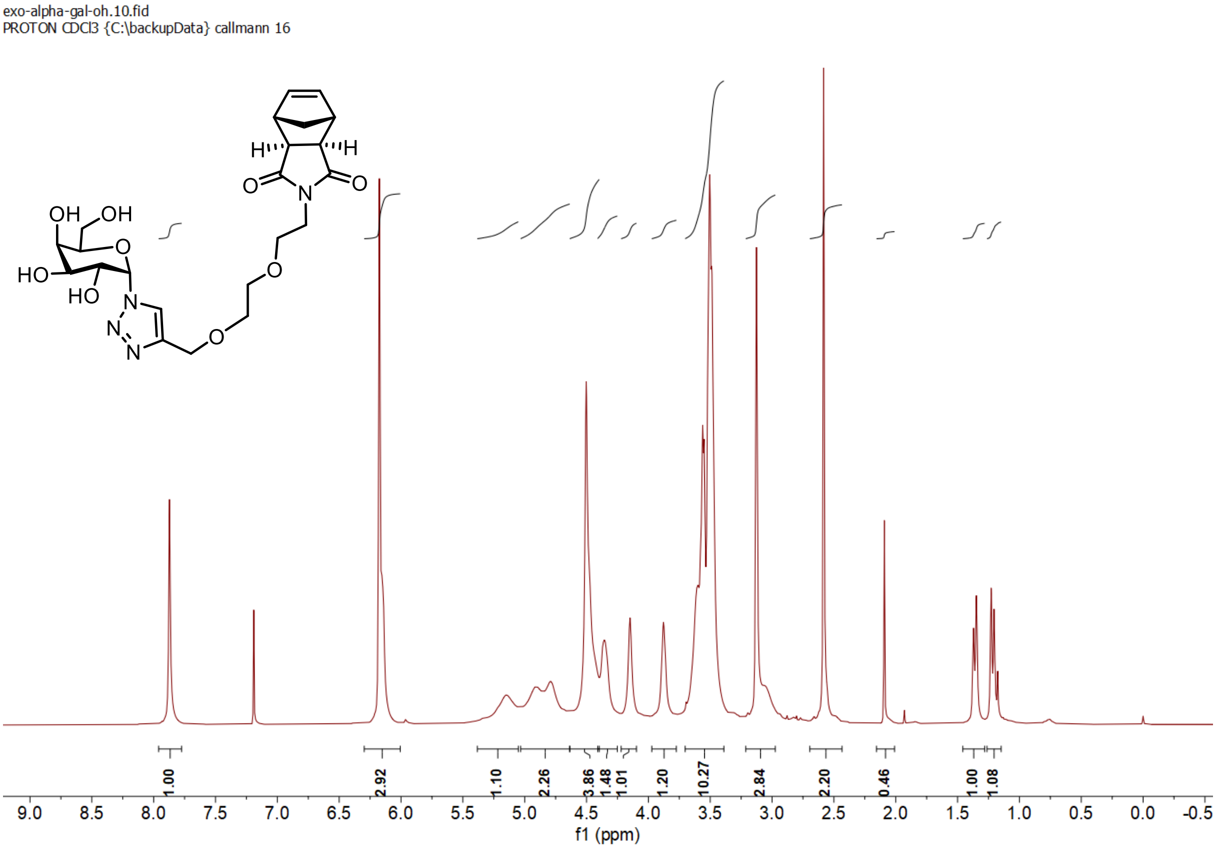

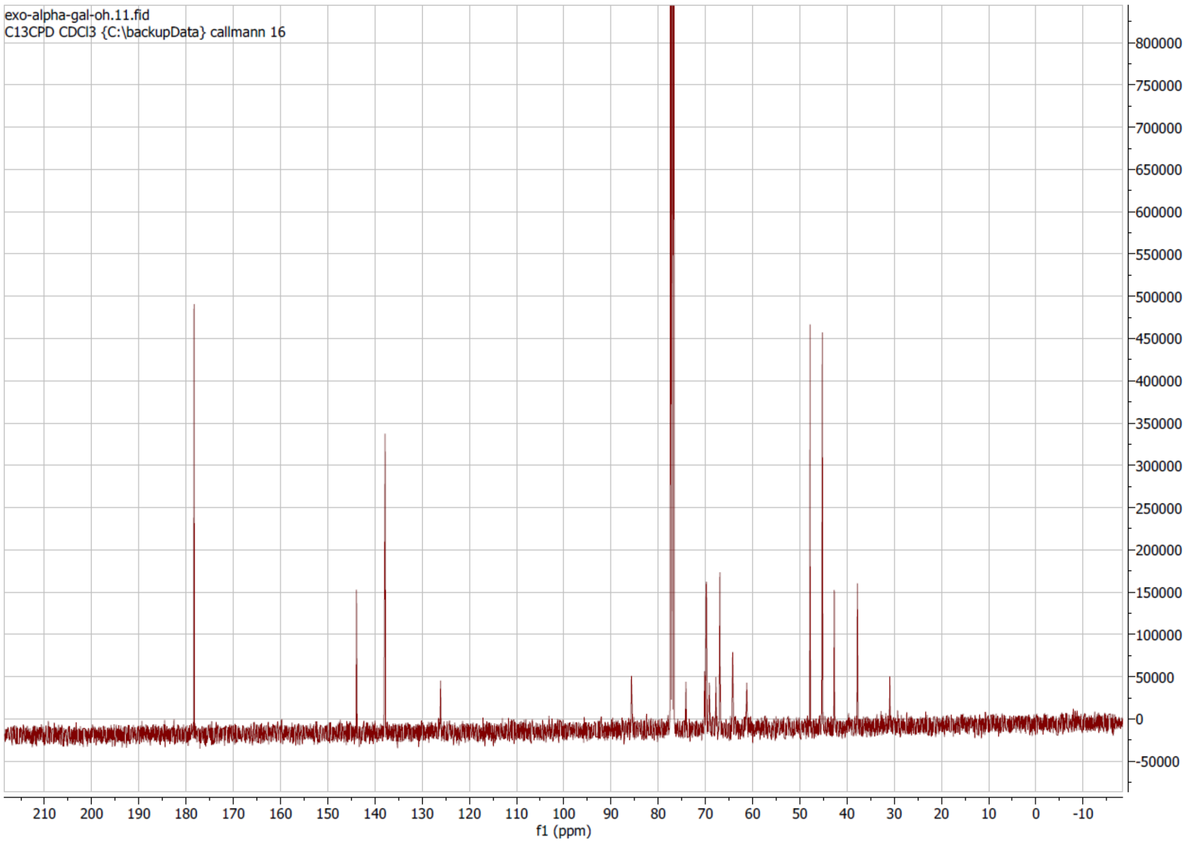

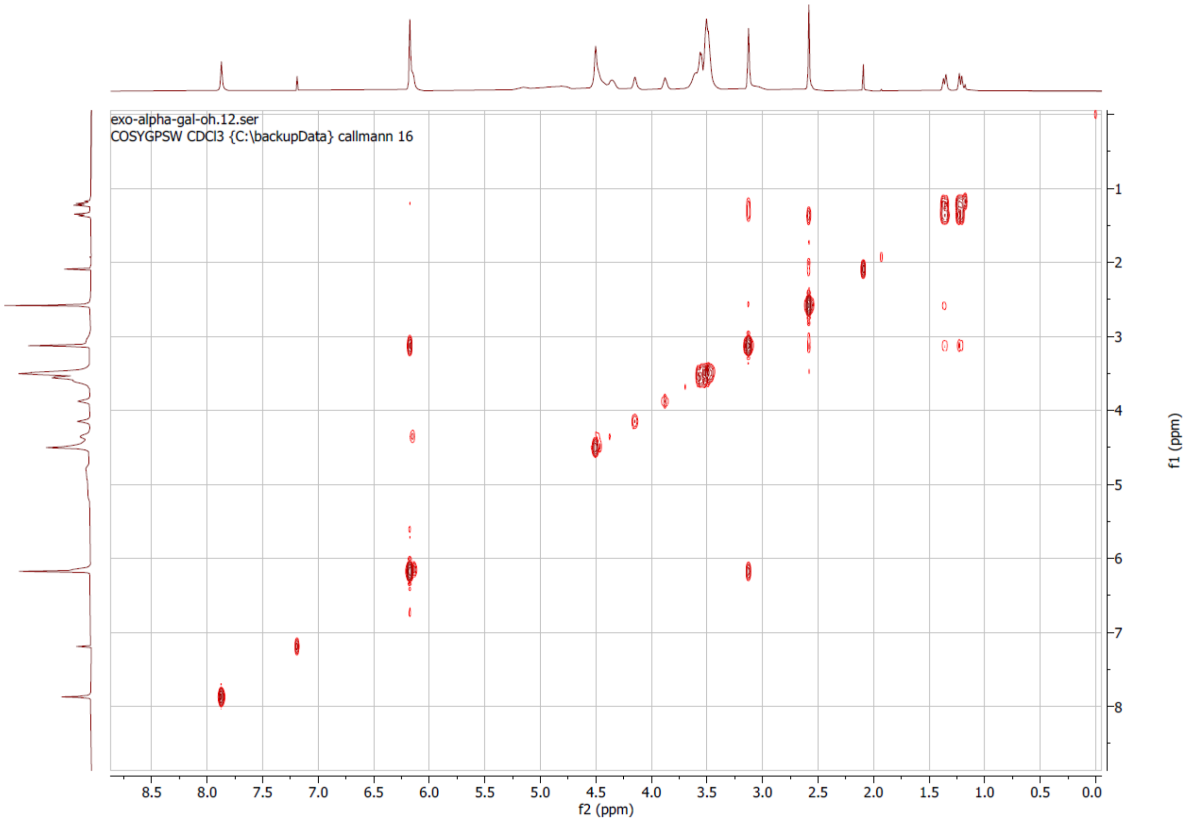
exo-alpha-gal-oh.12.ser
COSYGPSW CDCl3 {C:\backupData} callmann 16
8.5 8.0 7.5 7.0 6.5 6.0 5.5 5.0 4.5 4.0 3.5 3.0 2.5 2.0 1.5 1.0 0.5 0.0
f2 (ppm)
1 2 3 4 5 6 7 8
f1 (ppm)

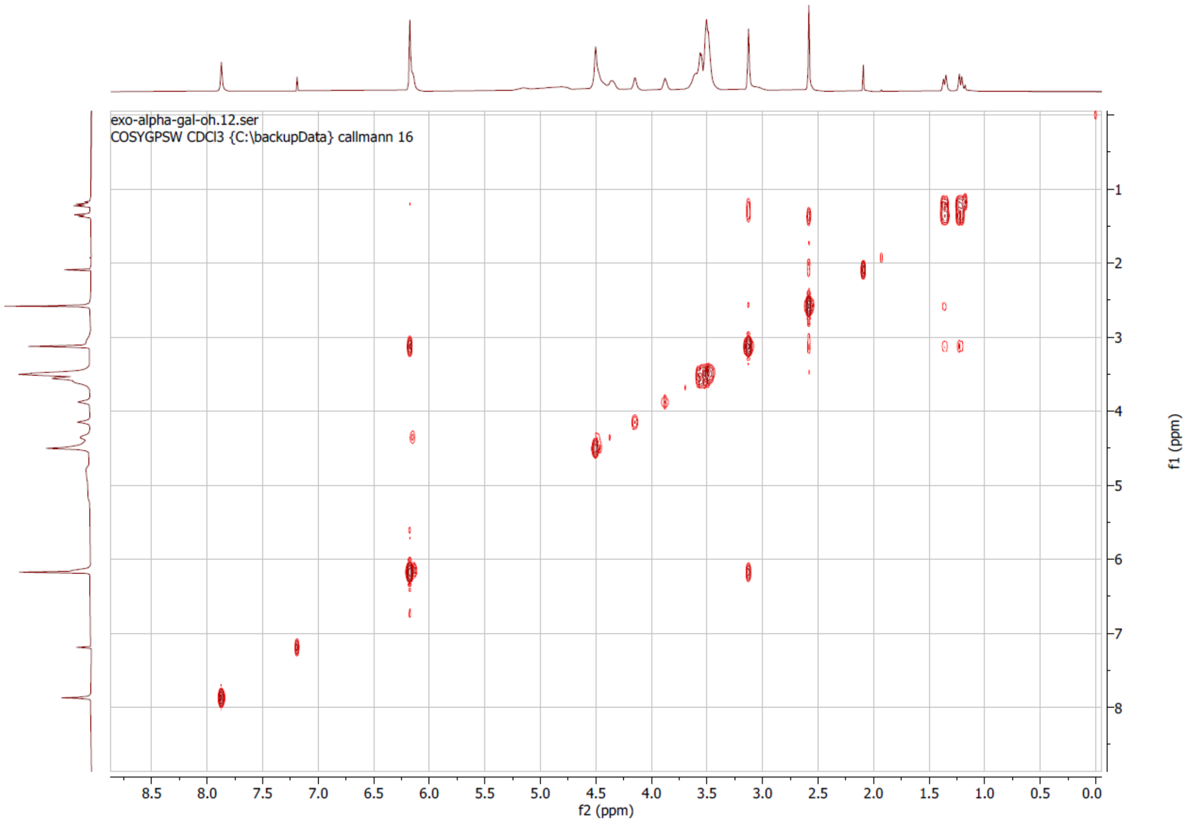
exo-alpha-gal-oh.12.ser
COSYGPSW CDCl3 {C:\backupData} callmann 16
8.5 8.0 7.5 7.0 6.5 6.0 5.5 5.0 4.5 4.0 3.5 3.0 2.5 2.0 1.5 1.0 0.5 0.0
f2 (ppm)
1 2 3 4 5 6 7 8
f1 (ppm)

^{1}H-NMR, ^{13}C, COSY, HSQC of Compound 18 (*exo*-α-Glc)

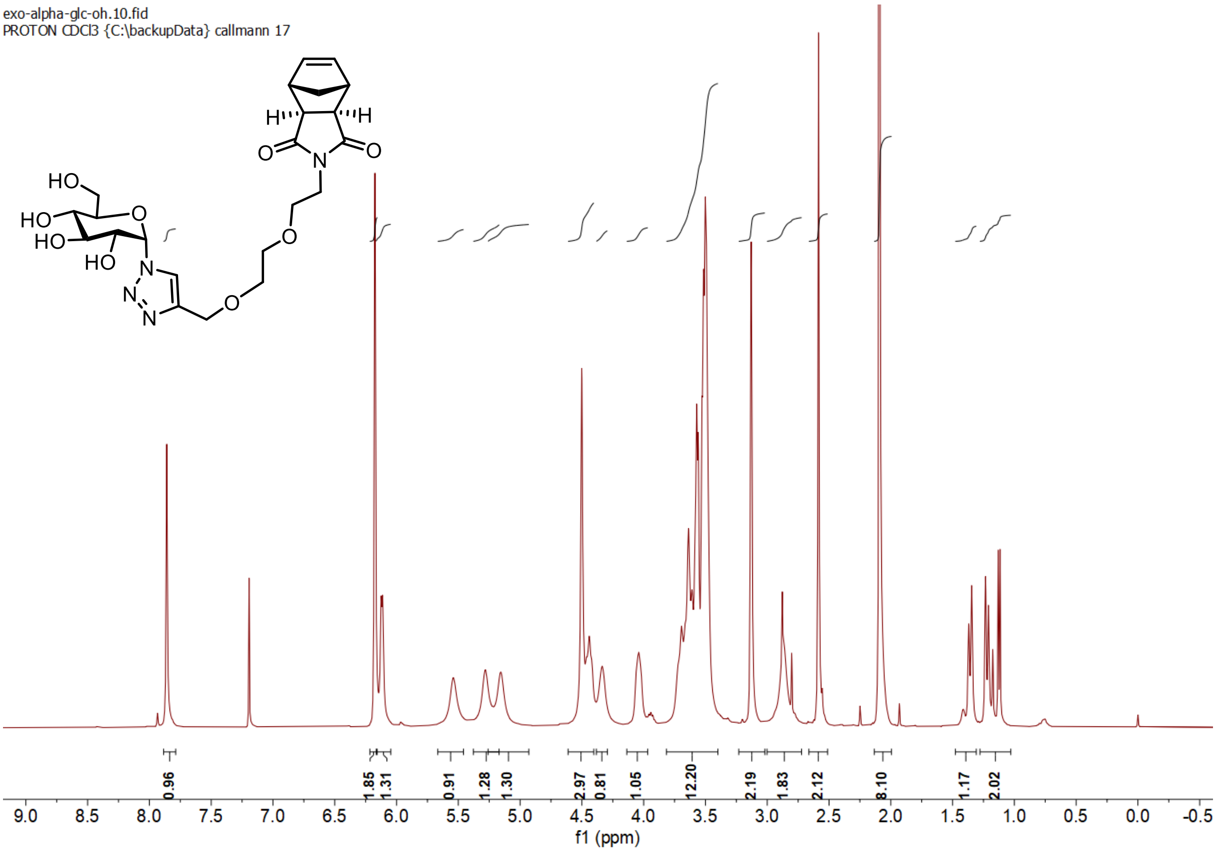

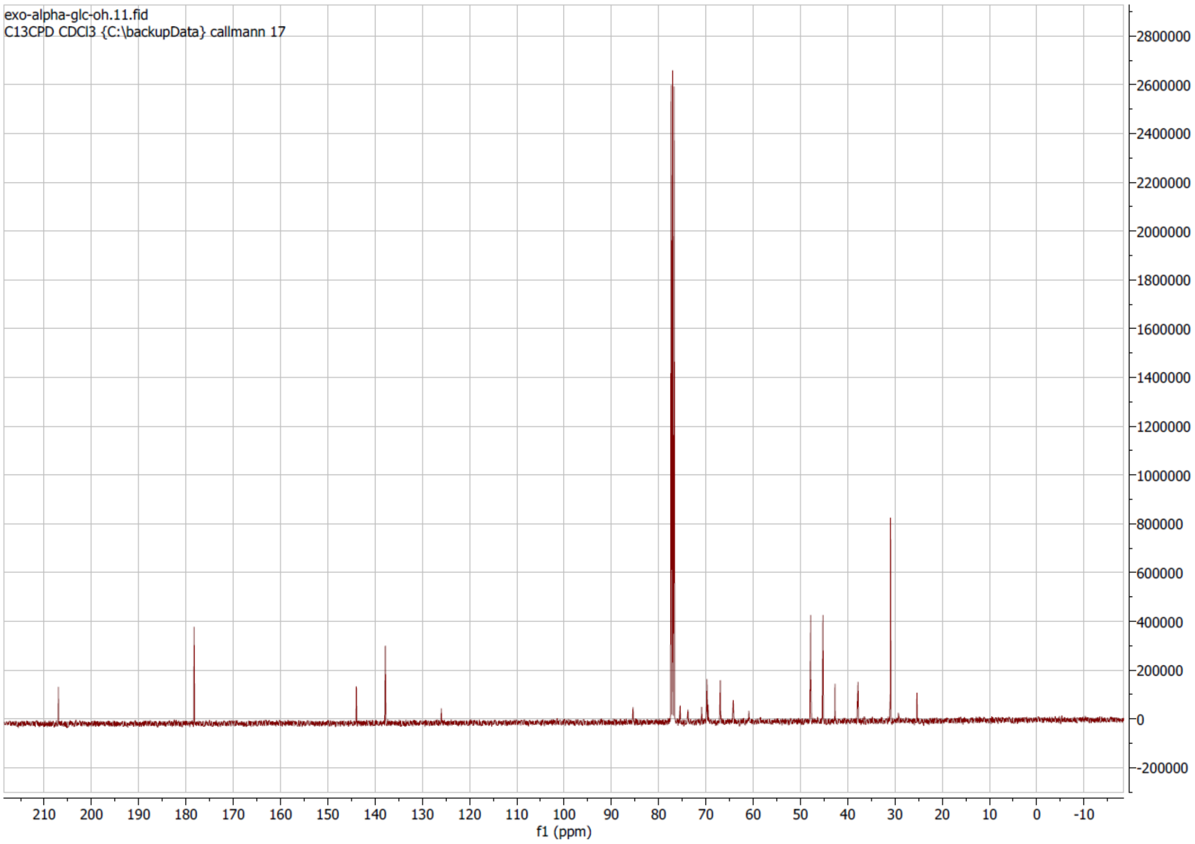

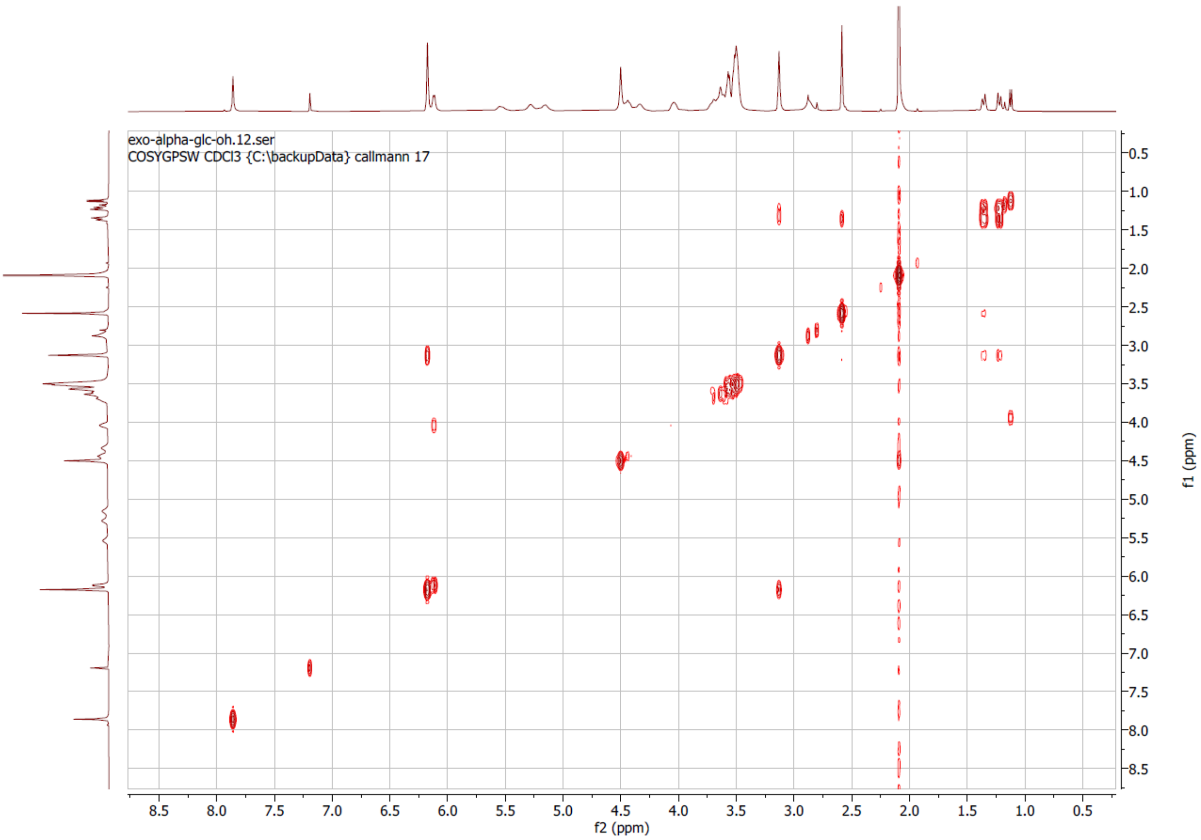

exo-alpha-glc-oh.12.ser
COSYGPSW CDCl3 {C:\backupData} callmann 17
f2 (ppm)
f1 (ppm)

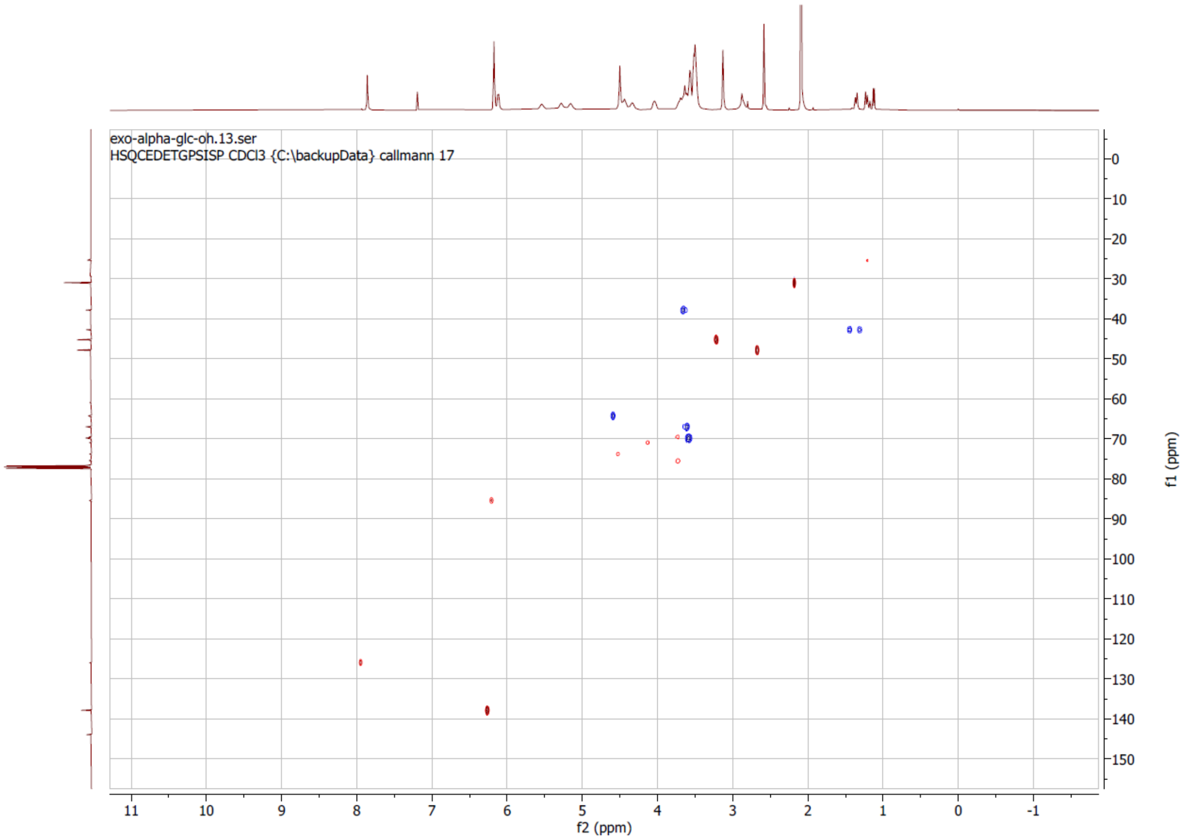

exo-alpha-glc-oh.13.ser
HSQCEDETGPSISP CDCl3 {C:\backupData} callmann 17
f2 (ppm)
f1 (ppm)

^{1}H-NMR, ^{13}C, COSY, HSQC of Compound 19 (*endo*-α-Man)

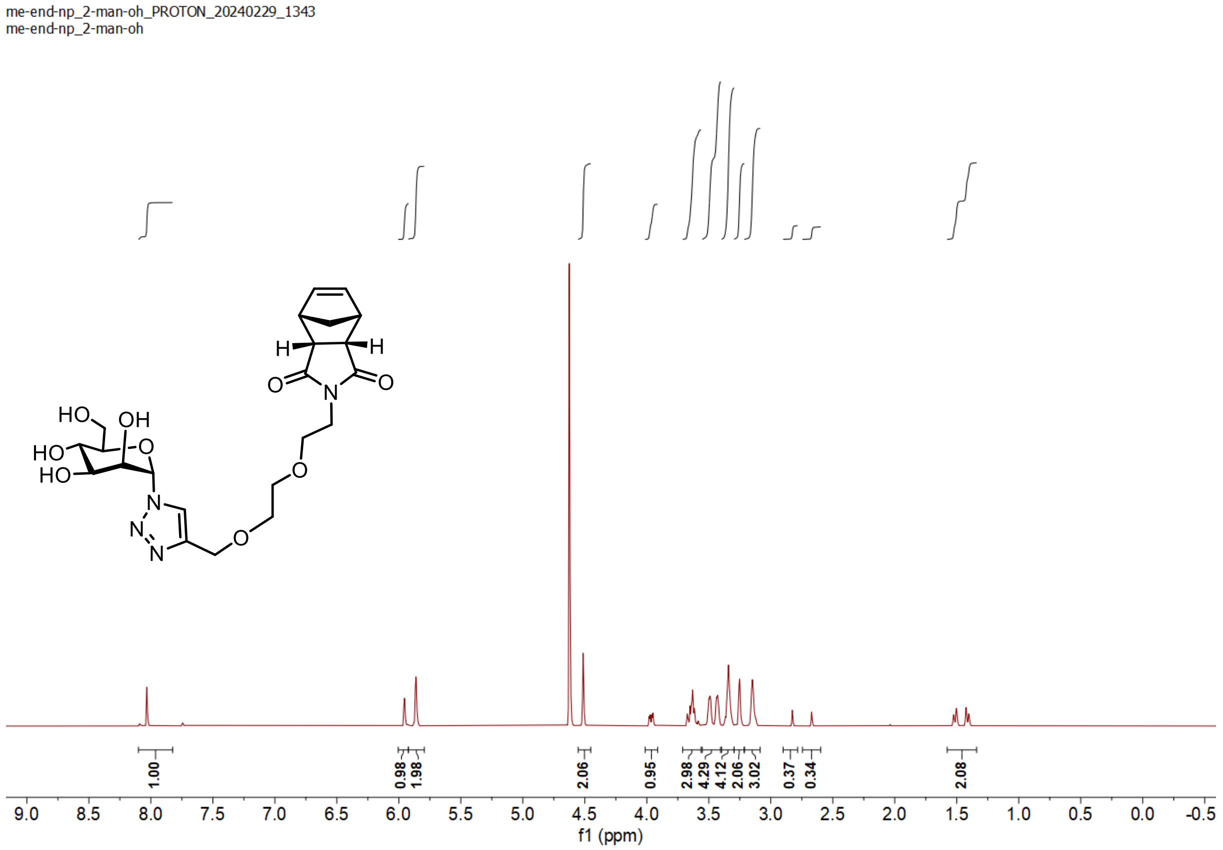

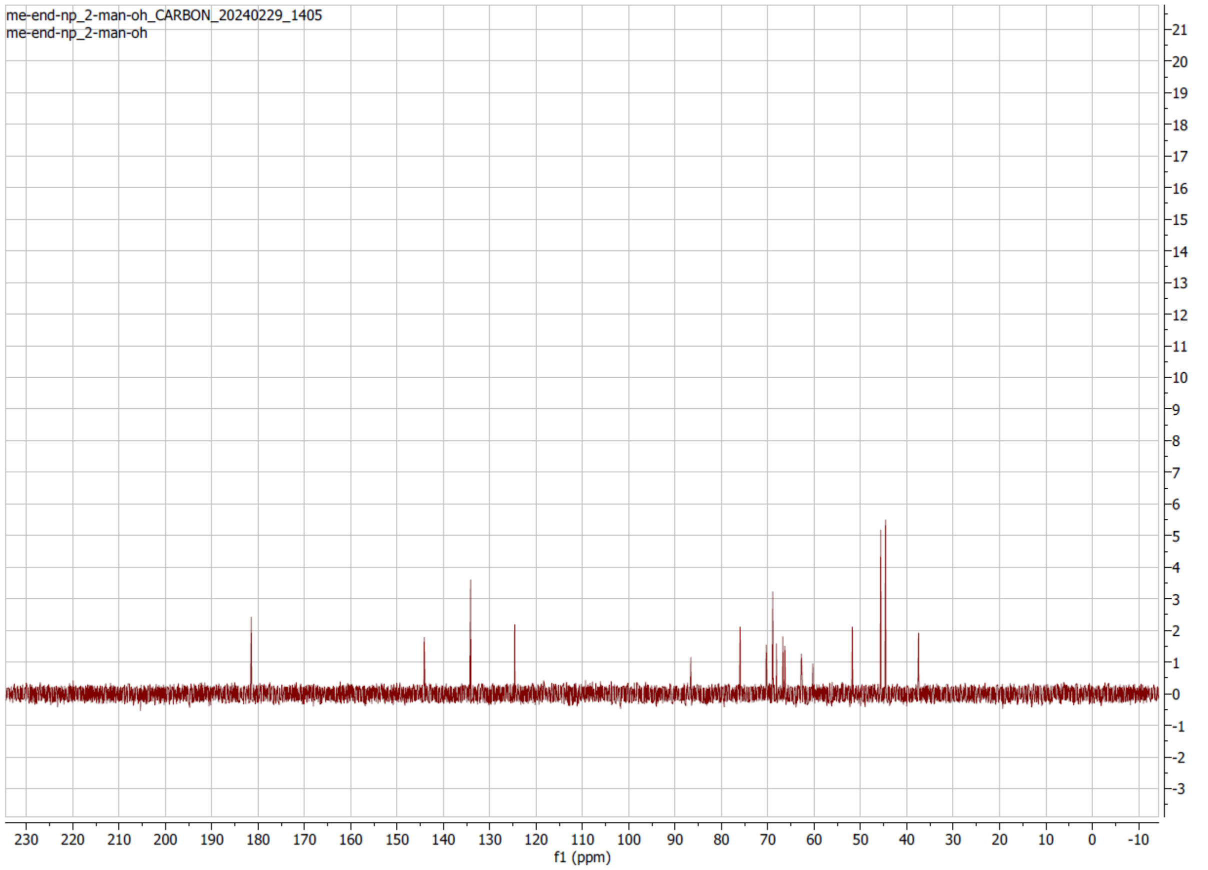

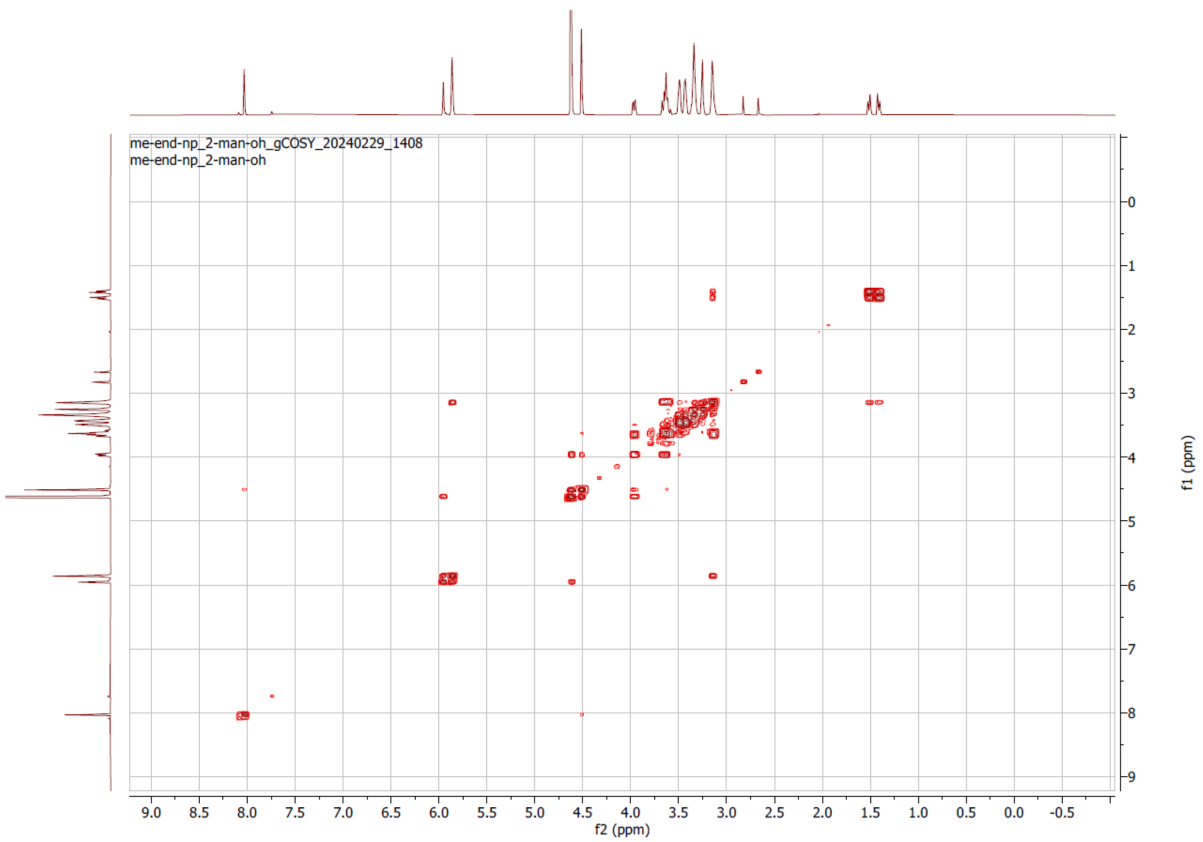
me-end-np_2-man-oh_gCOSY_20240229_1408
me-end-np_2-man-oh
f2 (ppm)
f1 (ppm)

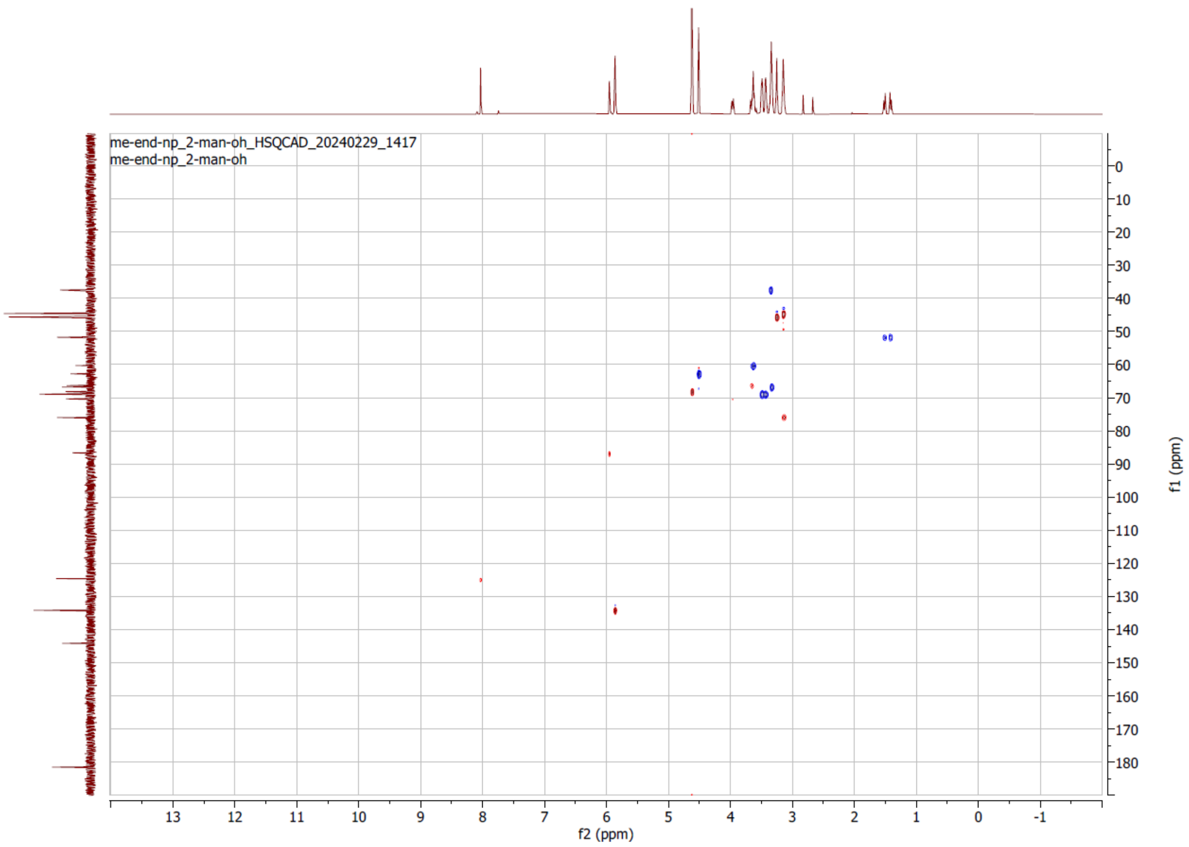
me-end-np_2-man-oh_HSQCAD_20240229_1417
me-end-np_2-man-oh
f2 (ppm)
f1 (ppm)

^{1}H-NMR, ^{13}C, COSY, HSQC of Compound 20 (*endo*-β-Glc)

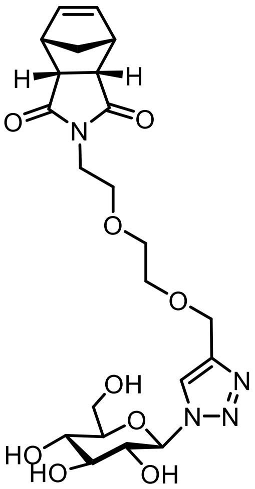

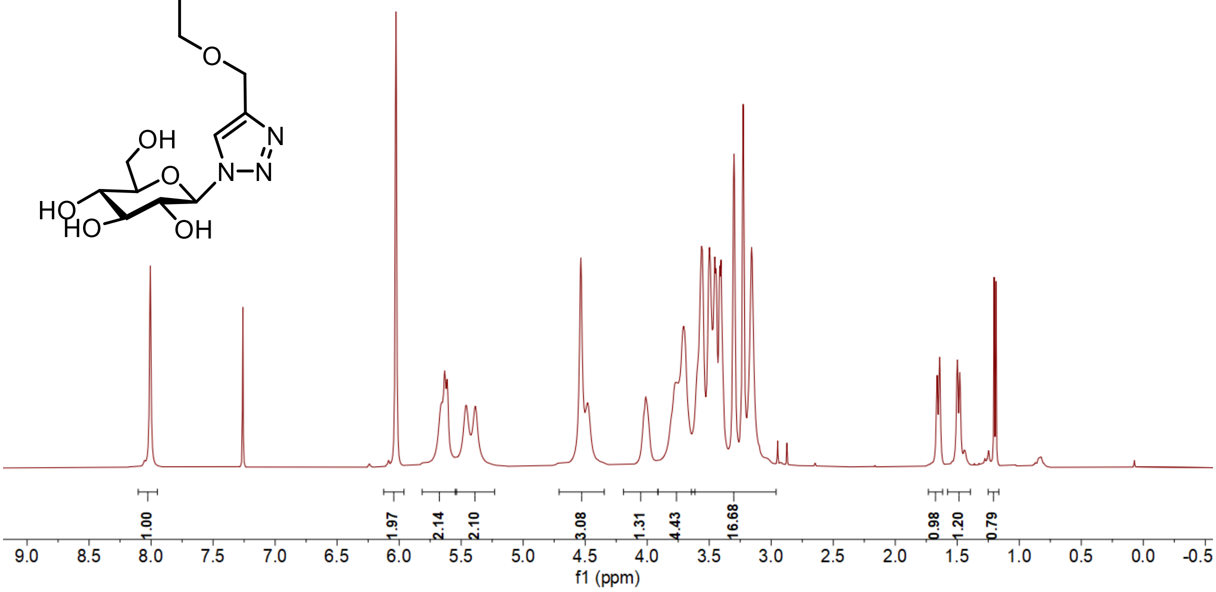

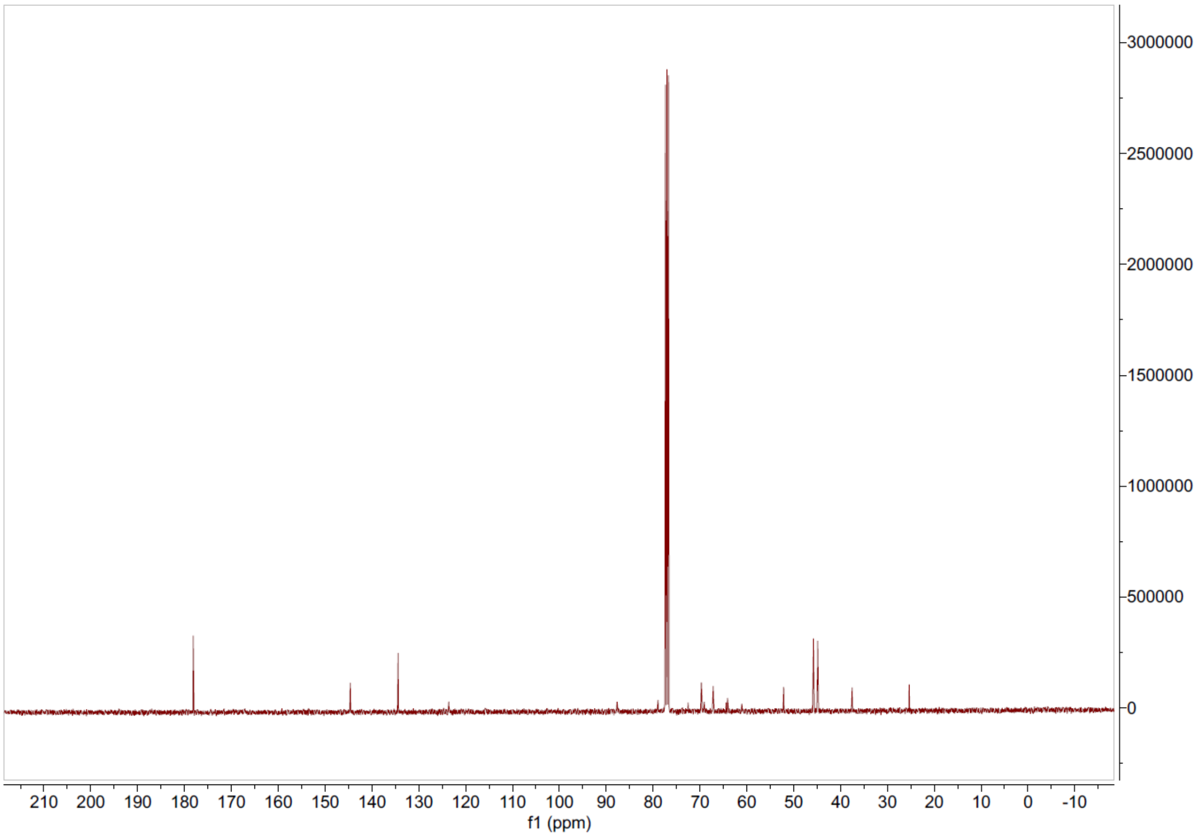

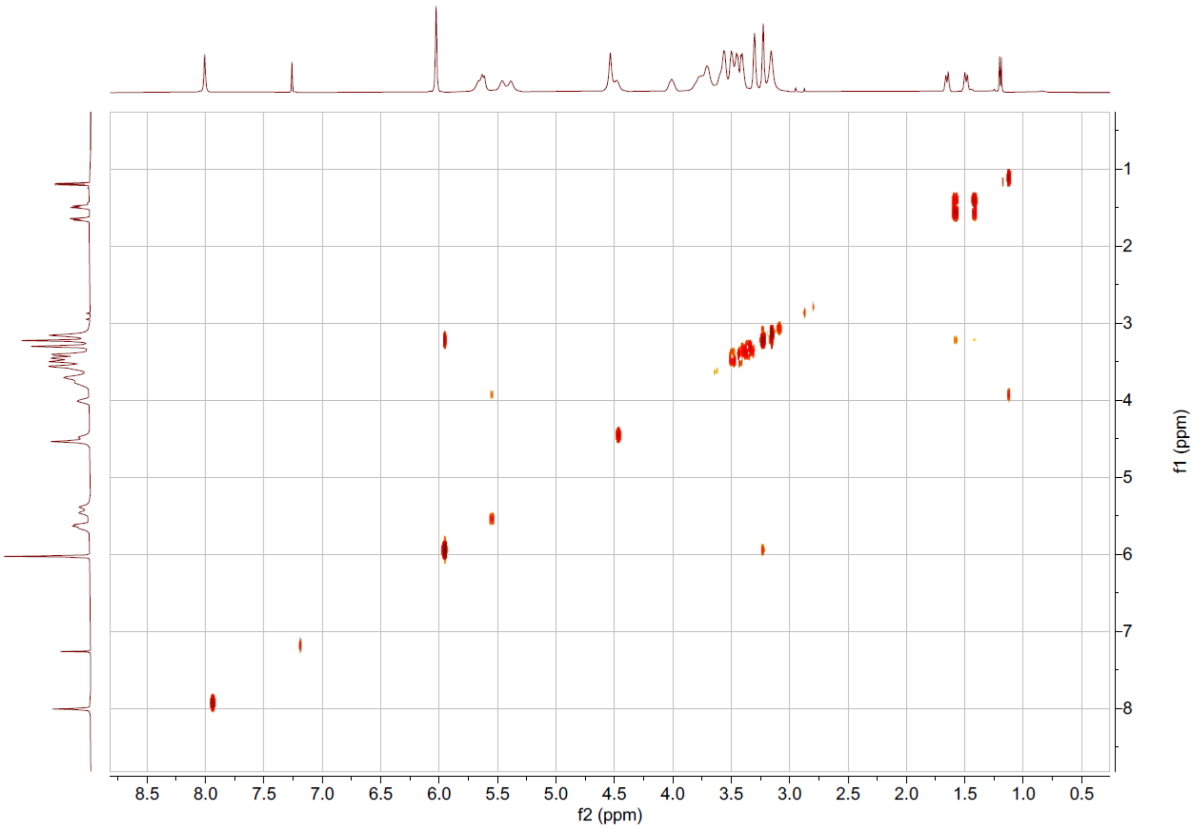
8.5
8.0
7.5
7.0
6.5
6.0
5.5
5.0
4.5
4.0
3.5
3.0
2.5
2.0
1.5
1.0
0.5
f2 (ppm)
1
2
3
4
5
6
7
8
f1 (ppm)

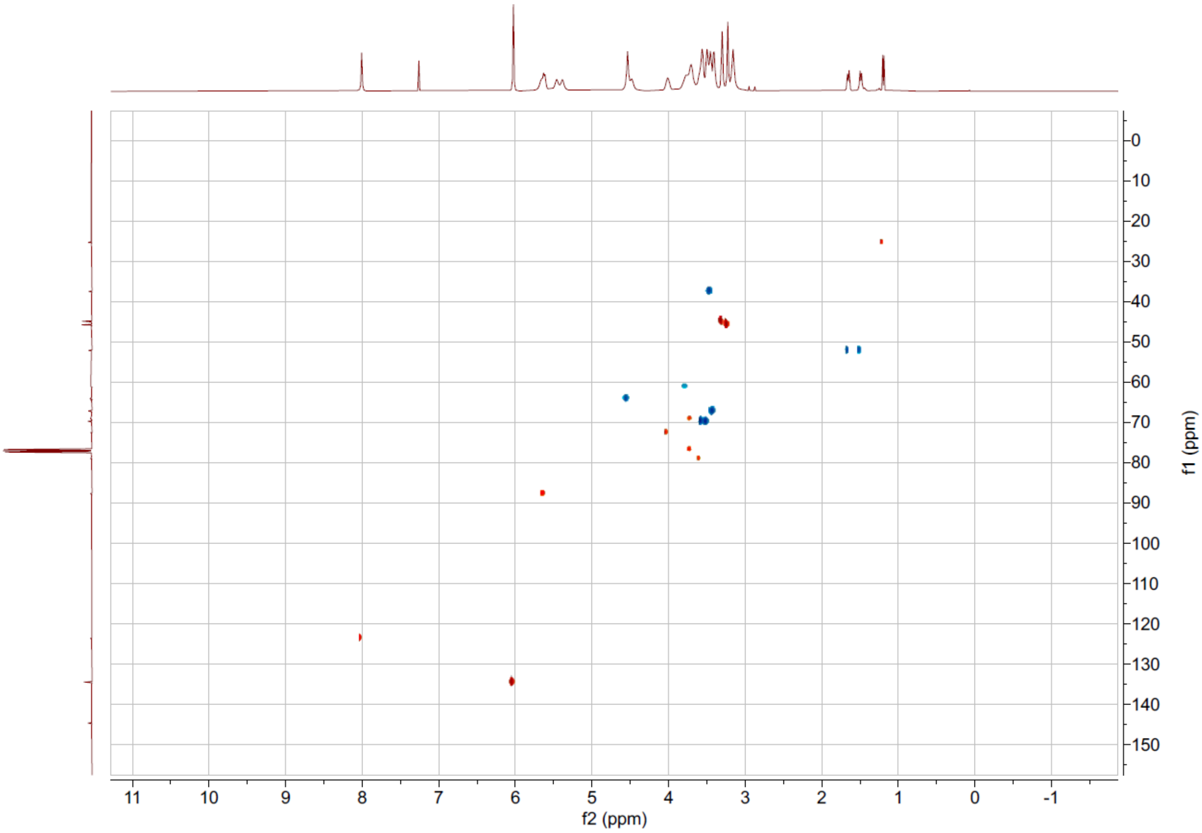
11
10
9
8
7
6
5
4
3
2
1
0
-1
f2 (ppm)
0
10
20
30
40
50
60
70
80
90
100
110
120
130
140
150
f1 (ppm)

^{1}H-NMR, ^{13}C, COSY, HSQC of Compound 21 (*endo*-β-Gal)

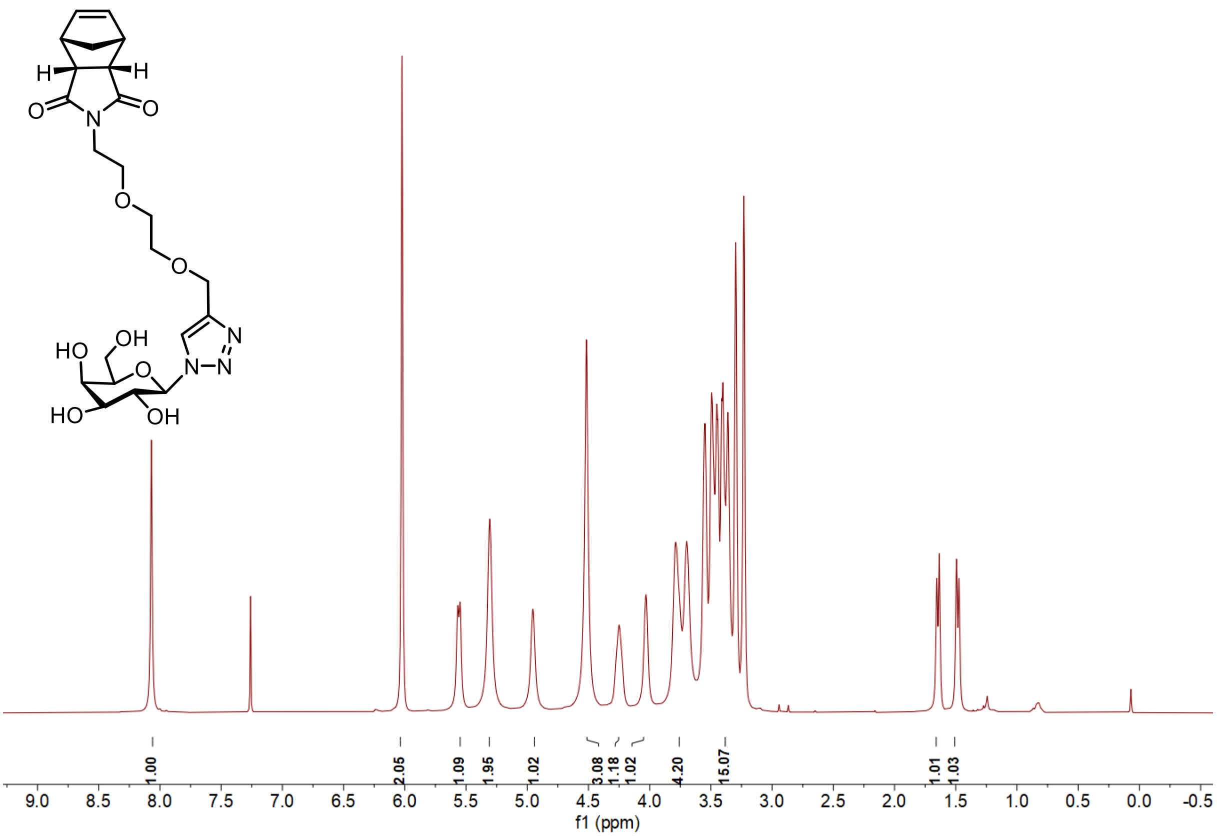

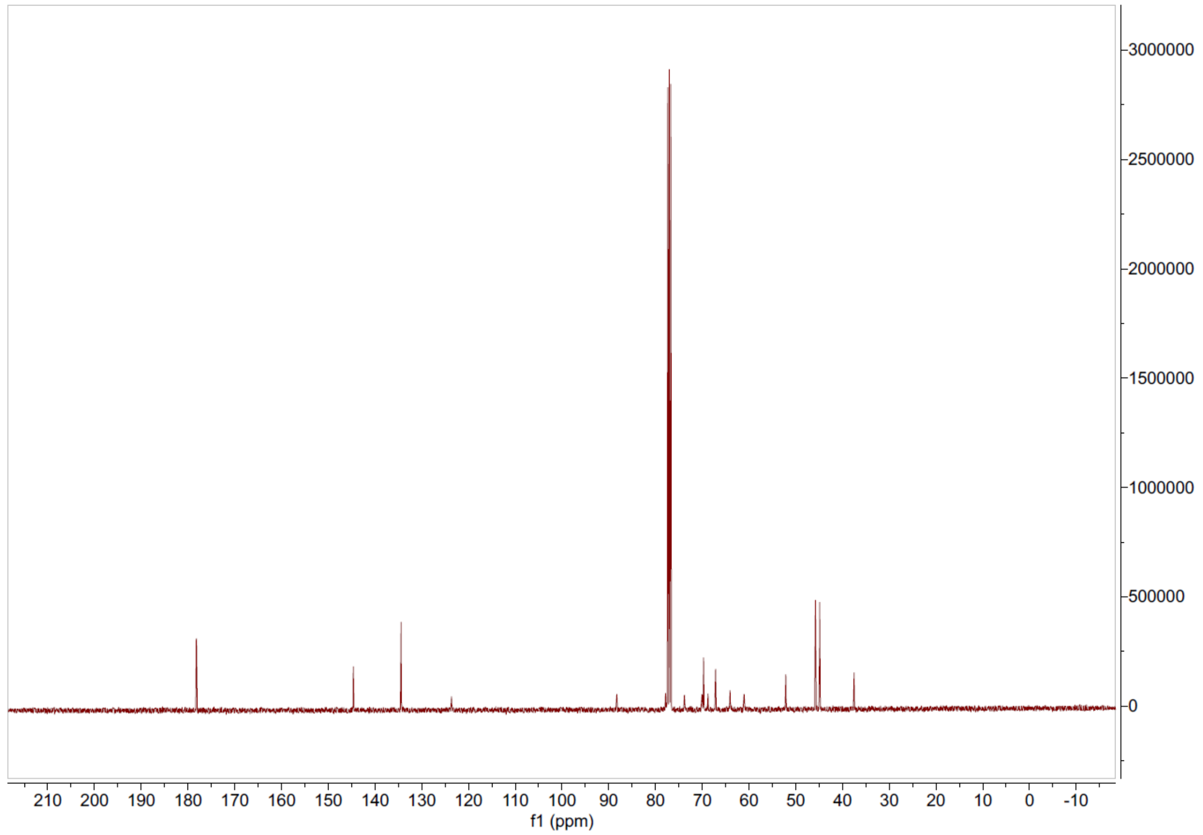

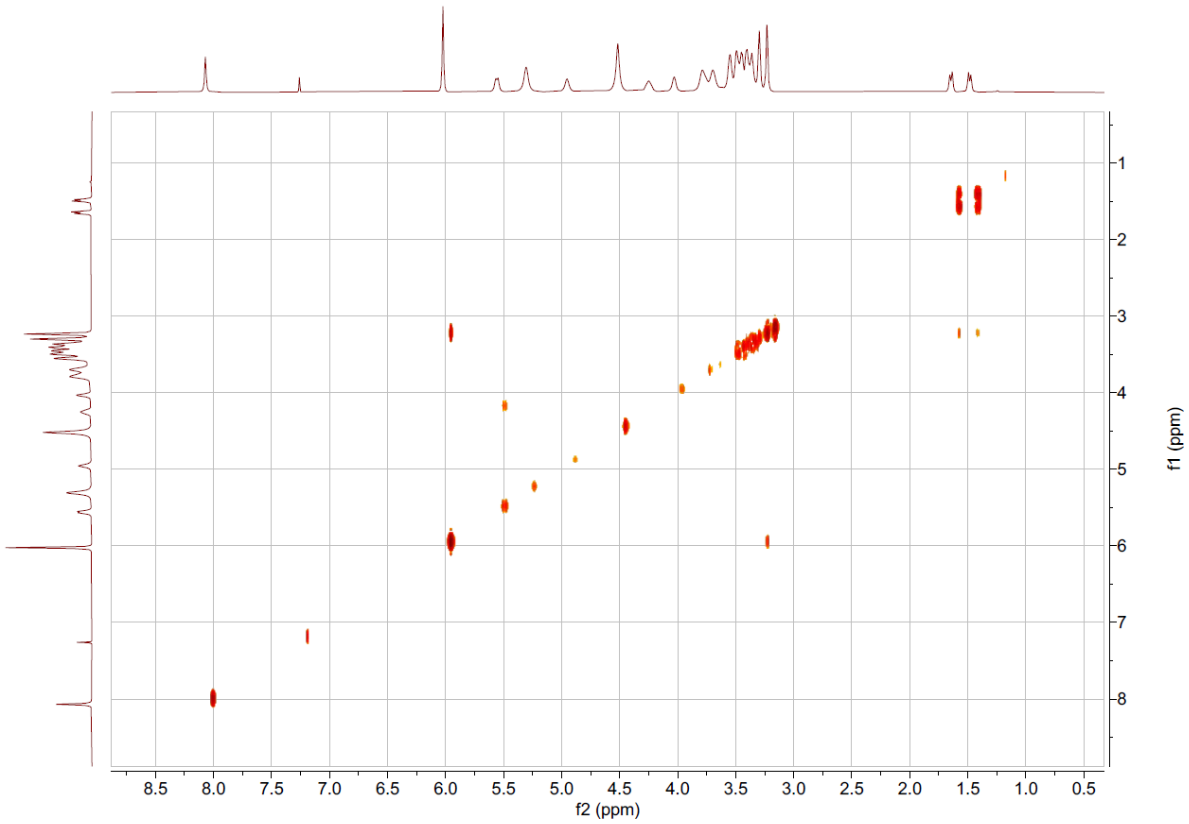

f2 (ppm)
f1 (ppm)

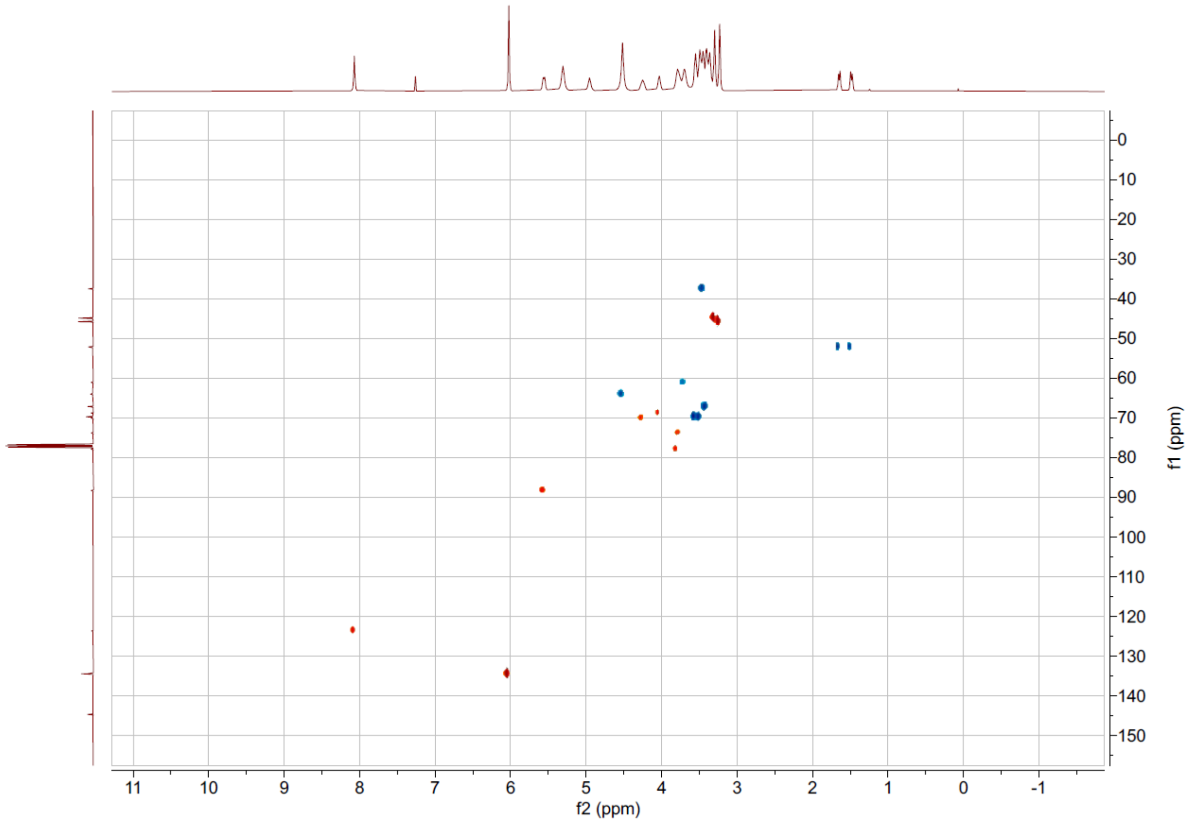

f2 (ppm)
f1 (ppm)

^{1}H-NMR, ^{13}C, COSY, HSQC of Glycopolymer – *exo*-PGP (*exo*-β-Gal)

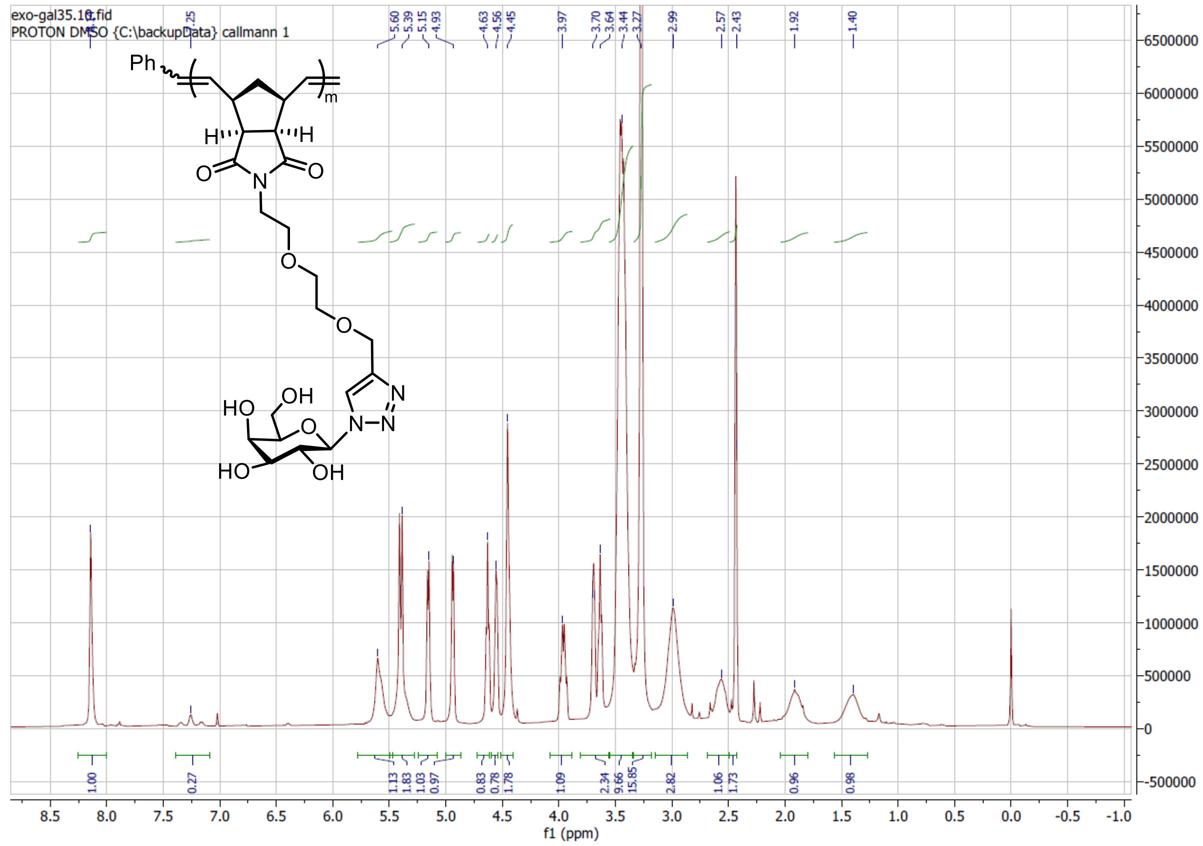

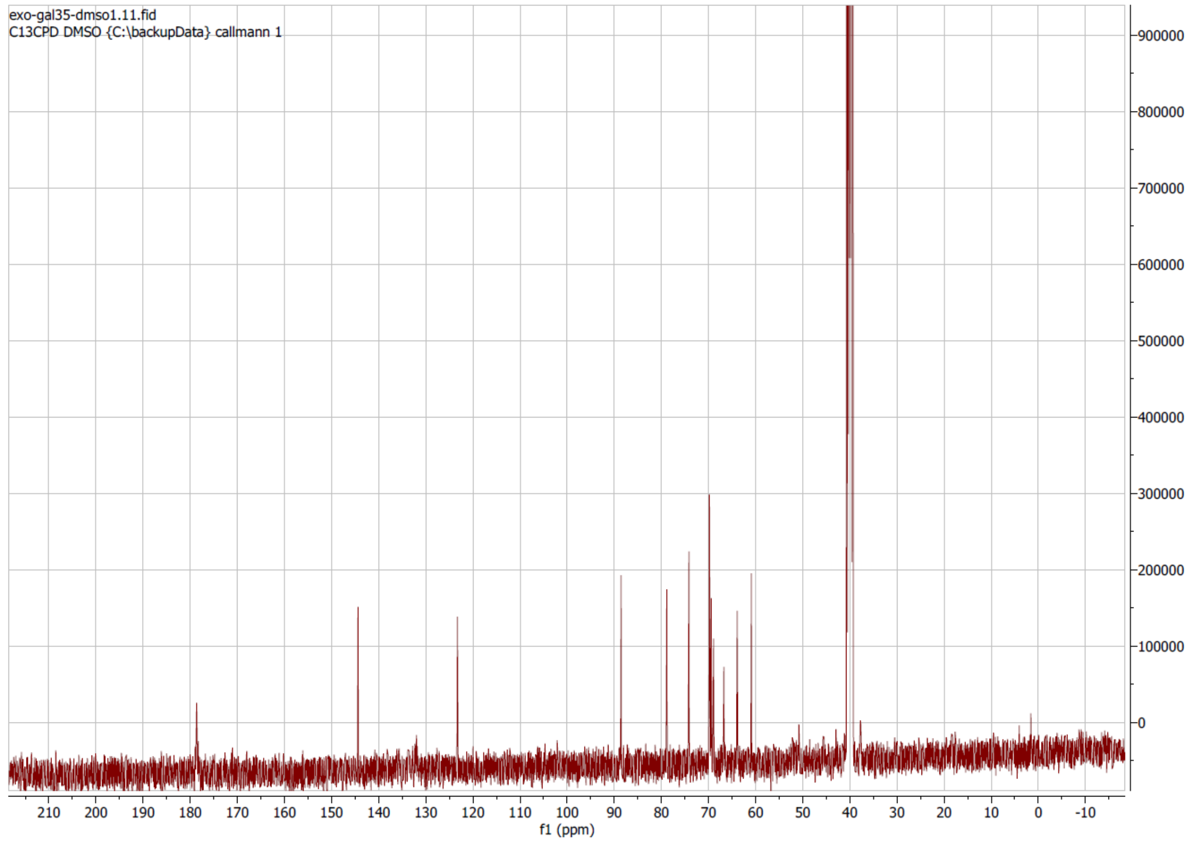

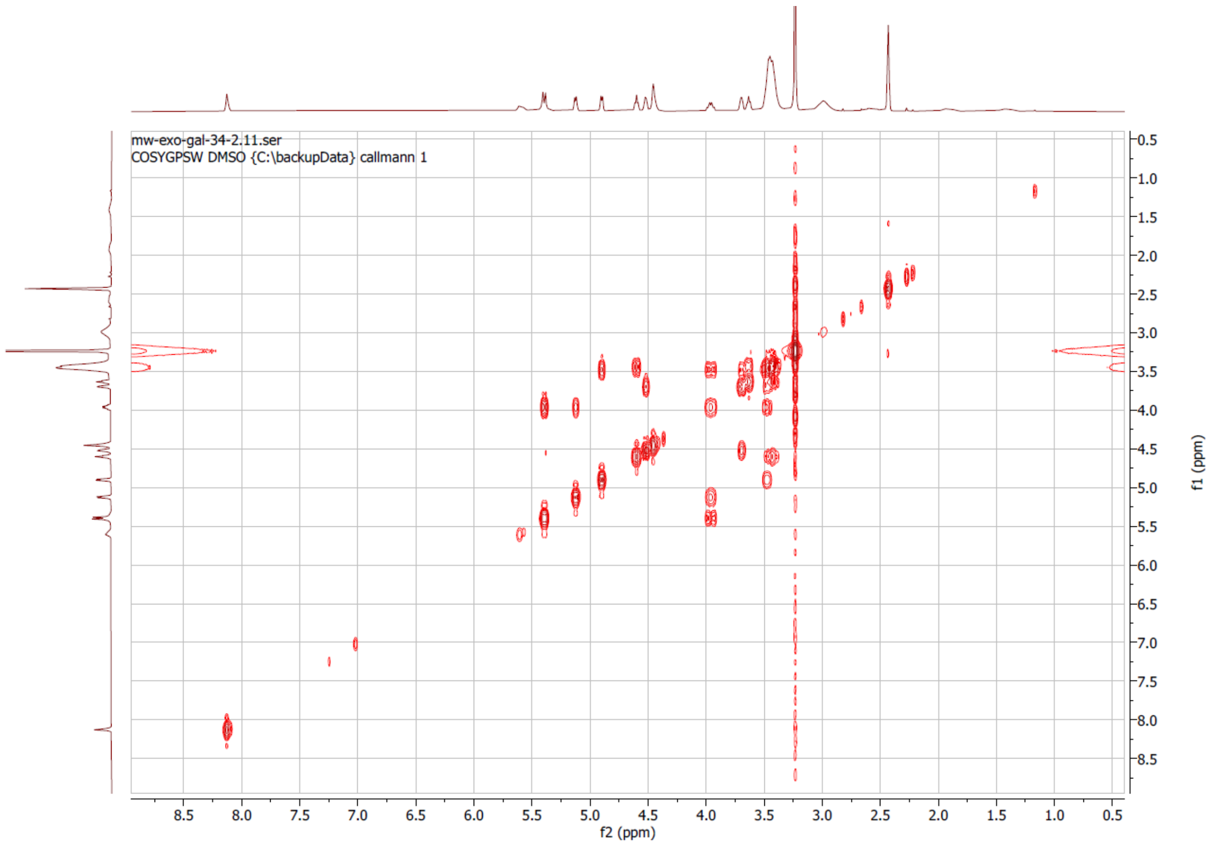
mw-exo-gal-34-2.11.ser
COSYGPSW DMSO {C:\backupData} callmann 1
f2 (ppm)
f1 (ppm)

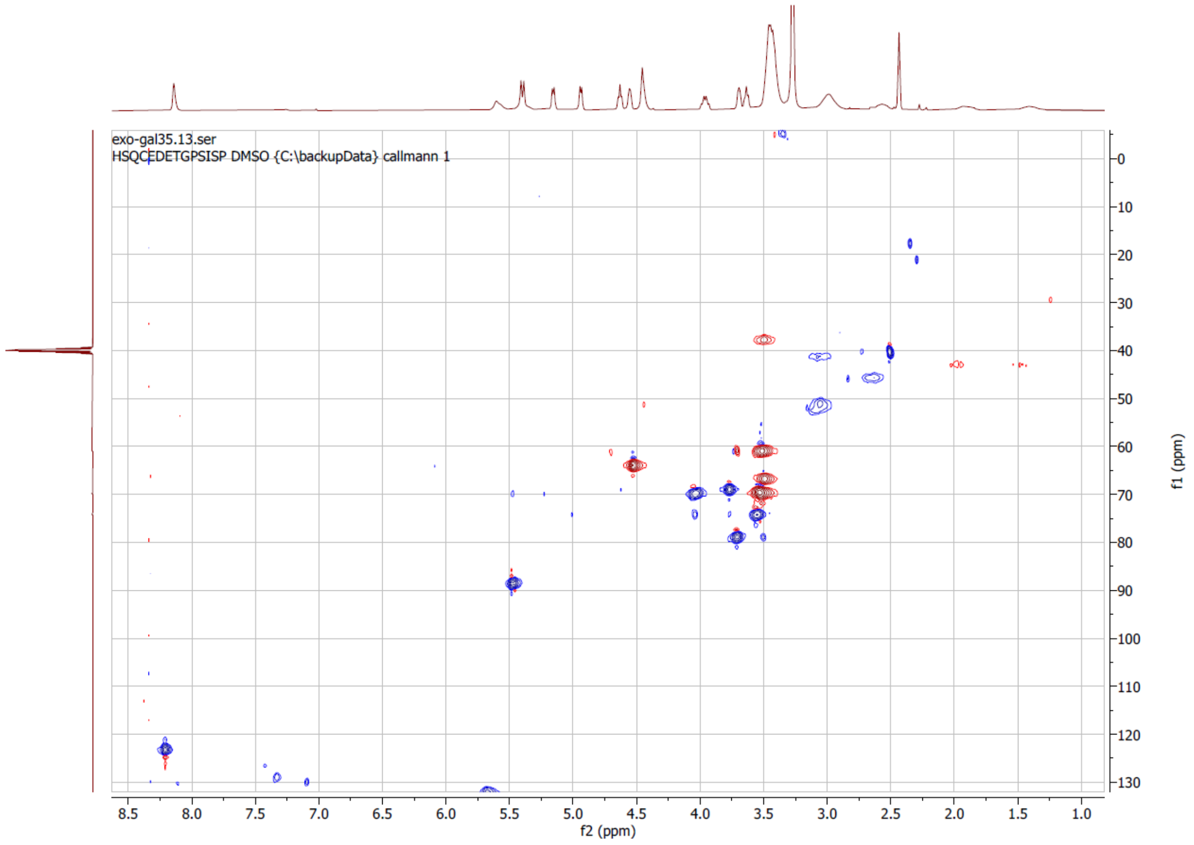
exo-gal35.13.ser
HSQCEDETGPSISP DMSO {C:\backupData} callmann 1
f2 (ppm)
f1 (ppm)

^{1}H-NMR, ^{13}C, COSY, HSQC of Glycopolymer – *endo*-PGP (*endo*-β-Gal)

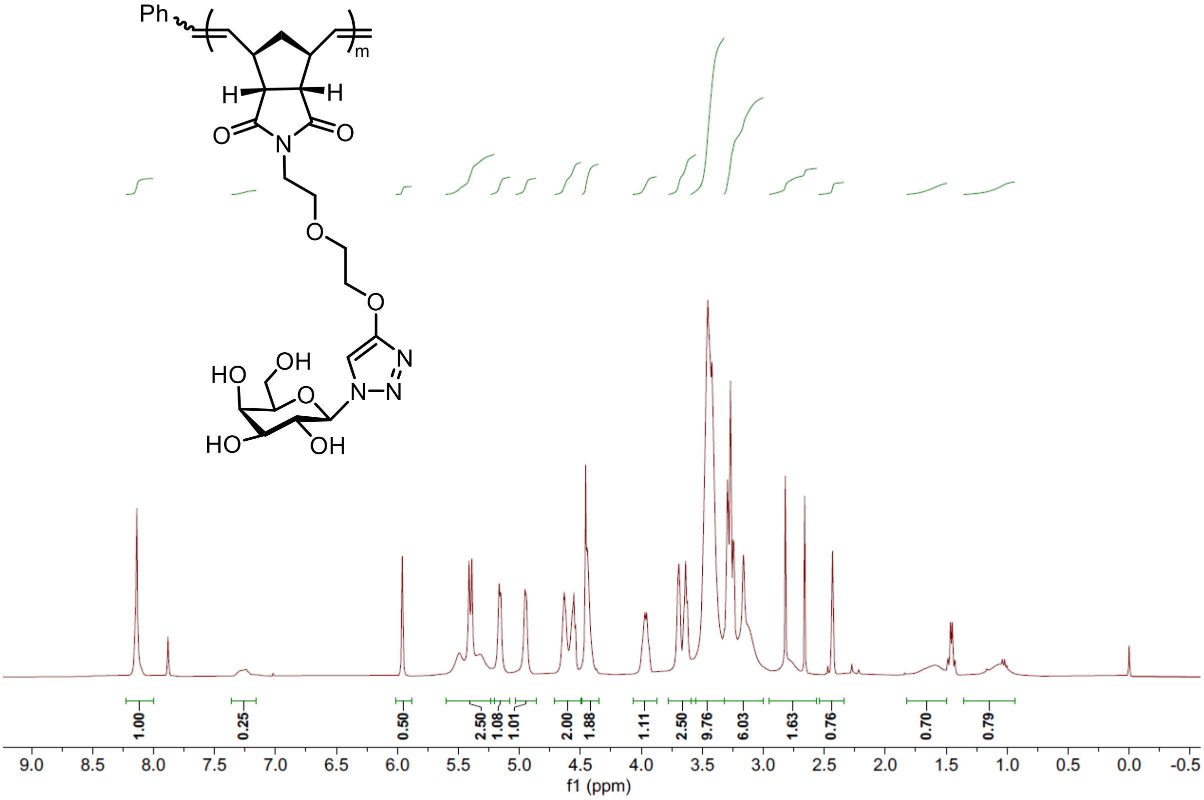

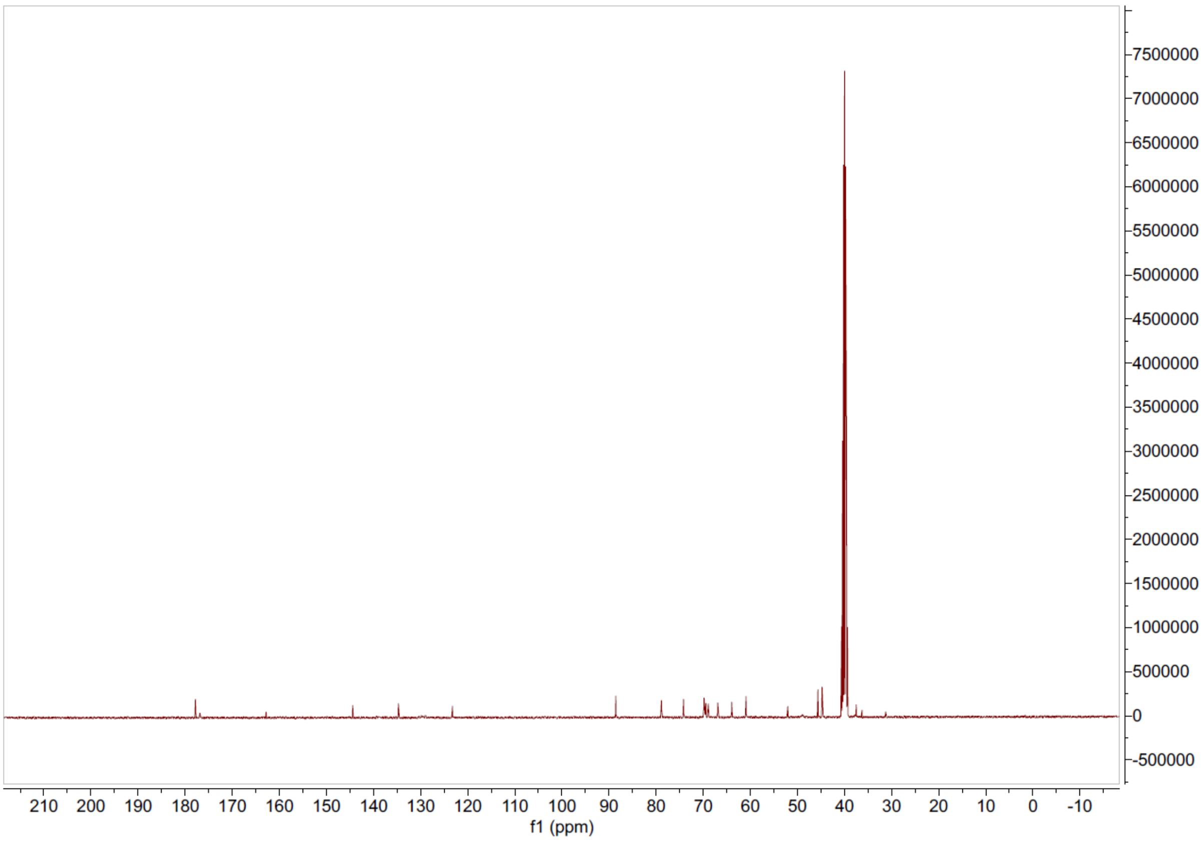

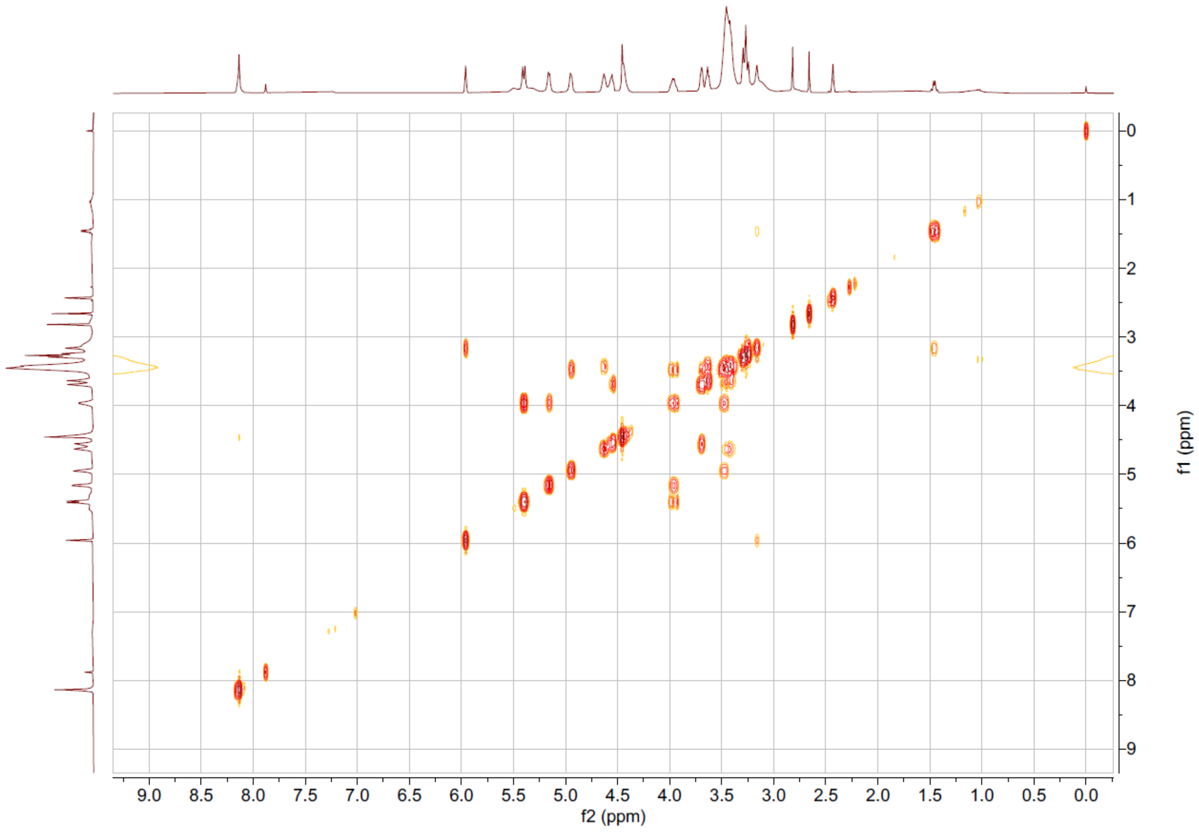
f2 (ppm)
f1 (ppm)

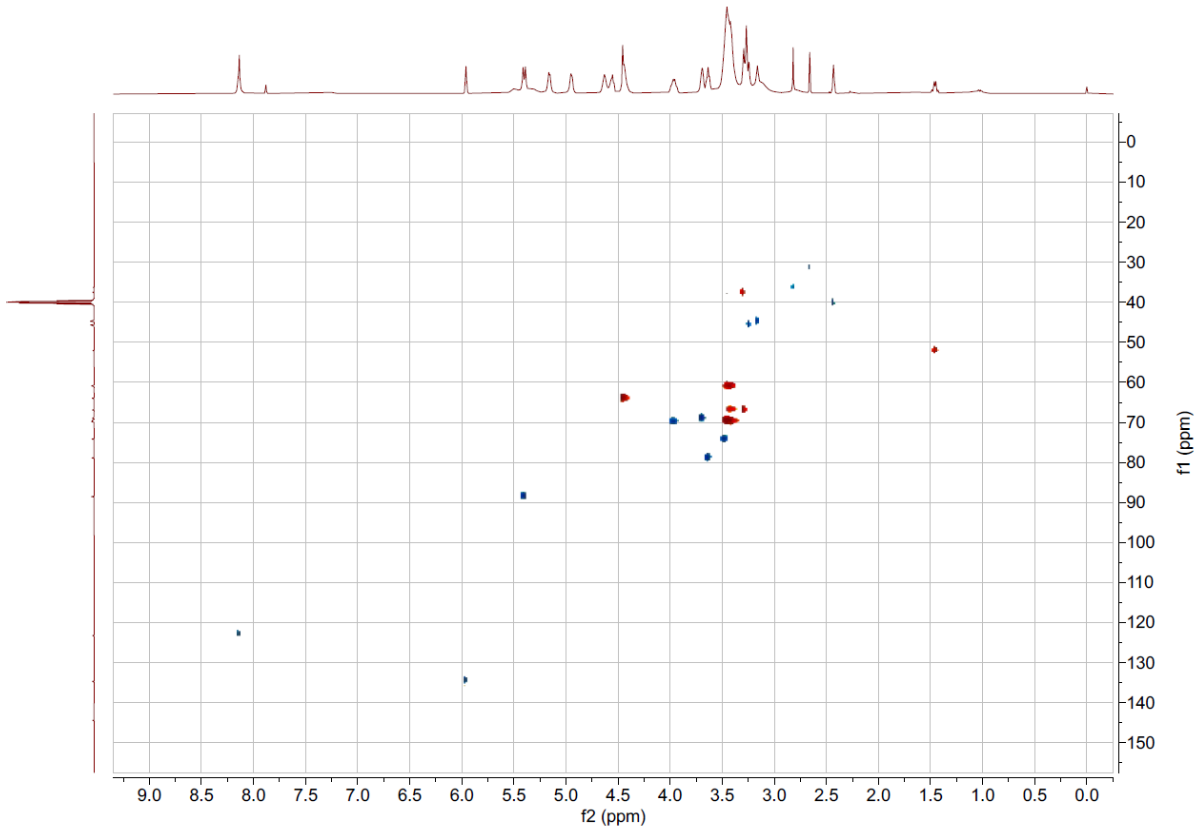
f2 (ppm)
f1 (ppm)

^{1}H-NMR *exo*-β-Glc

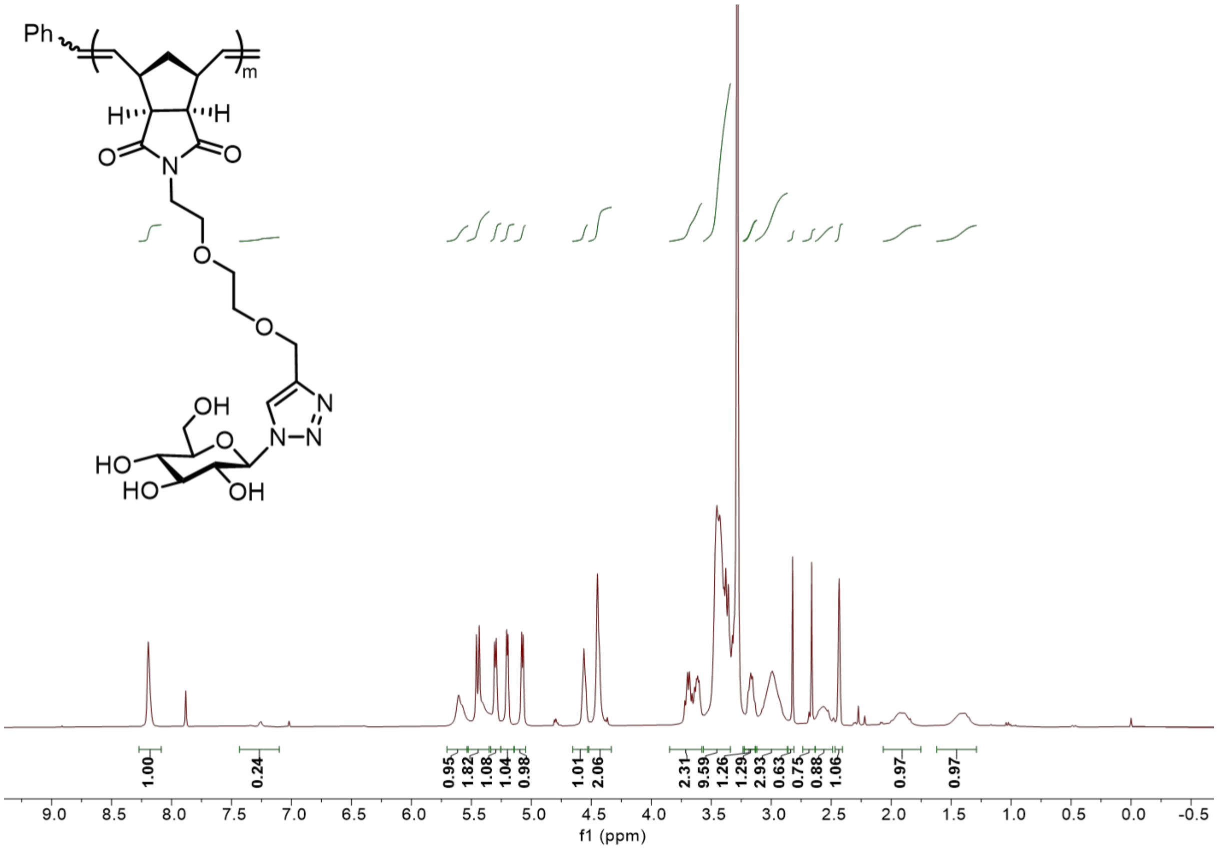

^{1}H-NMR *endo*-β-Glc

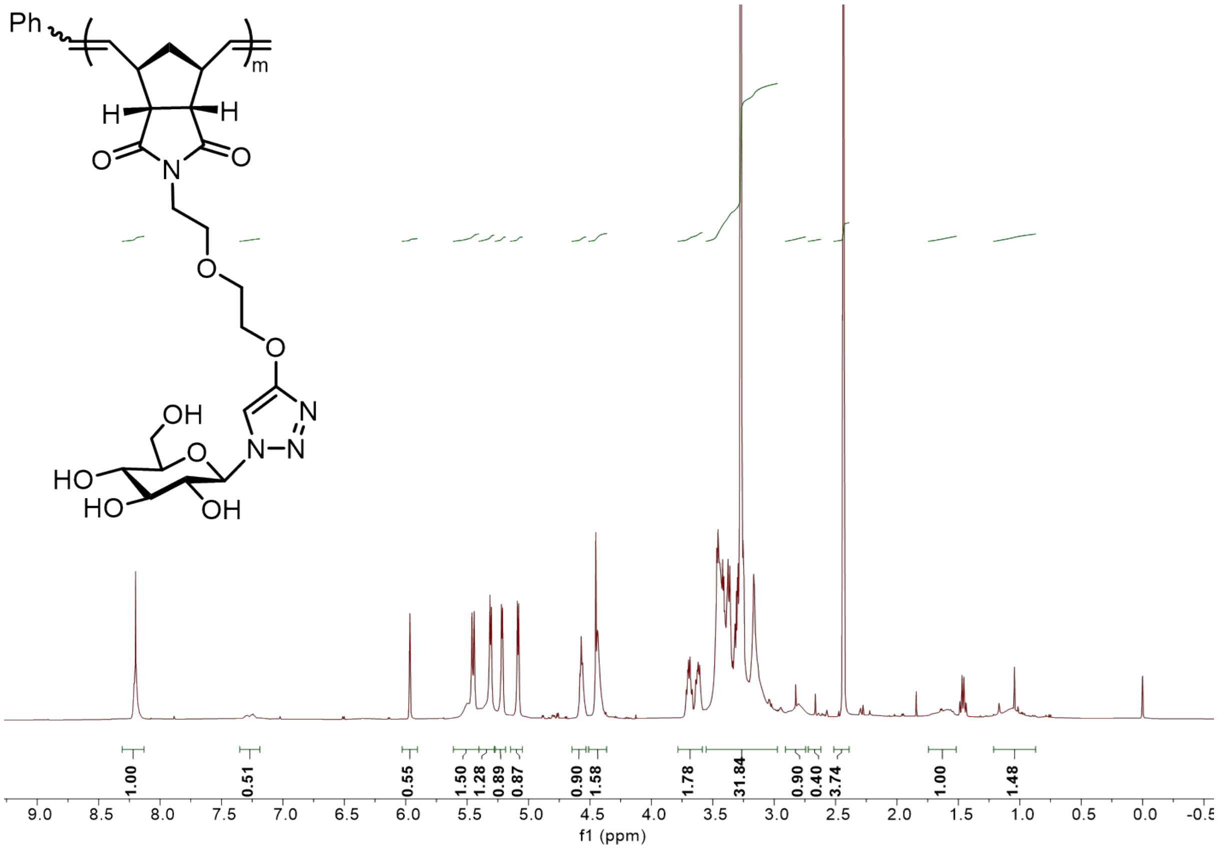

¹H-NMR *exo*-α-Man

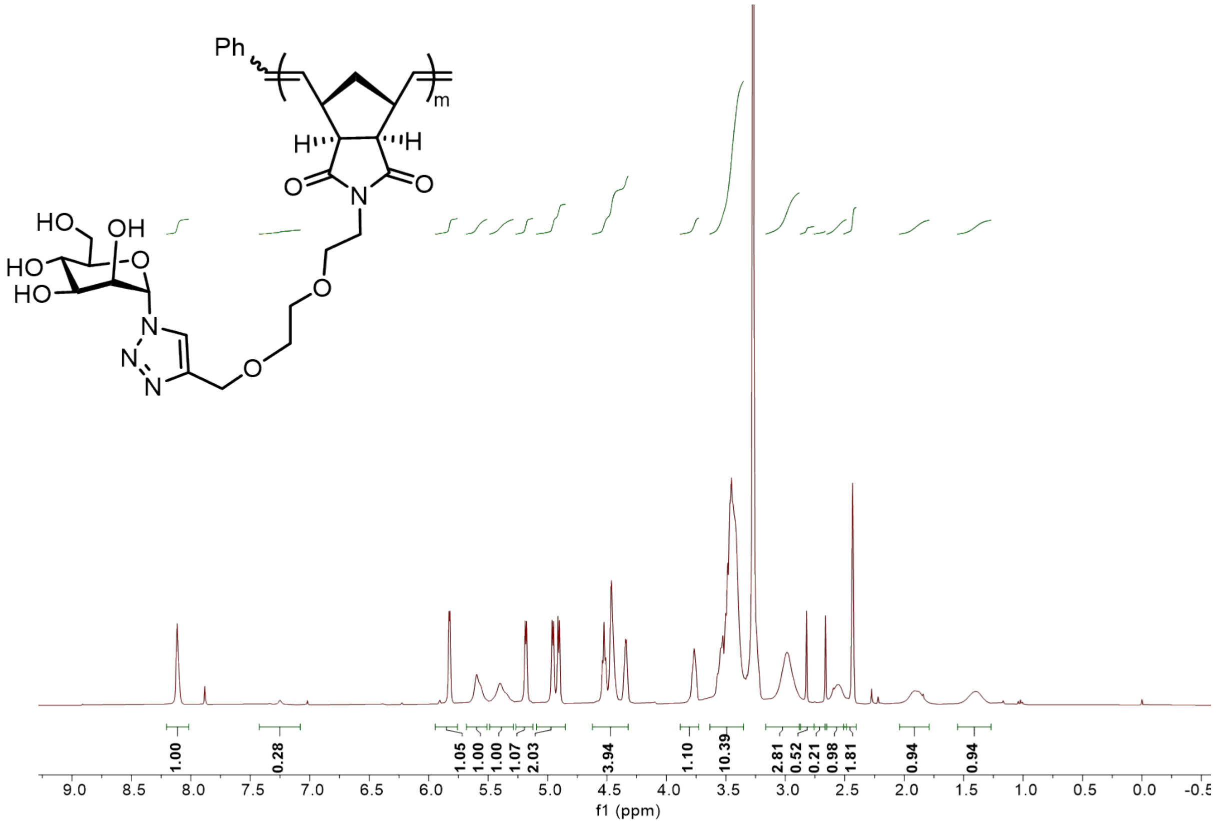

¹H-NMR *endo*-α-Man

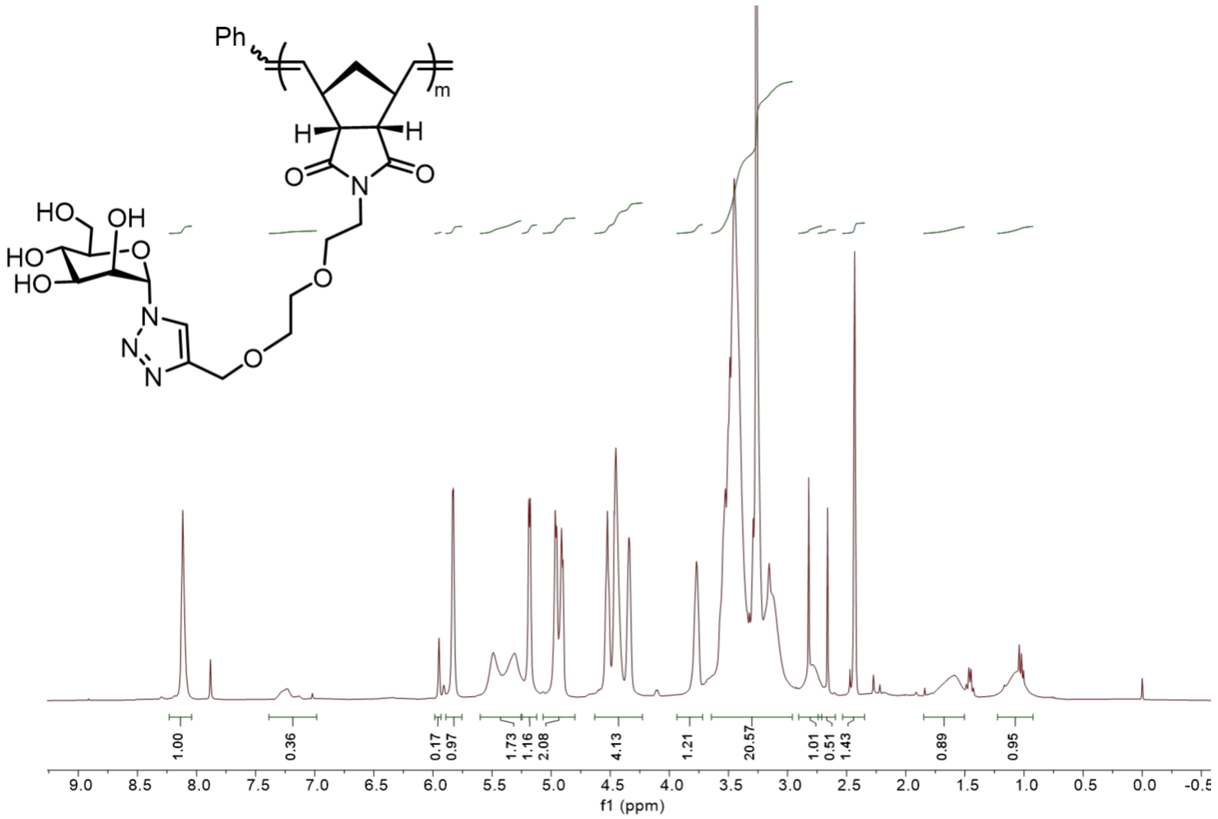

Kinetic NMR of Glycopolymer – *exo*-PGP (*exo*-OH)

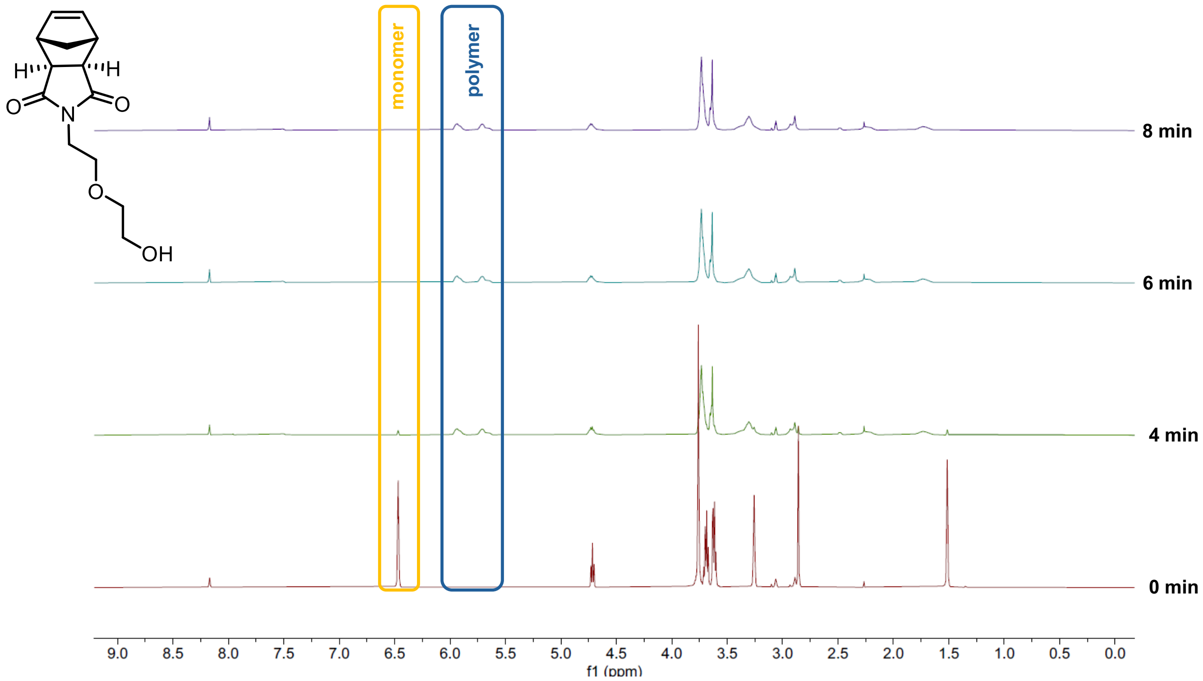

Kinetic NMR of Glycopolymer – *endo*-PGP (*endo*-OH)

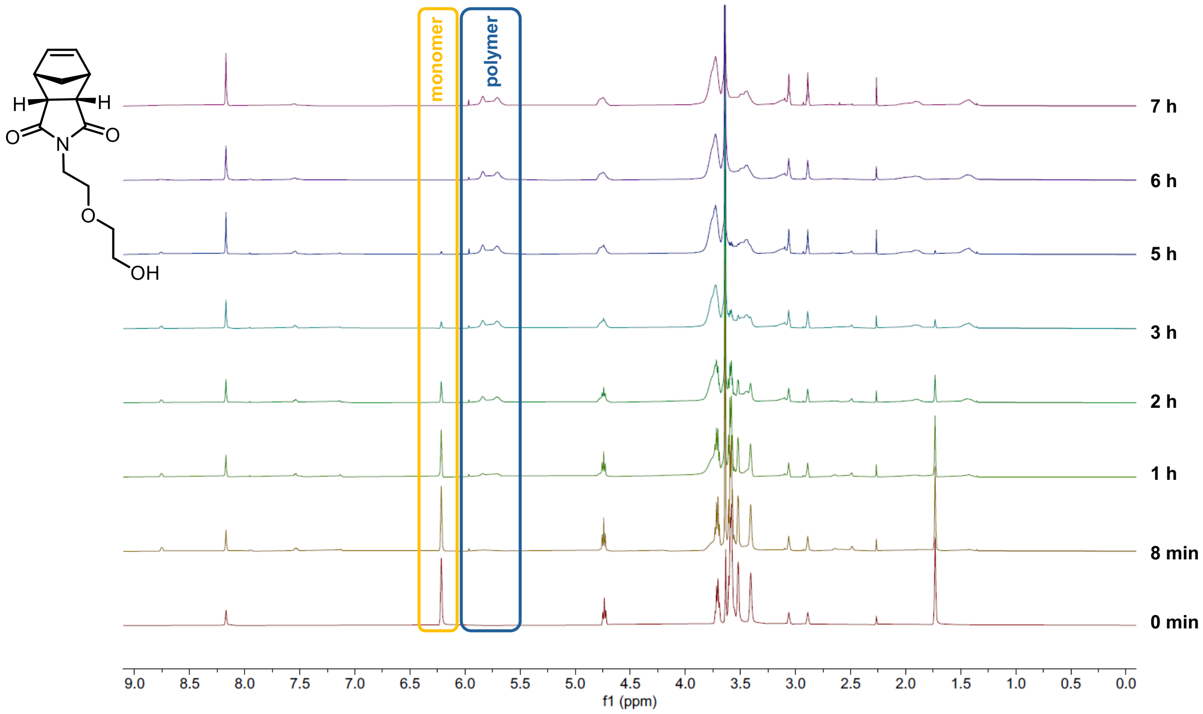

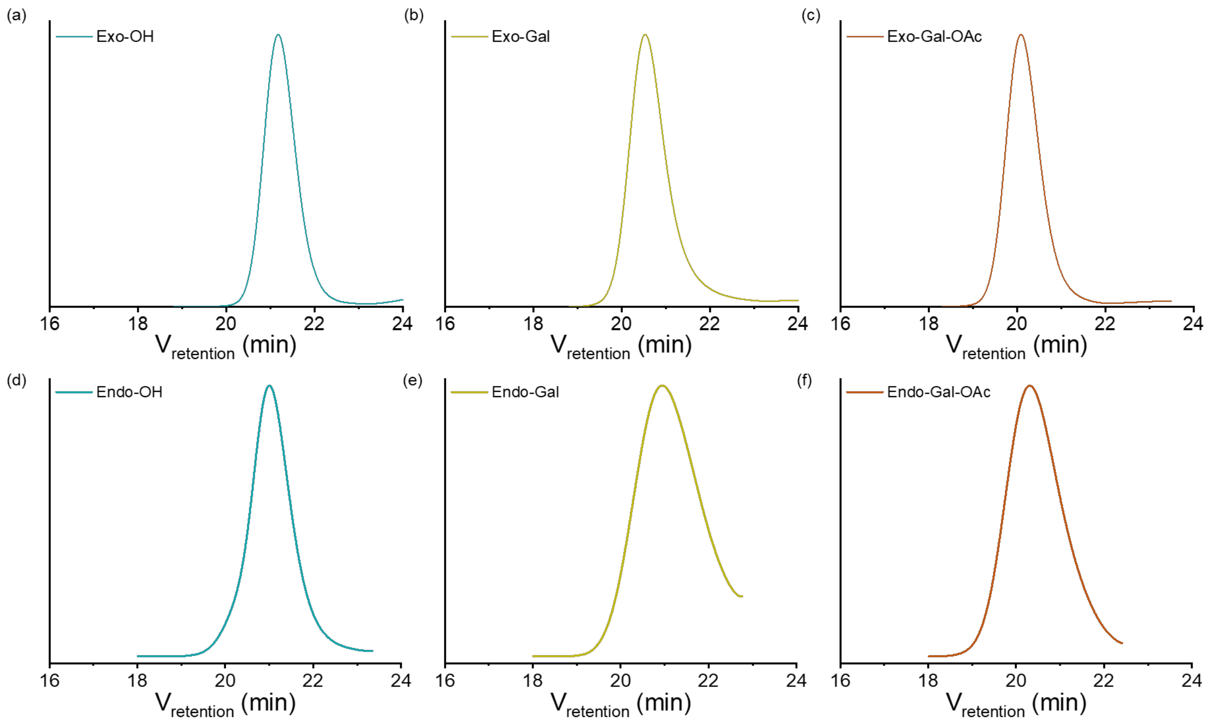

SEC-MALS traces for both *exo*- and *endo*-PGPs (OH: without Sugar, Gal: unprotected sugar, and Gal-OAc: protected sugar with OAC), polymerized at room temperature.

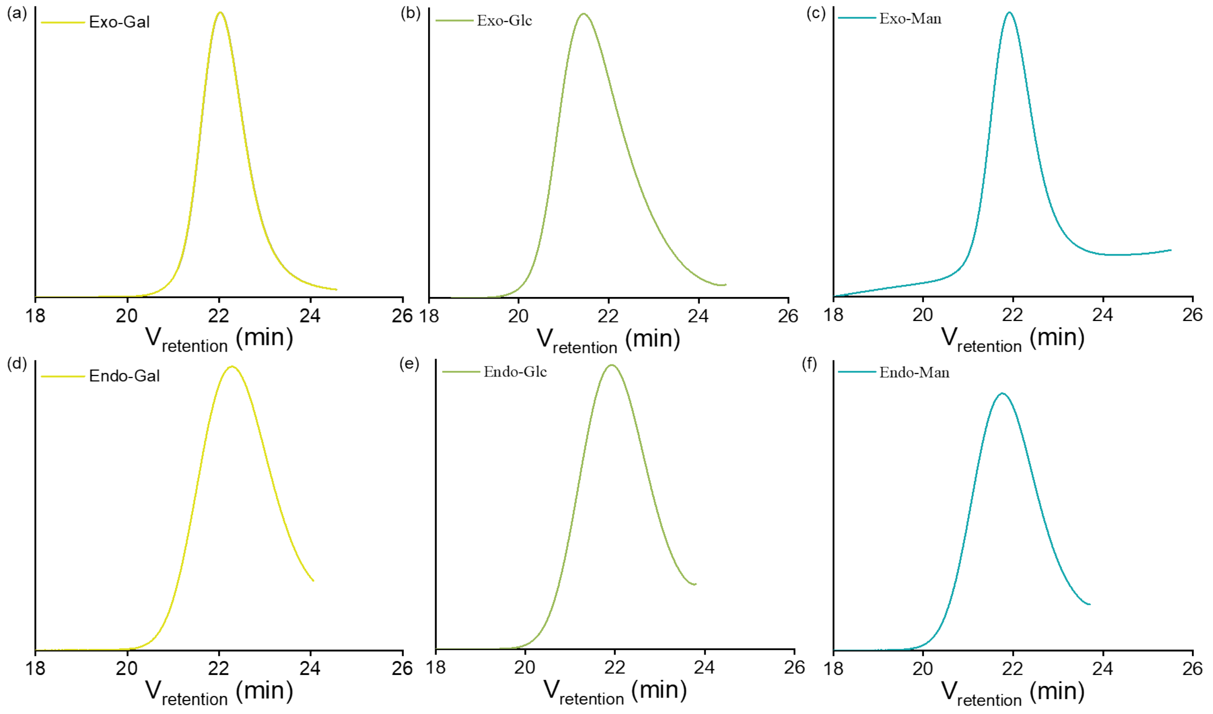

SEC-MALS traces for both *exo*- and *endo*-PGPs with different sugar epitopes: (a) *exo*-β-Gal, (b) *exo*-β-Glc, (c) *exo*-α-Man, (d) *endo*-β-Gal, (e) *endo*-β-Glc, and (f) *endo*-α-Man. **Note**: Exo-PGPs polymerized at room temperature and *endo*-PGPs polymerized at 60 °C respectively.

Cis/trans ratio of Exo- and Endo-PGPs were evaluated from ^{1}H NMR of glycopolymers.

PGPs	Trans (%)	Cis (%)
Exo-β-Gal	56.50	43.50
Endo-β-Gal	47.42	52.58
Exo-β-Glc	53.67	46.32
Endo-β-Glc	52.38	47.62
Exo-α-Man	50.00	50.00
Endo-α-Man	46.32	53.68

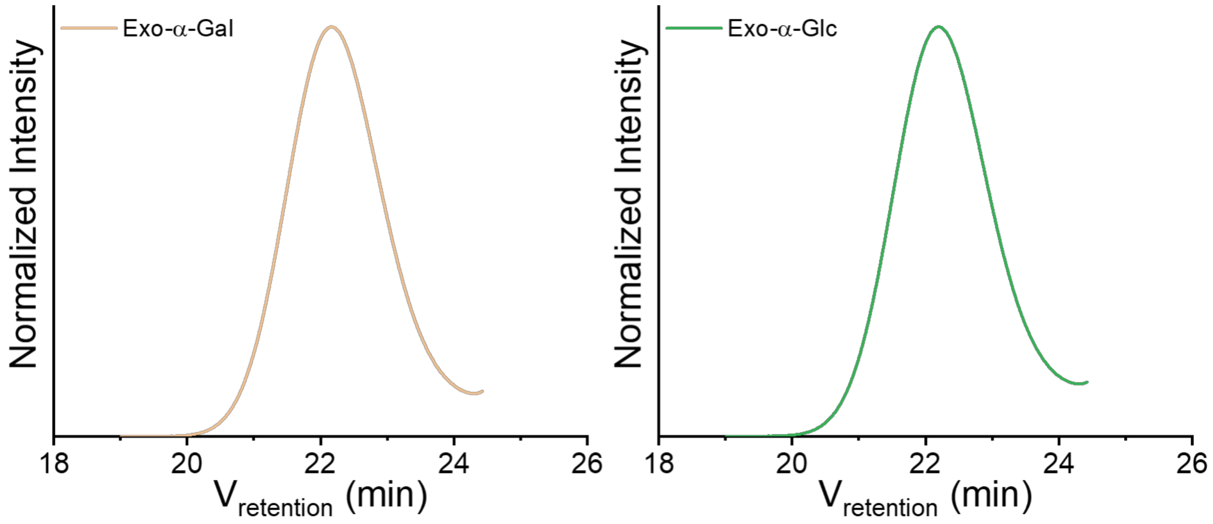

SEC-MALS traces for both *exo*-α-PGPs with different sugar epitopes: (a) *exo*-α-Gal, and (b) *exo*-α-Glc.

SEC data of alpha-PGPs, *exo*-α-Gal and *exo*-α-Glc.

PGPs	Target DP	M_n (KDa)	M_w (KDa)	Đ	Temp (°C)	DP*
Exo-α-Gal	35	16.71	56.21	1.05	r.t.	34
Exo-α-Glc	35	17.69	19.31	1.09	r.t.	35

- DP calculated as Mn/Mw,monomer

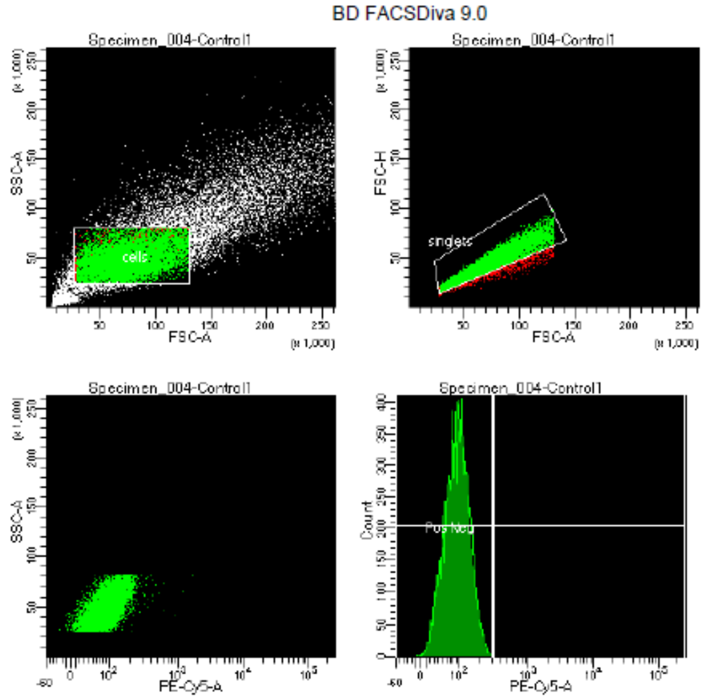

Flow cytometry data of control and shows gates used for single cells and %positive and %negative.

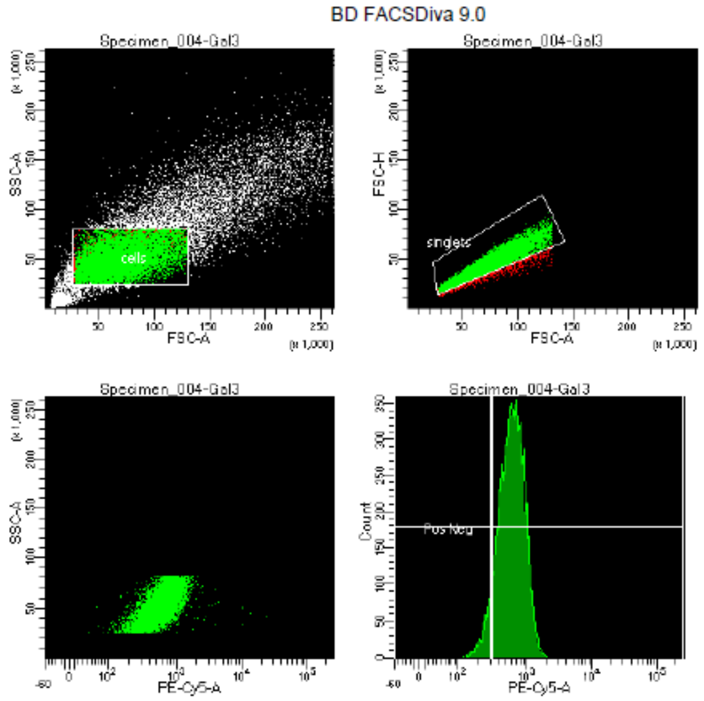

Flow cytometry data of *exo*-β-Gal and shows gates used for single cells and %positive and %negative.

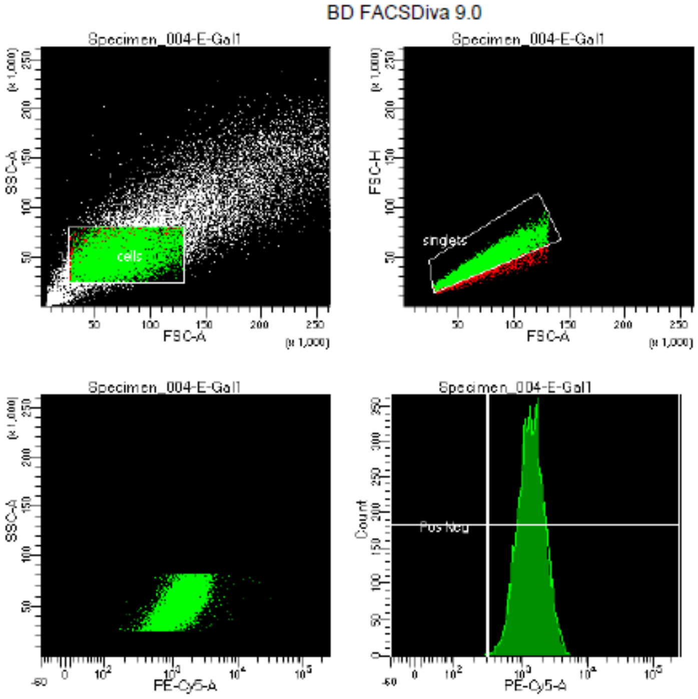

Flow cytometry data of *endo*-β-Gal and shows gates used for single cells and %positive and %negative.

References and Notes

(1) Williams, C.A.; Stone, D.J.; Joshi, S.Y.; Yilmaz, G.; Far-zeen, P.; Jeon, S.; Harris-Ryden, Z.; Becer, C.R.; Deshmukh, S.A.; and Callmann, C.E.; Systematic Evaluation of Macromolecular Carbohydrate-Lectin Recognition Using Precision Glycopolymers. Biomacromolecules **2024**, 25, 12, 7985–7994

(2) Jeon, S.; Haynie, T.; Chung, S.; Callmann, C. E.; Bioinspired, Carbohydrate-Containing Polymers Efficiently and Reversibly Sequester Heavy Metals. ACS Cent. Sci. **2024**, 10, 9, 1782–1788

(3) Abraham, M. J.; Murtola, T.; Schulz, R.; Páll, S.; Smith, J. C.; Hess, B.; Lindahl, E. GROMACS: High Performance Molecular Simulations through Multi-Level Parallelism from Laptops to Supercomputers. *SoftwareX* **2015**, *1-2*, 19–25.

(4) Bowers, K. J.; Chow, D. E.; Xu, H.; Dror, R. O.; Eastwood, M. P.; Gregersen, B. A.; Klepeis, J. L.; Kolossvary, I.; Moraes, M. A.; Sacerdoti, F. D.; *et al.* Scalable Algorithms for Molecular Dynamics Simulations on Commodity Clusters. In *ACM/IEEE SC 2006 Conference (SC'06)*; IEEE, 2006.

(5) Vanommeslaeghe, K.; Hatcher, E.; Acharya, C.; Kundu, S.; Zhong, S.; Shim, J.; Darian, E.; Guvench, O.; Lopes, P.; Vorobyov, I.; *et al.* CHARMM General Force Field: A Force Field for Drug-like Molecules Compatible with the CHARMM All-Atom Additive Biological Force Fields. *J Comput Chem* **2010**, *31*, 671–690.

(6) Guvench, O.; Greene, S. N.; Kamath, G.; Brady, J. W.; Venable, R. M.; Pastor, R. W.; Mackerell, A. D., Jr. Additive Empirical Force Field for Hexopyranose Monosaccharides. *J Comput Chem* **2008**, *29*, 2543–2564.

(7) Darden, T.; York, D.; Pedersen, L. Particle Mesh Ewald: AnN·log(N) Method for Ewald Sums in Large Systems. *J. Chem. Phys.* **1993**, *98*, 10089–10092.

(8) Steinbach, P. J.; Brooks, B. R. New Spherical-Cutoff Methods for Long-Range Forces in Macromolecular Simulation. *J. Comput. Chem.* **1994**, *15*, 667–683.

(9) Hess, B.; Bekker, H.; Berendsen, H. J. C.; Fraaije, J. G. E. LINCS: A Linear Constraint Solver for Molecular Simulations. *Journal of Computational Chemistry*, 1997, *18*, 1463–1472.

(10) Becker, O. M.; MacKerell, A. D., Jr; Roux, B.; Watanabe, M. *Computational Biochemistry and Biophysics*; CRC Press, 2001.

(11) Brooks, C. L. *Advances in Chemical Physics, Volume 71, Proteins: A Theoretical Perspective of Dynamics, Structure, and Thermodynamics*; 1988.

(12) Miyamoto, S.; Kollman, P. A. Settle: An Analytical Version of the SHAKE and RATTLE Algorithm for Rigid Water Models. *J. Comput. Chem.* **1992**, *13*, 952–962.

(13) Spreiter, Q.; Walter, M. Classical Molecular Dynamics Simulation with the Velocity Verlet Algorithm at Strong External Magnetic Fields. *J. Comput. Phys.* **1999**, *152*, 102–119.

(14) Hoover, W. G. Canonical Dynamics: Equilibrium Phase-Space Distributions. *Phys Rev A Gen Phys* **1985**, *31*, 1695–1697.

(15) Humphrey, W.; Dalke, A.; Schulten, K. VMD: Visual Molecular Dynamics. *J. Mol. Graph.* **1996**, *14*, 33–38, 27–28.

(16) Richards, S.-J.; Jones, M. W.; Hunaban, M.; Haddleton, D. M.; Gibson, M. I. Angew. Chem. Int. Ed. 2012, 51 (31), 7812–7816.

(17) Austin G. Kruger, Spencer D. Brucks, Tao Yan, Gerardo Cárcarmo-Oyarce, Yuan Wei, Deborah H. Wen, Dayanne R. Carvalho, Michael J. A. Hore, Katharina Ribbeck, Richard R. Schrock, and Laura L. Kiessling. ACS Central Science **2021** 7 (4), 624-630
